# Acute Kidney Injury Defined by Fluid-Corrected Creatinine in Premature Neonates: A Secondary Analysis of the PENUT Randomized Clinical Trial

**DOI:** 10.1001/jamanetworkopen.2023.28182

**Published:** 2023-08-10

**Authors:** Michelle C. Starr, Russell L. Griffin, Matthew W. Harer, Danielle E. Soranno, Katja M. Gist, Jeffrey L. Segar, Shina Menon, Lindsey Gordon, David J. Askenazi, David T. Selewski

**Affiliations:** 1 Division of Nephrology, Department of Pediatrics, Indiana University School of Medicine, Indianapolis; 2 Pediatric and Adolescent Comparative Effectiveness Research, Department of Pediatrics, Indiana University School of Medicine, Indianapolis; 3 Department of Epidemiology, University of Alabama at Birmingham; 4 Division of Neonatology, Department of Pediatrics, University of Wisconsin School of Medicine and Public Health, Madison; 5 Department of Bioengineering, Purdue University, West Lafayette, Indiana; 6 Division of Cardiology, Department of Pediatrics, Cincinnati Children’s Hospital Medical Center, University of Cincinnati College of Medicine, Cincinnati, Ohio; 7 Division of Neonatology, Departments of Pediatrics and Physiology, Medical College of Wisconsin, Milwaukee; 8 Division of Nephrology, University of Washington and Seattle Children’s Hospital, Seattle; 9 Division of Nephrology, Department of Pediatrics, University of Alabama at Birmingham; 10 Division of Nephrology, Department of Pediatrics, Medical University of South Carolina, Charleston

## Abstract

**Importance:**

Acute kidney injury (AKI) and disordered fluid balance are common in premature neonates; a positive fluid balance dilutes serum creatinine, and a negative fluid balance concentrates serum creatinine, both of which complicate AKI diagnosis. Correcting serum creatinine for fluid balance may improve diagnosis and increase diagnostic accuracy for AKI.

**Objective:**

To determine whether correcting serum creatinine for fluid balance would identify additional neonates with AKI and alter the association of AKI with short-term and long-term outcomes.

**Design, Setting, and Participants:**

This study was a post hoc cohort analysis of the Preterm Erythropoietin Neuroprotection Trial (PENUT), a phase 3, randomized clinical trial of erythropoietin, conducted at 19 academic centers and 30 neonatal intensive care units in the US from December 2013 to September 2016. Participants included extremely premature neonates born at less than 28 weeks of gestation. Data analysis was conducted in December 2022.

**Exposure:**

Diagnosis of fluid-corrected AKI during the first 14 postnatal days, calculated using fluid-corrected serum creatinine (defined as serum creatinine multiplied by fluid balance [calculated as percentage change from birth weight] divided by total body water [estimated 80% of birth weight]).

**Main Outcomes and Measures:**

The primary outcome was invasive mechanical ventilation on postnatal day 14. Secondary outcomes included death, hospital length of stay, and severe bronchopulmonary dysplasia (BPD). Categorical variables were analyzed by proportional differences with the χ^2^ test or Fisher exact test. The *t* test and Wilcoxon rank sums test were used to compare continuous and ordinal variables, respectively. Odds ratios (ORs) and 95% CIs for the association of exposure with outcomes of interest were estimated using unconditional logistic regression models.

**Results:**

A total of 923 premature neonates (479 boys [51.9%]; median [IQR] birth weight, 801 [668-940] g) were included, of whom 215 (23.3%) received a diagnosis of AKI using uncorrected serum creatinine. After fluid balance correction, 13 neonates with AKI were reclassified as not having fluid-corrected AKI, and 111 neonates previously without AKI were reclassified as having fluid-corrected AKI (ie, unveiled AKI). Therefore, fluid-corrected AKI was diagnosed in 313 neonates (33.9%). Neonates with unveiled AKI were similar in clinical characteristics to those with AKI whose diagnoses were made with uncorrected serum creatinine. Compared with those without AKI, neonates with unveiled AKI were more likely to require ventilation (81 neonates [75.0%] vs 254 neonates [44.3%] and have longer hospital stays (median [IQR], 102 [84-124] days vs 90 [71-110] days). In multivariable analysis, a diagnosis of fluid-corrected AKI was associated with increased odds of adverse clinical outcomes, including ventilation (adjusted OR, 2.23; 95% CI, 1.56-3.18) and severe BPD (adjusted OR, 2.05; 95% CI, 1.15-3.64).

**Conclusions and Relevance:**

In this post hoc cohort study of premature neonates, fluid correction increased the number of premature neonates with a diagnosis of AKI and was associated with increased odds of adverse clinical outcomes, including ventilation and BPD. Failing to correct serum creatinine for fluid balance underestimates the prevalence and impact of AKI in premature neonates. Future studies should consider correcting AKI for fluid balance.

**Trial Registration:**

ClinicalTrials.gov Identifier: NCT01378273

## Key Points

**Question:**

Does correcting serum creatinine for fluid balance identify previously missed episodes of acute kidney injury (AKI) in premature neonates?

**Findings:**

In this post hoc cohort analysis of a placebo-controlled, randomized clinical trial of 923 premature neonates, fluid correction increased the number of premature neonates with a diagnosis of AKI. Correction for fluid balance was associated with increased odds of adverse clinical outcomes, including ventilation and severe lung disease.

**Meaning:**

These findings suggest that failing to correct serum creatinine for fluid balance underestimates the prevalence and severity of AKI in premature neonates, and future studies should consider correcting serum creatinine for fluid status to improve identification of AKI in premature neonates.

## Teaser

This secondary analysis of a placebo-controlled, randomized clinical trial investigates the diagnosis of acute kidney injury when correcting serum creatinine for fluid balance and its association with short-term and long-term clinical outcomes in extremely low gestational age neonates.

## Introduction

Acute kidney injury (AKI) commonly occurs in extremely low gestational age neonates (ELGANS)^1^ and is associated with worse in-hospital outcomes and an increased risk of subsequent chronic kidney disease.^2,3,4^ Fluid overload, defined as a pathologic positive fluid balance, is also common in ELGANS and is associated with an increased risk of mechanical ventilation and development of bronchopulmonary dysplasia (BPD).^5,6^ Disordered fluid balance can complicate the diagnosis of AKI when using classifications based on variations in serum creatinine levels. Creatinine is distributed between the intracellular and extracellular compartments, and the measurement of its concentration is inversely related to fluid balance.^7,8^ Fluid balance can delay the diagnosis of AKI by diluting (ie, creating a positive fluid balance) or concentrating (ie, creating a negative fluid balance) the measured serum creatinine concentration, impacting the severity of diagnosed AKI, and complicating the association of AKI with outcomes.^9,10^

In critically ill children and adults, correcting serum creatinine for fluid balance increases the precision and accuracy of AKI diagnosis^11,12^ and clarifies the association between AKI and outcomes (eg, mortality, length of hospital stay, and mechanical ventilation).^10,13^ Evaluating fluid balance in neonates is particularly complicated because they are born with high total body water and quickly experience an expected physiologic diuresis.^7,8,14^ Correcting serum creatinine for fluid balance allows assessment of true changes in kidney function independently of fluctuations in fluid balance. Such correction may allow for earlier AKI diagnosis and faster implementation of medical therapy (eg, diuretics or changes in intravenous fluid administration) or kidney support therapy. Conversely, correcting serum creatinine in patients with appropriate diuresis may decrease the rate of AKI diagnosis, decreasing unnecessary interventions. To our knowledge, the epidemiology and impact of correcting for fluid balance on the diagnosis of AKI in ELGANS has not been evaluated in a prospective or multicenter study.

The Preterm Erythropoietin Neuroprotection (PENUT) study captured robust data on kidney function and fluid status, as well as short-term and long-term outcomes in ELGANS.^1,15^ In this secondary analysis of the PENUT study, we sought to (1) describe fluid-corrected serum creatinine curves in ELGANS during the first 2 postnatal weeks, (2) describe the prevalence of AKI before and after fluid correction, and (3) evaluate the associations with short-term and long-term outcomes of AKI before and after fluid correction. Our primary hypothesis was that the correction of serum creatinine for fluid balance would identify previously undiagnosed AKI episodes. Furthermore, we hypothesized that fluid-corrected AKI would clarify the association of AKI with clinical outcomes.

## Methods

### Study Population

Performed in December 2022, this study was a post hoc analysis of the PENUT Trial (NCT01378273), a phase 3, placebo-controlled, randomized clinical trial of erythropoietin in ELGANS in 19 academic centers and 30 NICUs in the US.^15^ The University of Washington institutional review board served as the central institutional review board, and each center received approval from their institutional review board. Written informed consent was obtained from the parent or legal guardian. The inclusion criteria were (1) infants born between gestational ages of 24 weeks and 0 of 7 days and 27 weeks and 6 of 7 days, (2) enrolled within less than 24 hours after birth, and (3) arterial or venous access. Exclusion criteria included (1) major life-threatening anomalies, (2) hematologic crises, (3) hematocrit greater than 65%, (4) hydrops fetalis, and (5) congenital infection. Participant race and ethnicity were determined by maternal self-report as part of the original PENUT study and included Black, White, other race (American Indian or Alaska Native, Asian, and Native Hawaiian or Other Pacific Islander), unknown race, Hispanic or Latino ethnicity, and non-Hispanic or Latino ethnicity. These characteristics were included as part of the parent randomized clinical trial to allow investigators to balance study groups with regard to multiple factors, including race and ethnicity.^15^ See the PENUT study protocol in Supplement 1 for more information. This study follows the Strengthening the Reporting of Observational Studies in Epidemiology (STROBE) reporting guideline for observational studies.^16^

### Fluid Balance and Fluid-Corrected Serum Creatinine Definitions

The PENUT trial included daily weights for the first 14 postnatal days. Fluid balance was based on recent consensus guidelines^17,18^ and calculated as change in weight from birth weight using the following equation:
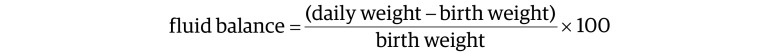
We calculated fluid-corrected serum creatinine, using an estimated total body water (milliliters) of 80% of daily weight (grams).^7,8^ If no daily weight was available on the day of serum creatinine assessment, the daily weight was imputed from surrounding days weights; weights were available for 97% of potential patient days. Fluid-corrected serum creatinine was calculated for each clinically available serum creatinine with the following equation:
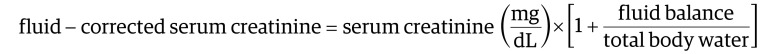
Each serum creatinine in the study was fluid adjusted, including the trough serum creatinine used to define the baseline and peak serum creatinine used for AKI diagnosis.

### AKI and Covariate Definition

AKI was defined using the serum creatinine criteria neonatal modified Kidney Disease: Improving Global Outcomes (KDIGO) definition.^19^ For this analysis, we assessed AKI during the first 14 postnatal days because this was when daily weights were available. Urine output criteria were not used due to incomplete reporting.^20^ Stage 1 AKI was defined as a 1.5- to 1.9-fold increase in serum creatinine or an increase of 0.3 mg/dL within 48 hours; stage 2 as a 2.0- to 2.9-fold increase; and stage 3 as a more than 3-fold increase in baseline serum creatinine or serum creatinine greater than 2.5 mg/dL. As with previous PENUT publications,^1^ AKI was determined using the lowest prior creatinine measurement as the baseline, and creatinine had to surpass a threshold of 0.5 mg/dL or more for diagnosis. Assessment of AKI began on postnatal day 3.^1^ Severe AKI was defined as KDIGO stage 2 or 3.

### AKI Class Switching

We defined class switching as neonates who moved from 1 stage of AKI to another after correcting for fluid balance. For example, a neonate with a positive fluid balance with no AKI by uncorrected serum creatinine may have had stage 2 AKI after correcting serum creatinine for fluid balance (ie, unveiled AKI). Conversely, a neonate with substantial diuresis and stage 1 AKI based on uncorrected serum creatinine may have been recategorized to no AKI after correction for a negative fluid balance (ie, overdiagnosed AKI) (eTable 1 in Supplement 2).

### Outcomes

The primary outcome was the need for invasive mechanical ventilation (ie, high-frequency or conventional ventilation) on postnatal day 14. Our secondary short-term outcomes included (1) BPD, defined by Neonatal Research Network definitions and Jensen criteria as neonates receiving respiratory support at 28 days of age,^21^ (2) grade 3 BPD defined as neonates receiving invasive mechanical ventilation at 36 weeks postmenstrual age,^21^ (3) hospital length of stay, and (4) death. Long-term kidney-related outcomes were evaluated at 24 months corrected gestational age (+2 months), and included decreased estimated glomerular filtration rate, albuminuria, and elevated blood pressure.^22^ Estimated glomerular filtration rate was defined by the serum creatinine and cystatin CKiD (chronic kidney disease in children) equation.^23^ Albuminuria was defined as an albumin-to-creatinine ratio more than 30 mg albumin per gram of creatinine. Elevated blood pressure was defined as values exceeding the 90th percentiles for age-related and sex-related norms.^24^

### Statistical Analysis

Categorical variables were analyzed by proportional differences with the χ^2^ test or Fisher exact test. The *t* test and Wilcoxon rank sums test were used to compare continuous and ordinal variables, respectively. Odds ratios (ORs) and associated 95% CIs for the association of exposure with outcomes of interest were estimated using unconditional logistic regression models. Multivariable logistic regression models, which used a backward selection process and were based on variables with *P* < .05 in bivariate analysis, were used to account for potential confounding variables; findings were reported as adjusted ORs (aORs). A repeated measures ANOVA with a 3-way interaction of fluid adjusted status, day of life and day of life category (ie, 1-3, 4-7, 8+) was used to compare fluid-corrected and uncorrected curves. In all analyses, a 2-sided *P *< .05 was considered statistically significant. Data visualization including Sankey diagrams were constructed using Flourish Studios.^25^ Analysis was performed using SAS statistical software version 9.4 (SAS Institute) in December 2022.

## Results

### Patient Characteristics

Of the 941 neonates who were enrolled in the PENUT trial, 923 (479 boys [51.9%]; median [IQR] birth weight 801 [668-940] g) were included in this analysis (eFigure in Supplement 2). Patient demographics are described in Table 1. Delivery room resuscitation included intubation (748 neonates [81.0%]) and surfactant administration (490 neonates [53.1%]). Commonly occurring neonatal complications included patent ductus arteriosus (384 neonates [41.6%]) and intraventricular hemorrhage (122 neonates [13.2%]) (Table 1).

**Table 1.** Comparison of Maternal and Neonatal Characteristics by Fluid-Corrected AKI Status

Characteristic	Participants, No. (%) (N = 923)				*P* value^a^
	True AKI (n = 202)	Unveiled AKI (n = 111)	Overdiagnosed AKI (n = 13)	No AKI (n = 597)	
Erythropoietin	104 (51.5)	51 (45.9)	6 (46.2)	293 (49.1)	.81
Sex					
Male	117 (57.9)	53 (47.7)	7 (53.8)	302 (50.6)	.25
Female	85 (42.1)	58 (52.3)	6 (46.2)	295 (49.4)	
Gestational age, wk					
24	76 (37.6)	32 (28.8)	8 (61.5)	111 (18.6)	<.001
25	59 (29.2)	40 (36.0)	2 (15.4)	141 (23.6)	
26	32 (15.8)	25 (22.5)	2 (15.4)	161 (27.0)	
27	35 (17.3)	14 (12.6)	1 (7.7)	184 (30.8)	
Birth weight, median (IQR), g	745 (650-860)	750 (650-860)	750 (660-780)	830 (685-960)	<.001
Small size for gestational age	32 (15.9)	16 (14.4)	1 (7.7)	92 (15.5)	.87
Apgar 1 min score, median (IQR)	4 (2-6)	3 (2-6)	2 (1-6)	4 (2-6)	.32
Apgar 5 min score, median (IQR)	6 (4-8)	7 (5-7)	4 (2-8)	7 (5-8)	.03
Delivery room resuscitation					
Any	199 (98.5)	109 (98.2)	13 (100.0)	575 (96.5)	.36
Intubation	97 (87.4)	177 (87.6)	13 (100.0)	461 (77.2)	<.001
Surfactant	125 (61.9)	69 (53.2)	9 (69.2)	287 (48.1)	.004
Chest compressions	17 (8.4)	7 (6.3)	3 (23.1)	45 (7.5)	.19
Resuscitation drugs	8 (4.0)	1 (0.9)	4 (30.8)	19 (3.2)	<.001
Maternal characteristics					
Multiple gestations	50 (24.8)	31 (28.0)	6 (46.2)	156 (26.1)	.38
Diabetes	9 (4.5)	2 (1.8)	0	37 (6.2)	.19
Hypertension	15 (7.4)	8 (7.2)	0	47 (7.9)	.76
Preeclampsia	24 (11.9)	17 (15.3)	1 (7.7)	98 (16.4)	.39
Maternal race					
Black	54 (26.7)	33 (29.7)	2 (15.4)	150 (25.1)	.59
White	135 (66.8)	68 (61.3)	9 (69.2)	392 (65.7)	
Other^b^	6 (3.0)	7 (6.3)	2 (15.4)	37 (6.2)	
Unknown	7 (3.5)	3 (2.7)	0	18 (3.0)	
Maternal ethnicity					
Hispanic or Latino	62 (30.7)	23 (20.7)	5 (38.5)	107 (17.9)	<.001
Not Hispanic or Latino	138 (68.3)	88 (79.3)	7 (53.8)	482 (80.7)	
Unknown	2 (1.0)	0	1 (7.7)	8 (1.3)	
Neonatal course					
Patent ductus arteriosus (treated)	104 (51.7)	73 (65.8)	7 (53.8)	200 (33.6)	<.001
Severe intraventricular hemorrhage	32(15.8)	25 (22.5)	1 (7.7)	64 (10.7)	.004
Outcomes					
Mechanical ventilation at postnatal d 14					<.001
High-flow cannula	19 (9.7)	12 (11.1)	0	98 (17.1)	
Noninvasive	39 (19.9)	15 (13.9)	5 (41.7)	222 (38.7)	
Invasive	138 (70.4)	81 (75.0)	7 (58.3)	254 (44.3)	
Bronchopulmonary dysplasia	83 (41.1)	42 (37.8)	5 (38.5)	211 (35.3)	.53
Length of hospital stay, median (IQR), d	96 (72-116)	102 (84-124)	97 (76-114)	90 (71-110)	.003
Mortality	30 (14.9)	9 (8.1)	3 (23.1)	58 (9.7)	.07

Abbreviation: AKI, acute kidney injury.

^a^ Based on χ^2^ test for categorical variables and Kruskal-Wallis test for continuous variables.

^b^ Other maternal race category includes American Indian or Alaska Native, Asian, Native Hawaiian, or Other Pacific Islander.

### Serum Creatinine Curves During the First 2 Postnatal Weeks

A total of 8757 serum creatinine values were obtained during the first 2 postnatal weeks with a median (IQR) of 11 (7 to 13) values per patient. The median (IQR) peak negative fluid balance was −10.3% (−14.6% to −5.6%) on median (IQR) postnatal day 3 (2 to 5). Median (IQR) peak positive fluid balance in the first 2 postnatal weeks in this population was 11.1% (4.3% to 19.6%) and occurred on median (IQR) postnatal day 12 (8 to 14). A total of 93 (10.1%) neonates never dropped below their birth weight.

**Figure 1.**
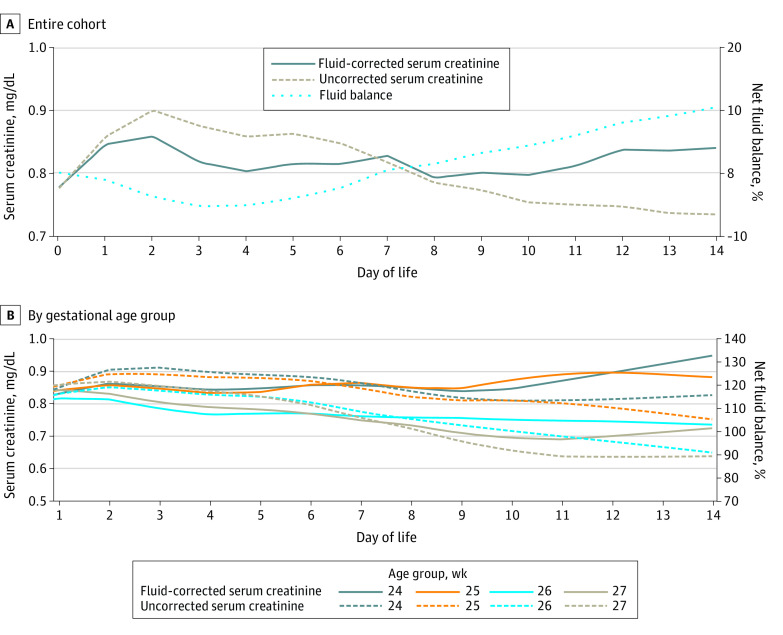
Uncorrected and Fluid-Corrected Serum Creatinine Curves The figure shows uncorrected serum creatinine and fluid-corrected serum creatinine curves for the entire cohort (A) and by gestational age group (B).

Uncorrected and fluid-corrected serum creatinine curves are shown for the entire cohort and by gestational age group (Figure 1). Correcting for fluid balance significantly altered the serum creatinine curve. From postnatal day 1 to 3, the fluid-corrected serum creatinine curve decreased (χ^2^_1_ = 9.79; *P* = .002) whereas the serum creatinine curve increased (χ^2^_1_ = 4.23; *P* = .04). From postnatal day 4 to 7, the fluid-corrected serum creatinine curve flattened whereas the uncorrected serum creatinine curve decreased (χ^2^_1_ = 21.45; *P* < .001). After postnatal day 7, fluid-corrected serum creatinine curve increased (χ^2^_1_ = 9.41; *P* = .002) whereas the uncorrected serum creatinine curve continued on a downward slope (χ^2^_1_ = 15.38; *P* < .001).

### AKI Diagnosis and Class Switching

**Figure 2.**
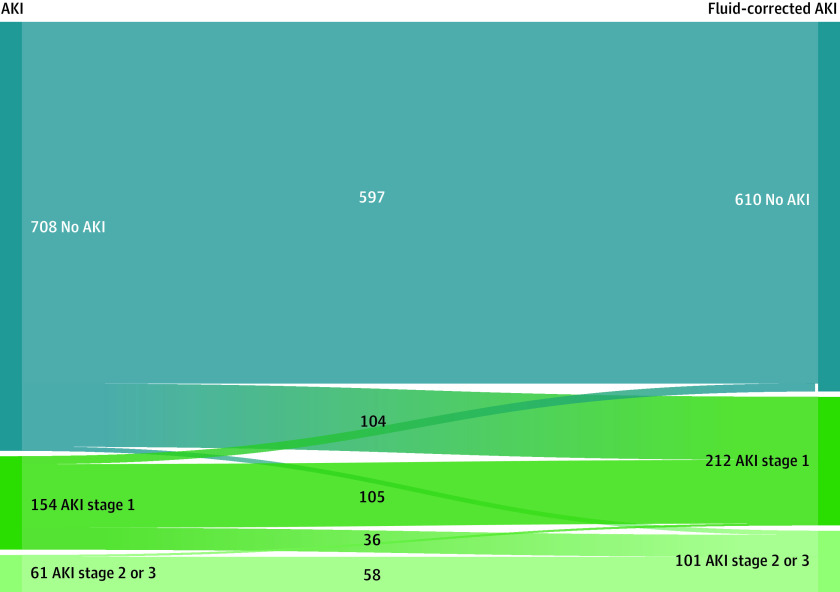
Acute Kidney Injury (AKI) Status and Class Switching After Fluid Correction The figure displays a Sankey diagram visualizing the change in AKI status and AKI class switching after correction for fluid status.

AKI diagnosis using uncorrected serum creatinine occurred for 215 neonates (23.3%), which included 154 neonates at stage 1, 45 at stage 2, and 16 at stage 3. No neonates in this study were treated with dialysis. Correcting for fluid balance increased the incidence of AKI to 33.9% (313 neonates), of which 101 neonates (32.3%) had severe AKI (Figure 2; eTable 1 in Supplement 2). There was 87% concordance in AKI diagnosis and 95% concordance in severe AKI diagnosis between the uncorrected AKI diagnosis and fluid-corrected AKI diagnosis.

After correcting serum creatinine for fluid balance, 124 neonates switched AKI classes. A total of 111 neonates were newly classified as having unveiled AKI, including 104 who went from no AKI to stage 1 AKI, and 7 who went from no AKI to stage 2 or 3 AKI. In 36 neonates, AKI severity increased from stage 1 to stage 2 or 3 AKI. After correcting for fluid balance, 16 neonates had their AKI stage decrease (13 patients went from stage 1 AKI to no AKI and 3 went from stage 2 or 3 AKI to stage 1 AKI). A summary of the shifts in the AKI staging before and after fluid correction is shown in Figure 2.

### Short-Term and Long-Term Outcomes

A total of 408 neonates (52.0%) were mechanically ventilated at postnatal day 14 (Table 1). The secondary outcomes of interest included 341 neonates with BPD (36.9%), 56 (6.1%) with grade 3 BPD, and 100 who died (10.8%). Long-term kidney-related outcomes of interest included 54 neonates (16.2%) with a decreased estimated glomerular filtration rate of less than 90 mL/min/1.73 m^2^, 155 neonates (35.6%) with albuminuria, and 160 neonates with elevated blood pressure (32.6%).

#### Association of Fluid-Corrected AKI With Outcomes

The bivariable associations with our primary outcome of mechanical ventilation on postnatal day 14 is described in eTable 2 in Supplement 2. In multivariable analysis, correction for fluid during AKI diagnosis was associated with increased odds of adverse clinical outcomes, including ventilation (aOR, 2.23; 95% CI, 1.56-3.18) and grade 3 BPD (aOR, 2.05; 95% CI, 1.15-3.64) (Table 2). These findings were similar in neonates with fluid-corrected severe AKI, including those who underwent mechanical ventilation (aOR, 2.67; 95% CI, 1.48-4.82) (eTable 3 in Supplement 2 and Table 3).

**Table 2.** Association of AKI With Short-Term and Long-Term Outcomes

Outcome	AKI				Fluid-corrected AKI		
	Crude OR (95% CI)		aOR (95% CI)^a^		Crude OR (95% CI)	aOR (95% CI)^a^	
Short-term outcome							
Mechanical ventilation at 14 d of life	2.90 (2.07-4.07)		1.75 (1.16-2.62)		3.25 (2.41-4.38)	2.23 (1.56-3.18)	
Prolonged hospital stay^b^	1.44 (1.05-1.96)		1.01 (0.72-1.42)		1.56 (1.19-2.06)	1.19 (0.89-1.61)	
Mortality	1.69 (1.07-2.67)		1.01 (0.59-1.71)		1.32 (0.86-2.04)	0.81 (0.49-1.34)	
Bronchopulmonary dysplasia^c^	1.27 (0.92-1.75)		0.90 (0.63-1.28)		1.22 (0.92-1.61)	0.92 (0.67-1.25)	
Grade 3 bronchopulmonary dysplasia^d^	2.38 (1.31-4.32)		2.03 (1.08-3.80)		2.24 (1.30-3.88)	2.05 (1.15-3.64)	
Long-term							
Estimated glomerular filtration rate <90 mL/min/1.73 m^2^	1.31 (0.65-2.62)		1.33 (0.62-2.84)		1.39 (0.76-2.52)	1.41 (0.73-2.73)	
Albumin to creatine ratio >30 mg albumin/g creatinine	1.09 (0.67-1.75)		1.12 (0.67-1.88)		0.98 (0.63-1.50)	1.05 (0.66-1.67)	
Blood pressure >90th percentile	1.28 (0.81-2.02)	1.34 (0.82-2.20)		1.06 (0.72-1.56)			1.10 (0.73-1.67)

Abbreviations: AKI, acute kidney injury; aOR, adjusted odds ratio; OR, odds ratio.

^a^ Adjusted for gestational age, sex, small for gestational age status, 5-minute Apgar score, intubation, epinephrine, chest compressions, necrotizing enteritis, sepsis, and intraventricular hemorrhage.

^b^ Refers to hospital length of stay longer than median of 93 days.

^c^ Bronchopulmonary dysplasia is defined as oxygen requirement on day 28 of age.

^d^ Grade 3 bronchopulmonary dysplasia is defined as invasive mechanical ventilation at 36 weeks corrected gestational age.

**Table 3.** Association of Severe AKI With Short-Term and Long-Term Outcomes in Extremely Low Gestational Age Neonates

Outcome	Severe AKI		Severe fluid-corrected AKI	
	Crude OR (95% CI)	aOR (95% CI)^a^	Crude OR (95% CI)	aOR (95% CI)^a^
Short-term outcome				
Mechanical ventilation at 14 d of life	3.27 (1.77-6.03)	2.31 (1.14-4.68)	4.04 (2.40-6.80)	2.67 (1.48-4.82)
Prolonged hospital stay^b^	0.96 (0.56-1.61)	0.70 (0.40-1.23)	1.15 (0.76-1.74)	0.83 (0.53-1.29)
Mortality	1.74 (0.85-3.55)	1.18 (0.51-2.71)	1.67 (0.93-2.98)	1.20 (0.85-2.17)
Bronchopulmonary dysplasia^c^	1.43 (0.85-2.42)	1.13 (0.64-1.99)	1.64 (1.08-2.48)	1.28 (0.81-2.00)
Grade 3 bronchopulmonary dysplasia^d^	1.47 (0.56-3.83)	1.19 (0.44-3.23)	1.61 (0.76-3.39)	1.37 (0.63-2.96)
Long-term outcome				
Estimated glomerular filtration rate <90 mL/min/1.73 m^2^	1.30 (0.46-3.62)	1.64 (0.54-4.98)	0.79 (0.29-2.12)	0.82 (0.28-2.36)
Albumin to creatine ratio >30 mg albumin/g creatinine	0.93 (0.39-2.25)	0.99 (0.39-2.51)	0.67 (0.34-1.35)	0.67 (0.33-1.38)
Blood pressure >90th percentile	1.67 (0.75-3.73)	1.92 (0.81-4.56)	1.28 (0.71-2.31)	1.37 (0.73-2.58)

Abbreviations: AKI, acute kidney injury; aOR, adjusted odds ratio; OR, odds ratio.

^a^ Adjusted for gestational, sex, small for gestational age status, 5-minute Apgar score, intubation, epinephrine, chest compressions, necrotizing enteritis, sepsis, and intraventricular hemorrhage.

^b^ Refers to hospital length of stay longer than median of 93 days.

^c^ Bronchopulmonary dysplasia is defined as oxygen requirement on day 28 of age.

^d^ Grade 3 bronchopulmonary dysplasia is defined as invasive mechanical ventilation at 36 weeks corrected gestational age.

Although the association of AKI with the short-term outcomes of BPD and prolonged hospital stay were not statistically significant after multivariable adjustment, exposure was associated with increased odds of adverse outcomes after adjustment for fluid balance (Table 2). The odds of mortality were lessened with fluid correction. Similar patterns were seen in neonates with severe AKI (eTable 3 in Supplement 2). There was no association of either AKI or fluid-corrected AKI with long-term kidney outcomes (Table 2 and Table 3).

#### Association of AKI Class Switching With Outcomes

With regard to most demographic and clinical characteristics, neonates with unveiled AKI were similar to those with AKI diagnosed using uncorrected serum creatinine (Table 1). Neonates with unveiled AKI had higher rates of patent ductus arteriosus requiring treatment (73 neonates [65.8%] vs 104 neonates [51.7%]) and severe intraventricular hemorrhage (25 neonates [22.5%] vs 32 neonates [15.8%]) compared with those with AKI diagnosed using uncorrected serum creatinine. We saw similar characteristics in neonates with severe AKI and fluid-corrected severe AKI (eTable 3 in Supplement 2).

Compared with those without AKI, neonates with unveiled AKI were more likely to require invasive mechanical ventilation on postnatal day 14 (81 neonates [75.0%] vs 254 neonates [44.3%]) and have longer hospital stays (median [IQR] stay, 102 [84-124] days vs 90 [71-110] days). There was no difference in mortality between these groups. Neonates with unveiled severe AKI were more likely to require ventilation on postnatal day 14 (33 neonates [82.5%] vs 400 neonates [50.7%]) compared with those without AKI, but we saw no difference in hospital length of stay or mortality (Table 3).

## Discussion

In this secondary analysis of a large, prospective, multicenter, randomized clinical trial of ELGANS, we evaluated the impact of fluid correction of serum creatinine on AKI diagnosis and its association with short-term and long-term outcomes. We found that fluid correction identified many neonates with previously undiagnosed AKI, and that correcting for fluid balance changed AKI staging in many patients. Fluid-corrected AKI correlated better with clinically relevant outcomes and was associated with increased odds of short-term clinical outcomes, including mechanical ventilation and grade 3 BPD. Furthermore, correcting serum creatinine for fluid balance created a new serum creatinine curve for neonates over the first 2 weeks of life, with a slow improvement in kidney function over time, which challenges the often cited peak and then fall serum creatinine trajectory over the first several postnatal days.^26^

Over the past several decades, the study of AKI has been most impacted by consensus definitions for diagnosis and staging.^17,19^ In recent years, opportunities for improving AKI diagnosis have been identified, including incorporating fluid balance into AKI diagnosis. In critically ill adults, fluid-corrected AKI better identifies AKI associated with increased mortality.^13^ In critically ill children, correction of AKI status for fluid balance increases the odds of experiencing adverse outcomes.^10^ To date there have only been 2 small single-center studies evaluating fluid correcting AKI in neonatal populations. In 1 small retrospective study^11^ of neonates undergoing cardiac surgery, it was found that correcting for fluid balance increased AKI diagnosis. In a second small single-center retrospective study^14^ of ELGANS, it was found that correcting for fluid balance decreased the incidence of AKI. We note that our findings are in contrast to this study and hypothesize this is due to our novel approach to the correction of both peak and trough serum creatinine. The correction of the trough creatinine, which serves as the baseline to diagnose and stage AKI, adds precision and accuracy to the novel method previously described.^14^ Our study underlines the importance of careful attention to fluid balance, including frequent measurement of weights in premature neonates because episodes of AKI associated with poor short-term outcomes may be diagnosed only after correcting for fluid balance.^17^

Our findings should be interpreted in the context of the unique physiology of the premature neonate. Premature neonates differ from older children in that they are subject to the full spectrum of fluid balance during the first 2 postnatal weeks including positive fluid balance, physiologic diuresis, and diuretic-induced diuresis, which impacts the diagnosis of AKI in neonates, because changes in fluid balance complicate the AKI diagnosis when using serum creatinine diagnostic criteria.^8^ This phenomenon is unique to the neonatal population because correcting serum creatinine for fluid balance can encompass the full spectrum from dilution to concentration. The current study suggests that accounting for changes in fluid balance when diagnosing AKI in the first 2 postnatal weeks improves the precision and accuracy of the AKI diagnosis in premature neonates. Confirmation of these findings and further study is warranted, particularly in specific neonatal populations where fluid balance may be particularly relevant.

### Strengths and Limitations

This multicenter prospective study has several strengths, including the robust high-quality data available in a large, prospective, multicenter study allowing for the detailed exploration of the impact of fluid balance on AKI diagnosis. Furthermore, the study has long-term kidney-specific outcomes for the cohort.

Despite these strengths, there are several limitations of this secondary analysis. Our findings were limited by clinically obtained serum creatinine, and weights and urine output were not collected. However, weights were available for 97% of potential patient days, and this analysis imputed missing values; therefore, misclassification is likely infrequent. Because the PENUT database only included daily weights from the first 2 postnatal weeks, our conclusions are limited to this period. Longer observation periods are necessary to extend our findings. We note that there are other variables, such as clinical conditions and management practices, that may result in changes in weight and are not captured in this analysis. Third, although defining AKI by neonatal modified KDIGO definitions is the current standard for this patient population, this may not be the optimal marker for detection of kidney dysfunction in premature neonates.^19,27^ Because the neonatal modified KDIGO definition relies on creatinine, factors such as maternal kidney function, gestational age, and infant growth also impact the interpretation of creatinine as a marker of kidney function. Additionally, we assumed a static percentage of weight as water (80%) across a range of gestation ages and during the first 2 postnatal weeks. Although this approach is consistent with previous studies^8^, it assumes that all changes in weight during the first 2 weeks are due to changes in water and does not take into account the physiologic natriuresis that occurs in the first several days after birth or any postnatal growth. Although using birth weight as an anchor weight for the first 14 postnatal days is currently the standard approach, this method may overestimate the fluid balance, especially during the first postnatal week. Further studies are needed to better assess the changes in body composition and body water in premature neonates.^7^

## Conclusion

In this secondary analysis of the multicenter PENUT trial, we describe fluid-corrected AKI and evaluate associations with short-term and long-term outcomes in premature neonates with fluid-corrected AKI. Taken together, the current study suggests a paradigm shift in how we describe kidney function in premature neonates in the first 2 postnatal weeks. The current study suggests that fluid balance should be considered in future definitions to improve the diagnosis of AKI in premature neonates. Failing to correct serum creatinine for fluid balance underestimates the prevalence and severity of AKI in premature neonates. Future studies should consider correcting AKI for fluid balance in the first 2 weeks to improve identification of neonates at high risk for poor outcomes.

## Notes

Supplement 1.
PENUT Trial Protocol and Statistical Analysis

Supplement 2. **eTable 1.** Definition and Distribution of AKI Status by Serum Creatinine and Fluid-Corrected Serum Creatinine AKI **eFigure.** CONSORT Diagram **eTable 2.** Comparison of Maternal And Neonatal Characteristics by Primary Outcome of Mechanical Ventilation at Post-Natal Day 14 **eTable 3.** Comparison of Maternal and Neonatal Characteristics by Severe Fluid-Corrected AKI Status

Supplement 3.
Data Sharing Statement
