# Supplementary material for: Acute Kidney Injury Defined by Fluid-Corrected Creatinine in Premature Neonates: A Secondary Analysis of the PENUT Randomized Clinical Trial
Source: JAMA Netw Open. 2023 Aug 10;6(8):e2328182. doi: 10.1001/jamanetworkopen.2023.28182 (PMC10415963; doi:10.1001/jamanetworkopen.2023.28182)
Supplement: Supplement 1. — PENUT Trial Protocol and Statistical Analysis [file jamanetwopen-e2328182-s001.pdf]

Protocol

This trial protocol has been provided by the authors to give readers additional information about their work.

Protocol for: Juul SE, Comstock BA, Wadhawan R, et al. A randomized trial of erythropoietin for neuroprotection in preterm infants. N Engl J Med 2020;382:233-43. DOI: 10.1056/NEJMoa1907423

Supplement to the PENUT Trial Manuscript

This supplement contains the following items:

1. Original protocol (V2.1, 4/5/2013)

    -V2.1 was the original version of the protocol prior to initiation of the trial

2. Final protocol (V5, 8/21/18)
3. Summary of changes

4. Original statistical analysis plan
5. Final statistical analysis plan
6. Summary of changes

PENUT Trial
Preterm Epo Neuroprotection

A Multi-Center, Randomized Placebo-Controlled Phase III 940-subject Trial of Erythropoietin for the Neuroprotection of Extremely Low Gestational Age Neonates (ELGANs)

Study Chair:
Sandra E. Juul, M.D., Ph.D.
Associate Division Head for Scholarship and Research
Professor of Pediatrics
University of Washington

Supported by:
The National Institute of Neurological Disorders and Stroke (NINDS)

PAR-11-173

Sponsor of IND (IDE):
IND Holder: Sandra Juul IND # 12656

Version 2.1
04/05/2013

TABLE OF CONTENTS
Page

SYNOPSIS ………..……………………………………………………………………….. 4

1. STUDY OBJECTIVES ………………………………………………………………... 7

1.1 Primary Objective …………………………………………………….………… 7
1.2 Secondary Objectives ..……………………………………………….………… 7

2. BACKGROUND ………………………………………………………………..….. 8
2.1 Rationale ………..………………………………………………………….. 8
2.2 Supporting Data …… ……………………………..………………………… 8

3. STUDY DESIGN ……………………………………………………...……………. 11

4. SELECTION AND ENROLLMENT OF SUBJECTS …………………..………….. 15

4.1 Inclusion Criteria ………………………………………………………..……….. 15
4.2 Exclusion Criteria ……………………………………………………….…..…… 16
4.3 Study Enrollment Procedures ………………………………………………….. 16

5. STUDY INTERVENTIONS ……………………………………………….……….... 21

5.1 Interventions, Administration, and Duration ...………………………….…… 21
5.2 Handling of Study Interventions ……………………………………….……… 28
5.3 Concomitant Interventions ……………………………………………….…. 32
5.4 Adherence Assessment ……………………………………………………… 32

6. CLINICAL AND LABORATORY EVALUATIONS ………………………………… 33

6.1 Schedule of Evaluations ……………………………………………….………… 35
6.2 Timing of Evaluations ……………………………………………….…………… 36
6.3 Special Instructions and Definitions of Evaluations ……………………….…… 37

7. MANAGEMENT OF ADVERSE EXPERIENCES ……...…………………...……. 41

8. CRITERIA FOR INTERVENTION DISCONTINUATION ……………..…………. 42

9. STATISTICAL CONSIDERATIONS ……………………………………...…….. 42

9.1 General Design Issues ……………………………………………….………... 42
9.2 Outcomes …………………………………………………………...………… 42
9.3 Sample size and recruitment …………………………………………..……….. 48
9.4 Data Monitoring ……………………………………………………………..……... 48
9.5 Data Analysis

10. DATA COLLECTION, SITE MONITORING, AND ADVERSE EXPERIENCE REPORTING ………………………………………………………………………...
10.1 Records to be Kept …………………………………………………………..…… 49
10.2 Role of Data Management ……………………………………………………..…… 49

10.3 Quality Assurance ……………………………………………………………………..…. 50
10.4 Adverse Experience Reporting ……………………………………………………..… 50

11. HUMAN SUBJECTS ……………………………………………………………...……. 51

11.1 Institutional Review Board (IRB) Review and Informed Consent ………………... 51
11.2 Subject Confidentiality ……………………………………………………………..……. 51
11.3 Study Modification/Discontinuation …………………………………………….…..…… 51

12. PUBLICATION OF RESEARCH FINDINGS ……………………………...……….. 51

13. REFERENCES……………………………………………………………...…….…. 52

APPENDICES

Appendix 1: Protocol

Appendix 2. Timeline

Appendix 3: PENUT Trial study sites

Appendix 4: Standardized Neurologic Exam

Appendix 5: W I D E A - F S

Appendix 6: CRF forms

Appendix 7: Clinical guidelines for PENUT Trial

Appendix 8: Pulse sequences

Appendix 9: MCHAT

Appendix 10: Consent form

SYNOPSIS

Study Title: **P**reterm **E**po **N**e**u**ropro**t**ection Trial (PENUT Trial)

Objectives

Overall Primary Objective:
Our primary goal is to test the hypothesis that early high dose recombinant human erythropoietin (Epo) treatment of preterm infants born between 24-0/7 and 27-6/7 weeks of gestation will improve long-term neurodevelopmental outcomes measured at 24-26 months post menstrual age (PMA). Secondary outcomes will include evaluation of: 1) short and long-term safety of high dose Epo, 2) effect of Epo on circulating biomarkers of brain injury and inflammation, and 3) Epo effects on brain structure evaluated at 36 weeks PMA. We will achieve these goals by conducting a multicenter blinded, randomized, placebo-controlled phase III study. Towards the end of this study, we will apply for additional funding to follow the enrolled cohort at 5 and 9 years of age to evaluate whether benefits are sustained over time. Dr. Juul holds IND 12656 Epo Neuroprotection. The trial is registered with clinicaltrials.gov, Identifier Number NCT01378273.

Primary Objectives

1. To determine whether Epo decreases the combined outcome of death or neurodevelopmental impairment (NDI) at 24-26 months PMA. Impairment is defined as the presence of any one of the following: CP, Bayley Scales of Infant and Toddler Development, 3rd Edition (Bayley-III) cognitive or motor scale < 70 (severe, 2 SD below mean) or 85 (moderate, 1 SD below mean).[2, 3] CP will be identified by standardized neurologic exam and categorized as no CP, diplegic CP, hemiplegic CP, or quadriplegic CP. Severity of CP will be determined using the Gross Motor Function Classification System (GMFCS) developed by Palisano.[4, 5]

Secondary Objectives

1. To determine whether there are risks to Epo administration in **E**xtremely **l**ow **g**estational **a**ge **n**eonates (ELGANs) by examining, in a blinded manner, Epo-related safety measures comparing infants receiving Epo with those given vehicle. As part of this aim, Epo pharmacokinetics will be determined.
2. To test whether Epo treatment decreases circulating inflammatory mediators and known biomarkers of brain injury over time.
3. To compare brain structure (as measured by MRI) in Epo treatment and control groups at 36 weeks PMA. MRI assessments will include documentation of intraventricular hemorrhage (IVH), white matter injury (WMI) and hydrocephalus (HC), volume of total and deep gray matter, white matter and cerebellum, brain gyrification, inter-hemispheric distance, and tract-based spatial statistics (TBSS based on diffusion tensor imaging).

As an **exploratory aim,** we will determine which of the above MRI measurements best predict neurodevelopment at 24-26 months PMA.

Design and Outcomes

Our primary goal is to determine whether administration of early high dose recombinant human erythropoietin (Epo) to premature infants born at ≤ 27-6/7 weeks of gestation will improve long-term neurodevelopmental outcomes measured at two years of age. Secondary outcomes will be to determine safety of this Epo dosing regimen, and to determine biochemical and structural biomarkers of Epo neuroprotective effects. These goals will be achieved by conducting a multicenter blinded, randomized, placebo-controlled phase III study.

Figure 1 depicts an overview of the proposed trial.

Figure 1. Study Overview

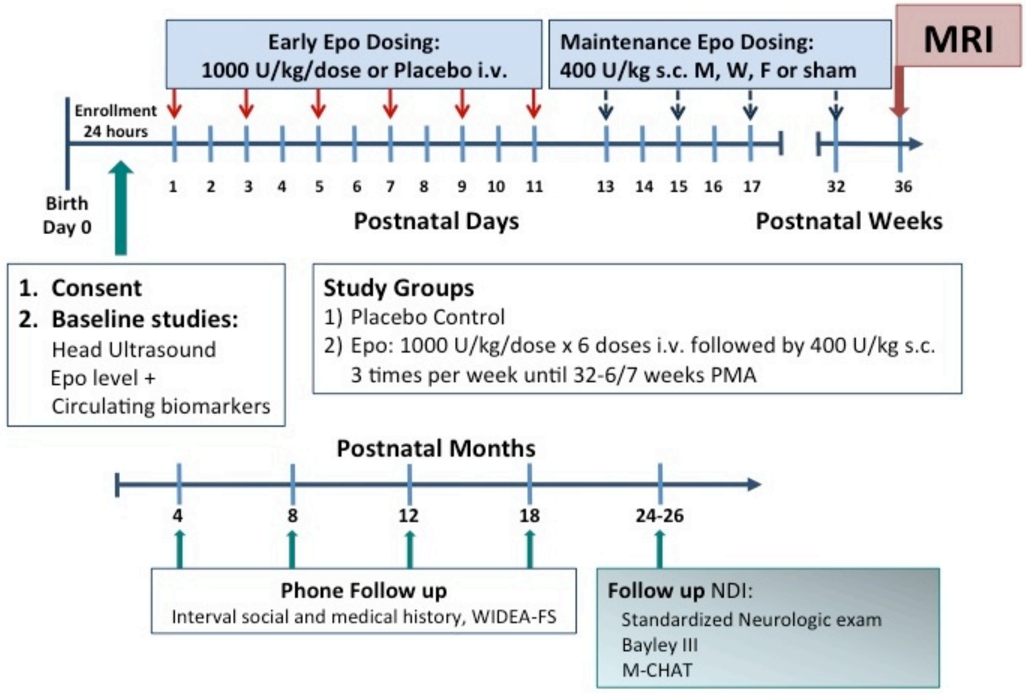

<u>Interventions and Duration</u>

Enrollment will occur prior to 24 hours of age. After enrollment but prior to the first dose of study drug, a cranial ultrasound will be obtained to document whether an intracranial hemorrhage has occurred prior to dosing. Baseline blood will be drawn to measure Epo concentration, inflammatory mediators and biomarkers of brain injury. Subjects will be randomized to Epo treatment or vehicle, based on randomization by the data coordinating center (DCC). Each subject will be on the study (intervention + time off intervention) until completion of the follow-up visit at 24-26 months.

Study Drug Dosing

Subjects will be randomized to Epo treatment or placebo for the first 6 doses of intravenous study drug, which will be given every 48 hours (q.o.d.). Subjects in the Epo group will receive 6 doses of 1000 U/kg during the first 2 weeks of life when physiologic vulnerability is highest. This will be followed by maintenance Epo dosing of 400 U/kg 3 X per week during the period of oligodendrocyte vulnerability (to 32-6/7 wks). Maintenance Epo (400 U/kg/dose) will be administered by s.c. injection on Monday, Wednesday and Friday, beginning on the day closest to completing the high dose series. To maintain blindness, control infants will have a saline-filled syringe dispensed from pharmacy that appears identical to the Epo-filled syringe dispensed to infants randomized to the Epo treatment group, but these infants <u>will not</u> undergo any injections. Treatments will be administered behind blinds. After injection or sham injection, all infants will have a small adhesive bandage placed over the “injection site”. The duration of treatment will depend on the subject's gestational age at birth, and will range from 9 doses of 400 U/kg for those born at 27-6/7 weeks to 21 doses for those born at 24-0/7 weeks.

<u>Group 1 (Epo):</u> 1000 U/kg/dose i.v. every 48 hours x 6 doses. Beginning at 13 days, and

continuing to 32-6/7 weeks, maintenance Epo (400 U/kg/dose three times per week) will be administered by s.c. injection.

Group 2: (Placebo): Saline placebo i.v. every 48 hours x 6 doses. Control infants will not receive any maintenance doses of study drug, and will not undergo any subcutaneous injections, but, similar to treated subjects, will have an adhesive bandage placed (so-called "sham injections"). To maintain blinding, treatments will be administered by the research nurse behind blinds.

Interventions that each subject will experience:
1. Head ultrasound prior to study drug dosing (all subjects)
2. Intravenous study drug treatment (Epo or placebo) for 6 doses, day 1-11 (all subjects)
3. Epo group: s.c. Epo injections three times a week until 32-6/7 weeks PMA; Control infants will receive sham injections (no injection) three times a week until 32-6/7 weeks PMA
4. Iron supplementation (all infants)
5. Timed blood draws totaling 2.0 mL during the study (see Figure 3)
6. Brain MRI at 36 weeks PMA (220 subjects only)
7. Follow up phone calls at 4, 8, 12, 18 months corrected age
8. Standardized neurologic exam, GMFCS evaluation, Bayley Scales of Infant and Toddler Development, 3rd Edition, and M-CHAT questionnaire at 24-26 months PMA.

Sample Size and Population
We will enroll at total of 940 subjects in order to maintain 752 (376 per arm) who survive and are followed at 24 to 26 months PMA. This takes into account patient mortality within and post hospitalization, withdrawal from the study protocol, and loss to follow up at 2 years.

Qualified infants less than 24 hours of age of both sexes and all races will be entered into the study if their parents or legal guardians consent.

Randomization will be stratified by site and by gestational age: 24-0/7 to 25-6/7 and 26-0/7 to 27-6/7. We will use block randomization within site using variable blocks of size 6-10 subjects. Using block randomization ensures that equal numbers of subjects are randomized to the intervention and control arm and that the two groups are balanced at period enrollment intervals. For multiple births (twins, triplets) all infants will be randomized the same treatment group (e.g. effective randomization of the mother). Subjects will also be stratified by English monolingual vs. all other language patterns, given that language spoken in the home may affect developmental testing.

Table 1. Frequently Used Abbreviations:

BPD	Bronchopulmonary dysplasia	MDI	Mental developmental index
CCC	Clinical coordinating center	NDI	Neurodevelopmental impairment
CP	Cerebral palsy	NEC	Necrotizing enterocolitis
DCC	Data coordinating center	NICU	Neonatal intensive care unit
DEHSI	Diffuse excessive high signal intensity	PDA	Patent ductus arteriosus
ELGANs	Extremely low gestational age neonates	PDI	Psychomotor developmental index
Epo	Erythropoietin	PMA	Postmenstrual age
EpoR	Epo receptor	PVL	Periventricular leukomalacia
GMFCS	Gross motor function classification	ROP	Retinopathy of prematurity

system			
HC	Hydrocephalus	TBSS	Tract based spatial statistics
IVH	Intraventricular hemorrhage	VON	Vermont Oxford Network
M-CHAT	Modified Checklist for Autism in Toddlers	WMI	White matter injury

1. STUDY OBJECTIVES

1.1. Primary Objective

Hypothesis: Epo treatment from 24 hours to 32-6/7 weeks post menstrual age (PMA) of **E**xtremely **L**ow **G**estational **A**ge **N**eonates (ELGANs) will safely decrease the combined outcome of death or neurologic impairment from 40% to 30% measured at two years of age.

Design: This is a prospective, randomized controlled study. We will enroll 940/2 = 470 subjects in each of two treatment groups: Epo-treated (from 24 hours of age to 32-6/7 weeks PMA) vs. *placebo control.*

Primary outcome variable: The primary outcome is the composite outcome of death or neurodevelopmental impairment at 24-26 months. Neurodevelopmental impairment (NDI) is defined as the presence of any one of the following: CP, Bayley III cognitive or motor scale < 70. There is a known inflation of scores from the Bayley II to III[2, 6, 7] and we will therefore also consider a threshold of <85 for secondary analysis. Subjects will be stratified by gestational age (24-0/7 to 25-6/7 and 26-0/7 to 27-6/7) and by study site. Table 2 shows how CP will be categorized based on features present on standardized neurologic exam.

Table 2. Motor deficit severity defined by CP and GMFCS function score.

Motor Deficit	**QP**	**Any CP** with GMFCS **3-5**	**HP or DP** with GMFCS **1-2**	**HP or DP** with GMFCS **0**	**No CP** but GMFCS **1-2**	**No CP** and GMFCS **0**
**Severe**	+	+				
**Moderate**			+		+	
**Mild**				+		
**None**						+

1.2 Secondary Objectives

Hypothesis 2. High dose Epo will be safe to administer from birth to 32-6/7 weeks of gestation in this population of ELGANs.

Specific aim 2. To determine whether there are excess adverse events in Epo treated ELGANs compared to control infants. Three time periods will be considered: 1. Safety during the treatment period; 2. Safety during the initial hospitalization; and 3. Long term outcomes. Timed Epo concentrations will be measured to monitor for drug accumulation and safety. A Population Pharmacokinetic Analysis will be done using these data.

Hypothesis 3. Therapeutic Epo administration during the period of oligodendrocyte vulnerability (24-32 weeks of gestation) promote normal brain development by increasing BDNF, decreasing circulating inflammatory mediators, and thereby decreasing biomarkers of brain injury.

Specific aim 3. To test whether Epo treatment changes sequential measures of BDNF, circulating inflammatory mediators and known biomarkers of brain injury in preterm infants.

Hypothesis 4. Epo treatment will improve brain development as assessed by MRI at 36 weeks PMA.

Hypothesis 5. A combination of structural brain measurements done by MRI at 36 weeks PMA

will sensitively and specifically predict neurodevelopment at 24-26 months corrected age.

Specific aim 4. To compare brain structure (as measured by MRI) in Epo treatment and control groups at 36 weeks PMA. MRI assessments will include documentation of intraventricular hemorrhage (IVH), white matter injury (WMI) and hydrocephalus (HC), volume of total and deep gray matter, white matter and cerebellum, brain gyrification, inter-hemispheric distance, and tract-based spatial statistics (TBSS based on diffusion tensor imaging). As an **exploratory aim,** we will determine which of the above MRI measurements best predict neurodevelopment (CP, cognitive and motor scales) at 24-26 months corrected age.

2. BACKGROUND

2.1 Rationale

Study Population. ELGANS are at high risk of death or NDI. In fact, of 9575 ELGANs born between 2003 to 2007 and admitted to the NICU, **death or NDI occurred in 91%, 80%, 66% and 56% of those born at 24, 25, 26 and 27 weeks gestation, respectively**.[8] These sobering statistics do *not* include infants that died before admission to a NICU, or those who died within 12 hours of admission. Major morbidities, which include CP, deafness, blindness, and mental disabilities are present in 50% of **surviving** extremely preterm infants at school age.[9-22] Long-term follow-up studies are now increasingly identifying behavioral dysfunctions such as attention deficit disorder and autism spectrum disorder.[23-27] Sequelae of extreme prematurity are a tremendous burden to the individuals, their families, and to our health care system, accounting for nearly half of the health care dollars spent on newborn care. Clearly, a neuroprotective intervention that improved outcomes for ELGANs would be profoundly beneficial both to the individual, the family and to society.[28, 29] We have chosen to study ELGANs born between 24-0/7 and 27-6/7 weeks of gestation because of their high likelihood of poor outcome, and the absence of therapeutic interventions to improve outcome. Because these infants are at such high risk of death or impairment, a strategy of prophylactic intervention is reasonable as there is great potential for benefit.[28, 29]

Restrictions: Babies born at 23-0/7 to 23-6/7 will be excluded from this study because their mortality and morbidity is too high. At this time, best estimates of death rates are 74%, and unimpaired survival rates are 1%.[8, 22] More mature babies are also excluded because their risk of poor outcomes is lower, thus prophylactic use of an unproven therapy is not indicated.

Intervention Regimen. Study infants in the Epo group will receive 6 i.v. doses of 1000 U/kg during the first 2 weeks of life when physiologic vulnerability is highest. This will be followed by maintenance dosing of 400 U/kg s.c. 3 X per week during the period of oligodendrocyte vulnerability (to 32-6/7 wks).

Dose Justification. The Epo dose, and duration of therapy chosen for this study is based upon available preclinical and clinical data for Epo neuroprotection.[30] In rodent and ovine models of neonatal brain injury, Epo doses of 1000-5000 U/kg/dose result in sustained neuroprotection, improving both short and long-term structure and function.[31-34] Higher doses are needed for neuroprotection due to the low percentage of circulating Epo that crosses the blood brain barrier.[35]

Although doses as high as 3000 U/kg/dose are being tested in preterm infants without apparent adverse effects,[36] preclinical data suggest that Epo neuroprotection has a U-shaped dosing curve, with too little or too much Epo resulting in diminished efficacy.[32, 37] In rats, repeated doses of 1000-5000 U/kg show optimal neuroprotection. To estimate how neuroprotective Epo doses in rat pups relate to human pharmacokinetics, plasma Epo concentrations were measured in extremely low birth weight infants ($\leq$ 1000 gm birth weight) after 500, 1000, and 2500 U/kg/dose.[38] Nonlinear kinetics were noted, consistent with previous studies in neonates.[39] In these infants, i.v. administration of 500 and 1000 U/kg resulted in similar peak concentrations but faster clearance than were achieved in rat pups after 5000 U/kg (Figure 2). Table 3 shows a comparison of pharmacokinetic indices in neonatal rats and humans. Doses of 1000 U/kg Epo resulted in area under the curve (AUC) measurements most similar to the most protective dose in rats.[32] The 500 U/kg dose fell short (one third to one quarter the protective AUC), while 2500 U/kg was close to three times the optimal dose in rats. Minimum steady-state concentrations (mean = 576 mU/ml) were produced using the 1000 U/kg/dose.

Figure 2. Epo concentrations in human preterms compared with rat pups given 5000 U/kg s.c. or i.p.

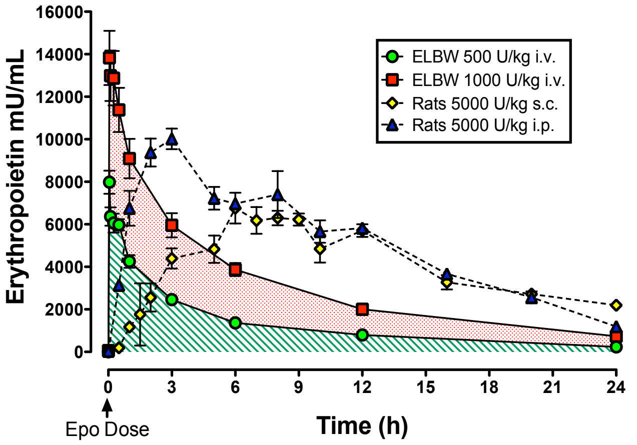

TABLE 3. Epo Pharmacokinetics

	Neonatal Rodents[40]		Preterm Infants $\leq$ 1000 gm[38]		
Epo Dose	5000 U/kg s.c.	5000 U/kg i.p.	500 U/kg	**1000 U/kg**	2500 U/kg
AUC (U*h)/L	117,677	140,331	31,412 ± 2780	81,498 ± 7067	317,881 ± 22941
Cmax (U/L)	6,224	10,015	8078 ± 538	14017 ± 1293	46467 ± 2987
$T_{1/2}$ Half-life (h)	8.4	6.7	5.4 ± 0.6	7.1 ± 0.7	8.7 ± 1.4

What constitutes clinical significance? Clinically significant studies are those that improve clinical practice and patient outcomes. Changes to clinical practice can occur slowly or rapidly depending on a number of factors: importance of the results, the size of the studies, risk of the therapy, and endorsement of medical associations such as the AAP. Ibrahim *et al.* performed a 13-item web-based questionnaire asking neonatologists what would convince them to adopt a new therapy in infants <28 weeks of gestation. The survey assumed no adverse results of treatment. Responses are shown in Table 4. We also have examples of changes in clinical practice based on published trials. Prenatal steroids reduce the risk of death or morbidity in preterm infants < 32 weeks gestation with a risk reduction of over 40%.[41] Despite this known benefit, change in clinical practice was slow, and required endorsement from ACOG. In contrast, therapeutic hypothermia for neonatal encephalopathy has been the subject of intense study, with multicenter randomized controlled trials published in 2005-2009.[42-44] A meta-analysis of these

studies reporting on 767 infants showed benefits of cooling with a significant reduction in death or NDI at 18 months: typical Risk Ratio, 0.81 (95% Confidence Intervals, 0.71, 0.93), P=0.002. The number needed to treat (NNT) ranged from 7-9 to prevent death or NDI.[45] Cooling for neonatal encephalopathy has rapidly become the standard of care. Animal studies of neonatal Epo neuroprotection using appropriate dosing have shown improvement of 49 to 79%.[30] Early clinical trials have shown safety, and preliminary benefit. Neonatologists are comfortable using this drug for erythropoiesis. We expect that Epo treatment will improve NDI-free survival compared to that seen in the ELGAN and NICHD trials. We expect that this benefit will translate into shorter and less complicated hospital stays and better neurodevelopmental outcomes. This outcome of the trial would represent an important advance in the care of ELGANs that could change the standard of care for high-risk infants. If Epo treatment has no demonstrable benefit, or if adverse effects are observed, this information will also be useful in the field because Epo is currently used anecdotally in many NICUs, without trial-based evidence, for some severely ill infants. The results of the PENUT trial will be of particular interest to pediatricians trained in neonatal-perinatal medicine. This group of practitioners is largely centered in academic or medical centers and they attend several meetings each year where new information about treatment can be presented. The PENUT trial results will be presented at regional Pediatric Research Society meetings such as the Western and Eastern Society for Pediatric Research, and at national and international meetings including the Pediatric Academic Societies combined meetings, the European Society for Pediatric Research meeting, the American Academy of Pediatrics meetings, and at other venues such as “Hot Topics in Neonatology”.

**Table 4. Survey of Neonatologists.** “Would you change practice if a study showed treatment X…”	% would change N=226
Reduced # Bayley scores < 80 by 25%	41
Reduced # Bayley scores < 80 by 50%	66
Improved Bayley scores by 10 points	56
Decreased CP by 25%	50
Decreased CP by 50%	81
Study size 200 per arm	29
Study size 400 per arm	64

Clinical Impact. Mortality of extremely preterm infants has decreased,[46, 47] but morbidity in survivors remains high due in part to increased survival of sicker infants. There remains an enormous burden of both medical and neurodevelopmental impairment in these children. Using the birth statistics for the US in 2009, we anticipate the magnitude of Epo treatment to translate as follows: with Epo treatment, the annual burden of death or severe NDI will decrease from 40% (12,226 babies) to 30% (9,170 babies, primary outcome), and that death plus moderate or severe NDI will decrease from 60% (18,340 babies) to 40% (12,226 babies, secondary outcome). We anticipate this change will derive primarily from improved neurodevelopmental outcomes. If Epo is proven to safely reduce combined morbidity and mortality of ELGANs, we anticipate a shift in neonatology practice that would improve the lives of babies and their families, and decrease the cost of healthcare.

2.2 Supporting Data

Vulnerabilities of the preterm brain. ELGANs are born during the second trimester when the fetal brain is rapidly increasing in size, shape and complexity.[48-50] Proper brain development is vulnerable to interruption by hypoxia-ischemia, oxidant stress, inflammation, and excitotoxicity, as evidenced by structural, biochemical, and cell-specific injury.[51-57] Pre-oligodendrocytes, which emerge and mature between 24 and 32 weeks of development, are particularly susceptible to injury, resulting in the WMI characteristic of preterm infants.[51, 58-60] Although the transition from fetal to early postnatal life is the period of greatest vulnerability,[61] ELGANs remain at risk for brain

injury throughout the period of oligodendrocyte development.

Epo Neuroprotection. Epo has anti-inflammatory, anti-excitotoxic,[62] anti-oxidant,[63] and anti-apoptotic effects on neurons and oligodendrocytes, and promotes neurogenesis and angiogenesis, which are essential for injury repair and normal neurodevelopment. Epo effects are dose-dependent, and multiple doses are more effective than single doses.[31, 32, 64] Epo reduces neuronal loss and learning impairment following brain injury,[65, 66] and even when initiated as late as 48-72 hours after injury, there is evidence of improved behavioral outcomes, enhanced neurogenesis, increased axonal sprouting, and reduced WMI.[67, 68]

Protective effects. Perinatal inflammation (chorioamnionitis, necrotizing enterocolitis (NEC), or sepsis) is associated with increased risk of NDI.[16, 69, 70] Microglial activation[71] and increased cytokine expression, particularly TNF-α, interleukin (IL)-6, and IL-8, have been associated with brain injury in preterm infants[72, 73] and in animal models of neonatal brain injury.[74] Epo has demonstrated anti-inflammatory effects, which may contribute to neuroprotection in the scenario of preterm birth and increased inflammatory activity.[75-81]

White matter injury. WMI is a common brain injury affecting preterm infants.[52, 56, 59] Epo decreases WMI in adult and neonatal animal models of brain injury[33, 67, 82-87] and maybe in humans.[88] Mechanisms for this may be Epo protection of vulnerable preoligodendrocytes: functional Epo receptors are expressed by immature oligodendrocytes, Epo promotes the proliferation, maturation and differentiation these cells,[89] and protects them from injury induced by interferon-γ, LPS, and hypoxic-ischemia.[67, 90, 91]

Apoptosis. Neurons in the developing brain are more likely than adult neurons to undergo apoptosis if exposed to injurious stimuli,[92, 93] and the anti-apoptotic properties of Epo may protect these vulnerable neurons.[32, 94, 95]

Repair. Epo stimulates growth factors required for normal brain growth such as brain-derived neurotrophic factor (BDNF) and glial cell derived neurotrophic factor (GDNF).[96, 97] Epo enhances neurogenesis,[31, 64, 95, 97-100] angiogenesis, repair and plasticity, thus providing long lasting neuroprotective and trophic effects.[64, 68, 98, 101-104]

Reactive Iron. Iron is highly reactive and normally sequestered by transport proteins. Unbound iron produces free radicals and subsequent oxidative injury. Preterm infants have measurable free iron, which increases after transfusions of red blood cells or during metabolic instability such as sepsis.[105-108] In our phase I/II study of Epo administration to extremely low birth weight infants we observed a transient increase in reticulocytosis, indicating an increase in iron utilization.[109] Epo may contribute to neuroprotection by decreasing free iron.

Molecular mechanisms of Epo neuroprotection. EpoR are present on neuron progenitor cells,[97] neurons,[110] astrocytes,[89] oligodendrocytes,[111] microglia,[112] endothelial cells[97] and erythrocyte progenitors. Epo has direct neuroprotective effects via EpoR binding: Epo-bound receptors dimerize to activate anti-apoptotic pathways via phosphorylation of JAK2, phosphorylation and activation of MAPK, ERK1/2, as well as the PI3K/Akt pathway and STAT5, which are critical in cell survival.[94, 112] Epo also functions through indirect effects, increasing iron utilization by increasing erythropoiesis, and by decreasing inflammation[77, 80] and oxidative injury.[113, 114]

Translational trials of neonatal Epo neuroprotection are in progress. Epo improved neurodevelopmental outcomes in two trials of term neonates with hypoxic-ischemic brain injury.[115, 116] Retrospective studies of cohorts of preterm infants that received Epo for erythropoiesis compared to controls show improved outcomes.[117-119] Two preliminary reports of preterm infants treated prospectively also show benefit: 1) preterm infants 500 to 1250 g treated with Epo (400 U/kg 3x/week) from birth to 35 weeks PMA had an average cumulative cognitive score 10 points higher than placebo/controls (98±14 Epo vs. 88±14 for controls). Epo recipients

also performed statistically better than controls on object permanence testing (Ohls, PAS 2012); 2) A phase III trial is ongoing in Switzerland to test the safety and neuroprotective efficacy of 3000 U/kg/dose x 3 doses given in the first 3 days after birth to neonates (<1500 g, < 32 weeks PMA). This group has presented preliminary data in abstract form on 100 infants showing that such dosing is safe, and that the Epo-treated group showed less WMI by MRI;[88] 3) Follow up of the infants studied in our phase I/II trial[38] shows that Epo treatment correlated with improvement of cognitive (R= .22, $p < 0.05$) and motor (R = .15, $p < 0.05$) scores.[120]

Known and potential risks of intervention: In adults, complications of prolonged Epo treatment include polycythemia, seizures, hypertension, stroke, myocardial infarction, congestive heart failure, tumor progression, and shortened time to death. None of these adverse effects have been reported in Epo-treated neonates. No prospective studies of neonatal Epo treatment have reported group differences in the incidence of neonatal morbidities, including IVH, ROP, NEC, BPD, or late onset sepsis,[121] but a Cochrane review found an increased risk of ROP with early treatment.[122] One retrospective report also described an increase in cutaneous hemangiomas in Epo-treated preterm infants.[123] Preliminary safety data from an ongoing randomized controlled trial of high dose Epo in Switzerland (PI: Hans Bucher), in which 3000 U/kg daily is given to preterm infants in the first 3 days of life has been reassuring. This group has kindly and confidentially sent results of their interim analysis of 270 of 420 planned infants. The mean gestational age for enrolled infants is 29.2, with a mean birth weight of 1201 gm. 137 subjects have been enrolled in the Epo group, and 133 in the placebo group. Sepsis occurred in 15 subjects in each group, BPD in 13 of 137 (9.4%) Epo-treated vs. 19 of 133 (14.3%) controls; ROP (grades 1-4) in 9 of 137 (6.6%) Epo-treated vs. 11 of 133 (8.3) controls; IVH in 27 of 137 (19.7%) Epo-treated vs. 23 of 133 (17.3%) controls. Furthermore, there were not significant differences in "other serious adverse events" or death (personal communication, JC Fauchere, with permission from Professor Bucher).

In summary, Epo trials in neonates for the purposes of testing its erythropoietic effect have shown it to be a safe drug for use in this population. There is robust data from preclinical animal work showing that Epo, when used at optimal doses (1000-5000 U/kg), shows short and long term improvement in brain injury that approximates 50-80%. Emerging data from prospective clinical trials of neuroprotection are promising. It is time for a definitive multicenter randomized controlled trial to test its efficacy as a neuroprotective therapy for preterm infants.

Federal oversight. This study of high dose Epo for the purposes of neuroprotection of preterm infants is registered with the FDA (IND # 12656). Dr. Juul, University of Washington, Seattle WA is the holder of the IND. The trial has been registered with clinicaltrials.gov and has been assigned Identifier Number NCT01378273.

3. STUDY DESIGN

Study Overview. This is a randomized, placebo controlled, double blind study of Epo neuroprotection in an ELGAN population. Figure 1 provides an overview of the study. 940 patients will be enrolled at 16 sites across the country in order to evaluate 752 at 24-26 months. Enrollment and initial treatment with study drug will occur by 24 hours after birth. Subjects will be randomized to either Epo treatment or vehicle, and treatment will continue until 32-6/7 weeks PMA. Short term, intermediate and long term safety measures will be determined by comparing Epo-treated and control infants. Mechanisms of Epo neuroprotection and potential biomarkers of outcome will be sought by measuring sequential inflammatory cytokines and markers of brain injury. In a

subset of subjects, a brain MRI will be done at 36 weeks PMA to determine whether Epo treatment preserves brain growth and decreases injury. After discharge from the hospital, phone contact will be made at 4-6 month intervals. Data will be collected on interval medical history and functional status. Face to face follow up will occur at two years corrected age, at which time standardized neurodevelopmental assessments will be made. The primary outcome goal is death or severe NDI at 24-26 months corrected age, with a secondary outcome goal of death, severe or moderate NDI.

4. SELECTION AND ENROLLMENT OF SUBJECTS

4.1 Inclusion Criteria

The disease or disorder under study is the neurodevelopmental outcomes of ELGAN infants at 24-26 months.

Study Goal: Prevention of neurodevelopmental impairment associated with extreme prematurity (24-0/7 to 27-6/7 weeks of gestation).

Inclusion Criteria
1. NICU Inpatients between 24-0/7 and 27-6/7 weeks of gestation
2. 24 hours of age or less
3. Arterial or venous access
4. Parental consent

When calculating gestational age, the following hierarchy will be used in order of accuracy:
1. Gestational age by *in vitro* fertilization
2. Gestational age by first trimester assessment (0 to 14-0/7 weeks)
3. Gestational age by second trimester assessment (up to 28-0/7 weeks)
5. Last menstrual period (LMP)
6. Newborn maturational assessment

We estimate, based on the ELGAN study, that 62% of subjects will have the most accurate dating based on #1 or #2, that 29% will have dating based on second trimester fetal ultrasound, 7% by menstrual dating, and only 1% by postnatal physical exam.

Postnatal age will be calculated based on time and date of birth.

Arterial or venous access is considered to be a functioning peripheral intravenous catheter, a peripherally inserted central catheter (PICC), an umbilical venous (UVC) or arterial catheter (UAC). One of these catheters must be available for the administration of study drug. It is preferable that the patient also have a catheter suitable for phlebotomy, although that is not an absolute entry criterion for the study.

To be admitted into this study, the legal guardian of the patient must give written informed consent. Each site will obtain IRB approval for the study, and will have IRB approved consent forms available in English and Spanish. A translator will be provided for Spanish speaking individuals as needed.

Demographics. There is no enrollment restriction based on gender, ethnicity or race.

4.2 Exclusion Criteria

Exclusion Criteria

1. Major life-threatening anomalies (brain, cardiac, fetal diagnosis of chromosomal anomalies)
2. Hematopoietic crises such as DIC or hemolysis due to blood group incompatibilities
3. Polycythemia (hematocrit > 65%)
4. Congenital infection
5. Prior use of Epo

As part of standard care of an extremely preterm infant, a CBC will be obtained within the first 24 hours of life. If the HCT is >65, the patient will not be eligible for the study. If the PLT count is < 50K, or if there are clinical signs of bleeding, a coagulation panel will be checked to determine whether there are signs of disseminated intravascular coagulopathy. If this condition exists, the patient is not eligible for the study.

Prior use of erythropoietin will exclude the patient from study.

Allergy/sensitivity to study drugs or their formulations will not be known in our study population given their newborn status.

Drug or alcohol use or dependence is not applicable to our patient population. Maternal drug use will not exclude a patient from the study.

All patients eligible for this study will be hospitalized in the neonatal intensive care unit (NICU). Many concurrent illnesses are expected for this population of extremely preterm infants. These include, but are not limited to: IVH, periventricular leukomalacia (PVL), hydrocephalus (HC), respiratory distress syndrome (RDS), bronchopulmonary dysplasia (BPD), hypotension, patent ductus arteriosus (PDA), NEC, early and late onset sepsis, and retinopathy of prematurity (ROP). None of these will exclude a child from study entry, but all will be considered in the safety evaluation of Epo.

4.3 Enrollment Procedures.

4.3.1 Admissions to the antepartum unit and to the NICU at each site will be screened daily for admissions that are potentially eligible for the trial. This will be done by the research coordinator or their designee. Once identified, the attending health care provider for the potential enrollee will be approached to remind them of the study so as the subject might be informed early and the subject can be notified of the study protocol. If the subject is interested in learning more about the study, the attending health care provider can notify the research coordinator or investigator so they can discuss the study with the parent and seek consent. When possible, and if there is IRB approval for this, consent will be obtained prior to delivery. Many mothers of preterm infants are admitted to an antepartum service for bed rest, fluids and observation. They are not acutely laboring, and the stress of imminent delivery is less acute. If permitted by the local IRB, these mothers will be approached during a time when they are able to fully evaluate the study and have opportunities for reflection and questions.

4.3.2 A screening log will be maintained to document patients screened, reasons for ineligibility, and reasons for nonparticipation of eligible subjects (Appendix 6). This will contain information on screening number, patient initials, date of birth, birth time, gender, race, ethnicity, gestational age,

birth weight, inborn/outborn status, eligibility (yes/no), whether they were enrolled, exclusion criteria, and if not enrolled, the reason why. These logs will be maintained in the REDCap electronic data management system. The logs will be reviewed at a regular interval to determine enrollment and reasons for non-enrollment. This information will be used to consider whether other recruitment methods should be used.

4.3.3 *Consent procedures.* Antenatal consent will be obtained when possible. Prenatally, the study investigator will obtain permission from the Maternal Fetal Medicine Attending to approach the mother to discuss the study. Postnatally, permission to approach the family will be obtained from the Attending Neonatologist. The Attending health care provider will seek parental agreement for an investigator to meet and discuss the study. If the parents are interested, the study investigator will discuss the study with family and seek consent in person. The consenting legal guardian will receive a copy of the consent form to review, and once signed, will be given a copy to keep. If the Attending physician is also a study investigator, an alternate study investigator will obtain consent, so as to avoid coercion. Investigators will only approach family after infant's attending heath care provider gives permission and family indicates that they are interested in further information about the study. No alteration in care will otherwise occur. Attending neonatologist and family can withdraw child from study at any time.

Consent will be obtained by the Investigator in a private room which ensures the privacy of the family, and which is free of potential coercive influences. Consent for participation must be obtained by the time the baby is 24 hours of age.

If a family has limited or no English speaking abilities, a certified interpreter will be provided. They will review the consent form with the family, and interpret the verbal explanation of the study during the discussion between the Investigator and the family members. If individual sites have a large population of non-English speakers, consent forms will be translated into the appropriate languages. If an interpreter is not available in a timely manner, the family will not be approached.

The parents of the research participants will be given opportunity to review the study both verbally and in writing. They will be given opportunity to ask questions of the investigator prior to giving consent. A sample consent form is shown in Appendix 10.

4.3.4 *Randomization* will take place centrally by the DCC. We will use block randomization within site using variable blocks of size 6 to 10 subjects. Using block randomization ensures that equal numbers of subjects are randomized to the intervention and control arm and that the two groups are balanced at period enrollment intervals. For multiple births (twins, triplets) all infants will be randomized the same treatment group (e.g. effective randomization of the mother). Randomization sequences will be provided to the research pharmacy at each site. Randomization will be stratified on site and on gestational age category (24-25 weeks, 26-27 weeks).

A modified intent-to-treat (ITT) approach[124] will be used, with all randomized infants who receive the first dose of study treatment to be included in the analysis. All pre-specified hypotheses will be tested using a two-sided type I error of 0.05 with no formal adjustment for multiple comparisons unless otherwise specified (such as with safety outcomes). Secondary analyses that focus on separate hypotheses will not require correction for multiple comparisons, but those analyses that use multivariate measures such as multiple brain image parameters would be corrected for multiple comparisons using standard methods.

Given that we anticipate enrollment of multiple births we require that all analyses properly account for the within-sibship correlation of outcomes. We will use Generalized Estimating Equations

(GEE), which is a versatile regression approach for the analysis of discrete and continuous outcomes.[125] Use of “robust” standard errors will provide valid statistical inference and fully account for the clustering of data.

5. STUDY INTERVENTIONS

5.1. Interventions, Administration, and Duration

This is a randomized placebo controlled trial. All patients will be treated in the NICU. Each subject will be randomized to one of two groups after establishing eligibility and obtaining signed consent for study participation and HIPAA compliance. Interventions/Treatments will be as outlined in Table 5 below. Subjects will have timed blood samples drawn over the period of study (total blood out = 2.0 mL, see Figure 3).

Figure 3. Summary of study blood draws

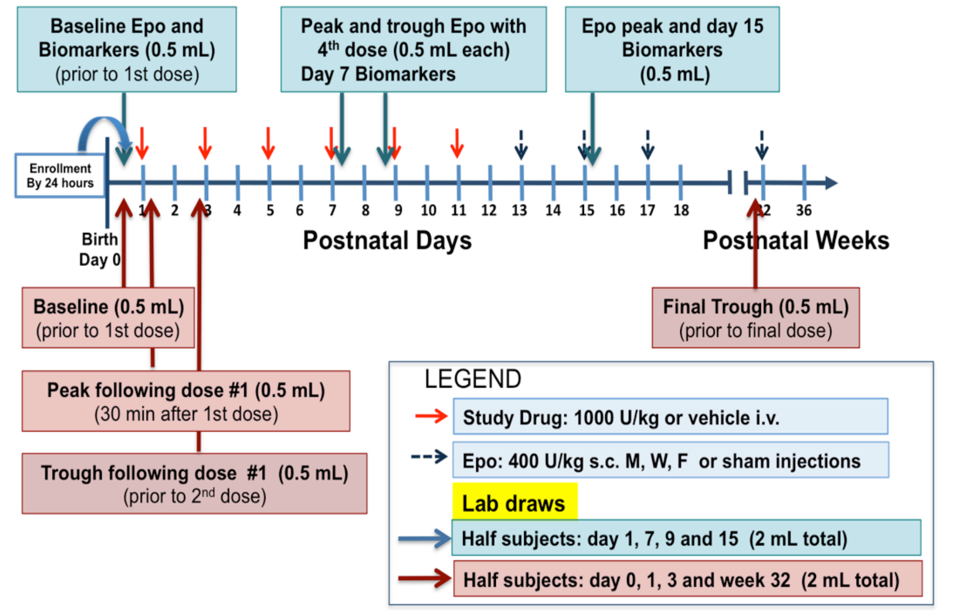

Short-term side effects in adults treated with Epo include hypertension, polycythemia, clotting, seizures and stroke. While these issues have never been reported in neonates, we will watch for them and record any SAEs. Long-term effects may include an increased risk for ROP and hemangiomas. Patients will be carefully monitored for these adverse outcomes. Adverse effects from the s.c. injection of Epo may include bruising and infection.

After written consent is obtained, but before administration of study drug, a cranial ultrasound will be done to document whether an intracranial hemorrhage has occurred prior to study drug dosing. Results of this examination will not affect clinical care, eligibility, or randomization for the study, but will be used as part of the safety analysis. All cranial ultrasounds will be read by Manjiri Dighe, MD, a UW radiologist who will be blinded to the treatment groups and any clinical information about the child. Results of this ultrasound will be available to the sites within 72 hours. (If there are clinical concerns about a subject, and a cranial ultrasound is indicated, it will be ordered by the caregiver on site, and read by the site radiologist as part of clinical care.)

Baseline Epo concentrations and circulating biomarkers (targeted inflammatory mediators and biomarkers of brain injury) will be drawn as noted in Figure 3.

Table 5: Phase III Trial

Time Line	Procedure	Sites	Control	Epo
0-24 hours from birth	Consent	All	All	All
Consent to 24 hours	Randomization	All	All	All
Consent to 24 hours	Head Ultrasound	All	All	All
Consent to 24 hours	Baseline blood draw	All	0.5 mL	0.5 mL
Study day 1	Epo dose 1 i.v.	All	Vehicle	Epo 1000 U/kg
Study day 1	Epo Peak after dose #1	Half	0.5 mL	0.5 mL
Study day 3	Epo Trough after dose #1	Half	0.5 mL	0.5 mL
Study day 3	Epo dose 2 i.v.	All	Vehicle	Epo 1000 U/kg
Study day 5	Epo dose 3 i.v.	All	Vehicle	Epo 1000 U/kg
Study day 7	Epo dose 4 i.v.	All	Vehicle	Epo 1000 U/kg
Study day 7	Epo Peak + biomarkers	Half	0.5 mL	0.5 mL
Study day 8	Begin iron supplementation per guidelines	All	All	All
Study day 9	Epo Trough + biomarkers	Half	0.5 mL	0.5 mL
Study day 9	Epo dose 5 i.v.	All	Vehicle	Epo 1000 U/kg
Study day 11	Epo dose 6 i.v.	All	Vehicle	Epo 1000 U/kg
Study day 13	Begin maintenance Epo vs. sham injections (nothing)	All	No injection	Epo 400 U/kg
Study day 15	Epo Peak + biomarkers	Half	0.5 mL	0.5 mL
Continue study drug until 32-6/7 wks PMA	Begin maintenance Epo vs. sham injections (nothing)	All	No injection	Epo 400 U/kg
Study day x at 32-6/7 wks PMA	Final Epo dose s.c.	All	No injection	Epo 400 U/kg
32-6/7 wks PMA	Epo Trough	Half	0.5 mL	0.5 mL
36-0/7 wks ± 2/7 days	MRI	MRI sites	110 per arm	110 per arm
Discharge	Discharge exam and questionnaire	All	All	All
4 m corrected age	Phone contact	All	All	All
8 m corrected age	Phone contact	All	All	All
12 m corrected age	Phone contact	All	All	All
18 m corrected age	Phone contact	All	All	All
24 m corrected age	Bayley III, neuro exam, M-CHAT	All	All	All

Epo Risks: In adults, complications of prolonged Epo treatment include polycythemia, seizures, hypertension, stroke, myocardial infarction, congestive heart failure, tumor progression, and shortened time to death.

None of these adverse effects have been reported in Epo-treated neonates. No prospective studies of neonatal Epo treatment have reported group differences in the incidence of neonatal morbidities, including IVH, ROP, NEC, BPD, or late onset sepsis,[121] but a Cochrane review found an increased risk of ROP with early treatment.[122] One retrospective report also described an increase in cutaneous hemangiomas in Epo-treated preterm infants.[123]

In the 2 pilot studies of high dose Epo administered to preterm infants done at the University of Washington Medical Center and in Switzerland, no increased risk of any complications were noted in a total of 60 treated patients, including ROP.[36, 38] In our study, we observed 5 cases of severe ROP in 23 surviving controls compared to 6 of 24 Epo treated infants.[38]

Preliminary safety data from an ongoing randomized controlled trial of high dose Epo in Switzerland (PI: Hans Bucher), in which 3000 U/kg daily is given to preterm infants in the first 3 days after birth has been reassuring. This group has kindly, and confidentially, sent results of their interim analysis of 270 of 420 planned infants. The mean gestational age for enrolled infants is 29.2, with a mean birth weight of 1201 gm. 137 subjects have been enrolled in the Epo group, and 133 in the placebo group. Sepsis occurred in 15 subjects in each group, bronchopulmonary dysplasia (BPD) in 13 of 137 (9.4%) Epo-treated vs. 19 of 133 (14.3%) controls; ROP (stages 1-4) in 9 of 137 (6.6%) Epo-treated vs. 11 of 133 (8.3) controls; IVH in 27 of 137 (19.7%) Epo-treated vs. 23 of 133 (17.3%) controls. Furthermore, there were not significant differences in "other serious adverse events" or death (personal communication, JC Fauchere, with permission from Professor Bucher).

Epo is a potent erythropoietic growth factor. Thus, high doses of Epo given for neuroprotective treatment might be expected to increase erythropoiesis, and possibly megakaryocytopoiesis. In neonatal rats, there is a transient increase in hematocrit following high-dose Epo,[66] but in preterm infants, while 3 doses of high dose Epo increased reticulocytosis, they did not affect hematocrit, likely due to early phlebotomy losses.[38] In term infants with neonatal encephalopathy, after up to 6 doses of high dose Epo, hematocrit fell an average of 14%, reflecting phlebotomy losses in these sick infants (NEAT trial, manuscript in press, *Pediatrics*).[126] The effect of brief treatments of high-dose Epo on iron balance is not known. Prolonged Epo treatment in neonates must be accompanied by iron supplementation, and does improve erythropoiesis. This is, in fact, the primary indication for Epo use in neonates. Dosing of 400 U/kg/dose three times a week has been shown to decrease (but not eliminate) need for blood transfusions.[127]

The potential contribution of Epo to the development of ROP in preterm populations is controversial. ROP occurs in two phases, the first involving a loss of retinal vasculature following birth, and the second involving uncontrolled proliferation of retinal vessels. Epo receptors (EpoR) are present on endothelial cells, and Epo stimulation increases their angiogenic expression.[128] Early high-dose Epo might theoretically have a protective effect on the retina by ameliorating the first stage of ROP. Alternatively, the angiogenic properties of Epo may prevail, resulting in an increase in ROP. The Cochrane meta-analyses of prospective studies of Epo (for which ROP was not a primary outcome measure) showed an increase risk of ROP after early Epo exposure.[122] This analysis could not separate out the potential effects of anemia or iron treatment as confounders.[129] Animal data suggest timing of Epo exposure might be very important. In a mouse model of ROP, early Epo treatment decreased the development of ROP, while late treatment given during the proliferative stage contributed to neovascularization and disease.[129] In contrast, using a rat model of ROP, no beneficial or harmful effects of repeated high-dose Epo

administration (5000 U/kg x 3 doses) on retinal vascularization were observed.[130] Transfusions have also been associated with ROP, and Epo treatment decreases (but does not eliminate) transfusion exposure.

Three Cochrane reviews have been published reviewing the safety and efficacy of Epo for erythropoietic purposes.[122, 131, 132] Conclusions from these reviews are as follows: Late Epo reduces the use of one or more RBC transfusion, the number of RBC transfusions per infant and the total volume of RBC transfused per infant. There was no increase in any of the complications of prematurity noted, i.e. use of Epo is safe in this population of preterm infants. There is no advantage of early vs. late Epo to promote erythropoiesis, although both reduce the number and volume of transfused blood needed. Early Epo with iron is associated with an increased risk of ROP (RR 1.18 overall, and 1.71 for stage 3). The authors state that "The increased risk for ROP may be associated with use of higher doses of supplemental of iron in the Epo group than in the control group." It is notable that there was an unfortunate error in this Cochrane Review showing that early Epo treatment of infants was associated with retinopathy of prematurity.[122] One of the studies included in this meta-analysis was misclassified as being during the first week of life when Epo was begun, when it in fact was during the second week of life (Romagnoli et al. European journal of pediatrics. 2000;159(8):627-628).[133] It should have been included in the meta-analysis for late Epo treatment of infants, i.e., after the first week of life.[131] Moving this study to the appropriate meta-analysis results in no significant effect of Epo on ROP either early or late. This will be corrected in the updated reviews (personal communication).

1) Safety during the high dose treatment period. Serious adverse events (SAEs) specific to Epo will be defined as any of the following during the treatment period: major symptomatic venous or arterial thrombosis (involving a major vessel not related to an infusion line); polycythemia (central hematocrit > 60%, or hematocrit increase ≥ 15% not due to red blood cell transfusion); hypertension (systolic arterial pressure > 70 for over 30 minutes within the first 5 days of postnatal life, > 90 from day 6-14);[134] or unexpected death. We will also consider ROP as a SAE. Subjects will be screened for ROP times as per the 2006 guidelines[135]: for 24-27 week gestation infants, the first screening will occur at 31 weeks PMA. The timing of follow up exams will be determined by findings on the initial exam. Additional relevant SAEs defined by the FDA include: a life-threatening event, hospitalization (initial or prolonged), disability or permanent damage, required intervention to prevent permanent impairment or damage, and other important medical events. This latter category includes any SAE that "does not fit the other outcomes, but the event may jeopardize the patient and may require medical or surgical intervention to prevent one of the other outcomes". SAE analysis uses a doubling of rates to trigger consideration of DSMB action irrespective of statistical significance at interim analysis, yet formal monitoring will be conducted every 6 months to evaluate the strength of statistical evidence for a difference across groups. Therefore, our proposed interim analyses will evaluate the accruing data using both formal statistical comparison in addition to consideration of absolute rates and is thus more conservative than use of only one criterion. Finally, we plan to conduct interim analysis that also report on the specific SAEs and AEs listed in Tables 10 and 11, and will provide p-values comparing treatment groups for these safety outcomes in addition to the SAE outcomes. A medical safety officer not involved in the study will review all SAEs, and the DSMB (appointed by NINDS) will decide when they want to review each type of SAE or AE. We recommend that each case of ROP reviewed as SAE in real time.

Criteria for Withholding/Stopping the Study Drug. Confirmed neutropenia (ANC <500/µL), central hematocrit (Hct)>60%, severe sepsis (defined as culture-proven sepsis requiring pressor or new ventilatory support), unexplained seizure, symptomatic clot, or hypertension (blood pressure 2 SD > mean for age.[134, 136] Study drug will be restarted when neutrophil counts increase

over 500/μL, Hct <45. In the case of sepsis, study drug will be interrupted for 3 days and then restarted. Parenteral iron will not administered while study drug is held. The rationale for withholding study medication is to hold iron during culture-proven sepsis. Iron has been reported to be a growth factor for selected bacteria and might worsen the patient's condition during sepsis.[137]

All necessary medical interventions will be available in the event of adverse events stemming from a subjects involvement in research. All adverse events will be monitored closely until resolution, and they will be recorded and reported to the local IRB as well as the DSMB.

2) Safety during the initial hospitalization. Due to the inherent vulnerability of this patient population, the rate of comorbidity is high. The most current week-specific data for large populations of similar infants are provided by the neonatal research network (9575 infants of extremely low GA (22–28 weeks),[8] and by the ELGAN study group (1506 infants 23-27 weeks).[47, 138-141] We will use standard definitions of respiratory distress syndrome (RDS), BPD, IVH, PVL, early-onset and late-onset sepsis, NEC, patent ductus arteriosus (PDA), clinically diagnosed seizures (confirmed by EEG), and ROP.[8] We will track the incidence of each of these disorders for all enrolled subjects using RedCAP data entry forms (selected draft forms are shown in Appendix 6). The DSMB will monitor the rates of these comorbidities at predetermined intervals, comparing the rates in treated and control infants. Expected rates will be based on the published literature. For example, in the Network trial, 93% had RDS, 46% PDA, 36% had any IVH with 16% severe, 11% NEC, 36% late-onset sepsis, 12% ROP. In the ELGAN study, 21% died by 2 years of age, 24% of all children had an IVH, 12% had moderate/severe ventriculomegaly, 24% had an ultrasound lesions (echodense or lucent).[139]

3) Long term outcomes. Neurodevelopmental outcomes will be documented as noted in Aim 1. In addition, at each phone interview, and at the face to face visit, data will be obtained regarding interval hospitalizations, medication use, resource utilization, and oxygen requirement.

<u>Safety Analysis</u>: For each SAE we will tabulate the event rate by treatment group, and then compare rates using Fisher's exact test.

Blood draws: Each subject will have scheduled blood draws for Epo levels as shown in Figure 3 (2.0 mL total per subject over the length of the study). This combination will minimize the blood draws for each subject, but will provide population pharmacokinetic data and documentation of Epo administration. Blood draws will be synchronized with the clinically needed blood samples when possible. Epo measurements will be done in all subjects, and cytokine measurements (Aim 3) will be done in a randomly selected subset of 200 subjects (100 per arm). Following serum ferritin (0.4 mL) or ZnPP/H (0.1 mL) to assess iron status is recommended, particularly for those minority of infants that require parenteral iron. Up to 3 blood draws for this may be performed during the Epo treatment period. This would increase the total blood volume drawn for study purposes by 0.3 (if ZnPP/H is assessed) to 1.2 additional mL of blood (if ferritin is assessed). These volumes (Maximum of 3.2 mL) are within the IRB recommendations for infants of this weight: "Existing guidelines for blood sample volume limits (ranging from 1–5% of total blood volume within 24 hours and up to 10% of total blood volume over 8 weeks) are consistent with the limited evidence available on "minimal risk" to children".[142] Given that the blood volume of premature infants ranges from 80 to 85 mL/kg, their total blood volume would range from 40 mL (80 x 0.5 Kg) to 85 mL (85 x 1.0 Kg). The maximum amount of blood to be drawn for study purposes is 2.0 + 1.2 mL = 3.2 mL, which is within the guidelines.

Blood draw precautions: If there is a venous or arterial catheter in place appropriate for blood

drawing, this will be used preferentially. If no access is available, standard neonatal phlebotomy techniques will be used.

Cerebral Spinal Fluid (CSF): In the event that a spinal tap is clinically indicated to rule out meningitis, we will request 0.15 mL CSF be saved for measurement of Epo concentration in any infant who undergoes a diagnostic lumbar puncture within 48 hours after an Epo dose is given. A blood Epo level (0.15 mL) will also be performed within 15 minutes of when the CSF sample is collected Epo measurements will be made using the R&D Epo ELISA. If significantly elevated Epo concentrations are present, further analysis, either by Western blotting or MALDI TOF mass spectrometric measurement will be done.

Cranial Ultrasound: In addition to routine screening head ultrasounds,[143] subjects enrolled in this study will undergo one additional ultrasound prior to study drug dosing. This will be part of the safety analysis for this study. Intracranial hemorrhage occurs commonly in this patient population, with 36% showing either bleeding, ventriculomegaly, or other abnormalities.[8] While it is likely that randomization will evenly distribute infants with intracranial bleeding into both groups, intracranial hemorrhage is a critical factor that influences outcomes. Infants with intracranial hemorrhage may also be more responsive to Epo neuroprotection.[118] Thus it is critical to document the presence or absence of bleeding prior to drug treatment.

Brain MRI: *A subset of 220 subjects will undergo a brain MRI at 36 weeks PMA.* Prior to scanning, infants will be fed and swaddled or immobilized using a MedVac Infant Vacuum Immobilization Bag (contour Fabricators, Inc., Fenton, Michigan).[144] Ear plugs, and ear covers [MiniMuffs® (Natus Medical Inc., San Carlos CA)] will provide noise reduction. Exams will be rescheduled if the child cannot be quieted sufficiently for the exam. Subjects will be monitored with an MRI-compatible pulse oximeter and electrocardiograph monitor throughout the duration of the scan. A caregiver trained in MRI procedures and neonatal resuscitation will be in attendance.

5.2 Handling of Study Interventions

Epo Quality control. To minimize lot variability across sites and over time, the central coordinating site (UW) will purchase single dose, preservative-free vials of Procrit®, Recombinant Epoetin Alfa, Amgen, 2000 Units/mL from a single lot, every 4-6 months to distribute to the participating centers. A pharmacy manual of operations, and accountability records will be maintained at each site. Saline will be used as placebo.

Blinding/Drug Administration. After enrollment the DCC will provide randomization information to the site pharmacy. The site pharmacy will then dispense study drug to the research nurse in a closed container. Epo and placebo (saline) are the same in appearance, and will be labeled “study drug”. The first 6 doses of study drug will be administered i.v. over 5 minutes. These i.v. doses can be administered either by the bedside nurse or a research nurse since the study drug will be blinded by the pharmacy (Epo and saline have the same appearance, and the syringe will be labeled “study drug”). Maintenance Epo will be administered by s.c. injection on Monday, Wednesday and Friday, beginning on the day closest to completing the high dose series. To maintain blindness, pharmacy will issue “study drug” to control infants in a clear syringe, but these infants will not undergo any maintenance injections. Treatments will be administered behind blinds by a research nurse. After injection or sham injection, all infants will have a small adhesive bandage placed over the “injection site”. The time, date, and study drug intervention will be documented for each drug administration event.

The risks of intravenous administration study of medication include infiltration, infection, possibly bleeding. The risks of subcutaneous injections include bruising, pain at the site of injection, and infection.

Iron supplementation: Iron supplements (oral and intravenous preparations) will be obtained from each site pharmacy as per their usual practice.

Iron Treatment Plan. When enteral feedings are started, a standard iron containing formula will be used if breast milk is unavailable. Once infants (all subjects) have an enteral intake of 60 mL/kg/d, they will be started on enteral iron at a dose of 3 mg/kg/d. Enteral iron will be increased to 6 mg/kg/d when infants achieve an enteral intake of 100 mL/kg/d.[145] This is a well tolerated dose that helps to prevent iron deficiency in preterm infants.[145] Serum ferritin ***or*** ZnPP/H ratios should be followed every two weeks, and iron adjusted accordingly. If the serum ferritin or ZnPP/H values are in the normal range (ferritin-15 to 200; ZnPP/H-30 to 183), 6 mg/kg/day will be administered for all subjects. However, if there is evidence of iron deficiency despite this (ferritin<15; ZnPP/H>184), the iron dose may be increased up to 12 mg/kg/day to maintain iron sufficiency. Iron should be held for Ferritin > 400.

If the subject is not taking oral/enteral feeding volumes totaling 60 mL/kg/day by study day 8, all subjects will receive 1.5 mg/kg/dose once a week of parenteral iron (iron dextran or iron sucrose)[146] by intravenous infusion until they achieve an enteral intake of 60 mL/kg/d.[127] Iron dextran can be added to the parenteral nutrition solution and administered over 24 h, or diluted in several milliliters of 10% dextrose in water or normal saline and administered over 4 h. This dose is within the range of normal requirements for growing premature infants, and is meant to ensure that iron deficiency is not a confounding factor for neurodevelopmental outcomes in this study. If subjects are made NPO at a later point in their course, parenteral iron should be used if no feedings are given for a period of 7 days or greater. We estimate that less than 20% of babies will receive one parenteral dose of iron, since feeding are most often started by day 2 or 3, and advanced by 10 to 20 mL/kg/day. More than one parenteral dose will be rare.

Magnetic Resonance Imaging. Please see Appendix 8 for pulse sequences.

MRI’s will be obtained on 110 subjects from each study arm at 36 weeks PMA. Prior to scanning, infants will be fed and swaddled or immobilized using a MedVac Infant Vacuum Immobilization Bag (contour Fabricators, Inc., Fenton, Michigan).[144] Ear plugs, and ear covers [MiniMuffs® (Natus Medical Inc., San Carlos CA)] will provide noise reduction. Exams will be rescheduled if the child cannot be quieted sufficiently for the exam. Subjects will be monitored with an MRI-compatible pulse oximeter and electrocardiograph monitor throughout the duration of the scan. A caregiver trained in MRI procedures and neonatal resuscitation will be in attendance.

All sites are accomplished in obtaining neonatal MRI scans. We have previous experience optimizing and acquiring data in 7 month old infants using a quiet version (low gradient sound amplitude) of all the scans needed for this project, as part of a project with PI Patricia Kuhl at the Institute for Language and Brain Sciences. In this project we were able to acquire scans in sleeping infants and also test the resulting data quality using the software FreeSurfer (Martinos Center for Biomedical Imaging, Charlestown, Massachusetts).

Automated Developing Tissue Segmentation: Analyses will be based on automated processing steps developed for specifically for analysis of early developing brain anatomy, as part of separate on-going R01 projects on fetal and premature neonatal brain image analysis, directed by Dr. Studholme (R01 NS 061957 and R01 NS 055064). Validated automated techniques will

segment gray matter, myelinated, and un-myelinated white matter using 3D T1W MRI data. These make use of age-specific spatial statistical priors synthesized from a computational spatio-temporal atlas[147, 148] that models age-related variations in tissue volume, tissue probability, and MRI contrast for an automated expectation maximization based labeling scheme, illustrated in Figure 4.

Figure 4. Preliminary results using a specialized template driven expectation maximization (EM) scheme (left) to label T1W MRI data of a premature neonate (right) with developed and developing tissues (cyan-myelinated white matter boundary, blue-unmyelinated white matter boundary, red-CSF boundary

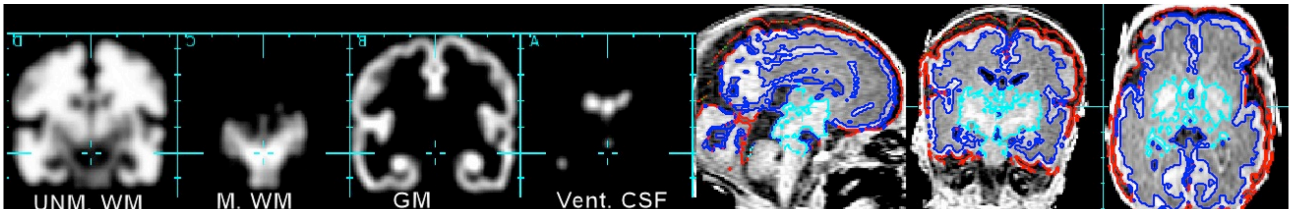

Figure 5: Left: Example delineation of the preterm cerebellum to create a set of 10 reference MRI scans with delineated anatomy. Right: 3D Rendering illustrating initial gyral marking protocols and techniques applied to a pair of MRI studies of a single infant acquired at 32 and 38 wks PMA.

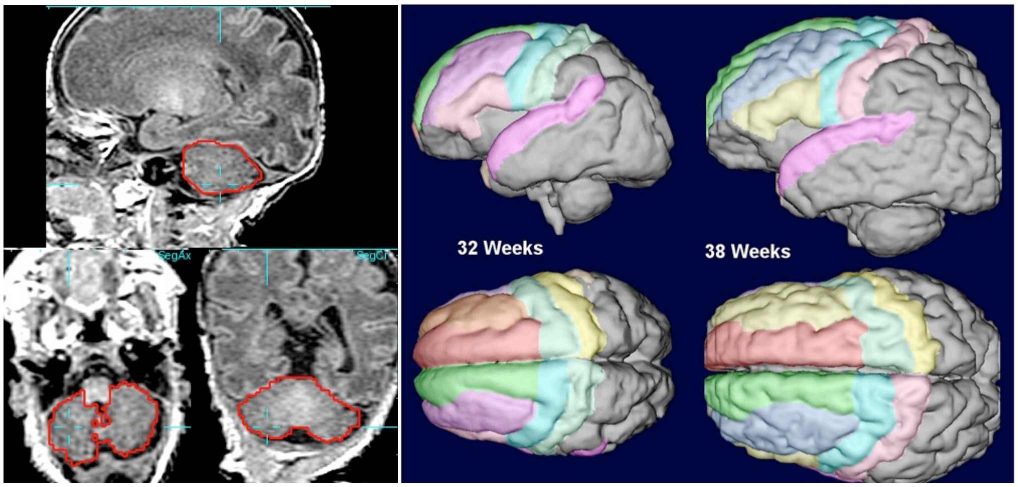

Following tissue segmentation, anatomical regions will be automatically labeled (Figure 5) using fine scale unbiased spatial normalization to form an unbiased minimum deformation group average anatomy.

Spatial Normalization for population based analyses. Accurate spatial normalization of developing anatomies allows population-based mapping of structural differences in subjects. For structural image analysis we will use template-free, symmetric, groupwise, spatial normalization of anatomies to form an unbiased average anatomy from all scans being studied. This will make use of accurate, automated developing brain tissue segmentation techniques to align only boundaries present in each subject's anatomy and exclude focal pathological variations in tissue contrast such as blood clots that may be present in some anatomies and potentially induce incorrect mappings between brain structures.

Evaluation of Global and Regional Tissue Growth (GRTG) summary measures. We will first use the automated tissue segmentations to evaluate summary measures for global and regional growth rates in each subject for conventional statistical analyses. Gray matter, myelinated and unmyelinated white matter will be estimated by integrating tissue label probabilities over the whole

brain and within a set of pre-defined brain regions. Each MRI will be automatically labeled with an optimized patch-based brain labeling technique[149] based on a subset of manually delineated MRI scans that are aligned to form a statistical model of the labeling. The labeling approach combines multi-atlas based methods with patch-based techniques to optimally assign region labels to each new individual MRI scan. The resulting label maps combined with tissue segmentations provide summary measures that can be analyzed using linear modeling described in more detail in the DCC.

Evaluation of patterns of tissue growth using Tensor-Based Morphometry (TBM) of Structural MRI. We will use TBM to look for spatially localized biomarkers of abnormal brain tissue growth across the population using techniques developed and validated in fetal brain imaging.[48] The local volume changes induced by the mapping from the group average to each individual brain will be used to characterize the developing brain anatomies. These will be evaluated from the Jacobian determinant of the deformation at each voxel.[150] We will apply general linear modeling to analyze these maps to account for age and other covariates together with main outcome measures on a voxel-by-voxel level, generating regression coefficients and associated t-statistics at every voxel in the average anatomies by spatial parametric mapping.[151] Correction for multiple comparisons will be achieved using an efficient parallel implementation of non-parametric permutation testing.[152] This analysis permits a spatial hypothesis free exploration of how local tissue growth rates are related to outcome and treatment variables across the study population.

Evaluation of Cortical Folding using Surface Curvature Mapping (SCM). We will use the automated segmentations of cortical tissue boundaries to evaluate the local and global state of neonatal brain folding to look for perturbations in cortical development, using techniques our group has previously used in both fetuses and premature neonates.[153, 154] Each cortical surface will be tessellated into a locally topologically-correct triangular mesh[155] and a set of normalized curvature measures will be evaluated at each point on the surface to provide direct quantitative measures of cortical evolution. Each subject's surface folding map will be transformed into the unbiased population average anatomy using the deformations described above for TBM analysis. These are mapped onto a common tessellated average surface[153] to allow statistical parametric mapping using a vertex-by-vertex general linear model. As with TBM, this generates a map of regression coefficients and t-statistics (here on a surface of vertex locations), which is corrected using an efficient parallel implementation of non-parametric permutation testing,[152] again permitting a spatial, hypothesis-free exploration of how local and cortical folding is related to outcome and treatment variables across the study population. Figure 4 shows an example of changes in surface folding over time in the developing fetus.

MRI Analysis. All MRI images will be sent electronically to UW for analysis, where they will be will be converted to DICOM format and stored on the DICOM server. Dr. Richards will write customized software to prepare data for analysis at the UW site such as reformatting, resorting, extraction of diffusion b-values, b-vectors for tensor calculations. He has already written b-values, b-vector extraction code for the Philips Acheiva scanners but will need to customize code for the Siemens scanners. Standardized phantoms (Magphan® Quantitative Imaging Phantom (ADNI)) will be used at each site to establish data integrity for quality control of MR data.

Qualitative image analysis will be performed by the clinical neuroradiologist at each participating site so the information is available to the subject's care team. For study purposes, each MRI will be evaluated by Drs. Dennis Shaw and Manjiri Dighe, who will be blinded to the treatment group, and all clinical data. Qualitative evaluation will involve rating each scan on a 3 point scale (1= normal; 2 = possibly abnormal; 3 = abnormal) for the following: Abnormal signal in basal ganglia and thalamic nuclei, internal capsules, periventricular white matter, cortex and subcortical regions:

presence of hemorrhage, or areas of reduced diffusion (see Table 6 below). Evidence of DEHSI, areas of atrophy or cyst formation will be documented.[1, 156-158]

Table 6. Qualitative Scoring System for MRI Evaluation. [1]

Table 1 White and grey matter scores

	Grade 1/ Score 1	Grade 2/ Score 2	Grade 3/ Score 3
WM signal changes	Normal<br>23/51 (45%)	Focal (one region)<br>14/51 (27.5%)	Extensive (> one region)<br>14/51 (27.5%)
Reduction in WM volume	Normal<br>24/52 (46%)	Mild<br>8/52 (35%)	Diffuse loss<br>10/52 (19%)
Cystic changes	Normal<br>50/51 (98%)	Focal (one region)<br>1/51 (2%)	Extensive (> one region)<br>0/51 (0%)
Myelination (M1-M4[#])/C callosum (C.c)	M3–M4/ normal C.c. or partial thinning of C.c.<br>36/52 (69%)	M2/ marked thinning of C.c.<br>16/52 (31%)	Impared PLIC myelination (M1) and marked thinning of C.c.<br>0/52 (0%)
Ventricular dilatation	Normal<br>27/52 (52%)	Mild-moderate<br>22/52 (42%)	Marked dilatation<br>3/52 (6%)
Subarachnoid space	Normal<br>35/52 (67%)	Mildly enlarged<br>14/52 (27%)	Moderate-severely enlarged<br>3 (6%)
Cortical grey matter signal abnormalities	Normal<br>50/51 (98%)	Focal (one region)<br>1/51 (2%)	Extensive (> one region)<br>0/51 (0%)
Gyral maturation	Normal<br>34/52 (65%)	Delayed 2–4 weeks<br>18/52 (35%)	Delayed > 4 weeks<br>0/52 (0%)

Myelination stage 1 (corresponding to a maturation before 37 weeks GA): Myelin in the brainstem, vermis and cerebellar hemispheres
Myelination stage 2 (37–38 weeks GA): Myelin in the posterior limb of the internal capsule and the lenticular nucleus and thalamus
Myelination stage 3 (38–39 weeks GA): Myelin in the central corona radiate and the caudate nucleus
Myelination stage 4 (40–42 weeks GA): Myelin in centrum semiovale

Quantitative analysis. We will use a range of highly sensitive automated techniques to quantitatively assess brain development at 36 weeks PMA using MRI analysis tools developed as part of on-going projects in the biomedical image computing group at UW (http://depts.washington.edu/bicg) led by Colin Studholme, PhD. We will examine global and regional summary measures of tissue volume,[159] cortical folding[154] and microscopic water diffusion properties from each subject scan to examine whether the state of overall brain growth at 36 weeks PMA has been modified by the administration of Epo. We will then explore the possible presence of focal differences in growth patterns that may be related to specific functional deficits. This will use groupwise spatial normalization to examine the spatial pattern of tissue volume increase,[48] and water diffusion, together with patterns of cortical surface folding.[153]

DTI Analysis: The methodology detailed below is currently used by our research group at the University of Washington's Diagnostic Imaging Sciences Center (DISC).

Preprocessing. DTI quantification will be preceded by head motion and eddy current correction using affine registration to a reference volume[160] with FDT (FMRIB's Diffusion Toolbox; http://www.fmrib.ox.ac.uk/fsl/fdt/index.html). Using the field maps, B_0-field inhomogeneity induced geometric distortion in the eddy current-corrected images will be corrected with PRELUDE (phase Region Expanding Labeller for Unwrapping Discrete Estimates)[161] and FUGUE (FMRIB's Utility for Geometrically Unwarping EPIs; http://www.fmrib.ox.ac.uk.offcampus.lib.washington.edu/fsl/fugue/). FSL software **DTIFIT** will be used to fit the diffusion tensor model at each voxel inorder to calculate DTI eigen vectors, eigen values, fractional anisotropy, axial diffusivity, radial diffusivity, and mean diffusivity (http://www.fmrib.ox.ac.uk/fsl/fdt/fdt_dtifit.html).

Development of 36 Week Infant Head Model for MRI. We will use software (MINC) developed at the Montreal Neurological Institute (http://www.bic.mni.mcgill.ca/ServicesSoftware/MINC) in order

to co-register and combine 50 MRI structural brain images of normal infants at 36 weeks in order to make head model to be used in DTI and VBM group comparisons Dr. Richards has experience using this software at I-LABS in collaboration with Dr. Patricia Kuhl where he used MINC to make a head model for 6 month old infant brain using 43 brains.

Evaluation of white matter integrity using DTI. Group differences in fractional anisotropy (FA), axial diffusivity, radial diffusivity, mean diffusivity (similar to ADC) will be determined using TBSS (Tract-Based Spatial Statistics).[162] TBSS was developed to conduct voxelwise analysis of multi-subject diffusion data utilizing improved non-linear registration techniques. An example of a 6 month old child studied at the University of Washington is shown in Figure 6. Data processing will be conducted according to the standard method detailed in the TBSS instruction manual (http://www.fmrib.ox.ac.uk/fsl/tbss/index.html). We plan to use Randomise, a permutation method, to test for between-group differences in these DTI measures. Correction for multiple comparisons will be done using whole brain cluster-based thresholding method: voxel height, $p < 0.01$; cluster extent, $p < 0.05$. Our group is well published in this area of diffusion imaging, DTI and DTI analysis.[163-166] Statistical comparison for TBSS will be performed using FSL-software called Randomise (http://www.fmrib.ox.ac.uk/fsl/randomise/index.html). This program will be used to perform a non-parametric voxel by voxel ANOVA (and correlations with clinical score) with multiple comparison correction using the Threshold-Free Cluster Enhancement option. Randomise is a permutation program enabling modeling and inference using standard General Linear Model design matrix setup using cluster-based tests. For more detail on permutation testing in neuroimaging see Nichols and Holmes.[152] The multiple comparison correction was performed based on cluster statistical characteristics such as extent of cluster size. Dr. Richards has written software to create the design matrix that will be used for both the ANOVA and clinical score correlations in software Randomise.

Figure 6. TBSS

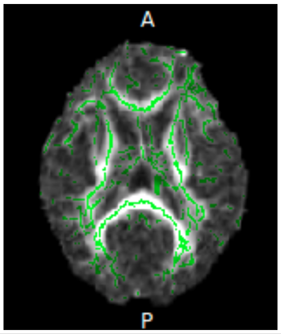

As part of the overall QA effort, we will examine various measures of study implementation across sites. In particular, recruitment, retention, data completeness, and measurement precision will be tabulated and compared across sites and will be included in our web-based reports. QA efforts and site visits will be focused on any sites that show evidence of problems.

Inflammatory Mediators. To test whether Epo treatment decreases serial measures of circulating inflammatory mediators, and biomarkers of brain injury.

Circulating Proteins: Blood will be drawn from 100 randomly assigned subjects in each treatment group on days 1, 7 and 15 for measurement of potential biomarkers. We will use Meso Scale Discovery (MSD) technology to measure the following inflammatory markers and growth factors: BDNF, interferon-gamma (IFN-γ), interleukin-1 beta (IL-1 β), IL-6, IL-8, IL-10, IL-12p70, tumor necrosis factor-α (TNF-α), and monocyte chemotactic protein-1 (MCP-1). Markers of neurotoxicity and injury will include: S100B, glial fibrillary acidic protein (GFAP), neuron specific enolase (NSE), Activin A, and Ubiquitin C-terminal hydrolase-L1 (UCH-L1).[167-169] This combination of factors will evaluate the subject's inflammatory state over time, provide insight as to the timing and severity of brain injury, and potentially, provide insight as to why some individuals might respond more optimally to Epo.[141, 167, 168, 170-172] Sample timing is based on the published data in ELGANs.[141, 171]

A number of circulating proteins have been evaluated as potential biomarkers of brain injury. Promising biomarkers include the GFAP, Ubiquitin C-terminal hydrolase-L1 (UCH-L1), myelin basic protein (MBP), S100B, Activin A, and neuron-specific enolase (NSE), although the sensitivity, specificity and timing of elevation after injury in preterm infants have yet to be determined. All of these proteins, with varying levels of specificity, are elevated after brain injury in studies of adult or neonates. Due to the immaturity of myelination, MBP is unlikely to be useful in preterm infants.

GFAP is a specific marker of differentiated astrocytes and increased circulating concentrations are detectable within hours and peak at up to four days after an ischemic stroke.[167-169, 173] Astrocyte foot processes make up the sub-endothelial component of the blood-brain barrier (BBB), so elevations of GFAP can also reflect loss of BBB integrity. UCHL1 expression is highly specific to neurons and to cells of the neuroendocrine system. Elevations have been demonstrated in term infants with HIE. S100B belongs to a family of calcium handling proteins and is localized predominately in glial cells in brain. It is thought to function in neurite extension, inhibition of PKC-mediated phosphorylation, axonal proliferation, and inhibition of microtubule assembly. NSE is a glycolytic enzyme localized to primarily to neurons. However NSE is also expressed in platelets and erythrocytes, so elevations may not be specific to brain injury. GFAP has not been studied in the context of preterm brain injury, but a direct comparison of GFAP, S100B, and NSE showed that GFAP was a superior biomarker of brain injury in the setting of traumatic brain injury.[174]

Activin, is a member of the transforming growth factor-β superfamily and is expressed by neurons. It is a trophic factor that regulates differentiation and proliferation of neurons. (Florio et al., 2007) In a cohort of 53 infants, Florio et al. (2006) showed that high concentrations of Activin A (>0.8 mcg/L) in blood samples drawn during their first hour of life was highly correlated with IVH in preterm infants <32 weeks gestation (100% sensitivity of 100%, and 93% specificity, with positive predictive value of 79%). Activin A is also increased in term newborns with moderate or severe asphyxia suggesting that activin is released after neuronal (Florio et al., 2004).

Population Pharmacokinetic Analysis. We will measure timed Epo concentrations to monitor for accumulation and safety, and to confirm Epo dosing. Plasma Epo concentrations will be measured using the Quantikine IVD™ human Epo Immunoassay ELISA (R&D Systems, Minneapolis, MN) at the UW Laboratory Core, under the direction of Dr. Juul. Epo measurements will be available for each DSMB review. A population pharmacokinetic analysis will be performed using sparse plasma Epo concentration data obtained at the following time points: baseline, drawn prior to the first study drug dose; peak and trough levels after the first and 4th doses, and a peak level after the second maintenance dose. NONMEM software (Icon Development Solutions, Ellicott City, MD) will be used to determine compartmental population pharmacokinetic parameters (i.e., clearance and volume). The base structural model will be implemented as established in the population pharmacokinetic model developed from the intensive pharmacokinetic data of our pilot study.[38] The random-effect model will be updated using the concentration data from this study. A covariate analysis will also be performed in a standard step-wise forward addition followed by backward elimination method to determine the influence of clinical factors (e.g. gestational age, birth weight, serum creatinine, concomitant medications) on pharmacokinetic parameters. Based on the final population model, post-hoc Bayesian individual pharmacokinetic parameters will then be estimated for each participant. Pharmacodynamic relationships will be determined between Epo pharmacokinetics and the clinical and biochemical

markers measured during the investigation.

Blood (0.5mL/sample, 2.0 mL total) will be obtained through an indwelling umbilical arterial or venous catheter when possible. Samples will be spun for 6 min at 2000 g; plasma and cellular components will be frozen in separate labeled tubes at -80°C. Samples will be labeled with a code provided by the DCC to maintain blinding. All samples will be sent to Dr. Juul's lab at the University of Washington. Each site will be sent premade packages containing the instructions, data sheets, sample labels, blood collection tubes and mailing labels with which to process the blood samples.

Neurodevelopmental Follow up: High-risk follow up for ELGANs is part of standard care at all sites. Routine visits generally occur at 4 months, 8 months, 12 months, 18 months and two years PMA. The timing of visits may vary depending on the child's need for intervention. If subjects are not scheduled at any one of these time points for routine high risk follow up, parents/guardians will be contacted by telephone at 4, 8, 12, and 18 months corrected age. The 30 minute phone contact will include an update of the subject's medical problems, medications, use of services such as physical therapy, and administration of the Warner Initial Developmental Evaluation of Adaptive and Functional Skills 50 instrument (Appendix 5).[6, 175-178]

Subject Retention. Additional contact to increase the likelihood of follow up participation will include sending birthday cards and appointment reminders. We will also use a social network internet site to enhance retention of subject families. Follow-up rates of $\geq$ 90% have been achieved in most of our study sites, using these methods. An offset for parents' travel/work loss expenses ($100) will be provided at the completion of the face-to-face follow up visit at 2 years corrected age. An additional $100 per visit is included in the budget to provide for unusually high travel expenses when needed. We estimate this will be needed for 15% of subjects.

The primary outcome will be neurodevelopment at 24-26 months corrected age. All personnel involved in the neurodevelopmental assessments will be blind to study treatment. Assessment at 24-26 months corrected age will include:

- Bayley III Scales of Infant and Toddler Development
- Standardized neurological examination based on ELGAN Neurological Exam Study protocol (Appendix 4)
- The Gross Motor Function Classification System (GMFCS)
- Modified Checklist for Autism in Toddlers (M-CHAT) (Appendix 8)

Bayley Scales of Infant Development (BSID) is the standard test used to evaluate early neurodevelopmental outcomes of preterm infants.[16, 22, 179, 180] The second edition Bayley Scales, used from 1969 to 2005, was modestly predictive of cognitive function at school age with MDI < 70 showing a positive predictive value of 0.19 – 0.8 for identification of children with an IQ < 70 at school age.[181-185] The cognitive portion of the Bayley test was revised in 2005[186, 187] (Bayley III Scales of Infant Development): the Mental Development index (MDI) reported in the Bayley II was a *composite* of cognitive, expressive and receptive language; in the revised Bayley III, individual scores for cognitive, expressive and receptive language are reported in addition to an assessment of social-emotional, and adaptive behavior. As experience has accumulated, it has become apparent that children score higher on the Bayley III compared to the Bayley II.[2, 6, 7, 187-191] Thus a cognitive score of 85 on the Bayley III likely corresponds to a Bayley II score of 70. Given that children assessed with the Bayley III are just now reaching school age, the value of the third edition of the Bayley Scales for prediction of cognitive impairment at school age and beyond is still under evaluation.[187] In order to ensure that the outcomes of this study are clinically important, we have identified two important stepped outcomes: the primary outcome is very stringent, and

uses a cut off of 2 standard deviations below the mean for cognitive or motor scales (<70). The secondary outcome uses a cut off of one standard deviation below the mean for these criteria (<85), which will still have a significant impact on the child, family, and healthcare system. The 2 year assessment will provide a window into early language development and early gross- and fine-motor development. We will submit further applications for long-term follow up of this cohort at 5 and 9 years of age to provide improved correlation with ultimate function.[192]

Inter-Rater Reliability Training:

Bayley Scales of Infant and Toddler Development, 3rd edition. Individuals performing the BSID-III assessments for this study will be required to be certified after attending a formal Training & Orientation Workshop 6 months before testing as organized by Drs. O'Shea and Lowe (run in tandem with a neurological training session). The certification process is comprised of two parts. *First*, each candidate Infant Assessor will review the test publisher's "Enhanced Administration DVD," which will be employed in this RCT as the "gold standard" for administration and scoring of the Bayley III. *Second*, the candidate Infant Assessor will administer the Bayley to healthy full-term infants who are 24-month-old. Video-recordings will be made of these administrations. The candidate Assessor will score each of his/her pilot administrations. A copy of the candidate's test protocol will be sent to the Study Psychologist (Jean Lowe, PhD) at the coordinating center, along with a copy of the video recording. The Study Psychologist will use these materials to assess the candidate's ability to (a) engage the infant in the Bayley activities, (b) administer and score each Bayley item, and (c) compile all summary scores. Infant Assessors will be required to meet the calibration standard for certification: 88% score concordance on items correctly administered. The Study Psychologist will provide prompt item-specific feedback, so that the candidate can make any needed modifications. Once Infant Assessors have begun to see enrolled patients, we will ask each Assessor to record one Bayley administration after the first 10 patients, or after the first 6 months, and then semi-annually. These video-recordings will also be reviewed by the Study Psychologist to guard against "calibration drift" over time. In addition, each month the Coordinating Center will run descriptive-statistics batch analyses as an additional quality-assurance device to safeguard against calibration drift. Training for Bayley assessment staff will include a semi-annual assessment to ensure continued reliable and valid assessment data. For Spanish speaking infants, we will use an interpreter who interprets each of the psychologist's requests to the child.

Neurological exam. To standardize the quality of data regarding neurological exams, examiners will attend a one-day workshop at 4 convenient hub locations in the U.S., view a training video, and then classify neurological findings illustrated on an assessment video.[193] Inter-observer variability assessments will be done to determine agreement with gold standard responses. Annotated feedback will be given to examiners regarding items that had a less than 85% correct rate, and, based on experience in the ELGAN study, we expect agreement rate to rise to over 90%.[47, 193] To minimize expectation bias, examiners will be blinded to the child's medical history and brain-imaging studies. Findings from the neurological exam will serve as the basis for an algorithm that classifies infants into one of four groups: no CP, diparetic CP, hemiparetic CP, and quadriparetic CP.[194] These categorizations correlate highly with long-term neurodevelopmental outcomes. We will use the neurologic exam forms created for the NINDS-funded ELGAN study (Appendix 4). In addition, CP severity will be determined using the Gross Motor Function Classification System (GMFCS) developed by Palisano.[4, 5, 195, 196] A GMFCS score of **2** or greater will be considered severe CP, given that these children will not be able to walk independently at age 2.

b) Standardized Diagnosis of Cerebral Palsy and Quantification of Motor Delay
The presence and sub-type of CP will be determined using the broadly accepted and standardized ELGAN Neurological Exam scoring system and CD based video teaching system.[193, 194] This software and training program developed by the PENUT collaborator **Dr. Karl Kuban** provides a distributable method of neurologic testing of subjects in a formalized, systemized method that is highly reproducible.[193] CP severity will be determined using the Gross Motor Function Classification System (GMFCS).[5, 195, 196]

Rationale for diagnosing sub-types of CP:
Although all forms of CP will be tabulated as part of the primary outcome, relationships between CP sub-types and cognitive development vary by the brain region affected and so we will classify CP into three sub-categories:

- Spastic Quadriparesis: has a higher association with structural injury and quadriparetic children are 9-times more likely than diparetic children to be severely impaired and 5-times more likely than diparetic to be microcephalic. They are also twice as likely to have MDI < 70 and a high rate of positive M-CHAT scores vs. diparetic and children without CP.

- Spastic Hemiparesis: represent an intermediate level of injury where 53% of this form of CP have Bayley < 70 vs. 75% for quadriparesis and only 34% for diparesis; consistent with a more common association with unilateral injury and focal lesions like middle cerebral artery stroke, that spare fibers closest to the ventricle in control of lower extremities.

- Spastic Diparesis: Diparesis is more commonly associated with symmetrical white matter injury close to the ventricle presumable due to injury of leg fibers of the pyramidal system that are closest to ventricles.

Gross Motor Function Classification System: The Gross Motor Function Classification System (GMFCS) system developed by Palisano, et al. focuses on children's functional achievements rather than on their limitations. It places emphasis on the child’s routine performance (not necessarily their best capacity) in the home or community setting.

The GMFCS system uses descriptions defining 5 levels of function that represent a broad ordinal scale where the distance between levels is not considered equal. Distinctions between levels are based on the need for assistive technology including mobility devices (such as walkers and wheeled mobility), and to a much lesser extent the quality of movements. Levels between 3 and 5 (the highest possible score) indicate progressively more serious limitations of gross motor function and that child is *severely* handicapped.[4]

Table 7. Summary of the Gross Motor Classification System for Cerebral Palsy. Adapted from Palisano et al., 1997.

Years	Level I	Level II	Level III	Level IV	Level V
0 ↓	- Able to sit up and balance while manipulating objects with both hands<br>- Crawls on hands and knees<br>- Begins to walk between 1.5-2yrs without assistance	- Maintains floor sitting but needs to use hands for support to maintain balance<br>- Creeps on stomach or crawls on hands and knees<br>- Pulls to stand & take steps while holding furniture	- Maintains floor sitting while low back is supported<br>- Rolls and creeps around on stomach	- Trunk support required for floor sitting<br>- Can roll to supine and may roll to prone positions	- Unable to maintain antigravity head and trunk postures while prone and sitting<br>- Requires assistance to roll
2 ↓ 4	- Able to move in and out of floor sitting positions and stand without assistance<br>- Walks as preferred method of mobility without assistive devices	- Maintains floor sitting but has difficulty with balance when both hands occupied<br>- Crawls, pulls to stand on stable surface & walks short distances with assistive devices	- Requires assistance to assume floor sitting, can maintain by "W sitting"<br>- Crawl on hands & knees, move using stable surfaces & walks short distances with assistive devices, adult assistance	- Floor sit when placed but unable to maintain balance & alignment without using hands for support<br>- Require mobility devices for sitting and standing, short-distance mobility achieved through rolling, creeping on stomach, crawling	- Voluntary control of movement and all areas of motor function restricted<br>- No means of independent mobility; require power wheelchair with extensive adaptations throughout life

The 2 year assessment will provide a window into early language development and early gross- and fine-motor development. **We plan to submit further grants for long-term follow up at 5 years of age, which correlates better with ultimate function.**[192]

Modified Checklist for Autism in Toddlers (M-CHAT). _Parent questionnaire_ (Appendix 9) will be administered to the mother/caretaker at the 24-26 month follow-up visit. This instrument is validated for screening toddlers between 16 and 30 months of age, to assess risk for autism spectrum disorders (ASD).

"The primary goal of the M-CHAT was to maximize sensitivity, meaning to detect as many cases of ASD as possible. Therefore, there is a high false positive rate, meaning that not all children who score at risk for ASD will be diagnosed with ASD. To address this, we have developed a structured follow-up interview for use in conjunction with the M-CHAT; it is available at www.firstsigns.org." Users should be aware that even with the follow-up questions, a significant number of the children who fail the M-CHAT will not be diagnosed with an ASD; however, these children are at risk for other developmental disorders or delays, and therefore, evaluation is warranted for any child who fails the screening.

The M-CHAT can be scored in less than two minutes. We will use the scoring template available at www.firstsigns.org. Children who fail more than 3 items total or 2 critical items (particularly if these scores remain elevated after the follow-up interview) will be referred for diagnostic evaluation by a specialist trained to evaluate ASD in very young children.

As the field of Neonatology has matured, more nuanced follow up of NICU graduates has become available. It is now clear that preterm babies have a higher risk than do term infants for psychological and behavioral problems, with autism spectrum disorders and attention deficit problems identified specifically.[197-201] We will therefore screen all subjects with the M-CHAT parental questionnaire at the 2 year follow up visit.[202] We anticipate positive screening will be higher in this ELGAN population than in term infants,[47] and if, as indicated in several MRI studies,[50, 201, 203-209] MRI imaging of brain structures correlates to psychological and behavioral function, we anticipate that Epo-treated neonates will have better preserved brain structure, and therefore, function.

5.3 Concomitant Interventions

Infants < 27-6/7 weeks of gestation require treatment for associated problems of prematurity. Interventions include, but are not limited to, mechanical ventilation, sedation, treatment of seizures, treatment of PDA either medically or surgically, treatment of sepsis (bacterial or fungal), transfusions with packed red blood cells or platelets, treatment of NEC, either medically or surgically, assessment and treatment for ROP. Data will be collected on associated conditions and medications used to treat them during the course of the subject enrollment.

5.3.1. Required Interventions: 1) Epo/vehicle treatment as designated by randomization; 2) head ultrasound prior to first study drug dose; 3) iron supplementation; 4) blood drawing 2.0 mL for Epo and biomarkers plus up to 1.2 mL for iron studies; 5) MRI at 36 weeks PMA depending on study site; neurodevelopmental follow up at 24-26 months corrected age at all study sites.

5.3.2. Use of any erythropoietic agents such as Epo or Darbepoietin off protocol is prohibited for subjects enrolled in this trial.

5.3.3. Precautionary Interventions. The attending physician or responsible family member can remove a subject from the study at any time. If a subject develops any SAE related to Epo treatment, Epo treatment will be discontinued.

Brain Imaging in Premature Infants. Please see Appendix 7.

The review titled:"Practice parameter: neuroimaging of the neonate: report of the Quality Standards Subcommittee of the American Academy of Neurology and the Practice Committee of the Child Neurology Society" published in 2002 gave recommendations for the timing and grading of ultrasound finings.[143]

Our study guidelines are modified from these recommendations as noted in the table below. Specifically, an extra head ultrasound will be done on day 1, and we will permit either CUS or MRI at 36 weeks PMA.

Table 8.

**Study Type**	**Timing of Brain Imaging**	**Rationale**
CUS	Day 1 (Prior to study drug administration)	Will identify early intracerebral and cerebellar hemorrhage and document CNS pathology present prior to study drug administration
CUS	Day 7-10	Will detect most cases of intracerebral and cerebellar hemorrhage and identify early hydrocephalus
CUS or MRI	$\geq$ 36-0/7 wks PMA or prior to discharge if this occurs earlier	Will detect all cases of severe intracerebral and cerebellar hemorrhage and can be used to assess periventricular white matter and ventricular size.

Iron Supplementation for ELGAN Infants. Please see Appendix 7.

5.3.2 Prohibited interventions.

No enrolled subject will be prescribed any Epo off study protocol.

5.3.2 Precautionary interventions.
None.

5.4 Adherence Assessment

All medication dosing will occur in the NICU and will be documented by the research study nurse and by the pharmacy. Accuracy and compliance will be in addition be assessed by plasma Epo concentrations drawn as shown in Figure 3.

In a subset of 220 subjects, a brain MRI will occur prior to discharge at 36 weeks PMA, and will be documented. The research study nurse will log phone contacts after discharge, and the follow-up assessments will be documented on study forms, which will be submitted electronically to REDCap.

All contact with families after discharge will be recorded. The family contact phone calls at 4, 8, 12 and 18 months corrected age will assess medical health and functional condition (WIDEA-FS) and remind parents of need for neurodevelopmental assessment. Assistance with scheduling the appointments will be offered.

Attendance at follow up clinic will be recorded.

Protocol Violation/Deviation Reporting.

Violations of the study drug administration protocol require completion of a Protocol Violation Form and notification to the CCC and DCC within 3 working d. Major Protocol Violations Include: Enrollment in light of exclusion criteria; Consent obtained not in accordance with IRB guidelines; Unblinding of study personnel; Study drug administration or dosing error.

6. CLINICAL AND LABORATORY EVALUATIONS

Data to be collected on each Subject.

Gestational age, birth weight, maternal history, placenta histology (intrauterine infection, insufficiency), Apgar scores at 1, 5 and 10 minutes, will be documented in addition to measures of maternal education, SES, and other factors known to affect cognitive development (e.g. drug/alcohol exposure). NINDS common language will be used when possible. A head ultrasound will be obtained prior to Study drug dosing.

6.1 Schedule of Evaluations

**Table 9. Evaluation**	Screening	Pre-Entry (Prior to 24 hrs)	Entry	1 day	7-10 days	12-15 days	28-32ndays	32 wks PMA	36 wks PMA	Discharge	4 months corrected age	8 months corrected age	12 months corrected age	18 months corrected age	24-26 months corrected age
Screen admissions to antepartum unit and NICU	X	X													
Documentation of Gestational Age		X													
Informed Consent		X													
Maternal Demographics and History			X							X					
Screening Head ultrasounds			X		X				X						
Blood Pressure			X	X	X	X		X							
Epo concentration			X	X	X	X		X							
Adherence Assessments			X		X	X		X							
Stored Plasma for biomarkers			X		X	X									
Ferritin or ZnPP/H						X	X	X							
MRI (subset of 220 subjects)									X						
Hematology (CBC/transfusions/phlebotomy loss)			X			X	X	X		X					
Liver/kidney Function Tests (as clinically available)						X	X	X							
Complete Physical Exam including weight, head circumference and length			X			X				X					X
Telephone contact to review current status											X	X	X	X	
WIDEA-FS phone questionnaire											X	X	X	X	
Neurodevelopmental assessment<br>Bayley III<br>Standardized Neurologic exam															X
M-CHAT parent questionnaire															X

6.2 Timing of Evaluations

Data collection for hematology, chemistry, liver and kidney function will be as clinically available. These tests are done routinely in all extremely preterm infants as part of usual care, and will not be required as part of the study protocol. The research nurse or her designee will collect these results from the medical record.

All head ultrasound and brain MRI results will be recorded. Analysis will be done using the most severe lesion noted.

6.2.1 Pre-Randomization Evaluations

These evaluations occur prior to the subject receiving any study interventions.

Screening

If appropriate, families will be approached for study entry while on the antepartum service, if delivery seems reasonably imminent. All NICU admissions will also be screened daily for eligible patients.

Pre-Entry

After birth, consent must be obtained by 24 hours of age to be eligible for study participation. The time window for initiation of study intervention from birth is 24 hours.

Entry

After consent is obtained, a cranial ultrasound must be obtained prior to the first study drug dose is administered. Results of the cranial ultrasound will not affect randomization or clinical care. These data will be used for later analysis of outcomes only.

6.2.2 On-Study/On-Intervention Evaluations

Blood draws are timed according to Study drug dosing (see Figure 3).
Physical exam will be done at study entry, after the first 6 doses of intravenous study drug, at 36 weeks PMA, and at discharge. A complete blood count (CBC) drawn as part of clinical care during the period between 34 to 36 weeks PMA will be recorded.

6.2.3. Intervention Discontinuation Evaluations

If a subject is removed from study treatment for any reason (parent decision, Attending physician choice, or because of SAE such as a symptomatic clot requiring anticoagulation), the reason for discontinuation will be recorded. Since this is an intention-to-treat study design, any subjects who discontinue intervention will continue to be followed and evaluated on study. Like all study subjects, these families will have a discharge questionnaire filled out, and will be contacted by phone at 4, 8, 12 and 18 months. They will be sent birthday cards for the baby and mother. A small monetary incentive will be given to each family at the completion of the 2 year follow up visit. For those individuals that must travel over 2.5 hours for the follow up visit, overnight accommodations may be offered.

6.2.4 On Study/Off-Intervention Evaluations

The MRI should be done between 36-0/7 and 36-6/7 weeks of PMA. If the patient is ready for discharge in the week prior, the MRI should be done at this time. This will be done in a subset of patients at 4 of the study sites (Wake Forest, University of Florida, University of MN Amplaz and Methodist Children's.)

6.2.5 Final On-Study Evaluations

Study drug treatment will end at 32-6/7 weeks PMA, and each subject will be followed for 2 years,

until their neurodevelopmental assessment at 24-26 months of corrected age.

At the 24-26 month follow-up visits, documentation of the standardized neurologic exam, and results of the Bayley III exam will be recorded.

DOCUMENTATION AND RECORD RETENTION

Documentation. Each site must provide the CCC project director or designate with the following documents prior to study initiation. A copy of these documents must be maintained in the investigator's study files.

- IRB approved informed consent form - All IRB approvals and correspondence (including approved revisions, protocol, advertisements, etc.) - Copies of all correspondence pertaining to the study (excluding any budgetary matters) - Copies of all serious adverse events submitted to the IRB - Copy of all safety reports.

The UW DCC co-supports an installation of REDCap, software specifically designed for electronic data capture that we have used successfully in other multi-site studies. REDCap features include differentiated user roles and privileges, password and user authentication security, electronic signatures, SSL encryption, and comprehensive auditing to record and monitor access and data changes (http://www.project-redcap.org/software.php). REDCap will serve as the architectural backbone for all data captured prospectively, with all data linked by study subject ID.

Record Retention. The clinical site is responsible for maintaining all records (i.e., case report forms, original data, screening logs, signed informed consent forms, correspondence, etc.) until notified, in writing, by CCC, that these records are no longer needed. The Investigator must notify CCC project director if the site or records are relocated, if the investigator leaves the institution, etc. and a new address for the records must be provided.

6.2.6 Off-Study Requirements

When the subject has completed the 2 year follow up visit, there are no further requirements for study participation. Participation in any further follow up will require new consent.

6.3 Special Instructions and Definitions of Evaluations

- Screen admissions to antepartum and NICU: Screening for eligible subjects will be done daily. For antepartum admissions, this will involve determining whether any admissions within the past 24 hours are likely to deliver imminently, and if so, whether they are within the gestational age criteria for the study. If both these criteria are satisfied, the Attending Perinatologist will be asked whether this patient might be an appropriate study candidate, and if so, whether it is permissible to approach the mother to discuss the study.

  For neonatal admissions to the NICU, screening will involve determining the infant's time of birth and gestational age. If they qualify for the study, the Attending Neonatologist will be asked whether they meet inclusion (but not exclusion) criteria. If the baby meets criteria, the Attending will be asked whether it is permissible to approach the family to discuss the study. Once it is confirmed that the family is willing to hear about the study, they will be approached for informed consent.

  A screening log will be completed for all screened patients (Appendix 6).

6.3.1 Informed Consent

Antenatal consent will be obtained when possible. Prenatally, the study investigator will obtain permission from the Maternal Fetal Medicine Attending to approach the mother to discuss the study. Postnatally, permission to approach the family will be obtained from the Attending Neonatologist. The Attending physician will seek parental agreement for an investigator to meet and discuss the study. If the parents are interested, the study investigator will discuss the study with family and seek consent in person. The consenting legal guardian will receive a copy of the consent form to review, and once signed, will be given a copy to keep. If the Attending physician is also a study investigator, an alternate study investigator will obtain consent, so as to avoid coercion. Investigators will only approach family after infant's Attending physician gives permission and family indicates that they are interested in further information about the study. No alteration in care will otherwise occur. Attending neonatologist and family can withdraw child from study at any time.

Consent will be obtained by the Investigator in a private room which ensures the privacy of the family, and which is free of potential coercive influences. Consent for participation must be obtained by the time the baby is 24 hours of age.

If a family has limited or no English speaking abilities, a certified interpreter will be provided. They will review the consent form with the family, and interpret the verbal explanation of the study during the discussion between the Investigator and the family members. If individual sites have a large population of non-English speakers, consent forms will be translated into the appropriate languages. If an interpreter is not available in a timely manner, the family will not be approached.

The parents of the research participants will be given opportunity to review the study both verbally and in writing. The will be given opportunity to ask questions of the investigator prior to giving consent.

If there are changes in the protocol or safety information that require consent forms to be updated, they will be sent through the IRB process for approval. The study entry form has a space for documentation of a signed consent form as well as a signed HIPAA form. These forms will be maintained in the REDCap electronic database.

A model informed consent form is included as Appendix 10.

6.3.2 **Documentation of Gestational Age** will be done according to the following hierarchy, and the method by which gestational age was determined will be logged on the enrollment form:

1. Gestational age by in vitro fertilization if available.
2. Gestational age by first trimester assessment (0-14-0/7 weeks)
3. Gestational age by second trimester assessment (up to 28-0/7 weeks)
4. Last menstrual period (LMP)
5. Newborn maturational assessment

Documentation of intraventricular hemorrhage. Screening Head Ultrasounds will be obtained as per the guidelines in Section 5.1 page 22. Only the first ultrasound is done for study purposes. The subsequent two ultrasounds (or MRI at 36 weeks) will be done as part of routine clinical care. All head ultrasound results will be recorded. The Guidelines delineate how pathology will be documented and graded.
Documentation of clinical findings will be done in REDCap after chart extraction.

Documentation of neurodevelopmental follow up will be at the time of the 24-26 month follow up

visit. A standardized neurologic exam and Bayley III exam will be done. The M-CHAT questionnaire will also be administered at this time.

6.3.3 **Maternal demographics and history** will be obtained and documented on the demographics and maternal history forms after mother consents to participation in the study (Appendix 6).

6.3.4. **Treatment history.** Not applicable since subjects are newborns

6.3.5. **Concomitant treatments** while the subject is in the NICU will be documented.

6.3.6. **Study Intervention Modifications.** Confirmed neutropenia (ANC <500/µL), central hematocrit (Hct)>60%, severe sepsis (defined as culture-proven sepsis requiring pressor or new ventilatory support), unexplained seizure, symptomatic clot, or hypertension (blood pressure 2 SD > mean for age.[134, 136] Study drug will be restarted when neutrophil counts increase over 500/µL, Hct <45. In the case of sepsis, study drug will be interrupted for 3 days and then restarted. Parenteral iron will not administered while study drug is held. The rationale for withholding iron during serious infection is that iron has been reported to be a growth factor for selected bacteria and might worsen the patient's condition during sepsis.[137]

6.3.7. Clinical Assessments.

Clinical safety parameters

1) **Complete physical exam** will be done on study entry, after the first 6 doses of study drug (day 11-13), at the time of MRI (36 weeks PMA), and at discharge. Growth parameters (head circumference, height and weight) will be obtained at these times. The presence of microcephaly (OFC<10th percentile) or relative microcephaly (discrepancy of > 50 percentile between weight, length and head circumference) will be recorded.

2) **Vital signs and blood pressure**. All ELGANs are monitored continuously for heart rate and arterial saturation (pulse oximetry), with either continuous or intermittent blood pressure readings as part of routine care in the NICU. The high and low blood pressure will be recorded for the first 14 days of the study, and at timed intervals thereafter (weekly, then at 32, 36 weeks PMA and at discharge). If blood pressure is 2 standard deviations above the norm for gestational age for 3 measurements 2 hours apart, study drug dosing will be held until the blood pressure normalizes. Any long-term effects of Epo on blood pressure will be assessed at 32-6/7 weeks and at discharge.

3) **Hematologic data**. Complete blood count (CBC) including hematocrit, white blood cell count, absolute neutrophil count, platelet counts and blood smear are obtained routinely in extremely preterm neonates as part of their care. This is done to evaluate for infection, and to follow the need for transfusion. Results of these tests will be recorded weekly, as available, through 36-6/7 weeks PMA. The number and volume of blood transfusions, donor exposures, and phlebotomized blood volume will be recorded for the entire hospital stay. Ferritin or ZnPP/H will be followed to monitor iron status. While important for all preterm newborns, this is strongly recommended for those minority of infants receiving parenteral iron. Iron supplementation will be modified based on these results. (See iron guidelines)

4) **Organ function** (BUN and Creatinine), and **liver enzymes** (bilirubin T/D, AST, ALT, alkaline phosphatase) will be recorded weekly as available, through 36-6/7 weeks PMA. These data are checked routinely on all infants requiring parenteral nutrition support, and as part of routine

assessment of nutritional status.

5) **Respiratory data.** Maximum and minimum daily respiratory support will be recorded for the first two weeks of life, then support documented at 28 days and 36 weeks PMA.

6) **Complications of extreme prematurity** defined as follows will be recorded:

- Bronchopulmonary dysplasia (BPD). BPD will be defined at 36 ± 1 week corrected gestational age. Infants requiring ventilation or CPAP are defined as having BPD. Infants receiving continuous oxygen < 30% will undergo an oxygen reduction challenge in accordance with guidelines developed by the NICHD Network.[210] Infants failing to wean from supplemental oxygen (oxygen saturations of < 88% in room air) fail the challenge, and are defined as having BPD.
- Sepsis will be defined as culture proven bacteremia.
- Necrotizing Enterocolitis (NEC). Bell's staging criteria will be used to define NEC. All surgeries for NEC, and for strictures or bowel obstructions occurring as sequelae of NEC, will be recorded.
- Retinopathy of prematurity (ROP). All infants will be followed using the screening recommendations published in 2006.[211] The international classification of ROP will be used.[212]
- Intracranial Hemorrhage (ICH), white matter injury (WMI) or hydrocephalus (HC). A head ultrasound will be done prior to the first study drug dose as part of the study. Brain imaging will also be done as part of routine care at 7-10 days and 36 weeks PMA. The presence, location and extent of any intracranial bleeding, hydrocephalus or periventricular echolucencies/densities will be documented.
- Clinical seizures. If clinical seizures are suspected, an EEG will be done to confirm the diagnosis.
- Hypertension. Blood pressure is monitored at least daily as part of standard care. If the blood pressure is recorded as two standard deviations above the mean for PMA, on 2 sequential days, hypertension will be diagnosed.
- Patent ductus arteriosus that is treated with either medical or surgical intervention. The indications for treatment will be recorded (ECHO parameters and/or clinical symptoms). Age at treatment will be recorded.
- Sepsis, confirmed by blood culture, or presumed (clinical symptoms present and antibiotic treatment instituted for 7-10 days despite negative cultures).
- Mortality. The timing and circumstances of any deaths in this study population will be recorded and reviewed by the DSMB.
- Data on medication administration, including use of steroids, caffeine, antibiotics and indomethacin will be collected.

7) **Blood samples.** Blood (0.5 mL/sample, 2.0 mL maximum) will be obtained through an indwelling umbilical arterial or venous catheter when possible. If no access is available, all efforts will be made to combine the blood draw with clinically indicated phlebotomy times. The timing of blood draws is shown in Figure 3.

The CCC will provide sample collection packs to each site. These will contain biohazard bags,

purple top tubes for blood collection, a microfuge tube for plasma collection, and labels for subject identification and date. Samples will be spun, and plasma frozen at -80°C. Batched samples from each site will be sent to Dr. Juul's lab at the University of Washington.

Biomarkers. Plasma from these samples will be used to investigate the effect of Epo on inflammatory mediators and growth factors, as well as Epo pharmacokinetics.

Epo Pharmacokinetics. See section 5.2, Figure 3.

Sample storage. Samples will be spun on site to separate plasma from cells. Plasma and cells will be stored in separate, labeled containers at -70ºF. They will be sent to the UW in batched quantities, after every 5 subjects. The stored cells for each subject will be kept for later genome wide association studies (GWAS) of problems related to prematurity that are beyond the scope of this study.

8) MRI will be done at selected sites at 36-0/7 to 36-6/7 weeks PMA

9) Neurodevelopmental assessment will be completed on each subject at 24-26 months corrected age:

- Bayley III Scales of Infant Development: Composite Language and Composite Motor Scale in 5 domains - cognitive language (receptive, expressive), motor (fine and gross), social-emotional, and adaptive.[186]
- Modified Checklist for Autism in Toddlers (M-CHAT) (Appendix 9)
- Standardized neurological examination (Appendix 4)
- The Gross Motor Function Classification System (GMFCS)

Please see the accompanying draft CRFs that will be used to document these facts.

6.3.9. Epo concentrations will be obtained at multiple time points as previously described, and as outlined in Figure 3. Plasma for inflammatory mediators and biomarkers of brain injury will be obtained at the same blood draw on days 1, 7 and 15. Population Pharmacokinetic Analysis will be done, and Epo concentrations will also be used to document study adherence for subjects assigned to the Epo (and control) arms.

- Adherence Assessments will include documentation by pharmacy that they dispensed study drug, and documentation by the research nurse that study drug was administered. Further confirmation will be obtained by Epo blood levels.

7.0 MANAGEMENT OF ADVERSE EXPERIENCES

All complications will be treated in the site NICUs.

8.0 CRITERIA FOR INTERVENTION DISCONTINUATION

The attending neonatologist or parent may withdraw the infant study subject at any time for any reason. The reason for such withdrawal will be recorded. The study interventions will be discontinued in any infant subject who suffers a SAE due to Epo administration. The research coordinator and study investigator will evaluate all subjects on an ongoing basis for evidence of Epo SAE occurrence (thrombosis, hypertension, polycythemia, stroke). See section 7 for further delineation of SAE issues.

9.0 STATISTICAL CONSIDERATIONS

9.1 General Design Issues

Hypothesis: Epo treatment from 24 hours to 32-6/7 weeks postmenstrual age (PMA) of **E**xtremely **L**ow **G**estational **A**ge **N**eonates (ELGANs) will safely decrease the combined outcome of death or neurologic impairment from 40% to 30% measured at two years of age.

Design: *We will enroll 940/2 = 470 subjects in each of two treatment groups: Epo-treated (from 24 hours of age to 32-6/7 weeks PMA)* vs. *placebo control.*

Primary outcome variable: The primary outcome is the composite outcome of death or neurodevelopmental impairment at 24-26 months. Neurodevelopmental impairment (NDI) is defined as the presence of any one of the following: CP, Bayley III cognitive or motor scale < 70. There is a known inflation of scores from the Bayley II to III[2, 6, 7] and we will therefore also consider a threshold of <85 for secondary analysis. Subjects will be stratified by gestational age (24-0/7 to 25-6/7 and 26-0/7 to 27-6/7) and by study site.

Primary Analysis: The primary analysis will be a test of equality of the rate of the primary outcome (death or NDI) across the two randomized investigational groups. Specifically, we will use a GEE Wald test based on logistic regression, with stratification by recruitment center and gestational age. We will perform intent to treat analysis and expect minimal non-compliance due to the nature of the intervention in relation to in-patient care. For the primary endpoint we expect uniform and complete ascertainment of death but may not evaluate all subjects for developmental impairment. We plan to perform a primary analysis based on complete cases and will exclude those subjects for whom vital status is known (alive) but NDI cannot be assessed. Sensitivity analysis will use multiple imputation to evaluate the potential impact of any missing data. Secondary analysis will be for quantitative measures of brain volume, and for these endpoints an unadjusted t-test provides inference regarding the mean response across the treatment groups. We will adjust all secondary outcome analyses for recruitment site using regression methods.

Power and Sample Size for Primary Outcome: In order to determine the necessary sample size for efficacy evaluation we need to formulate assumptions for the primary outcome rate in the treated and untreated groups.

The primary outcome measure is the rate of death or severe neurodevelopmental impairment (NDI). Using data from two sources we can compute expected the expected rates of death or NDI for the neonates that we will enroll. The Vermont Oxford Network 2008 Follow-up Report[213] evaluated the disability status of infants born in 2008 only, and the combined 2004-2008 cohorts.

Follow-up status was determined at age 18-24 months and information regarding death and NDI is provided for subgroups of children based on their gestational age. Therefore, we can use these data to forecast expected trial results for our eligible subjects (24-27 weeks gestational age).

The VON reports on severe NDI and specifically states that components of severe disability include: cerebral palsy, or a Bayley score less than 70 or too severely delayed for Bayley testing.[213]

In addition, data from Gargus et al. (2009)[22] provides follow-up information at 18-22 months for approximately 3,800 neonates born between 24 and 27 weeks gestational age, based on babies born between 1998 and 2001.

We have combined the VON and Gargus (2009) data in order to determine the anticipated characteristics of our proposed trial. While the VON data is contemporary, it has relatively low follow-up for two-year outcomes (approximately 50%) and therefore may be biased toward more easily followed or more favorable subjects. Table 13 displays our best estimates of gestational age specific, and overall rates of death and NDI.

Table 13: Combined Vermont Oxford Network (VON) and Gargus (2009) data showing death rates and severe neurodevelopmental impairment (NDI) rates for different gestational ages (GA).

Gestational age (weeks)	Fraction of enrolled	Death	NDI	Total (Death+NDI)	Expected Treated (Death + NDI*0.45)
24	0.25	32	25.0	54.4	44.8
25	0.25	19	24.2	40.1	32.3
26	0.25	13	21.2	30.4	24.7
27	0.25	9	18.1	24.5	19.0

Our assumed death rates of 32, 19, 12, and 9 percent are exactly those presented in the VON 2008 report, and are lower than the rates reported in Gargus (2009) where among neonates born between 1998 and 2001 GA-specific death rates of 45, 25, 18, and 15 percent were observed. Using our combined data assumptions we expect an overall death rate of approximately 18%, which is approximately the rate observed in VON (17%), and is lower than the 26% death rate observed for Gargus (2009).

In addition, Gargus (2009) report GA-specific rates of NDI as 21, 25, 23, and 19 percent. The VON 2008 report estimates NDI rates as 22, 21, 17, and 16 percent respectively. We have combined VON and Gargus (2009) data and further assumed that NDI rates are decreasing with increasing gestational age to obtain our NDI estimates. Using these data we expect an overall NDI rate of 22%, which equals the overall rate observed in the VON 2008 report and in Gargus (2009).

In order to estimate the overall rate observed among treated neonates we have assumed that there will be no effect of treatment on death, but that Epo will lead to a decrease in the rate of NDI. If we assume a multiplicative reduction in the NDI rate of 0.45 then we expect a treated NDI rate of 12 percent and an overall rate of death+NDI of 30.4% as compared to the control rate of

40.4%. *Therefore, in order to obtain a target sample size we assume: an overall control rate of 40%, and an overall treated rate of 30% corresponding to an overall treatment rate ratio of 0.75.*

Using the control and treated rates of 40% and 30% respectively leads to a sample size of 376 evaluated subjects per arm or a total evaluated sample size of 752 subjects.

Efficacy Trial Statistical Analysis Plan – Secondary Outcomes

Secondary Aims and Outcomes:

1. To compare safety measures between infants receiving Epo and placebo to determine whether there are risks associated with Epo administration.
2. To compare moderate/severe impairment (< 85 on Bayley III cognitive or motor score, or CP).
3. To compare neuroimaging outcomes across the two treatment groups.
4. To assess whether treatment effects vary by gender.
5. To evaluate whether individual biomarkers measured through 36 weeks are predictive of 2-year outcomes, and whether a derived multivariate combination of markers has predictive performance greater than individual marker performance.

Analysis for Secondary Aims and Outcomes:

Safety: The primary safety outcomes are the serious adverse events and adverse events listed above in section 2.c.3.2. Analysis will compare the proportion of subjects with an AE across the two treatment groups and will use a 2-sample test of proportions. Inference for the (5) individual SAE outcomes will use a Bonferroni correction for multiple comparisons.

Power: The expected prevalence of SAEs range from less than 1% for polycythemia, to 18% for death. In order to characterize power we show the effect sizes for which we have 80% power based on having n=376 subjects evaluated in each group:

Table 13.

Baseline (control) rate	Alternative rate for 80% power<br>Using alpha=0.05	Using alpha=0.01
5%	11% (RR = 2.2)	13% (RR = 2.6)
10%	18% (RR = 1.8)	19% (RR = 1.9)
15%	24% (RR = 1.6	25% (RR = 1.7)
20%	30% (RR = 1.5)	31% (RR = 1.5)

Secondary Efficacy Outcome: Our key secondary long-term outcome is the rate of death or NDI using severe or moderate impairment defined by a Bayley less than 85. In order to estimate power for the secondary outcome we assume the same overall rates for death and severe NDI as above, and then assume a 20% rate for moderate impairment. Similar to the primary outcome we assume that the death rate is unchanged by Epo and that the rate of severe NDI is reduced from 22% to 12%. We also assume that the moderate NDI group would have an effect similar to the severe group where the 20% rate would be reduced by a factor of 0.45 to 11% but that among the 10% of severe subjects impacted by Epo half of these would only move from severe to moderate impairment. Therefore we assume:

	Control	Treated
Death	18%	18%
Severe NDI	22%	12%
Moderate NDI	20%	16% = 11% remain mod + 5% from severe
**Total**	60%	46%

Power: Using these assumptions and the target evaluated of n=752 *leads to greater than 95% power to detect a difference on the secondary outcome*.

Imaging Outcomes: The neuroimaging outcomes are: Myelinated white matter volume; Total gray matter volume; and White matter integrity (TBSS FA corpus callosum). We will conduct a single MANOVA test using the multivariate outcome and comparing treated and control subjects. The mean and standard deviation will also be calculated (by treatment group) for each individual measure. Note that MR measures will be obtained on a subset of infants (110 per treatment group).

Given the a priori hypothesis that treatment effect may differ according to gender we will conduct a single subgroup analysis that assesses treatment effects separately for males and for females. Subgroup specific treatment effects will be computed and inference will be based on a single Gender-by-Treatment test for interaction using logistic regression.

*Power***:** Multiple MRI variables will be collected and analyzed in this study. To provide a basic sample size for a treatment effect we selected one common basic measure of structural injury that has been identified in the literature for the premature neonates that we hypothesize will be preserved: The size of the cerebellum tissue when compared to the whole head volume. We used a comparable set of T1 weighted brain scans of 22 premature babies aged between 34 and 36 PMA, with a representative range of brain injuries present on MRI and applied the proposed segmentation methods to the data. We estimated the ratio of the cerebellum to whole brain size as our marker across this group and used the mean and variance of these measures on which to base our power calculation for a treatment effect. Assuming age matched groups in the treated and untreated groups, from this data we estimate that 110 subjects will be required to see 5% increase in the cerebellum size. The recognition of cerebellar atrophy in extremely preterm infants has only recently come to light with the increased use of MRI.[201, 214-219] Cerebellar atrophy has been linked to cognitive as well as motor deficits.[216, 220, 221] In summary, we will focus on four primary measures that are established to be predictive of 2-year neurodevelopmental status and therefore can be used to establish support for the hypothesis of long-term benefit of Epo treatment. The neuroimaging biomarkers are: 1) myelinated white matter volume; 2) total gray matter volume; 3) cerebellar volume and 4) white matter integrity assessed by TBSS (using FA corpus callosum).

Biomarker Analysis: We will consider two main classes of potential predictors of 2-year status: neuroimaging measures; and inflammatory markers. Interest is in the prognostic potential of individual and/or combined biomarker measurements. Given that the primary outcome is a binary measure (NDI) we will evaluate the predictive potential of individual quantitative measures using ROC curves showing the full potential of sensitivity and specificity across marker cut points. We will compute ROC curves for the (4) primary neuroimaging measures, and separately for individual inflammatory markers. Only 220 subjects will have data on the inflammatory markers, and these will be the same subjects identified for MR measures. We will derive two multivariate predictive models: using the inflammatory markers; and using the MR measures. We will use AIC

and 10-fold cross-validation to develop and validate predictive models. A final multivariate model will combine markers from both MR and inflammatory measures, and 10-fold cross-validation will permit inference in the incremental value of adding markers in combination by comparing ROC curves and associated area under the ROC curve (AUC).

9.3 Meeting Recruitment Targets. We have chosen sites for this trial that can be expected to enroll a minimum of 30 infants/year in the study (most expect to enroll more than 35/year). We plan to recruit at 16 sites, several of which have participated in the NO CLD and TOLSURF studies. Based on our experience to date with these studies, we anticipate that the participating sites will meet their enrollment targets and that subject accrual will follow the projected timeline. We plan 4-6 months after receiving a promising score for finalizing preparations to begin enrollment in the study. We have provided very conservative estimates of enrollment, recognizing that some sites will do better, and some worse than predicted. Accordingly, we will assess site enrollment every 6 months after initiation of the study. Should enrollment at an individual site fall below 6 in 6 month period, the PI and Clinical Steering Committee will give warning and evaluate whether it is appropriate to drop the site from the trial. If no patients are entered during a 6 month period the executive committee will recommend that the site be dropped and a new site recruited. If a single site appears to be enrolling more than 20% of the total infants enrolled in the trial we will temporarily limit enrollment at that site.

In order to obtain the target number of evaluated subjects we propose recruitment from 16 centers with a base population of approximately 1900 infants over the planned recruitment timeframe. With a base population of 1900 eligible infants, we expect:

Enrollment and Follow-up Assumptions:
- Recruitment base of n=1900 over 2 years
- 10% ineligible
- 20% refusal rate
- This would yield 1368 enrolled subjects
- Assuming 2% refusal post-enrollment
- Assuming a 19% death rate (note 18.2% assumed above)
- Assuming a 10% lost to follow-up rate
- This would yield a total of 1230 evaluated subjects

Therefore, our anticipated enrollment capability exceeds the necessary target by approximately 60% (e.g. 1230/752 = 1.63). Using these same assumptions we would need to enroll 836 subjects to obtain 752 evaluated babies. In order to be conservative we allow for a potentially greater loss to follow-up rate of 15% (versus 10%), and potential variance inflation due to clustering of twin outcomes, and therefore will target enrollment of n=940 subjects (20% greater than the needed n=752). The Vermont Oxford Network reports that 25% of ELBW infants are from multiple births and a variance inflation factor would therefore involve the expected cluster size (1.25) and the within-cluster correlation (ρ) and equal: $1 + (1.25-1)\,\rho$. If the within sib-ship correlation is 0.50 then the variance inflation factor (VIF) would be 1.125 implying a 12.5% increase in target sample size necessary to retain appropriate power.

In both the phase I/II Epo trial of extremely low birth weight infants and the NEAT trial (Epo neuroprotection of term infants with hypoxic ischemic encephalopathy), consent was obtained in over 90% of approached parents. This extremely high consent rate reflects the concern parents have about their children's neurodevelopment, and their willingness to participate in research that might result in improved outcome.

Site performance will be tracked with both enrollment and follow-up tables. Examples are shown below.

EXAMPLE ENROLLMENT AND FOLLOW-UP TABLES

Shell Table 1. *Open Report:* Cumulative and Current Report Period Study Recruitment.

	**Current Report Period**			**Cumulative**		
**Site**	**Screened**	**Eligible**	**Enrolled**	**Screened**	**Eligible**	**Enrolled**
Site-Name-1						
…						
Site-Name-16						
**Total**						

Shell Table 2. *Open Report:* Follow-up rates by site, n / N (%).

**Site**	**4 month**	**8 month**	**12 month**	**18 month**	**24 month**
Site-Name-1					
…					
Site-Name-16					
Total					

9.4 DATA MONITORING

Per NIH Guidelines (1998), this Data and Safety Monitoring Plan (DSMP) defines the oversight and monitoring activities that will ensure and maintain both the safety of participants and the scientific integrity and validity of the trial data. The plan also describes the procedures for adverse event reporting and detailed guidelines for recommendations related to trial continuation.

PENUT is a multi-center randomized placebo-controlled phase III trial to determine whether recombinant human erythropoietin (Epo) will safely improve the long-term neurodevelopmental outcome of preterm infants ≤ 27-6/7 weeks of gestation. The PENUT study will randomize n=940 subjects to receive either Epo or a placebo.

9.4.1. Data and Safety Monitoring Board (DSMB)

Review of the PENUT trial's study performance and safety outcomes will be conducted by the Data and Safety Monitoring Board (DSMB) as required by the NIH for multi-site clinical investigations. The DSMB will consist of 5 members with a designated chair, and safety officer. Committee membership should include expertise in both biostatistics and bioethics, and should have clinical expertise appropriate for the intervention and target population. The DSMB is expected to meet two times (every 6 months) per year to review study performance and safety outcomes in Open and Closed Reports, and to review study enrollment reports quarterly. Ad hoc sessions may be scheduled as required should a serious adverse event need to be reviewed by the group.

Aggregate safety and efficacy data and study performance monitoring data will be presented during the open sessions of DSMB meetings. Blinded safety and efficacy data will be presented by treatment arm during the closed sessions. Review by the DSMB provides assurances that the trial can continue without jeopardizing patient safety. The DSMB is also responsible for protecting the confidentiality of the trial data and for monitoring the quality of both the data and study implementation procedures.

The DCC will work closely with the CCC in developing and implementing a comprehensive system for safety monitoring. Safety monitoring includes the systematic review of safety data for trends that may impact patient safety. The processing, reviewing, and reporting of adverse experiences (AEs) and serious AEs are also part of this process. The infants in this study are hospitalized at study entry, and the duration of their hospitalization is highly variable and not predictable at birth. It ranges from as short as three months to more than a year.

Data and Safety Monitoring Schedule: Our target enrollment is n=940 which is expected to accrue during the first 2.5 years of the trial. Therefore, we will enroll approximately 250 subjects every 6 months. Our planned DSMB safety analyses will occur every 6 months after trial initiation. At the first safety evaluation we expect to have 250 subjects enrolled, but would only have 3-month outcomes on the first 125 subjects. Each subsequent 6-month period would increase the number of babies by 250 leading to the following cumulative number of subjects available for analysis:

9.4.2. Monitoring Guidelines

Based on findings following review of study data by the DSMB, the Board may recommend: continuation of the trial, termination of the trial, or modifications to the protocol (e.g. adding new measurements for safety monitoring, discontinuing high risk subjects, extending the trial in time, increasing the trial sample). Decision guidelines in the PENUT trial are based on group differences in adverse event rates as explained below, and on whether SAE rates exceed pre-determined thresholds.

9.4.2.4.1 Performance Monitoring

Performance monitoring will be an ongoing activity performed by the study principal investigator and statistician, and status reports will be reviewed by the DSMB during their regular meetings. Procedural reviews to address protocol compliance with respect to subject recruitment and eligibility, retention and follow-up, randomization and blinding, and quality of data will be conducted and monthly reports generated. Any protocol violation that affects patient safety will be reported to the DSMB immediately.

Performance data will be reviewed in aggregate and by site. It is expected that:

(1) The <u>response rate</u> for determination of the primary outcome (e.g. 24 month Bayley) will be no less than 80%;
(2) <u>Missing interviews</u> will be no greater than 15% at the 4, 8, 12 and 24 month follow-ups;
(3) The overall <u>enrollment rate</u> will not drop below the expected rate (40 subjects per month for 24 months) by more than 25%.

Data will be entered centrally into a clinical trial management system utilizing a REDCap database hosted at the DCC. Data will be entered into fields with automated validation and logical check built in. Data will be double entered on the outcome measures of Bayley III and CP assessment by standardized neurologic exam. Compliance will be assessed based on the weekly conference calls and data submitted to the DCC on a weekly basis. If it is determined by the study PI that either 1) study protocol is not being followed, or 2) that reporting is inadequate at any site, further action will be taken to address these issues. These actions may include additional in-person site visits if appropriate or additional educational/problem-solving sessions by phone or in person regarding the study protocol.

9.4.2.2 Safety Monitoring:

The research coordinator at each site will monitor each subject weekly for the presence of any complications. Serious adverse events will be brought to the attention of the IRB and the DSMB in writing. An independent medical monitor will review all cases of serious adverse events. A potential risk that is unique to preterm infants is the risk of ROP.[122] In the published studies of preterm neonates receiving potentially neuroprotective doses of Epo, no difference has been noted between treatment and control groups.[36, 38, 118, 119]

As part of the Data and Safety Monitoring Plan (DSMP) we will perform continuous and interim analysis of accruing safety data. We have defined potentially treatment (Epo) related serious adverse events (SAEs) that will be monitored throughout the course of the study. Specifically, for SAEs we will compare absolute rates to expected rates based on published data for similar newborns, and will seek careful DSMB review and guidance when observed rates exceed pre-specified thresholds. In addition, at planned interim analysis we will formally compare the event

rates across the two treatment groups using appropriate small sample methods such as Fisher's exact test.

9.4.2.3. Data and Safety Monitoring Schedule.
See section *9.4.3.1*, Table 12.

9.4.2.4. Treatment Efficacy Monitoring

Treatment efficacy will be monitored by the DSMB but recognizing that 24 month efficacy outcomes will not be obtained until after enrollment is complete (or nearly complete). Therefore, although formal interim analyses will be conducted using group-sequential boundaries there may not be compelling reasons to terminate the trial on the basis of accruing efficacy data. Efficacy measures will be provided in a blinded fashion to the DSMB with treatment groups labeled "A" and "B" as well as a z-score assessment of the evidence for the difference between treatment arms at 24 months. An O'Brien-Fleming interim stopping boundary will be provided to the DSMB to help assess the magnitude of the z-score.

9.4.3. Scheduled Reporting

One month prior to each DSMB review, the Data Coordinating Center (DCC) will summarize monthly administrative reports that describe study progress including subject accrual by site, demographics, and the sites' adherence to inclusion/exclusion criteria and other protocol requirements, including retention rates at each follow up point. These reports are prepared monthly and reviewed internally by the study research team for ongoing quality control and are also presented to the DSMB and NIH as requested. The DCC will also produce safety reports that list adverse events, serious adverse events, deaths, and disease or treatment specific events by site and in aggregate to the DSMB.

With each review the DSMB will approve the study and protocol as is, recommend protocol changes in the interest of patient safety or stop the study based on overwhelming evidence of treatment benefit or safety concerns. The DSMB will provide the recommendation in written minutes provided to the NIH. If the NIH concurs with these recommendations, they will be forwarded to the principal investigator. The recommendations will include any suggested changes in the proposed timing of future DSMB reviews. The review may result in an amendment to the protocol, which must be approved by the IRB, the NIH and the sponsor.

The DSMB report will begin with a brief narrative section that describes the status of the study, progress or findings to-date, issues, and the procedures that produced the report (e.g., data obtained by a specific date). The report will provide a study description along with a current organization chart, current timetable and study schedule as well as a list of study clinical and administrative centers. Data will be presented that describe the administrative status of the study including recruitment and forms handling. Study data reports describe demographic and baseline clinical characteristics and provide a safety assessment. Tables will be provided by site as well as for the whole study population. AE/SAE rates for each group will be presented.

Following each DSMB meeting, the NINDS will send the study's principal investigator a letter confirming that the DSMB met, reviewed all accumulated study data, and made a recommendation that the study continue as planned (or, possibly, to modify the protocol). The

principal investigator will forward a copy of this letter to each of the clinical investigators who, in turn, are responsible for forwarding it to their local IRB.

9.4.3.1. Serious Adverse Event Reporting

We divide potential adverse events by the severity:
Serious Adverse Event (SAE): Below we detail the defined SAEs with associated expected event rates and thresholds that would trigger DSMB review. Most of the SAEs would occur during the Epo treatment period, or during hospitalization and therefore would be immediately recorded.

Table 10. Serious Adverse Event (SAE)	Expected Rate	Threshold
Death	Approx. 18%	30%
Hypertension	5%	10%
Other life threatening event (e.g. cardiac arrest, pulmonary hemorrhage)	Rare < 10%	15%
Major symptomatic venous or arterial thrombosis	Rare <5%	10%
Polycythemia (hematocrit > 60%)	Rare <1%	2%
Retinopathy of Prematurity, all stages	12% (7% severe)	20% (14% sev)

Adverse Event (AE): In addition to the monitoring of SAEs we will also measure and compare rates of adverse events across the two treatment arms. Given the high-risk study population a number of adverse events are expected, and the key monitoring function will be to assess whether evidence is accruing that suggests a differential adverse event rate associated with treatment.

Table 11. Adverse Event (AE)	Expected Rate
Respiratory distress syndrome (RDS)	93%
Patent ductus arteriosus (PDA)	46%
Bronchopulmonary dysplasia (BPD)	42%
Early-onset and late-onset sepsis	36%
Intraventricular hemorrhage (IVH)	24% - 36% / 16% (severe)
Necrotizing enterocolitis (NEC)	11%
Periventricular leukomalacia (PVL)	4%

Hydrocephalus	2%
Clinical Seizures	6%-13%

Estimates here are based on three primary sources: Stoll et al. (2010); O'Shea et al. (2009) ELGAN study; and The Vermont Oxford Network (VON) 2008 Follow-up Report (2011).[8, 47, 213]

Table 12: Cumulative number of subjects evaluated for safety events and for the long-term efficacy outcome.

**Month**	**6**	**12**	**18**	**24**	**30**	**36**	**42**	**48**	**54**	**60**
**Safety (3 month)**	125	375	625	875	940					
**Efficacy (24 month)**	0	0	0	0	125	375	625	875	940	

Safety evaluation will be based on all available follow-up but we expect the majority of SAEs and AEs to occur during hospitalization and therefore within the first three months study follow-up.

Figure 7: Probability (power) of exceeding established thresholds for safety outcomes. Here we consider an expected event rate of 5% with a threshold of 10% used to trigger DSMB action.

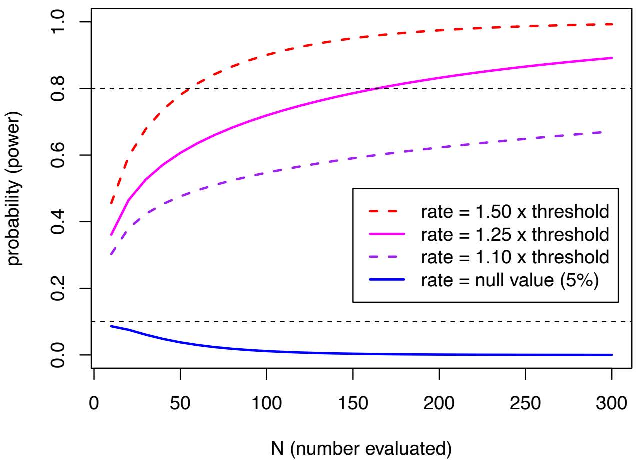

<u>SAEs and Threshold for Action</u>: For each SAE listed in Table 10 we have established expected rates of SAEs for the target study population, and thresholds for each treatment arm at which

careful evaluation would be required by DSMB review. The DSMB would be asked to review all information associated with the line listing for the SAEs and then make a decision as to whether the study should be modified, continued or stopped. In Figure 7 we show the operating characteristics for an SAE that has an expected (normal) rate of 5%, and a threshold of 10% for review. If the true SAE rate is 1.5 times the threshold (e.g. 15%) then with 100 or more subjects there is a greater than 80% probability (power) that the observed rate of greater than the threshold would occur and trigger DSMB review. However, if the true SAE rate was only 1.25 times greater than the threshold then 150 or more subjects would be needed to have 80% power. Although a total of (5) SAEs are monitored for their absolute rate we will not use any multiple comparison correction for continuous monitoring of safety leading to DSMB review. However, formal interim analysis comparing safety across the two study groups will account for both multiple comparisons due to multiple individual safety outcomes, and for multiple interim analyses.

Formal Interim Analysis: Our primary objective for interim analysis is to allow careful and continuing analysis of safety outcomes. Specifically, we propose to conduct formal statistical analysis and inference for each SAE and AE at three interim and one final analysis time. We will continue to analyze all AE events that have occurred during follow-up, but focus interim analysis on those events that occur within the first three months of follow-up since this is the time period in which the major treatment related events would be expected. We will conduct formal safety evaluation at: 6, 12, 18, and 36 months following the start of enrollment. As shown above in Table 12 we expect to have 3-month data available on 125 subjects at the first safety analysis, and 375 at one year, 625 at 2 years, and all 940 subjects by three years. For interim analysis we plan to control the overall significance level using O'Brien-Fleming boundaries.[222] In addition, we plan to conduct a separate interim analysis for death using a net alpha=0.05 significance (accounting for three interim and one final analysis), and then use a Bonferroni corrected alpha of 0.05/4 = 0.0125 for each of the other (4) SAEs. Again, for each SAE sequential monitoring boundaries will be used to control the outcome-specific family-wise error rate.

We have (6) SAE and (9) AE outcomes and therefore plan to display both a standard O'Brien-Fleming sequential monitoring guideline based on alpha=0.05, and a Bonferroni corrected sequential guideline using a significance of 0.05/10 to correct for multiple outcomes.

Since reporting rules vary by institution, by IRB, and by government agency (NIH), the following statements are a conservative guide to reporting adverse events for this trial and may be further refined with DSMB guidance.

When an SAE occurs, the PI of the CCC (S. Juul) will be notified by the site within 24 hours. She will be responsible for notifying the Safety monitor, and he/she will notify the DSMB if indicated. All events will be reported to the DSMB in semi-annual reports.

Unexpected adverse events which are serious, but not life threatening, and have a causal relation to the research, (unexpected in this context means not mentioned in the informed consent) must be reported to the DSMB and NIH within 7 days and to the local IRB within two weeks of the event. Serious adverse events will be reported to the study PI, who will distribute them to the DSMB chair, as they occur. The DSMB may call an emergency meeting, if necessary.

EXAMPLE SAFETY MONITORING TABLES

Shell Table 3. *Closed Report:* Serious adverse event (SAE) incidence rate (number of events / number at risk) and threshold for action.

Event	Group A	Group B	Expected	Threshold For Action	p-value**
Serious Adverse Events					
Death			18%	30%	
Hypertension			5%	10%	
Other life threatenin event			<10%	15%	
Major symptomatic Venous or arterial thrombosis			<5%	10%	
Polycythemia			<1%	2%	

** P-value calculated for the rate ratio comparing treatment Group A to Group B.

Shell Table 4. *Closed Report:* Adverse event (AE) incidence rate (number of events / number at risk) and comparison across groups.

Event	Group A	Group B	p-value**
Respiratory distress Syndrome			
Patent ductus arteriosus			
Bronchopulmonary dysplasia			
Early-onset or Late-onset sepsis			
Intraventricular hemorrhage			
Retinopathy of prematurity			
Necrotizing enterocolitis			
Periventricular leukomalacia			
Hydrocephalus			
Clinical seizures			
Other			

** P-value calculated for the rate ratio comparing treatment Group A to Group B.

EXAMPLE DETAILED SAFETY SUMMARIES

Event Number	Subject ID	Treatment Group	Age	Sex	Enrollment Date	Start Date	Stop Date	Frequency[1]	Serious? (Y/N)	Relationship to Study[2]	Action Taken[3]	Outcome[4]
Ex1	110001	A	70	F	22Apr2011	23Apr2011	24Apr2011	3	Y	2	1	1
Ex2	210002	B	70	M	30Oct2011	30Nov2011	30Nov2011	1	N	1	3	4

Shell Table 5. *Closed Report***:** Detailed tabulation of (Closed Report) Serious Adverse Events that have occurred during the PENUT study.

[1]Frequency: 1. Single Episode
2. Intermittent
3. Continuous

[2]Relationship: 1. Unrelated
2. Unlikely
3. Possibly related
4. Probably related
5. Definitely related

[3]Action Taken: 1. None
2. Conservative action
3. Concomitant Medication
4. Operative action

[4]Outcome: 1. Resolved, no residual effects
2. Resolved, residual effects
3. Continuing
4. Subject lost to follow-up. No data available
5. Subject died

10.0 DATA COLLECTION, SITE MONITORING, AND ADVERSE EXPERIENCE REPORTING

10.1 Records to Be Kept

All patient data will be identified by a Study Identification Number (SID). The link between the SID and the patient name/medical record number will be maintained in a locked file available only to the site investigator. This link will be destroyed after study completion, data analysis and publication of results. Retained medical record information will include maternal education, SES, other factors known to affect cognitive development (e.g. drug. alcohol exposure), date of birth, birth weight, medical treatments, complications, length of hospitalization, outcome measures including neurodevelopmental examination results and Bayley III test results, and neuroimages. All retained data will be coded with a Study Identification Number (SID) so no direct link will be available for others besides the site investigator.

Clinical Site Data Storage

Access to research data collected by the PENUT project will be restricted to study team members at each site and UW DCC personnel. The clinical recruitment sites will maintain a secure electronic database (e.g. Access, MySQL, REDCap, etc.) that links the SID generated by the PENUT portal to study subject contact information. The clinical recruitment sites are responsible for scheduling patient follow-up visits, phone calls, and collection of survey data. Efforts made to contact parents in the study-related follow-up visits will be documented in this database. The database will be stored on a secure electronic server with user name and passwords log in for individual users and will be backed up nightly.

The REDCap servers are virtual machines (VMs) located on UW DCC hardware in a secure server room. While no PHI will be collected by the UW DCC as a part of the PENUT project, this server room meets the technical requirements for HIPAA compliance and hosts other servers containing PHI.

Storage for all study data is backed by 2 dedicated Network Appliance FAS2050 storage appliances. The filer provides highly fault tolerant storage using large RAID volumes, on-line hot-spare drives, and built-in, proprietary 'snapshot' file system technology that automatically creates hourly, daily, and weekly on-line backups of modified files. Each filer provides approximately 2.6 terabytes (2.6TB) of usable storage space.

The VM Operating System will be kept fully patched and firewalled in accordance with UW Medicine Information Security Policy SEC05.04, which can be found online at http://security.uwmedicine.org/policies.

The CBS maintains a Linux-based infrastructure server that uses and manages a central department storage server. A Silicon Mechanics SM-1272A rack-mount server installed in the CBS server room rack and an HP dc5750 desktop PC are installed as so-called “logical firewalls”, such that that all PCs and operate on a private logical network.

10.2 Role of Data Management

10.2.1 Each clinical site will be responsible for data collection on enrolled subjects and data entry into the REDCap system. Each clinical site must maintain link between the subject and Study Identification Number (SID) in a secure locked file. Once the study is complete as described in 10.1, each clinical site will be responsible for destruction of link and any retained clinical information.

10.2.2 The responsibility of the Statistical Center (DCC) will be to maintain confidentiality of all the submitted data by appropriate data encryption methods and password protection entry to database. Please see DCC application for further information

10.3 Quality Assurance

Each clinical site will need local IRB approval of the study protocol prior to enrollment. Each clinical site will be

visited by Dr. Sandra Juul and the Lead study research coordinator prior to initiation of subject enrollment. This visit will focus on protocol review, data collection and entry and maintenance of quality control. In addition, the lead research coordinator will visit each clinical site on a regular basis, at least once a year to assure protocol compliance and quality data entry. These visits will include review of all pertinent records, maintenance of study and pharmacy regulatory documents, and review/resolve any data accuracy concerns. Each site will be required to make all study documents and pertinent records available for inspection by the research coordinator or other monitoring authorities.

Data Quality Assurance. We will monitor the accuracy of data entry by the sites both internally and externally. We will review all study data on arrival for completeness. We will then subject each submitted data set to a set of preliminary checks to search for values that are out-of-range or otherwise inappropriate. We will send Data Quality Queries to the sites within 2 work days if there are any missing values, out-of-range values, or ambiguous responses, and we will ask the sites to provide a response within two work days.

Once the preliminary checks have been completed, we will work with the Study Biostatistician to compile descriptive statistics by site and by assessor. If any outliers are identified, we will pursue the possibility that additional calibration training should be undertaken, either via video-recordings, or, if necessary, on-site.

External monitoring will consist of regular monitoring visits to every site while actively enrolling. Initial monitoring visits will take place prior to subject enrollment, and then every six to 12 months. All medical charts will be monitored and compared to the CRFs for meeting entry criteria, adherence to protocol, primary and secondary outcomes, and adverse event reporting. In addition, a 25% random sample of all data points in the CRFs will be compared with the medical record. Any outstanding data queries will be resolved with the research coordinator at the time of the visit. After each study site visit a report will be prepared and copies sent to the Study File, the DCC, the study PI (S. Juul), the site PI, and the site coordinator.

10.4 Adverse Experience Reporting

Data and Safety Monitoring Plans. A data and safety monitoring board (DSMB) will be created to review the accruing data quarterly to: 1) ensure that the study is adequately enrolling; 2) to ensure that there are no serious safety concerns; and 3) to assess whether the study efficacy appears overwhelming. The DSMB will be assigned by NINDS. The research coordinator at each site will monitor each subject weekly for the presence of any complications. Serious adverse events will be brought to the attention of the IRB and the DSMB in writing. An independent medical monitor will review all cases of serious adverse events. A potential risk that is unique to preterm infants is the risk of ROP.[122] In the published studies of preterm neonates receiving potentially neuroprotective doses of Epo, no difference has been noted between treatment and control groups.[36, 38, 118, 119]

As part of the Data and Safety Monitoring Plan (DSMP) we will perform continuous and interim analysis of accruing safety data. We have defined potentially treatment (Epo) related serious adverse events (SAEs) that will be monitored throughout the course of the study. Specifically, for SAEs we will compare absolute rates to expected rates based on published data for similar newborns, and will seek careful DSMB review and guidance when observed rates exceed pre-specified thresholds. In addition, at planned interim analysis we will formally compare the event rates across the two treatment groups using appropriate small sample methods such as Fisher’s exact test.

11.0 HUMAN SUBJECTS

11.1 Institutional Review Board (IRB) Review and Informed Consent

This protocol and the informed consent document and any subsequent modifications will be reviewed and approved by the IRB or ethics committee responsible for oversight of the study. A signed consent form will be obtained from the subject’s parent, legal guardian, or person with power of attorney. The consent form will describe the purpose of the study, the procedures to be followed, and the risks and benefits of participation. A copy of the consent form will be given to the subject, parent, or legal guardian, and this fact will be documented in the subject’s record. A model informed consent form is included in Appendix 10.

11.2 Subject Confidentiality

The UW DCC will support an https secured web page (https://www.penut-trial.org) that provides a centralized location for information about the Preterm Epo Neuroprotection (PENUT) Trial for patients, providers, investigators, and institutional agencies. The web page will also contain a link to the PENUT Portal https://www.penut-trial.org/portal where all research personnel will log in with individual user names and passwords to securely perform study data management activities.

The PENUT Portal controls the assignment of sequentially generated Study Identification Numbers (SID) for all patients screened under the PENUT project. All patient contact information will be linked to the SID within databases hosted at the clinical recruitment sites. No patient contact information is ever hosted by the UW DCC.

All laboratory specimens, evaluation forms, reports, video recordings, and other records that leave the site will be identified only by the SID to maintain subject confidentiality. All records will be kept in a locked file cabinet at each site. All computer entry and networking programs will be done using SIDs only. Clinical information will not be released without written permission of the subject, except as necessary for monitoring by IRB, the FDA, the NINDS, the OHRP, the sponsor, or the sponsor's designee.

Certain aspects of the subject's medial history and demographics will be collected for this study. Loss of privacy may lead to problems with insurability or social stigmatization. However, all data will be collected by study personnel with due attention to patient privacy. A Certificate of Confidentiality will be obtained by the CCC PI for all sites, to protect the subjects' confidential information.

11.3 Study Modification/Discontinuation

The study may be modified or discontinued at any time by the DSMB, IRB, the NINDS, the sponsor, the OHRP, the FDA, or other government agencies as part of their duties to ensure that research subjects are protected.

12.0 PUBLICATION OF RESEARCH FINDINGS

The DCC will be responsible for setting up systems for working with the Steering Committee to develop publication guidelines that include procedures for reviewing and tracking publications and presentations. Within the initial six months of the study a Publications Guidelines document will be developed which defines processes for defining study publications, for assigning authors in accord with JAMA criteria, and for reviewing publications prior to submission. A proposal for a manuscript will be initiated by submitting a structured summary proposal that includes an analysis plan. The proposal will be reviewed by the DCC and Steering Committee. Similarly, all abstracts, presentations, and publications must be approved by the Steering Committee, and it will be the responsibility of the DCC to ensure that the process is transparent and timely. The study website will include a searchable list of all analysis proposals and will track their status toward publication.

We expect that Epo treatment will improve NDI-free survival compared to that seen in the ELGAN and NICHD trials. We expect that this benefit will translate into shorter and less complicated hospital stays and better neurodevelopmental outcomes. This outcome of the trial would represent an important advance in the care of ELGANs that could change the standard of care for high-risk infants. If Epo treatment has no demonstrable benefit, or if adverse effects are observed, this information will also be useful in the field because Epo is currently used anecdotally in many NICUs, without trial-based evidence, for some severely ill infants. The results of the PENUT trial will be of particular interest to pediatricians trained in neonatal-perinatal medicine. This group of practitioners is largely centered in academic or medical centers and they attend several meetings each year where new information about treatment can be presented. The PENUT trial results will be presented at regional Pediatric Research Society meetings such as the Western and Eastern Society for Pediatric Research, and at national and international meetings including the Pediatric Academic Societies combined meetings, the European Society for Pediatric Research meeting, the American Academy of Pediatrics meetings, and at other venues such as "Hot Topics in Neonatology".

13.0 REFERENCES

1. Horsch S, Hallberg B, Leifsdottir K, Skiold B, Nagy Z, Mosskin M, Blennow M, Aden U. Brain abnormalities in extremely low gestational age infants: a Swedish population based MRI study. Acta Paediatr. 2007;96(7):979-84.
2. Moore T, Johnson S, Haider S, Hennessy E, Marlow N. The Bayley-III cognitive and language scales: how do scores relate to the Bayley II? Arch Dis Child. 2011;96(Suppl 1):A1–A100.
3. Rouse DJ, Hirtz DG, Thom E, Varner MW, Spong CY, Mercer BM, Iams JD, Wapner RJ, Sorokin Y, Alexander JM, Harper M, Thorp JM, Jr., Ramin SM, Malone FD, Carpenter M, Miodovnik M, Moawad A, O'Sullivan MJ, Peaceman AM, Hankins GD, Langer O, Caritis SN, Roberts JM. A randomized, controlled trial of magnesium sulfate for the prevention of cerebral palsy. N Engl J Med. 2008;359(9):895-905. PMCID: 2803083.
4. Palisano R, Rosenbaum P, Walter S, Russell D, Wood E, Galuppi B. Development and reliability of a system to classify gross motor function in children with cerebral palsy. Dev Med Child Neurol. 1997;39(4):214-23.
5. Palisano RJ, Rosenbaum P, Bartlett D, Livingston MH. Content validity of the expanded and revised Gross Motor Function Classification System. Dev Med Child Neurol. 2008;50(10):744-50.
6. Msall ME. Measuring outcomes after extreme prematurity with the Bayley-III Scales of infant and toddler development: a cautionary tale from Australia. Arch Pediatr Adolesc Med. 2010;164(4):391-3.
7. Anderson PJ, De Luca CR, Hutchinson E, Roberts G, Doyle LW. Underestimation of developmental delay by the new Bayley-III Scale. Arch Pediatr Adolesc Med. 2010;164(4):352-6.
8. Stoll BJ, Hansen NI, Bell EF, Shankaran S, Laptook AR, Walsh MC, Hale EC, Newman NS, Schibler K, Carlo WA, Kennedy KA, Poindexter BB, Finer NN, Ehrenkranz RA, Duara S, Sanchez PJ, O'Shea TM, Goldberg RN, Van Meurs KP, Faix RG, Phelps DL, Frantz ID, 3rd, Watterberg KL, Saha S, Das A, Higgins RD. Neonatal outcomes of extremely preterm infants from the NICHD Neonatal Research Network. Pediatrics. 2010;126(3):443-56. PMCID: 2982806.
9. La Pine TR, Jackson JC, Bennett FC. Outcome of infants weighing less than 800 grams at birth: 15 years' experience. Pediatrics. 1995;96(3 Pt 1):479-83.
10. Vohr BR, Wright LL, Dusick AM, Perritt R, Poole WK, Tyson JE, Steichen JJ, Bauer CR, Wilson-Costello DE, Mayes LC. Center differences and outcomes of extremely low birth weight infants. Pediatrics. 2004;113(4):781-9.
11. Hack M, Fanaroff AA. Outcomes of children of extremely low birthweight and gestational age in the 1990s. Semin Neonatol. 2000;5(2):89-106.
12. Wilson-Costello D, Friedman H, Minich N, Fanaroff AA, Hack M. Improved survival rates with increased neurodevelopmental disability for extremely low birth weight infants in the 1990s. Pediatrics. 2005;115(4):997-1003.
13. Tyson JE, Parikh NA, Langer J, Green C, Higgins RD. Intensive care for extreme prematurity--moving beyond gestational age. N Engl J Med. 2008;358(16):1672-81.
14. Eichenwald EC, Stark AR. Management and outcomes of very low birth weight. N Engl J Med. 2008;358(16):1700-11.
15. Hoekstra RE, Ferrara TB, Couser RJ, Payne NR, Connett JE. Survival and long-term neurodevelopmental outcome of extremely premature infants born at 23-26 weeks' gestational age at a tertiary center. Pediatrics. 2004;113(1 Pt 1):e1-6.
16. Hintz SR, Kendrick DE, Stoll BJ, Vohr BR, Fanaroff AA, Donovan EF, Poole WK, Blakely ML, Wright L, Higgins R. Neurodevelopmental and growth outcomes of extremely low birth weight infants after necrotizing enterocolitis. Pediatrics. 2005;115(3):696-703.
17. Wood NS, Marlow N, Costeloe K, Gibson AT, Wilkinson AR. Neurologic and developmental disability after extremely preterm birth. EPICure Study Group. N Engl J Med. 2000;343(6):378-84.
18. Wood NS, Costeloe K, Gibson AT, Hennessy EM, Marlow N, Wilkinson AR. The EPICure study: growth and associated problems in children born at 25 weeks of gestational age or less. Arch Dis Child Fetal Neonatal Ed. 2003;88(6):F492-500.

19. Wood NS, Costeloe K, Gibson AT, Hennessy EM, Marlow N, Wilkinson AR. The EPICure study: associations and antecedents of neurological and developmental disability at 30 months of age following extremely preterm birth. Arch Dis Child Fetal Neonatal Ed. 2005;90(2):F134-40.
20. Beauchamp MH, Thompson DK, Howard K, Doyle LW, Egan GF, Inder TE, Anderson PJ. Preterm infant hippocampal volumes correlate with later working memory deficits. Brain. 2008;131(Pt 11):2986-94.
21. Edgin JO, Inder TE, Anderson PJ, Hood KM, Clark CA, Woodward LJ. Executive functioning in preschool children born very preterm: relationship with early white matter pathology. J Int Neuropsychol Soc. 2008;14(1):90-101.
22. Gargus RA, Vohr BR, Tyson JE, High P, Higgins RD, Wrage LA, Poole K. Unimpaired outcomes for extremely low birth weight infants at 18 to 22 months. Pediatrics. 2009;124(1):112-21. PMCID: 2856069.
23. Marlow N, Hennessy EM, Bracewell MA, Wolke D. Motor and executive function at 6 years of age after extremely preterm birth. Pediatrics. 2007;120(4):793-804.
24. Johnson S, Hollis C, Kochhar P, Hennessy E, Wolke D, Marlow N. Autism spectrum disorders in extremely preterm children. J Pediatr. 2010;156(4):525-31.
25. Buchmayer S, Johansson S, Johansson A, Hultman CM, Sparen P, Cnattingius S. Can association between preterm birth and autism be explained by maternal or neonatal morbidity? Pediatrics. 2009;124(5):e817-25.
26. Kuban KC, O'Shea TM, Allred EN, Tager-Flusberg H, Goldstein DJ, Leviton A. Positive screening on the Modified Checklist for Autism in Toddlers (M-CHAT) in extremely low gestational age newborns. J Pediatr. 2009;154(4):535-40 e1. PMCID: 2693887.
27. Scott MN, Taylor HG, Fristad MA, Klein N, Espy KA, Minich N, Hack M. Behavior Disorders in Extremely Preterm/Extremely Low Birth Weight Children in Kindergarten. J Dev Behav Pediatr. 2012.
28. Petrou S, Henderson J, Bracewell M, Hockley C, Wolke D, Marlow N. Pushing the boundaries of viability: the economic impact of extreme preterm birth. Early Hum Dev. 2006;82(2):77-84.
29. Rushing S, Ment LR. Preterm birth: a cost benefit analysis. Semin Perinatol. 2004;28(6):444-50.
30. van der Kooij MA, Groenendaal F, Kavelaars A, Heijnen CJ, van Bel F. Neuroprotective properties and mechanisms of erythropoietin in in vitro and in vivo experimental models for hypoxia/ischemia. Brain Res Rev. 2008;59(1):22-33.
31. Gonzalez FF, Abel R, Almli CR, Mu D, Wendland M, Ferriero DM. Erythropoietin sustains cognitive function and brain volume after neonatal stroke. Dev Neurosci. 2009;31(5):403-11.
32. Kellert BA, McPherson RJ, Juul SE. A comparison of high-dose recombinant erythropoietin treatment regimens in brain-injured neonatal rats. Pediatr Res. 2007;61(4):451-5.
33. Rees S, Hale N, De Matteo R, Cardamone L, Tolcos M, Loeliger M, Mackintosh A, Shields A, Probyn M, Greenwood D, Harding R. Erythropoietin is neuroprotective in a preterm ovine model of endotoxin-induced brain injury. J Neuropathol Exp Neurol. 2010;69(3):306-19.
34. Loeliger MM, Mackintosh A, De Matteo R, Harding R, Rees SM. Erythropoietin protects the developing retina in an ovine model of endotoxin-induced retinal injury. Invest Ophthalmol Vis Sci. 2011;52(5):2656-61.
35. Juul SE, McPherson RJ, Farrell FX, Jolliffe L, Ness DJ, Gleason CA. Erytropoietin concentrations in cerebrospinal fluid of nonhuman primates and fetal sheep following high-dose recombinant erythropoietin. Biol Neonate. 2004;85(2):138-44.
36. Fauchere JC, Dame C, Vonthein R, Koller B, Arri S, Wolf M, Bucher HU. An approach to using recombinant erythropoietin for neuroprotection in very preterm infants. Pediatrics. 2008;122(2):375-82.
37. Weber A, Dzietko M, Berns M, Felderhoff-Mueser U, Heinemann U, Maier RF, Obladen M, Ikonomidou C, Buhrer C. Neuronal damage after moderate hypoxia and erythropoietin. Neurobiol Dis. 2005;20(2):594-600.
38. Juul SE, McPherson RJ, Bauer LA, Ledbetter KJ, Gleason CA, Mayock DE. A phase I/II trial of high-dose erythropoietin in extremely low birth weight infants: pharmacokinetics and safety. Pediatrics. 2008;122(2):383-91.

39. Widness JA, Veng-Pedersen P, Peters C, Pereira LM, Schmidt RL, Lowe LS. Erythropoietin pharmacokinetics in premature infants: developmental, nonlinearity, and treatment effects. J Appl Physiol. 1996;80(1):140-8.
40. Statler PA, McPherson RJ, Bauer LA, Kellert BA, Juul SE. Pharmacokinetics of high-dose recombinant erythropoietin in plasma and brain of neonatal rats. Pediatr Res. 2007;61(6):671-5.
41. Mwansa-Kambafwile J, Cousens S, Hansen T, Lawn JE. Antenatal steroids in preterm labour for the prevention of neonatal deaths due to complications of preterm birth. Int J Epidemiol. 2010;39 Suppl 1:i122-33. PMCID: 2845868.
42. Gluckman PD, Wyatt JS, Azzopardi D, Ballard R, Edwards AD, Ferriero DM, Polin RA, Robertson CM, Thoresen M, Whitelaw A, Gunn AJ. Selective head cooling with mild systemic hypothermia after neonatal encephalopathy: multicentre randomised trial. Lancet. 2005;365(9460):663-70.
43. Shankaran S, Laptook AR, Ehrenkranz RA, Tyson JE, McDonald SA, Donovan EF, Fanaroff AA, Poole WK, Wright LL, Higgins RD, Finer NN, Carlo WA, Duara S, Oh W, Cotten CM, Stevenson DK, Stoll BJ, Lemons JA, Guillet R, Jobe AH. Whole-body hypothermia for neonates with hypoxic-ischemic encephalopathy. N Engl J Med. 2005;353(15):1574-84.
44. Azzopardi DV, Strohm B, Edwards AD, Dyet L, Halliday HL, Juszczak E, Kapellou O, Levene M, Marlow N, Porter E, Thoresen M, Whitelaw A, Brocklehurst P. Moderate hypothermia to treat perinatal asphyxial encephalopathy. N Engl J Med. 2009;361(14):1349-58.
45. Edwards AD, Brocklehurst P, Gunn AJ, Halliday H, Juszczak E, Levene M, Strohm B, Thoresen M, Whitelaw A, Azzopardi D. Neurological outcomes at 18 months of age after moderate hypothermia for perinatal hypoxic ischaemic encephalopathy: synthesis and meta-analysis of trial data. BMJ. 2010;340:c363. PMCID: 2819259.
46. Field DJ, Dorling JS, Manktelow BN, Draper ES. Survival of extremely premature babies in a geographically defined population: prospective cohort study of 1994-9 compared with 2000-5. Bmj. 2008;336(7655):1221-3. PMCID: 2405852.
47. O'Shea TM, Allred EN, Dammann O, Hirtz D, Kuban KC, Paneth N, Leviton A. The ELGAN study of the brain and related disorders in extremely low gestational age newborns. Early Hum Dev. 2009;85(11):719-25. PMCID: 2801579.
48. Rajagopalan V, Scott JA, Habas PA, Corbett-Detig JM, Kim K, Rousseau F, Barkovich AJ, Glenn OA, Studholme C. Local tissue growth patterns underlying normal fetal human brain gyrification quantified in utero. J Neurosci. 2011;31(8):2878-87.
49. Lodygensky GA, Vasung L, Sizonenko SV, Huppi PS. Neuroimaging of cortical development and brain connectivity in human newborns and animal models. J Anat. 2010;217(4):418-28.
50. Huppi PS. Growth and development of the brain and impact on cognitive outcomes. Nestle Nutr Workshop Ser Pediatr Program. 2010;65:137-49; discussion 49-51.
51. Back SA, Luo NL, Borenstein NS, Levine JM, Volpe JJ, Kinney HC. Late oligodendrocyte progenitors coincide with the developmental window of vulnerability for human perinatal white matter injury. J Neurosci. 2001;21(4):1302-12.
52. Back SA, Rivkees SA. Emerging concepts in periventricular white matter injury. Semin Perinatol. 2004;28(6):405-14.
53. Volpe JJ. Brain injury in the premature infant: overview of clinical aspects, neuropathology, and pathogenesis. Semin Pediatr Neurol. 1998;5(3):135-51.
54. Volpe JJ. Neurobiology of periventricular leukomalacia in the premature infant. Pediatr Res. 2001;50(5):553-62.
55. Volpe JJ. Perinatal brain injury: From pathogenesis to neuroprotection. Ment Retard Dev Disabil Res Rev. 2001;7(1):56-64.
56. Volpe JJ. Brain injury in premature infants: a complex amalgam of destructive and developmental disturbances. Lancet Neurol. 2009;8(1):110-24. PMCID: 2707149.
57. Volpe JJ. The encephalopathy of prematurity--brain injury and impaired brain development inextricably intertwined. Semin Pediatr Neurol. 2009;16(4):167-78. PMCID: 2799246.
58. Back SA, Han BH, Luo NL, Chricton CA, Xanthoudakis S, Tam J, Arvin KL, Holtzman DM. Selective vulnerability of late oligodendrocyte progenitors to hypoxia-ischemia. J Neurosci. 2002;22(2):455-63.

59. Back SA, Luo NL, Mallinson RA, O'Malley JP, Wallen LD, Frei B, Morrow JD, Petito CK, Roberts CT, Jr., Murdoch GH, Montine TJ. Selective vulnerability of preterm white matter to oxidative damage defined by F2-isoprostanes. Ann Neurol. 2005;58(1):108-20.
60. Back SA, Riddle A, McClure MM. Maturation-dependent vulnerability of perinatal white matter in premature birth. Stroke. 2007;38(2 Suppl):724-30.
61. Volpe JJ. Neurology of the Newborn. 4th ed: W.B. Saunders; 2001.
62. Zacharias R, Schmidt M, Kny J, Sifringer M, Bercker S, Bittigau P, Buhrer C, Felderhoff-Muser U, Kerner T. Dose-dependent effects of erythropoietin in propofol anesthetized neonatal rats. Brain Res. 2010;1343:14-9.
63. Kumral A, Gonenc S, Acikgoz O, Sonmez A, Genc K, Yilmaz O, Gokmen N, Duman N, Ozkan H. Erythropoietin increases glutathione peroxidase enzyme activity and decreases lipid peroxidation levels in hypoxic-ischemic brain injury in neonatal rats. Biol Neonate. 2005;87(1):15-8.
64. Gonzalez FF, McQuillen P, Mu D, Chang Y, Wendland M, Vexler Z, Ferriero DM. Erythropoietin enhances long-term neuroprotection and neurogenesis in neonatal stroke. Dev Neurosci. 2007;29:321-30.
65. Demers EJ, McPherson RJ, Juul SE. Erythropoietin protects dopaminergic neurons and improves neurobehavioral outcomes in juvenile rats after neonatal hypoxia-ischemia. Pediatr Res. 2005;58(2):297-301.
66. McPherson RJ, Demers EJ, Juul SE. Safety of high-dose recombinant erythropoietin in a neonatal rat model. Neonatology. 2007;91(1):36-43.
67. Iwai M, Stetler RA, Xing J, Hu X, Gao Y, Zhang W, Chen J, Cao G. Enhanced oligodendrogenesis and recovery of neurological function by erythropoietin after neonatal hypoxic/ischemic brain injury. Stroke. 2010;41(5):1032-7. PMCID: 2919308.
68. Reitmeir R, Kilic E, Kilic U, Bacigaluppi M, ElAli A, Salani G, Pluchino S, Gassmann M, Hermann DM. Post-acute delivery of erythropoietin induces stroke recovery by promoting perilesional tissue remodelling and contralesional pyramidal tract plasticity. Brain. 2011;134(Pt 1):84-99.
69. Leviton A, Fichorova R, Yamamoto Y, Allred EN, Dammann O, Hecht J, Kuban K, McElrath T, O'Shea TM, Paneth N. Inflammation-related proteins in the blood of extremely low gestational age newborns. The contribution of inflammation to the appearance of developmental regulation. Cytokine. 2011;53(1):66-73. PMCID: 2987520.
70. Martin CR, Dammann O, Allred EN, Patel S, O'Shea TM, Kuban KC, Leviton A. Neurodevelopment of extremely preterm infants who had necrotizing enterocolitis with or without late bacteremia. J Pediatr. 2010;157(5):751-6 e1. PMCID: 2952050.
71. Ivacko JA, Sun R, Silverstein FS. Hypoxic-ischemic brain injury induces an acute microglial reaction in perinatal rats. Pediatr Res. 1996;39(1):39-47.
72. Foster-Barber A, Dickens B, Ferriero DM. Human perinatal asphyxia: correlation of neonatal cytokines with MRI and outcome. Dev Neurosci. 2001;23(3):213-8.
73. Bartha AI, Foster-Barber A, Miller SP, Vigneron DB, Glidden DV, Barkovich AJ, Ferriero DM. Neonatal encephalopathy: association of cytokines with MR spectroscopy and outcome. Pediatr Res. 2004;56(6):960-6.
74. Juul S, Felderhoff-Mueser U. Epo and other hematopoietic factors. Semin Fetal Neonatal Med. 2007;12(4):250-8. PMCID: 2018740.
75. Gorio A, Gokmen N, Erbayraktar S, Yilmaz O, Madaschi L, Cichetti C, Di Giulio AM, Vardar E, Cerami A, Brines M. Recombinant human erythropoietin counteracts secondary injury and markedly enhances neurological recovery from experimental spinal cord trauma. PNAS. 2002;99(14):9450-5. PMCID: 123161.
76. Villa P, Bigini P, Mennini T, Agnello D, Laragione T, Cagnotto A, Viviani B, Marinovich M, Cerami A, Coleman TR, Brines M, Ghezzi P. Erythropoietin selectively attenuates cytokine production and inflammation in cerebral ischemia by targeting neuronal apoptosis. J Exp Med. 2003;198(6):971-5. PMCID: 2194205.
77. Sun Y, Calvert JW, Zhang JH. Neonatal hypoxia/ischemia is associated with decreased inflammatory mediators after erythropoietin administration. Stroke. 2005;36(8):1672-8.

78. Siren AL, Fratelli M, Brines M, Goemans C, Casagrande S, Lewczuk P, Keenan S, Gleiter C, Pasquali C, Capobianco A, Mennini T, Heumann R, Cerami A, Ehrenreich H, Ghezzi P. Erythropoietin prevents neuronal apoptosis after cerebral ischemia and metabolic stress. PNAS. 2001;98(7):4044-9.
79. Bian XX, Yuan XS, Qi CP. Effect of recombinant human erythropoietin on serum S100B protein and interleukin-6 levels after traumatic brain injury in the rat. Neurol Med Chir (Tokyo). 2010;50(5):361-6.
80. Juul SE, Beyer RP, Bammler TK, McPherson RJ, Wilkerson J, Farin FM. Microarray analysis of high-dose recombinant erythropoietin treatment of unilateral brain injury in neonatal mouse hippocampus. Pediatr Res. 2009;65(5):485-92.
81. Yatsiv I, Grigoriadis N, Simeonidou C, Stahel PF, Schmidt OI, Alexandrovitch AG, Tsenter J, Shohami E. Erythropoietin is neuroprotective, improves functional recovery, and reduces neuronal apoptosis and inflammation in a rodent model of experimental closed head injury. FASEB J. 2005;19(12):1701-3.
82. Vitellaro-Zuccarello L, Mazzetti S, Madaschi L, Bosisio P, Fontana E, Gorio A, De Biasi S. Chronic erythropoietin-mediated effects on the expression of astrocyte markers in a rat model of contusive spinal cord injury. Neuroscience. 2008;151(2):452-66.
83. Li L, Jiang Q, Ding G, Zhang L, Zhang ZG, Li Q, Panda S, Kapke A, Lu M, Ewing JR, Chopp M. MRI identification of white matter reorganization enhanced by erythropoietin treatment in a rat model of focal ischemia. Stroke. 2009;40(3):936-41. PMCID: 2730918.
84. Zhang L, Chopp M, Zhang RL, Wang L, Zhang J, Wang Y, Toh Y, Santra M, Lu M, Zhang ZG. Erythropoietin amplifies stroke-induced oligodendrogenesis in the rat. PLoS ONE. 2010;5(6):e11016. PMCID: 2884017.
85. Yamada M, Burke C, Colditz P, Johnson DW, Gobe GC. Erythropoietin protects against apoptosis and increases expression of non-neuronal cell markers in the hypoxia-injured developing brain. J Pathol. 2011.
86. Savino C, Pedotti R, Baggi F, Ubiali F, Gallo B, Nava S, Bigini P, Barbera S, Fumagalli E, Mennini T, Vezzani A, Rizzi M, Coleman T, Cerami A, Brines M, Ghezzi P, Bianchi R. Delayed administration of erythropoietin and its non-erythropoietic derivatives ameliorates chronic murine autoimmune encephalomyelitis. J Neuroimmunol. 2006;172(1-2):27-37.
87. Vitellaro-Zuccarello L, Mazzetti S, Madaschi L, Bosisio P, Gorio A, De Biasi S. Erythropoietin-mediated preservation of the white matter in rat spinal cord injury. Neuroscience. 2007;144(3):865-77.
88. Ha-Vinh Leuchter R, Gui L, Hagmann C, Lodygensky GA, Sizonenko S, Martin E, Koller B, Bucher HU, Hüppi PS. Neuroprotective effects of early Epo in preterm infants: an MRI study. Pediatric Research. 2011;70:28.
89. Sugawa M, Sakurai Y, Ishikawa-Ieda Y, Suzuki H, Asou H. Effects of erythropoietin on glial cell development; oligodendrocyte maturation and astrocyte proliferation. Neurosci Res. 2002;44(4):391-403.
90. Genc K, Genc S, Baskin H, Semin I. Erythropoietin decreases cytotoxicity and nitric oxide formation induced by inflammatory stimuli in rat oligodendrocytes. Physiol Res. 2006;55(1):33-8.
91. Mizuno K, Hida H, Masuda T, Nishino H, Togari H. Pretreatment with low doses of erythropoietin ameliorates brain damage in periventricular leukomalacia by targeting late oligodendrocyte progenitors: a rat model. Neonatology. 2008;94(4):255-66.
92. Oppenheim RW. Cell death during development of the nervous system. Annu Rev Neurosci. 1991;14:453-501.
93. McDonald JW, Behrens MI, Chung C, Bhattacharyya T, Choi DW. Susceptibility to apoptosis is enhanced in immature cortical neurons. Brain Res. 1997;759(2):228-32.
94. Digicaylioglu M, Lipton SA. Erythropoietin-mediated neuroprotection involves cross-talk between Jak2 and NF-kappaB signalling cascades. Nature. 2001;412(6847):641-7.
95. Xiong T, Qu Y, Mu D, Ferriero D. Erythropoietin for neonatal brain injury: opportunity and challenge. Int J Dev Neurosci. 2011.
96. Dzietko M, Felderhoff-Mueser U, Sifringer M, Krutz B, Bittigau P, Thor F, Heumann R, Buhrer C, Ikonomidou C, Hansen HH. Erythropoietin protects the developing brain against N-methyl-D-aspartate receptor antagonist neurotoxicity. Neurobiol Dis. 2004;15(2):177-87.

97. Wang L, Zhang Z, Wang Y, Zhang R, Chopp M. Treatment of stroke with erythropoietin enhances neurogenesis and angiogenesis and improves neurological function in rats. Stroke. 2004;35(7):1732-7.
98. Iwai M, Cao G, Yin W, Stetler RA, Liu J, Chen J. Erythropoietin promotes neuronal replacement through revascularization and neurogenesis after neonatal hypoxia/ischemia in rats. Stroke 2007;38:2795-803.
99. Shingo T, Sorokan ST, Shimazaki T, Weiss S. Erythropoietin regulates the in vitro and in vivo production of neuronal progenitors by mammalian forebrain neural stem cells. J Neurosci. 2001;21(24):9733-43.
100. Osredkar D, Sall JW, Bickler PE, Ferriero DM. Erythropoietin promotes hippocampal neurogenesis in in vitro models of neonatal stroke. Neurobiol Dis. 2010;38(2):259-65. PMCID: 2854222.
101. Ransome MI, Turnley AM. Systemically delivered Erythropoietin transiently enhances adult hippocampal neurogenesis. J Neurochem. 2007;102(6):1953-65.
102. Bocker-Meffert S, Rosenstiel P, Rohl C, Warneke N, Held-Feindt J, Sievers J, Lucius R. Erythropoietin and VEGF promote neural outgrowth from retinal explants in postnatal rats. Invest Ophthalmol Vis Sci. 2002;43(6):2021-6.
103. Yang Z, Covey MV, Bitel CL, Ni L, Jonakait GM, Levison SW. Sustained neocortical neurogenesis after neonatal hypoxic/ischemic injury. Ann Neurol. 2007;61(3):199-208.
104. Wang L, Chopp M, Gregg SR, Zhang RL, Teng H, Jiang A, Feng Y, Zhang ZG. Neural progenitor cells treated with EPO induce angiogenesis through the production of VEGF. J Cereb Blood Flow Metab. 2008;28(7):1361-8.
105. Marzocchi B, Perrone S, Paffetti P, Magi B, Bini L, Tani C, Longini M, Buonocore G. Nonprotein-bound iron and plasma protein oxidative stress at birth. Pediatr Res. 2005;58(6):1295-9.
106. Buonocore G, Perrone S, Longini M, Paffetti P, Vezzosi P, Gatti MG, Bracci R. Non protein bound iron as early predictive marker of neonatal brain damage. Brain. 2003;126(Pt 5):1224-30.
107. Collard KJ. Is there a causal relationship between the receipt of blood transfusions and the development of chronic lung disease of prematurity? Med Hypotheses. 2006;66(2):355-64.
108. Ozment CP, Turi JL. Iron overload following red blood cell transfusion and its impact on disease severity. Biochim Biophys Acta. 2009;1790(7):694-701.
109. Juul SE, Zerzan JC, Strandjord TP, Woodrum DE. Zinc protoporphyrin/heme as an indicator of iron status in NICU patients. J Pediatr. 2003;142(3):273-8.
110. Wallach I, Zhang J, Hartmann A, van Landeghem FK, Ivanova A, Klar M, Dame C. Erythropoietin-receptor gene regulation in neuronal cells. Pediatr Res. 2009;65(6):619-24.
111. Nagai A, Nakagawa E, Choi HB, Hatori K, Kobayashi S, Kim SU. Erythropoietin and erythropoietin receptors in human CNS neurons, astrocytes, microglia, and oligodendrocytes grown in culture. J Neuropathol Exp Neurol. 2001;60(4):386-92.
112. Chong ZZ, Kang JQ, Maiese K. Erythropoietin fosters both intrinsic and extrinsic neuronal protection through modulation of microglia, Akt1, Bad, and caspase-mediated pathways. Br J Pharmacol. 2003;138(6):1107-18.
113. Kumral A, Tugyan K, Gonenc S, Genc K, Genc S, Sonmez U, Yilmaz O, Duman N, Uysal N, Ozkan H. Protective effects of erythropoietin against ethanol-induced apoptotic neurodegenaration and oxidative stress in the developing C57BL/6 mouse brain. Brain Res Dev Brain Res. 2005;160(2):146-56.
114. Chattopadhyay A, Choudhury TD, Bandyopadhyay D, Datta AG. Protective effect of erythropoietin on the oxidative damage of erythrocyte membrane by hydroxyl radical. Biochem Pharmacol. 2000;59(4):419-25.
115. Zhu C, Kang W, Xu F, Cheng X, Zhang Z, Jia L, Ji L, Guo X, Xiong H, Simbruner G, Blomgren K, Wang X. Erythropoietin improved neurologic outcomes in newborns with hypoxic-ischemic encephalopathy. Pediatrics. 2009;124(2):e218-26.
116. Elmahdy H, El-Mashad AR, El-Bahrawy H, El-Gohary T, El-Barbary A, Aly H. Human recombinant erythropoietin in asphyxia neonatorum: pilot trial. Pediatrics. 2010;125(5):e1135-42.
117. Brown MS, Eichorst D, Lala-Black B, Gonzalez R. Higher cumulative doses of erythropoietin and developmental outcomes in preterm infants. Pediatrics. 2009;124(4):e681-7.

118. Neubauer AP, Voss W, Wachtendorf M, Jungmann T. Erythropoietin improves neurodevelopmental outcome of extremely preterm infants. Ann Neurol. 2010;67(5):657-66.
119. Bierer R, Peceny MC, Hartenberger CH, Ohls RK. Erythropoietin concentrations and neurodevelopmental outcome in preterm infants. Pediatrics. 2006;118(3):e635-40.
120. McAdams RM, McPherson RJ, Mayock DE, Juul SE. Neurodevelopmental Outcomes For Extremely Low Birth Weight Infants Given Neonatal High-Dose Erythropoietin. J Perinatol. 2012;In Press.
121. Ohls RK. The use of erythropoietin in neonates. Clin Perinatol. 2000;27(3):681-96.
122. Ohlsson A, Aher SM. Early erythropoietin for preventing red blood cell transfusion in preterm and/or low birth weight infants. Cochrane Database Syst Rev. 2006;3:CD004863.
123. Doege C, Pritsch M, Fruhwald MC, Bauer J. An association between infantile haemangiomas and erythropoietin treatment in preterm infants. Arch Dis Child Fetal Neonatal Ed. 2012;97(1):F45-9.
124. Fisher L, Dixon DO, Herson J, al. e. Intention to treat in clinical trials. In: Peace KE, editor. Statistical Issues in Drug Research and Development. New York: Marcel Dekker; 1991. p. 331-50.
125. Diggle PJ, Heagerty PJ, Liang KY, Zeger SL. Analysis of Longitudinal Data. Second Edition ed: Oxford University Press; 2002.
126. Wu YW, Bauer LA, Ballard RA, Ferriero DM, Glidden DV, Mayock DE, Chang, Durand DJ, Song D, Bonifacio SL, Gonzalez FF, Glass HC, Juul SE. High-dose erythropoietin for neuroprotection in neonatal encephalopathy: a Phase I Safety and Pharmacokinetic Study. Pediatrics. 2012;In Press.
127. Ohls RK, Ehrenkranz RA, Wright LL, Lemons JA, Korones SB, Stoll BJ, Stark AR, Shankaran S, Donovan EF, Close NC, Das A. Effects of early erythropoietin therapy on the transfusion requirements of preterm infants below 1250 grams birth weight: a multicenter, randomized, controlled trial. Pediatrics. 2001;108(4):934-42.
128. Ribatti D, Presta M, Vacca A, Ria R, Giuliani R, Dell'Era P, Nico B, Roncali L, Dammacco F. Human erythropoietin induces a pro-angiogenic phenotype in cultured endothelial cells and stimulates neovascularization In vivo. Blood. 1999;93(8):2627-36.
129. Chen J, Smith LE. A double-edged sword: erythropoietin eyed in retinopathy of prematurity. J Aapos. 2008;12(3):221-2.
130. Slusarski JD, McPherson RJ, Wallace GN, Juul SE. High-dose erythropoietin does not exacerbate retinopathy of prematurity in rats. Pediatr Res. 2009;66(6):625-30.
131. Aher S, Ohlsson A. Late erythropoietin for preventing red blood cell transfusion in preterm and/or low birth weight infants. Cochrane Database Syst Rev. 2006;3:CD004868.
132. Aher SM, Ohlsson A. Early versus late erythropoietin for preventing red blood cell transfusion in preterm and/or low birth weight infants. Cochrane Database Syst Rev. 2006;3:CD004865.
133. Romagnoli C, Zecca E, Gallini F, Girlando P, Zuppa AA. Do recombinant human erythropoietin and iron supplementation increase the risk of retinopathy of prematurity? Eur J Pediatr. 2000;159(8):627-8.
134. Flynn JT. Neonatal Hypertension: Diagnosis and management. Pediatr Nephrol. 2000;14:332-41.
135. AAP. Screening examination of premature infants for retinopathy of prematurity. Pediatrics. 2006;117(2):572-6.
136. Dionne JM, Abitbol CL, Flynn JT. Hypertension in infancy: diagnosis, management and outcome. Pediatr Nephrol. 2011.
137. Sunder-Plassmann G, Patruta SI, Horl WH. Pathobiology of the role of iron in infection. Am J Kidney Dis. 1999;34(4 Suppl 2):S25-9.
138. Dammann O, Naples M, Bednarek F, Shah B, Kuban KC, O'Shea TM, Paneth N, Allred EN, Leviton A. SNAP-II and SNAPPE-II and the risk of structural and functional brain disorders in extremely low gestational age newborns: the ELGAN study. Neonatology. 2010;97(2):71-82. PMCID: 2790760.
139. Leviton A, Allred EN, Kuban KC, Hecht JL, Onderdonk AB, O'Shea T M, Paneth N. Microbiologic and histologic characteristics of the extremely preterm infant's placenta predict white matter damage and later cerebral palsy. the ELGAN study. Pediatric Research. 2010;67(1):95-101. PMCID: 2794973.
140. Leviton A, Dammann O, Engelke S, Allred E, Kuban KC, O'Shea TM, Paneth N. The clustering of disorders in infants born before the 28th week of gestation. Acta Paediatrica. 2010;99(12):1795-800.
141. O'Shea TM, Allred EN, Kuban KC, Dammann O, Paneth N, Fichorova R, Hirtz D, Leviton A. Elevated concentrations of inflammation-related proteins in postnatal blood predict severe developmental delay

at 2 years of age in extremely preterm infants. The Journal of Pediatrics. 2012;160(3):395-401 e4. PMCID: 3279610.
142. Howie SRC. Blood sample volumes in child health research: review of safe limits. Bulletin of the World Health Organization. 2011;89(1):46-53.
143. Ment LR, Bada HS, Barnes P, Grant PE, Hirtz D, Papile LA, Pinto-Martin J, Rivkin M, Slovis TL. Practice parameter: neuroimaging of the neonate: report of the Quality Standards Subcommittee of the American Academy of Neurology and the Practice Committee of the Child Neurology Society. Neurology. 2002;58(12):1726-38.
144. Mathur AM, Neil JJ, McKinstry RC, Inder TE. Transport, monitoring, and successful brain MR imaging in unsedated neonates. Pediatr Radiol. 2008;38(3):260-4.
145. Franz AR, Mihatsch WA, Sander S, Kron M, Pohlandt F. Prospective randomized trial of early versus late enteral iron supplementation in infants with a birth weight of less than 1301 grams. Pediatrics. 2000;106(4):700-6.
146. Silverstein SB, Rodgers GM. Parenteral iron therapy options. American Journal of Hematology. 2004;76(1):74-8.
147. Habas PA, Kim K, Corbett-Detig JM, Rousseau F, Glenn OA, Barkovich AJ, Studholme C. A spatiotemporal atlas of MR intensity, tissue probability and shape of the fetal brain with application to segmentation. Neuroimage. 2010;53(2):460-70. PMCID: 2930902.
148. Habas PA, Kim K, Rousseau F, Glenn OA, Barkovich AJ, Studholme C. Atlas-based segmentation of the germinal matrix from in utero clinical MRI of the fetal brain. Med Image Comput Comput Assist Interv. 2008;11(Pt 1):351-8.
149. Rousseau F, Habas PA, Studholme C. A supervised patch-based approach for human brain labeling. IEEE Trans Med Imaging. 2011;In Press.
150. Davatzikos C, Vaillant M, Resnick SM, Prince JL, Letovsky S, Bryan RN. A computerized approach for morphological analysis of the corpus callosum. J Comput Assist Tomogr. 1996;20(1):88-97.
151. Friston K, Holmes A, Worsley K, Poline J, Frith C, Frackowiak RSJ. Statistical parametric maps in functional imaging: A general linear approach. Human Brain Mapping. 1995;2:189-210.
152. Nichols TE, Holmes AP. Nonparametric permutation tests for functional neuroimaging: a primer with examples. Human Brain Mapping. 2002;15(1):1-25.
153. Habas PA, Scott JA, Roosta A, Rajagopalan V, Kim K, Rousseau F, Barkovich AJ, Glenn OA, Studholme C. Early folding patterns and asymmetries of the normal human brain detected from in utero MRI. Cereb Cortex. 2011;In Press.
154. Rodriguez-Carranza CE, Mukherjee P, Vigneron D, Barkovich J, Studholme C. A framework for in vivo quantification of regional brain folding in premature neonates. Neuroimage. 2008;41(2):462-78. PMCID: 2741002.
155. Lopes A, Brodlie K. Improving the robustness and accuracy of the marching cubes algorithm for isosurfacing. . IEEE Trans Vis Comput Graph. 2003;9:16-29.
156. Inder TE, Wells SJ, Mogridge NB, Spencer C, Volpe JJ. Defining the nature of the cerebral abnormalities in the premature infant: a qualitative magnetic resonance imaging study. J Pediatr. 2003;143(2):171-9.
157. Inder TE, Warfield SK, Wang H, Huppi PS, Volpe JJ. Abnormal cerebral structure is present at term in premature infants. Pediatrics. 2005;115(2):286-94.
158. Woodward LJ, Anderson PJ, Austin NC, Howard K, Inder TE. Neonatal MRI to predict neurodevelopmental outcomes in preterm infants. N Engl J Med. 2006;355(7):685-94.
159. Habas PA, Corbett-Detig JM, Kim K, Rousseau F, Glenn OA, Barkovich AJ, Studholme C, editors. Global and regional patterns of tissue volume growth in the normal fetal brain from in utero MRI. 16th Annual Meeting of the Organization for Human Brain Mapping; 2010; Barcelona, Spain.
160. Jenkinson M, Smith S. A global optimisation method for robust affine registration of brain images. Med Image Anal. 2001;5(2):143-56.
161. Jenkinson M. Fast, automated, N-dimensional phase-unwrapping algorithm. Magn Reson Med. 2003;49(1):193-7.

162. Smith SM, Jenkinson M, Johansen-Berg H, Rueckert D, Nichols TE, Mackay CE, Watkins KE, Ciccarelli O, Cader MZ, Matthews PM, Behrens TE. Tract-based spatial statistics: voxelwise analysis of multi-subject diffusion data. Neuroimage. 2006;31(4):1487-505.
163. Richards T, Stevenson J, Crouch J, Johnson LC, Maravilla K, Stock P, Abbott R, Berninger V. Tract-Based Spatial Statistics of Diffusion Tensor Imaging in Adults with Dyslexia. American Journal of Neuroradiology. 2008;29:1134-9.
164. Weaver KE, Richards TL, Liang O, Laurino MY, Samii A, Aylward EH. Longitudinal diffusion tensor imaging in Huntington's Disease. Exp Neurol. 2009;216(2):525-9.
165. Heide AC, Richards TL, Alvord EC, Jr., Peterson J, Rose LM. Diffusion imaging of experimental allergic encephalomyelitis. Magn Reson Med. 1993;29(4):478-84.
166. Richards TL, Alvord EC, Jr., He Y, Petersen K, Peterson J, Cosgrove S, Heide AC, Marro K, Rose LM. Experimental allergic encephalomyelitis in non-human primates: diffusion imaging of acute and chronic brain lesions. Mult Scler. 1995;1(2):109-17.
167. Douglas-Escobar M, Yang C, Bennett J, Shuster J, Theriaque D, Leibovici A, Kays D, Zheng T, Rossignol C, Shaw G, Weiss MD. A pilot study of novel biomarkers in neonates with hypoxic-ischemic encephalopathy. Pediatric Research. 2010;68(6):531-6.
168. Ehrenreich H, Kastner A, Weissenborn K, Streeter J, Sperling S, Wang KK, Worthmann H, Hayes RL, Von Ahsen N, Kastrup A, Jeromin A, Herrmann M. Circulating damage marker profiles support a neuroprotective effect of erythropoietin in ischemic stroke patients. Mol Med. 2011.
169. Ennen CS, Huisman TA, Savage WJ, Northington FJ, Jennings JM, Everett AD, Graham EM. Glial fibrillary acidic protein as a biomarker for neonatal hypoxic-ischemic encephalopathy treated with whole-body cooling. Am J Obstet Gynecol. 2011;205(3):251 e1-7.
170. Leviton A, Kuban K, O'Shea TM, Paneth N, Fichorova R, Allred EN, Dammann O. The relationship between early concentrations of 25 blood proteins and cerebral white matter injury in preterm newborns: the ELGAN study. The Journal of Pediatrics. 2011;158(6):897-903 e1-5.
171. Leviton A, Kuban KC, Allred EN, Fichorova RN, O'Shea TM, Paneth N. Early postnatal blood concentrations of inflammation-related proteins and microcephaly two years later in infants born before the 28th post-menstrual week. Early Hum Dev. 2011;87(5):325-30.
172. Florio P, Abella R, Marinoni E, Di Iorio R, Li Volti G, Galvano F, Pongiglione G, Frigiola A, Pinzauti S, Petraglia F, Gazzolo D. Biochemical markers of perinatal brain damage. Front Biosci (Elite Ed). 2010;2:47-72.
173. Bembea MM, Savage W, Strouse JJ, Schwartz JM, Graham E, Thompson CB, Everett A. Glial fibrillary acidic protein as a brain injury biomarker in children undergoing extracorporeal membrane oxygenation. Pediatr Crit Care Med. 2011;12(5):572-9.
174. Honda M, Tsuruta R, Kaneko T, Kasaoka S, Yagi T, Todani M, Fujita M, Izumi T, Maekawa T. Serum glial fibrillary acidic protein is a highly specific biomarker for traumatic brain injury in humans compared with S-100B and neuron-specific enolase. J Trauma. 2010;69(1):104-9.
175. Msall ME. Neurodevelopmental surveillance in the first 2 years after extremely preterm birth: evidence, challenges, and guidelines. Early Hum Dev. 2006;82(3):157-66.
176. Msall ME. The limits of viability and the uncertainty of neuroprotection: challenges in optimizing outcomes in extreme prematurity. Pediatrics. 2007;119(1):158-60.
177. Msall ME, Park JJ. The spectrum of behavioral outcomes after extreme prematurity: regulatory, attention, social, and adaptive dimensions. Semin Perinatol. 2008;32(1):42-50.
178. Duffner PK, Granger C, Lyon N, Niewczyk P, Barczykowski A, Bauer S, Msall ME. Developmental and Functional Outcomes in Children with a Positive Newborn Screen for Krabbe Disease: A Pilot Study of a Phone-Based Interview Surveillance Technique. The Journal of Pediatrics. 2012.
179. Laptook AR, O'Shea TM, Shankaran S, Bhaskar B. Adverse neurodevelopmental outcomes among extremely low birth weight infants with a normal head ultrasound: prevalence and antecedents. Pediatrics. 2005;115(3):673-80.
180. Hintz SR, Kendrick DE, Vohr BR, Poole WK, Higgins RD. Changes in neurodevelopmental outcomes at 18 to 22 months' corrected age among infants of less than 25 weeks' gestational age born in 1993-1999. Pediatrics. 2005;115(6):1645-51.

181. Hack M, Taylor HG, Drotar D, Schluchter M, Cartar L, Wilson-Costello D, Klein N, Friedman H, Mercuri-Minich N, Morrow M. Poor predictive validity of the Bayley Scales of Infant Development for cognitive function of extremely low birth weight children at school age. Pediatrics. 2005;116(2):333-41.
182. Roberts G, Anderson PJ, Doyle LW. The stability of the diagnosis of developmental disability between ages 2 and 8 in a geographic cohort of very preterm children born in 1997. Arch Dis Child. 2010;95(10):786-90.
183. Schmidt B, Anderson PJ, Doyle LW, Dewey D, Grunau RE, Asztalos EV, Davis PG, Tin W, Moddemann D, Solimano A, Ohlsson A, Barrington KJ, Roberts RS. Survival without disability to age 5 years after neonatal caffeine therapy for apnea of prematurity. JAMA. 2012;307(3):275-82.
184. Potharst ES, Houtzager BA, van Sonderen L, Tamminga P, Kok JH, Last BF, van Wassenaer AG. Prediction of cognitive abilities at the age of 5 years using developmental follow-up assessments at the age of 2 and 3 years in very preterm children. Dev Med Child Neurol. 2012;54(3):240-6.
185. Munck P, Niemi P, Lapinleimu H, Lehtonen L, Haataja L. Stability of cognitive outcome from 2 to 5 years of age in very low birth weight children. Pediatrics. 2012;129(3):503-8.
186. Bayley N. Bayley scales of infant and toddler development, third edition. San Antonio, TX: Harcourt Assessment, Inc; 2006.
187. Vohr BR, Stephens BE, Higgins RD, Bann CM, Hintz SR, Das A, Newman JE, Peralta-Carcelen M, Yolton K, Dusick AM, Evans PW, Goldstein RF, Ehrenkranz RA, Pappas A, Adams-Chapman I, Wilson-Costello DE, Bauer CR, Bodnar A, Heyne RJ, Vaucher YE, Dillard RG, Acarregui MJ, McGowan EC, Myers GJ, Fuller J. Are Outcomes of Extremely Preterm Infants Improving? Impact of Bayley Assessment on Outcomes. The Journal of Pediatrics. 2012.
188. Robertson CM, Hendson L, Biggs WS, Acton BV. Application of the Flynn effect for the Bayley III Scales. Arch Pediatr Adolesc Med. 2010;164(11):1072-3; author reply 3.
189. Msall ME. Overestimating neuroprotection in congenital heart disease: problems with Bayley III outcomes. Pediatrics. 2011;128(4):e993-4.
190. Lowe JR, Erickson SJ, Schrader R, Duncan AF. Comparison of the Bayley II Mental Developmental Index and the Bayley III Cognitive Scale: are we measuring the same thing? Acta Paediatrica. 2012;101(2):e55-8.
191. Silveira RC, Filipouski GR, Goldstein DJ, O’Shea TM, Procianoy RS. Agreement between Bayley Scales Second and Third Edition Assessments of Very Low Birth Weight Infants. Archives of Pediatric and Adolescent Medicine. 2012;In Press.
192. Johnson S, Wolke D, Hennessy E, Marlow N. Educational outcomes in extremely preterm children: neuropsychological correlates and predictors of attainment. Dev Neuropsychol. 2011;36(1):74-95.
193. Kuban KC, O'Shea M, Allred E, Leviton A, Gilmore H, DuPlessis A, Krishnamoorthy K, Hahn C, Soul J, O'Connor SE, Miller K, Church PT, Keller C, Bream R, Adair R, Miller A, Romano E, Bassan H, Kerkering K, Engelke S, Marshall D, Milowic K, Wereszczak J, Hubbard C, Washburn L, Dillard R, Heller C, Burdo-Hartman W, Fagerman L, Sutton D, Karna P, Olomu N, Caldarelli L, Oca M, Lohr K, Scheiner A. Video and CD-ROM as a training tool for performing neurologic examinations of 1-year-old children in a multicenter epidemiologic study. J Child Neurol. 2005;20(10):829-31.
194. Kuban KC, Allred EN, O'Shea M, Paneth N, Pagano M, Leviton A. An algorithm for identifying and classifying cerebral palsy in young children. The Journal of Pediatrics. 2008;153(4):466-72. PMCID: 2581842.
195. Palisano RJ, Cameron D, Rosenbaum PL, Walter SD, Russell D. Stability of the gross motor function classification system. Dev Med Child Neurol. 2006;48(6):424-8.
196. Rosenbaum PL, Palisano RJ, Bartlett DJ, Galuppi BE, Russell DJ. Development of the Gross Motor Function Classification System for cerebral palsy. Dev Med Child Neurol. 2008;50(4):249-53.
197. Johnson S, Marlow N. Preterm Birth and Childhood Psychiatric Disorders. Pediatric Research. 2011.
198. Mulder H, Pitchford NJ, Marlow N. Inattentive behaviour is associated with poor working memory and slow processing speed in very pre-term children in middle childhood. Br J Educ Psychol. 2011;81(1):147-60.

199. Lind A, Korkman M, Lehtonen L, Lapinleimu H, Parkkola R, Matomaki J, Haataja L. Cognitive and neuropsychological outcomes at 5 years of age in preterm children born in the 2000s. Dev Med Child Neurol. 2011;53(3):256-62.
200. Raz S, Debastos AK, Newman JB, Batton D. Extreme prematurity and neuropsychological outcome in the preschool years. J Int Neuropsychol Soc. 2010;16(1):169-79.
201. Limperopoulos C. Autism spectrum disorders in survivors of extreme prematurity. Clin Perinatol. 2009;36(4):791-805, vi.
202. Kuban KC, Allred EN, O'Shea TM, Paneth N, Westra S, Miller C, Rosman NP, Leviton A. Developmental correlates of head circumference at birth and two years in a cohort of extremely low gestational age newborns. The Journal of Pediatrics. 2009;155(3):344-9 e1-3. PMCID: 2803763.
203. Kontis D, Catani M, Cuddy M, Walshe M, Nosarti C, Jones D, Wyatt J, Rifkin L, Murray R, Allin M. Diffusion tensor MRI of the corpus callosum and cognitive function in adults born preterm. Neuroreport. 2009;20(4):424-8.
204. Gaddlin PO, Finnstrom O, Samuelsson S, Wadsby M, Wang C, Leijon I. Academic achievement, behavioural outcomes and MRI findings at 15 years of age in very low birthweight children. Acta Paediatrica. 2008;97(10):1426-32.
205. Nagy Z, Ashburner J, Andersson J, Jbabdi S, Draganski B, Skare S, Bohm B, Smedler AC, Forssberg H, Lagercrantz H. Structural correlates of preterm birth in the adolescent brain. Pediatrics. 2009;124(5):e964-72.
206. Mathur AM, Neil JJ, Inder TE. Understanding brain injury and neurodevelopmental disabilities in the preterm infant: the evolving role of advanced magnetic resonance imaging. Semin Perinatol. 2010;34(1):57-66.
207. Wustenberg T, Begemann M, Bartels C, Gefeller O, Stawicki S, Hinze-Selch D, Mohr A, Falkai P, Aldenhoff JB, Knauth M, Nave KA, Ehrenreich H. Recombinant human erythropoietin delays loss of gray matter in chronic schizophrenia. Mol Psychiatry. 2010.
208. Northam GB, Liegeois F, Chong WK, J SW, Baldeweg T. Total brain white matter is a major determinant of IQ in adolescents born preterm. Annals of neurology. 2011.
209. Woodward LJ, Clark CA, Pritchard VE, Anderson PJ, Inder TE. Neonatal white matter abnormalities predict global executive function impairment in children born very preterm. Dev Neuropsychol. 2011;36(1):22-41.
210. Walsh MC, Yao Q, Gettner P, Hale E, Collins M, Hensman A, Everette R, Peters N, Miller N, Muran G, Auten K, Newman N, Rowan G, Grisby C, Arnell K, Miller L, Ball B, McDavid G. Impact of a physiologic definition on bronchopulmonary dysplasia rates. Pediatrics. 2004;114(5):1305-11.
211. Screening examination of premature infants for retinopathy of prematurity. Pediatrics. 2006;117(2):572-6.
212. The International Classification of Retinopathy of Prematurity revisited. Arch Ophthalmol. 2005;123(7):991-9.
213. Vermont Oxford Network ELBW Follow-up Report Birth Year 2008 All Centers [database on the Internet]Sept 2011.
214. Volpe JJ. Cerebellum of the premature infant: rapidly developing, vulnerable, clinically important. J Child Neurol. 2009;24(9):1085-104. PMCID: 2799249.
215. Limperopoulos C, Chilingaryan G, Guizard N, Robertson RL, du Plessis AJ. Cerebellar injury in the premature infant is associated with impaired growth of specific cerebral regions. Pediatr Res. 2010.
216. Limperopoulos C, Bassan H, Gauvreau K, Robertson RL, Jr., Sullivan NR, Benson CB, Avery L, Stewart J, Soul JS, Ringer SA, Volpe JJ, duPlessis AJ. Does cerebellar injury in premature infants contribute to the high prevalence of long-term cognitive, learning, and behavioral disability in survivors? Pediatrics. 2007;120(3):584-93.
217. Limperopoulos C, Soul JS, Gauvreau K, Huppi PS, Warfield SK, Bassan H, Robertson RL, Volpe JJ, du Plessis AJ. Late gestation cerebellar growth is rapid and impeded by premature birth. Pediatrics. 2005;115(3):688-95.

218. Messerschmidt A, Pataraia A, Helmer H, Kasprian G, Sauer A, Brugger PC, Pollak A, Weber M, Prayer D. Fetal MRI for prediction of neonatal mortality following preterm premature rupture of the fetal membranes. Pediatr Radiol. 2011;41(11):1416-20.
219. Messerschmidt A, Fuiko R, Prayer D, Brugger PC, Boltshauser E, Zoder G, Sterniste W, Weber M, Birnbacher R. Disrupted cerebellar development in preterm infants is associated with impaired neurodevelopmental outcome. Eur J Pediatr. 2008;167(10):1141-7.
220. Limperopoulos C, Robertson RL, Sullivan NR, Bassan H, du Plessis AJ. Cerebellar injury in term infants: clinical characteristics, magnetic resonance imaging findings, and outcome. Pediatr Neurol. 2009;41(1):1-8.
221. Tam EW, Rosenbluth G, Rogers EE, Ferriero DM, Glidden D, Goldstein RB, Glass HC, Piecuch RE, Barkovich AJ. Cerebellar hemorrhage on magnetic resonance imaging in preterm newborns associated with abnormal neurologic outcome. J Pediatr. 2011;158(2):245-50. PMCID: 3010295.
222. O'Brien PC, Fleming TR. A multiple testing procedure for clinical trials. Biometrics. 1979;35(3):549-56.

1. PROTOCOL V5 (UPDATED 8/21/18)

PROTOCOL TABLE OF CONTENTS

SYNOPSIS 3

1. STUDY OBJECTIVES 7
   1.1 Primary Objective 7
   1.2 Secondary Objectives 7
2. BACKGROUND 8
   2.1 Rationale 8
   2.2 Supporting Data 10
3. STUDY DESIGN 12
4. SELECTION AND ENROLLMENT OF SUBJECTS 12
   4.1 Inclusion Criteria 12
   4.2 Exclusion Criteria 13
   4.3 Enrollment Procedures 14
5. STUDY INTERVENTIONS 15
   5.1 Interventions, Administration, and Duration 15
   5.2 Handling of Study Interventions 21
   5.3 Concomitant Interventions 23
   5.4 Adherence Assessment 24
6. CLINICAL AND LABORATORY EVALUATIONS 25
   6.1 Schedule of Evaluations 25
   6.2 Data to be Collected on Each Subject 27
   6.3 Timing of Evaluations 27
   6.4 Special Instructions and Definitions of Evaluations 39
7. MANAGEMENT OF ADVERSE EVENTS 43
8. CRITERIA FOR INTERVENTION DISCONTINUATION 43
9. STATISTICAL CONSIDERATIONS 44
   9.1 General Design Issues 44
   9.2 Outcomes 45
   9.3 Meeting Recruitment Targets 49
   9.4 Data Monitoring 50
   9.5 Formal Interim Analysis 55

10. DATA COLLECTION, SITE MONITORING, AND ADVERSE EVENT REPORTING 56

10.1 Records to be Kept 56
10.2 Role of Data Management 57
10.3 Quality Assurance 57
10.4 Adverse Event Reporting 61

11. HUMAN SUBJECTS 62

11.1 Institutional Review Board (IRB) Review and Informed Consent 62
11.2 Subject Confidentiality 62
11.3 Study Modification/Discontinuation 62

12. PUBLICATION OF RESEARCH FINDINGS 62

13. REFERENCES 64

SYNOPSIS

Study Title: Preterm Epo Neuroprotection Trial (PENUT Trial)

Objectives

Primary Objective:
Our primary goal is to test the hypothesis that early high dose recombinant human erythropoietin (Epo) treatment of preterm infants born between 24-0/7 and 27-6/7 weeks of gestation will improve long-term neurodevelopmental outcomes measured at 22-26 months (referred throughout the document also as "2 year(s)") corrected age. Specifically, we will determine whether Epo decreases the combined outcome of death or neurodevelopmental impairment (NDI) at 22-26 months corrected age.

Impairment is defined as the presence of any one of the following: cerebral palsy (CP), Bayley Scales of Infant and Toddler Development, 3rd Edition (Bayley-III) Motor Standard Score, Language or Cognitive Standard Score < 70 (severe, 2 SD below mean) or 85 (moderate, 1 SD below mean).[1, 2] CP will be identified by standardized neurologic exam and categorized as no CP, diplegic CP, hemiplegic CP, or quadriplegic CP. Severity of CP will be determined using the Gross Motor Function Classification System (GMFCS) developed by Palisano.[3, 4]

Secondary Objectives

- To determine whether there are risks to high dose Epo administration in extremely low gestational age neonates (ELGANs) by examining, in a blinded manner, Epo-related safety measures comparing infants receiving Epo with those given placebo. As part of this aim, Epo pharmacokinetics will be determined.
- To test whether Epo treatment decreases circulating inflammatory mediators and known biomarkers of brain injury over time.
- To compare brain structure (as measured by MRI) in Epo treatment and control groups at 36 weeks post menstrual age (PMA). MRI assessments will include documentation of intracranial hemorrhage (ICH), white matter injury (WMI) and hydrocephalus (HC), volume of total and deep gray matter, white matter and cerebellum, brain gyrification, inter-hemispheric distance, and tract-based spatial statistics (TBSS based on diffusion tensor imaging).
- As an exploratory aim, we will determine which of the above MRI measurements best predict neurodevelopment at 22-26 months corrected age.
- To determine the incidence of acute kidney injury (AKI) in this cohort of extremely low gestational age neonates (REPAIReD ancillary study).

Study Design
Prospective, multicenter, randomized, double blind, placebo controlled trial.

Figure 1 depicts an overview of the proposed trial.

Figure 1. Study Overview

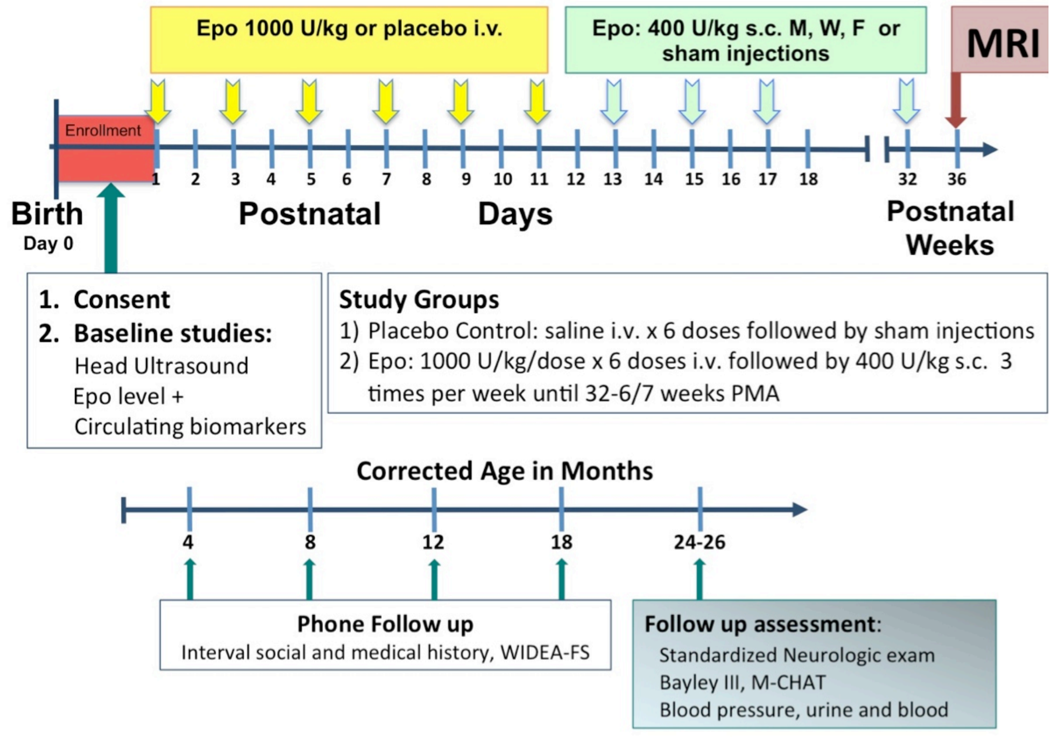

Interventions and Duration

Enrollment will occur prior to 24 hours of age. After enrollment but prior to the first dose of study drug, a cranial ultrasound (CUS) will be obtained to document whether an intracranial hemorrhage has occurred prior to dosing (while the goal will be to obtain the ultrasound prior to study drug dosing, a 6 hour grace period will be allowed). Baseline blood will be drawn to measure Epo concentration, inflammatory mediators and biomarkers of brain injury. Subjects will be randomized to Epo treatment or placebo by the data coordinating center (DCC). Each subject will be on the study (intervention + time off intervention) until completion of the follow-up visit at 22-26 months corrected age. After the in-person 2-year visit, with parental permission, PENUT subjects will continue to be followed at 6 month intervals by phone.

Study Drug

Drug Dosages and Routes of Administration

Subjects will be randomized to Epo treatment or placebo.

Initially, subject will receive Epo 1000 units /kg or placebo IV every 48 ± 2 hours x 6 doses (during the first 2 weeks of life when physiologic vulnerability is highest).

This will be followed by maintenance Epo 400 units/kg subcutaneously, or sham injection, Monday, Wednesday and Friday, during the period of oligodendrocyte vulnerability (to 32-6/7 weeks). Controls will not receive any injections. Maintenance study drug doses will begin on the day closest to completing the high dose series.

To maintain the blind in the maintenance portion of the treatment period, control infants will

have an empty syringe dispensed from pharmacy. Infants assigned to placebo will not undergo any subcutaneous maintenance injections. All blinded doses will be dispensed in an opaque bag with tamper resistant tape. Maintenance treatments will be administered behind blinds or curtains. After Epo injection or sham injection (no shot), all infants will have a 2” by 2” gauze placed over the “injection site”, and this will be covered and wrapped with gauze that is taped to itself, so no adhesive touches the child’s skin.

Group 1 (Epo):
1. High dose Epo (1000 units/kg) will be administered intravenously every 48 hours x 6 doses. Dosing will be based on birth weight. Study drug may be administered by the bedside nurse or a research nurse, given that the study drug appears identical.

2. Maintenance Epo (400 units/kg) will be administered by subcutaneous injection three times weekly. Dosing will be adjusted weekly after the subject has regained birth weight. A nurse who is not directly involved with the care of the patient, and who will maintain confidentiality will administer all treatments. A cohort of charge nurses, or General Clinical Research Center (GCRC) nurses would be ideal for this task.

Group 2 (Placebo):
1. Placebo (saline) will be given intravenously every 48 hours x 6 doses. The volume of the saline placebo will be equivalent to the volume which would be administered if the subject was receiving active drug. Study drug may be administered by the bedside nurse or a research nurse, given that the study drug appears identical.

2. Empty syringes (SHAM doses) will be delivered to infants in the control group however they will not undergo subcutaneous injections. Maintenance doses will be administered behind blinds or in a private room, and the “injection site” will be covered as described above. A nurse who is not directly involved with the care of the patient, and who will maintain confidentiality will administer all treatments. A cohort of charge nurses, or General Clinical Research Center (GCRC) nurses would be ideal for this task.

Exceptional situations:

- If a dose of study drug is unavoidably delayed or missed, the dose should be given as soon as possible. This should be recorded as a study deviation. The normal schedule of administration should then be resumed. At least 24 hours must separate the high dose study drug doses. This allows the plasma Epo concentration to return to baseline.

- If severe edema (anasarca) or other circumstances prohibit the use of subcutaneous injection, up to three doses of Epo or saline placebo may be given intravenously using the normal IV protocol. In this case, pharmacy must be notified so they can provide an appropriate study drug formulation for IV use (saline rather than an empty syringe used for sham injection). This should not be recorded as a deviation for up to 3 doses. Subsequent IV doses would be considered a deviation.

- If the baby’s IV is removed prior to completing the 6 intravenous study drug doses, the dose can be administered subcutaneously. This should be recorded as a study deviation.

Interventions that each subject will experience:

1. Cranial ultrasound prior to study drug dosing (all subjects)
2. Intravenous study drug treatment (Epo or placebo) for 6 doses, day 1-11 (all subjects)

3. *Epo group:* Subcutaneous Epo injections three times a week until 32-6/7 weeks PMA; *Control group:* Sham injections (no shots) three times a week until 32-6/7 weeks PMA
4. Iron supplementation (all infants)
5. Timed blood draws totaling 2 mL during the first 2 weeks of life (see Figure 3)
6. Ten timed, sequential urine collections from study entry to discharge
7. Brain MRI at 36 weeks PMA (220 subjects only)
8. Follow-up phone calls at 4, 8, 12, 18 months corrected age
9. Follow-up visit at 22-26 months corrected age will include: Blood pressure, weight, (optional) blood draw (0.5 mL), urine collection, standardized neurologic exam, GMFCS evaluation, Bayley Scales of Infant and Toddler Development, 3rd Edition, and M-CHAT-R questionnaire.

Sample Size and Population

In order to maintain 752 (376 per arm) who survive and are followed at 22 to 26 months corrected age, we will enroll a total of 940 subjects. This number accounts for attrition due to death, loss to follow-up, and accounts for multiple births. The Vermont Oxford Network reports that 25% of ELBW infants are from multiple births and a variance inflation factor would therefore involve the expected cluster size (1.25) and the within-cluster correlation (ρ) and equal: 1 + (1.25-1) ρ. If the within sib-ship correlation is 0.50 then the variance inflation factor (VIF) would be 1.125 implying a 12.5% (n=94) increase in target sample size necessary to retain appropriate power. Due to withdrawal or attrition by 2 years, we anticipate losing 12.5% of the effective sample size of 752 and will therefore increase the overall sample by an additional 94 subjects for a total enrollment target of n=940 subjects.

Qualified infants less than 24 hours of age of both sexes and all races will be entered into the study if their parents or legal guardians consent.

Randomization will be stratified by site, multiple births, and by gestational age: 24-0/7 to 25-6/7 and 26-0/7 to 27-6/7. We will use block randomization within site using variable blocks of size 4, 6, 8 and 10 subjects. Using block randomization ensures that equal numbers of subjects are randomized to the intervention and control arm and that the two groups are balanced at period enrollment intervals. For multiple births (twins, triplets) all infants will be randomized into the same treatment group (e.g. effective randomization of the mother).

Table 1. Frequently Used Abbreviations:

BDNF	Brain derived neurotrophic factor	MDI	Mental developmental index
BPD	Bronchopulmonary dysplasia	NDI	Neurodevelopmental impairment
CCC	Clinical coordinating center	NEC	Necrotizing enterocolitis
CP	Cerebral palsy	NICU	Neonatal intensive care unit
DCC	Data coordinating center	PDA	Patent ductus arteriosus
DEHSI	Diffuse excessive high signal intensity	PDI	Psychomotor developmental index
ELGANs	Extremely low gestational age neonates	PMA	Postmenstrual age
Epo	Erythropoietin	PVL	Periventricular leukomalacia
EpoR	Epo receptor	ROP	Retinopathy of prematurity
GMFCS	Gross motor function classification system	SOP	Standard operating procedure
HC	Hydrocephalus	SC	Subcutaneous
IV	Intravenous	TBSS	Tract based spatial statistics

ICH	Intracranial hemorrhage	VON	Vermont Oxford Network
M-CHAT-R	Modified Checklist for Autism in Toddlers	WMI	White matter injury

1. STUDY OBJECTIVES

1.1 Primary Objective

Hypothesis 1. Epo treatment from 24 hours to 32-6/7 weeks PMA of ELGANs will safely decrease the combined outcome of death or NDI from 40% to 30% measured at two years corrected age.

Objective 1. Our primary goal is to test the hypothesis that early high dose recombinant human erythropoietin (Epo) treatment of preterm infants born between 24-0/7 and 27-6/7 weeks of gestation will improve long-term neurodevelopmental outcomes measured at 22-26 months corrected age.

1.2 Secondary Objectives

Hypothesis 2. High dose Epo will be safe to administer from birth to 32-6/7 weeks of gestation in this population of ELGANs.

Objective 2. To determine whether there are excess adverse events in Epo treated ELGANs compared to control infants. Three time periods will be considered: 1. Safety during the treatment period; 2. Safety during the initial hospitalization; and 3. Long term outcomes. Timed Epo concentrations will be measured to monitor for drug accumulation and safety. A Population Pharmacokinetic Analysis will be done using these data.

Hypothesis 3. Therapeutic Epo administration during the period of oligodendrocyte vulnerability (24-32 weeks of gestation) will promote normal brain development by increasing brain derived neurotrophic factor (BDNF), decreasing circulating inflammatory mediators, and thereby decreasing biomarkers of brain injury.

Objective 3. To test whether Epo treatment changes sequential measures of BDNF, circulating inflammatory mediators and known biomarkers of brain injury in preterm infants.

Hypothesis 4. Epo treatment will improve brain development as assessed by MRI at 36 weeks PMA.

Objective 4. To compare brain structure (as measured by MRI) in Epo treatment and control groups at 36 weeks PMA. MRI assessments will include documentation of intracranial hemorrhage (ICH), white matter injury (WMI) and hydrocephalus (HC), volume of total and deep gray matter, white matter and cerebellum, brain gyrification, inter-hemispheric distance, and tract-based spatial statistics (TBSS) based on diffusion tensor imaging. As an exploratory aim, we will determine which of the above MRI measurements best predict neurodevelopment (CP, cognitive and motor scales) at 22-26 months corrected age.

Hypothesis 5. Epo treatment may affect kidney development and incidence of acute kidney injury.

Objective 5. To establish the incidence of acute kidney injury in ELGANs, and to determine whether Epo treatment influences this.

2. BACKGROUND

2.1 Rationale

Study Population. ELGANS are at high risk of death or NDI. In fact, of 9,575 ELGANs born between 2003 and 2007 and admitted to the NICU, death or NDI occurred in 91%, 80%, 66% and 56% of those born at 24, 25, 26 and 27 weeks gestation, respectively.[5] These sobering statistics do not include infants that died before admission to a NICU, or those who died within 12 hours of admission. Major morbidities, which include CP, deafness, blindness, and mental disabilities, are present in 50% of surviving extremely preterm infants at school age.[6-19] Long-term follow-up studies are now increasingly identifying behavioral dysfunctions such as attention deficit disorder and autism spectrum disorder.[20-24] Sequelae of extreme prematurity are a tremendous burden to the individuals, their families, and to our health care system, accounting for nearly half of the health care dollars spent on newborn care. Clearly, a neuroprotective intervention that improved outcomes for ELGANs would be profoundly beneficial both to the individual, the family and to society.[25, 26] We have chosen to study ELGANs born between 24-0/7 and 27-6/7 weeks of gestation because of their high likelihood of poor outcome, and the absence of therapeutic interventions to improve outcome. Because these infants are at such high risk of death or impairment, a strategy of prophylactic intervention is reasonable as there is great potential for benefit.[25, 26]

Restrictions: Babies born at 23-0/7 to 23-6/7 will be excluded from this study because their mortality and morbidity is too high. At this time, best estimates of death rates are 74%, and unimpaired survival rates are 1%.[5, 19] More mature babies are also excluded because their risk of poor outcomes is lower, thus prophylactic use of an unproven therapy is not indicated.

Intervention Regimen. Study infants in the Epo group will receive 6 IV doses of 1000 U/kg during the first 2 weeks of life when physiologic vulnerability is highest. This will be followed by maintenance dosing of 400 U/kg SC 3 times per week during the period of oligodendrocyte vulnerability (to 32-6/7 wks).

Dose Justification. The Epo dose, and duration of therapy chosen for this study is based upon available preclinical and clinical data for Epo neuroprotection.[27] In rodent and ovine models of neonatal brain injury, Epo doses of 1000-5000 U/kg/dose result in sustained neuroprotection, improving both short and long-term structure and function.[28-31] Higher doses are needed for neuroprotection due to the low percentage of circulating Epo that crosses the blood brain barrier.[32]

Although doses as high as 3000 U/kg/dose are being tested in preterm infants without apparent adverse effects,[33] preclinical data suggest that Epo neuroprotection has a U-shaped dosing curve, with too little or too much Epo resulting in diminished efficacy.[29, 34] In rats, repeated doses of 1000-5000 U/kg show optimal neuroprotection. To

Figure 2. Epo concentrations in human preterms compared with rat pups given 5000 U/kg s.c. or i.p.

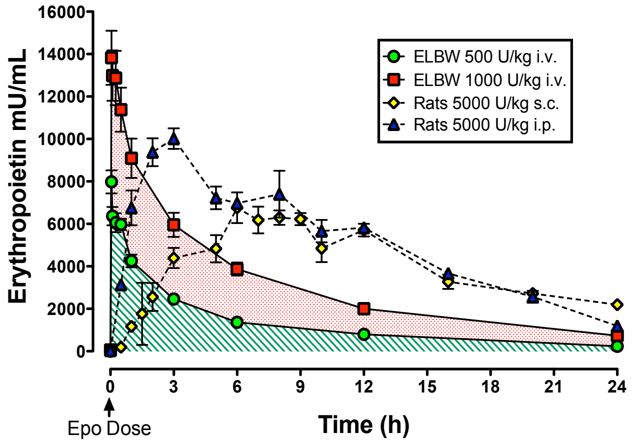

estimate how neuroprotective Epo doses in rat pups relate to human pharmacokinetics, plasma Epo concentrations were measured in extremely low birth weight infants (< 1000 g birth weight) after 500, 1000, and 2500 U/kg/dose.[35] Nonlinear kinetics were noted, consistent with previous studies in neonates.[36] In these infants, IV administration of 500 and 1000 U/kg resulted in similar peak concentrations but faster clearance than were achieved in rat pups after 5000 U/kg (Figure 2). Table 2 shows a comparison of pharmacokinetic indices in neonatal rats and humans. Doses of 1000 U/kg Epo resulted in area under the curve (AUC) measurements most similar to the most protective dose in rats.[29] The 500 U/kg dose fell short (one third to one quarter the protective AUC), while 2500 U/kg was close to three times the optimal dose in rats. Minimum steady-state concentrations (mean = 576 mU/ml) were produced using the 1000 U/kg/dose.

Table 2. Epo Pharmacokinetics

	**Neonatal Rodents[37]**		**Preterm Infants < 1000 gm[35]**		
Epo Dose	5000 U/kg s.c.	5000 U/kg i.p.	500 U/kg	1000 U/kg	2500 U/kg
AUC (U*h)/L	117,677	140,331	31,412 ± 2780	81,498 ± 7067	317,881±22941
Cmax (U/L)	6,224	10,015	8078 ± 538	14017 ± 1293	46467 ± 2987
T1/2 Half-life (h)	8.4	6.7	5.4 ± 0.6	7.1 ± 0.7	8.7 ± 1.4

What constitutes clinical significance? Clinically significant studies are those that improve clinical practice and patient outcomes. Changes to clinical practice can occur slowly or rapidly depending on a number of factors: importance of the results, the size of the studies, risk of the therapy, and endorsement of medical associations such as the AAP. Ibrahim et al. performed a 13-item web-based questionnaire asking neonatologists what would convince them to adopt a new therapy in infants < 28 weeks of gestation. The survey assumed no adverse results of treatment. Responses are shown in Table 3. We also have examples of changes in clinical practice based on published trials. Prenatal steroids reduce the risk of death or morbidity in preterm infants < 32 weeks gestation with a risk reduction of over 40%.[38] Despite this known benefit, change in clinical practice was slow, and required endorsement from ACOG. In contrast, therapeutic hypothermia for neonatal encephalopathy has been the subject of intense study, with multicenter randomized controlled trials published in 2005-2009.[39-41] A meta-analysis of these studies reporting on 767 infants showed benefits of cooling with a significant reduction in death or NDI at 18 months: typical Risk Ratio, 0.81 (95% Confidence Intervals, 0.71, 0.93), P=0.002. The number needed to treat (NNT) ranged from 7-9 to prevent death or NDI. Cooling for neonatal encephalopathy has rapidly become the standard of care. Animal studies of neonatal Epo neuroprotection using appropriate dosing have shown improvement of 49 to 79%.[27] Early clinical trials have shown safety and preliminary benefit. Neonatologists are comfortable using this drug for erythropoiesis. We expect that Epo treatment will improve NDI-free survival compared to that seen in the ELGAN and NICHD trials. We expect that this benefit will translate into shorter and less complicated hospital stays and better neurodevelopmental outcomes. This outcome of the trial would represent an important advance in the care of ELGANs that could change the standard of care for high-risk infants. If Epo treatment has no demonstrable benefit, or if adverse effects are observed, this information will also be useful in the field because Epo is currently used

**Table 3**. Survey of Neonatologists. "Would you change practice if a study showed treatment X…?"	% would change N=226
Reduced # Bayley scores < 80 by 25%	41
Reduced # Bayley scores < 80 by 50%	66
Improved Bayley scores by 10 points	56
Decreased CP by 25%	50
Decreased CP by 50%	81
Study size 200 per arm	29
Study size 400 per arm	64

anecdotally in many NICUs, without trial-based evidence, for some severely ill infants. The results of the PENUT trial will be of particular interest to pediatricians trained in neonatal-perinatal medicine. This group of practitioners is largely centered in academic or medical centers and they attend several meetings each year where new information about treatment can be presented. The PENUT trial results will be presented at regional Pediatric Research Society meetings such as the Western and Eastern Society for Pediatric Research, and at national and international meetings including the Pediatric Academic Societies combined meetings, the European Society for Pediatric Research meeting, the American Academy of Pediatrics meetings, and at other venues such as "Hot Topics in Neonatology".

Clinical Impact. Mortality of extremely preterm infants has decreased,[42, 43] but morbidity in survivors remains high due in part to increased survival of sicker infants. There remains an enormous burden of both medical and neurodevelopmental impairment in these children. Using the birth statistics for the US in 2009, we anticipate the magnitude of Epo treatment to translate as follows: with Epo treatment, the annual burden of death or severe NDI will decrease from 40% (12,226 babies) to 30% (9,170 babies, primary outcome), and that death plus moderate or severe NDI will decrease from 60% (18,340 babies) to 40% (12,226 babies, secondary outcome). We anticipate this change will derive primarily from improved neurodevelopmental outcomes. If Epo is proven to safely reduce combined morbidity and mortality of ELGANs, we anticipate a shift in neonatology practice that would improve the lives of babies and their families, and decrease the cost of healthcare.

2.2 Supporting Data

Vulnerabilities of the preterm brain. ELGANs are born during the second trimester when the fetal brain is rapidly increasing in size, shape and complexity.[44-46] Proper brain development is vulnerable to interruption by hypoxia-ischemia, oxidant stress, inflammation, and excitotoxicity, as evidenced by structural, biochemical, and cell-specific injury.[47-53] Pre-oligodendrocytes, which emerge and mature between 24 and 32 weeks of development, are particularly susceptible to injury, resulting in the WMI characteristic of preterm infants.[47, 54-56] Although the transition from fetal to early postnatal life is the period of greatest vulnerability,[57] ELGANs remain at risk for brain injury throughout the period of oligodendrocyte development.

Epo Neuroprotection. Epo has anti-inflammatory, anti-excitotoxic,[58] anti-oxidant,[59] and anti-apoptotic effects on neurons and oligodendrocytes, and promotes neurogenesis and angiogenesis, which are essential for injury repair and normal neurodevelopment. Epo effects are dose-dependent, and multiple doses are more effective than single doses.[28, 29, 60] Epo reduces neuronal loss and learning impairment following brain injury,[61, 62] and even when initiated as late as 48-72 hours after injury, there is evidence of improved behavioral outcomes, enhanced neurogenesis, increased axonal sprouting, and reduced WMI.[63, 64]

Protective effects. Perinatal inflammation (chorioamnionitis, necrotizing enterocolitis (NEC), or sepsis) is associated with increased risk of NDI.[13, 65, 66] Microglial activation[67] and increased cytokine expression, particularly TNF-α, interleukin (IL)-6, and IL-8, have been associated with brain injury in preterm infants[68, 69] and in animal models of neonatal brain injury.[70] Epo has demonstrated anti-inflammatory effects, which may contribute to neuroprotection in the scenario of preterm birth and increased inflammatory activity.[71-77]

White matter injury. WMI is a common brain injury affecting preterm infants.[48, 52, 55] Epo decreases WMI in adult and neonatal animal models of brain injury[30, 63, 78-83] and maybe in

humans.[84] Mechanisms for this may be Epo protection of vulnerable preoligodendrocytes: functional Epo receptors are expressed by immature oligodendrocytes; Epo promotes the proliferation, maturation and differentiation these cells,[85] and protects them from injury induced by interferon-γ, LPS, and hypoxic-ischemia.[63, 86, 87]
Apoptosis. Neurons in the developing brain are more likely than adult neurons to undergo apoptosis if exposed to injurious stimuli,[88, 89] and the anti-apoptotic properties of Epo may protect these vulnerable neurons.[29, 90, 91]

Repair. Epo stimulates growth factors required for normal brain growth such as brain-derived neurotrophic factor (BDNF) and glial cell derived neurotrophic factor (GDNF).[92, 93] Epo enhances neurogenesis,[28, 60, 91, 93-96] angiogenesis, repair and plasticity, thus providing long lasting neuroprotective and trophic effects.[60, 64, 94, 97-100]

Reactive Iron. Iron is highly reactive and normally sequestered by transport proteins. Unbound iron produces free radicals and subsequent oxidative injury. Preterm infants have measurable free iron, which increases after transfusions of red blood cells or during metabolic instability such as sepsis.[101-104] In our phase I/II study of Epo administration to extremely low birth weight infants we observed a transient increase in reticulocytosis, indicating an increase in iron utilization.[105] Epo may contribute to neuroprotection by decreasing free iron.

Molecular mechanisms of Epo neuroprotection. EpoR are present on neuron progenitor cells,[93] neurons,[106] astrocytes,[85] oligodendrocytes,[107] microglia,[108] endothelial cells[93] and erythrocyte progenitors. Epo has direct neuroprotective effects via EpoR binding: Epo-bound receptors dimerize to activate anti-apoptotic pathways via phosphorylation of JAK2, phosphorylation and activation of MAPK, ERK1/2, as well as the PI3K/Akt pathway and STAT5, which are critical in cell survival.[90, 108] Epo also functions through indirect effects, increasing iron utilization by increasing erythropoiesis, and by decreasing inflammation[73, 76] and oxidative injury.[109, 110]

Translational trials of neonatal Epo neuroprotection are in progress. Epo improved neurodevelopmental outcomes in two trials of term neonates with hypoxic-ischemic brain injury.[111, 112] Retrospective studies of cohorts of preterm infants that received Epo for erythropoiesis compared to controls show improved outcomes.[113-115] Two preliminary reports of preterm infants treated prospectively also show benefit:

1) Preterm infants 500 to 1250 g treated with Epo (400 U/kg 3x/week) from birth to 35 weeks PMA had an average cumulative cognitive score 10 points higher than placebo/controls (98±14 Epo vs. 88±14 for controls). Epo recipients also performed statistically better than controls on object permanence testing;[116]

2) A phase III trial is ongoing in Switzerland to test the safety and neuroprotective efficacy of 3000 U/kg/dose x 3 doses given in the first 3 days after birth to neonates (<1500 g, < 32 weeks PMA). This group has presented preliminary data in abstract form on 100 infants showing that such dosing is safe, and that the Epo-treated group showed less WMI by MRI;[84]

3) Follow-up of the infants studied in our phase I/II trial[35] shows that Epo treatment correlated with improvement of cognitive ($R= .22$, $p < 0.05$) and motor ($R = .15$, $p < 0.05$) scores.[117]

Known and potential risks of intervention: In adults, complications of prolonged Epo treatment include polycythemia, seizures, hypertension, stroke, myocardial infarction, congestive heart failure, tumor progression, and shortened time to death. None of these

adverse effects have been reported in Epo-treated neonates in over 3000 patients enrolled in randomized controlled trials.[118] Epo trials in neonates for the purposes of testing its erythropoietic effect have shown it to be a safe drug for use in this population. There is robust data from preclinical animal work showing that Epo, when used at optimal doses (1000-5000 U/kg), shows short and long term improvement in brain injury that approximates 50-80%, and no safety issues have been discovered. Preterm infants administered Epo may be at increased risk of ROP and other as yet unknown complications.

Federal oversight. This study of high dose Epo for the purposes of neuroprotection of preterm infants is registered with the FDA (IND # 12656). Dr. Juul, University of Washington, Seattle WA is the holder of the IND. The trial has been registered with clinicaltrials.gov and has been assigned Identifier Number NCT01378273.

3. STUDY DESIGN

Study Overview. This is a randomized, placebo controlled, double blind study of Epo neuroprotection in an ELGAN population. Figure 1 provides an overview of the study. 940 patients will be enrolled at 16-18 sites across the country in order to evaluate 752 at 22-26 months corrected age. Enrollment and initial treatment with study drug will occur by 24 hours after birth. Subjects will be randomized to either Epo treatment or placebo, and treatment will continue until 32-6/7 weeks PMA. Short term, intermediate and long term safety measures will be determined by comparing Epo-treated and control infants. Mechanisms of Epo neuroprotection and potential biomarkers of outcome will be sought by measuring sequential inflammatory cytokines and markers of brain injury. In a subset of subjects, a brain MRI will be done at 36 weeks PMA to determine whether Epo treatment preserves brain growth and decreases injury. After discharge from the hospital, phone contact will be made at 4 month intervals. Data will be collected on interval medical history and functional status. Face to face follow-up will occur at two years corrected age, at which time standardized neurodevelopmental assessments will be made. The primary outcome is death or severe NDI at 22-26 months corrected age, with a secondary outcome of death, severe or moderate NDI.

4. SELECTION AND ENROLLMENT OF SUBJECTS

4.1 Inclusion Criteria

- NICU Inpatients between 24-0/7 and 27-6/7 weeks of gestation
- 24 hours of age or less
- Arterial or venous access
- Parental consent

When calculating gestational age, the following hierarchy will be used in order of accuracy:
1. Gestational age by in vitro fertilization
2. Gestational age by first trimester assessment (0 to 14-0/7 weeks)
3. Gestational age by second trimester assessment (up to 28-0/7 weeks)
4. Last menstrual period (LMP)
5. Newborn maturational assessment

We estimate, based on the ELGAN study, that 62% of subjects will have the most accurate dating based on #1 or #2, that 29% will have dating based on second trimester fetal ultrasound,

7% by menstrual dating, and only 1% by postnatal physical exam.

Postnatal age will be calculated based on time and date of birth.

Arterial or venous access is considered to be a functioning peripheral intravenous catheter, a peripherally inserted central catheter (PICC), an umbilical venous (UVC) or arterial catheter (UAC). One of these catheters must be available for the administration of study drug. It is preferable that the patient also have a catheter suitable for phlebotomy, although that is not an absolute entry criterion for the study.

To be admitted into this study, the legal guardian of the patient must give written informed consent. Each site will obtain IRB approval for the study, and will have IRB approved consent forms available in English and a second language of their choosing. A translator will be provided for non-English speaking individuals as needed. If a translator is not available, the family will not be approached.

4.2 Exclusion Criteria

- Known major life-threatening anomalies (e.g. fetal diagnosis of brain, cardiac, or renal malformations
- Known or suspected chromosomal anomalies
- Severe hematologic crises such as clinically evident disseminated intravascular coagulopathy, twin-twin transfusion such that 1 twin is not eligible due to polycythemia or hydrops
- Polycythemia (hematocrit > 65%)
- Hydrops fetalis
- Known congenital infection such as toxoplasmosis, CMV, rubella or syphilis.
- Prior administration of erythropoietin to the baby
- Infant is likely to die due to severity of illness or withdrawal of support is being considered prior to enrollment

As part of standard care of an extremely preterm infant, a CBC will be obtained within the first 24 hours of life. If the HCT is > 65%, the patient will not be eligible for the study. If the PLT count is < 50K, or if there are clinical signs of bleeding, a coagulation panel will be checked to determine whether there are signs of disseminated intravascular coagulopathy. If this condition exists, the patient is not eligible for the study.

All patients eligible for this study will be hospitalized in the neonatal intensive care unit (NICU). Many concurrent illnesses are expected for this population of extremely preterm infants. These include, but are not limited to: ICH, periventricular leukomalacia (PVL), hydrocephalus (HC), respiratory distress syndrome (RDS), bronchopulmonary dysplasia (BPD), hypotension, patent ductus arteriosus (PDA), NEC, early and late onset sepsis, and retinopathy of prematurity (ROP). **None of these will exclude a child from study entry, but all will be considered in the safety evaluation of Epo.**

4.3 Enrollment Procedures.

4.3.1 Screening. Admissions to the antepartum unit and to the NICU at each site will be screened at least daily for admissions that are potentially eligible for the trial. This will be done

by the research coordinator or their designee. Once identified, the Attending health care provider will seek parental agreement for an investigator to meet and discuss the study. If the mother is interested in learning more about the study, the attending health care provider can notify the research coordinator or investigator so they can discuss the study with the parent and seek consent. When possible, and if there is IRB approval for this, consent will be obtained prior to delivery. Many mothers of preterm infants are admitted to an antepartum service for bed rest, fluids and observation. They are not acutely laboring, and the stress of imminent delivery is less acute. If permitted by the local IRB, these mothers will be approached during a time when they are able to fully evaluate the study and have opportunities for reflection and questions.

4.3.2 A site screening log will be maintained to document patients screened, reasons for ineligibility, and reasons for nonparticipation of eligible subjects. This document will be maintained by the site research coordinator or their designee and will contain the following information: screening number, screening date, patient surname, medical record number, date of birth, gender, eligibility (yes/no), whether they were enrolled, and if not enrolled, the reason why. If a subject is enrolled, document that consent was obtained, and the study ID number.

In addition, selected site screening information will be entered electronically and maintained in the PENUT Portal data management system. Entered data will not contain patient names or medical record numbers. This cumulative log will be reviewed at regular intervals to determine enrollment and reasons for non-enrollment. This information will be used to consider whether other recruitment methods should be used.

4.3.3 Consent procedures. Antenatal consent will be obtained when possible. The study investigator will obtain permission from the Maternal Fetal Medicine Attending to approach the mother to discuss the study. If consent is obtained prenatally, this will be re-confirmed at the time of the child's birth if required by the local IRB. Postnatally, permission to approach the family will be obtained from the Attending Neonatologist. The Attending health care provider will seek parental agreement for an investigator to meet and discuss the study. If the parents are interested, the study investigator will discuss the study with family and seek consent in person. The consenting legal guardian will receive a copy of the consent form to review, and once signed, will be given a copy to keep. Ideally, if the Attending physician is also a study investigator, an alternate study investigator, or their designee, should obtain consent, so as to avoid the appearance of coercion. If this is not required by the site IRB, an investigator who is also the Attending physician may obtain consent. Investigators will only approach family after infant's attending heath care provider gives permission and family indicates that they are interested in further information about the study. No alteration in care will otherwise occur. The attending neonatologist and family can withdraw child from study at any time. If permissible by the site IRB, phone consent may be obtained, but must be reaffirmed with the family when they are present. Investigator should retain original signed document. The investigator will obtain consent in a room which ensures the privacy of the family, and which is free of potential coercive influences. Consent for participation must be obtained before the baby is 24 hours of age. Consent for participation the continued phone contacts may be obtained at the 2-year in-person visit, or by phone.

A certified interpreter will be provided if a family has limited or no English speaking abilities. They will review the consent form with the family, and interpret the verbal explanation of the study during the discussion between the Investigator and the family members. If individual sites have a large population of non-English speakers, consent forms will be translated into the appropriate languages. If an interpreter is not available in a timely manner, the family will not be approached.

The parents of the research participants will be given opportunity to review the study both verbally and in writing. They will be given opportunity to ask questions of the investigator prior to giving consent. A sample consent form is shown in Appendix 1.

4.3.4 Randomization procedures. Site-specific study personnel associated with the PENUT Trial will screen and randomize patients by logging in to the PENUT Portal at www.penut-trial.org. Once it is confirmed that a patient meets inclusion criteria, does not have any exclusion criteria, and that consent and HIPAA forms have been signed, the DCC web page will generate the patient study ID for trial affiliated staff to provide to the site pharmacy at the time of study drug ordering. The site pharmacy will then look up the study ID in their binder and dispense the blinded study drug as requested.

5. STUDY INTERVENTIONS

5.1 Interventions, Administration, and Duration

All patients will be treated in the NICU. Each subject will be randomized to one of two groups after establishing eligibility and obtaining signed consent for study participation and HIPAA compliance. Interventions/Treatments will be as outlined in Table 4. Study drug will be administered according to the schedule shown in Figure 3.

Cranial Ultrasound. After written consent is obtained, but before administration of study drug, a cranial ultrasound will be done to document whether an intracranial hemorrhage has occurred. Results of this examination will not affect clinical care, eligibility, or randomization for the study, but will be used as part of the safety analysis. Dr. Manjiri Dighe, a UW radiologist who will be blinded to the treatment groups and any clinical information about the child, will read the cranial ultrasounds. If a cranial ultrasound is indicated based on the patient's condition, it will be ordered by the caregiver on site and read by the site radiologist as part of clinical care. For more details please see the Cranial Ultrasound guidelines, Appendix 6.

Phlebotomy: Subjects will have four timed blood samples drawn during the first two weeks of life (see Figure 3). The first blood draw may be obtained from cord blood, if available. At the two year follow-up visit, an additional (optional) blood sample of 0.5 mL will be drawn.

Figure 3.

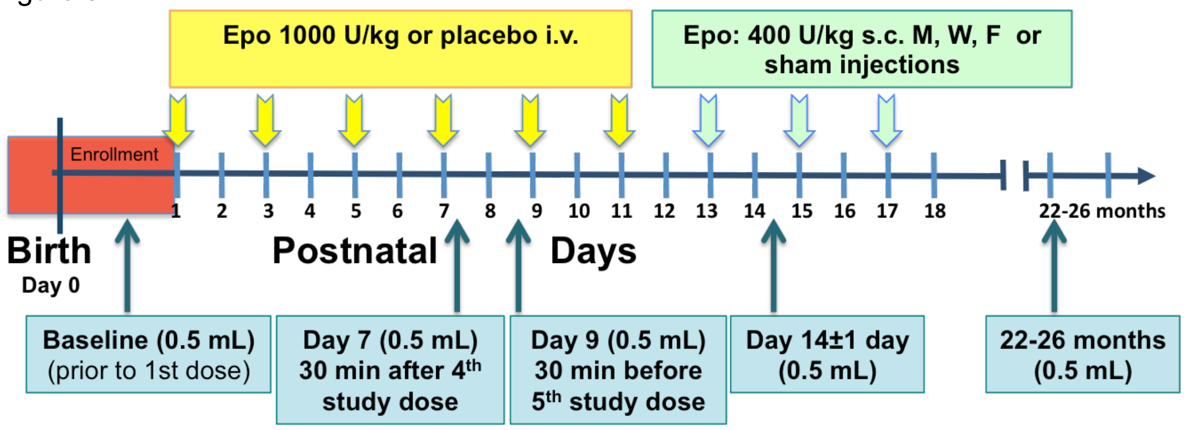

Cerebral Spinal Fluid (CSF): If a clinically-indicated spinal tap is ordered for an enrolled subject (e.g. to rule out meningitis), then residual CSF may be available for the PENUT study to collect. In that event, we request 0.15 mL CSF be collected and frozen for measurement of CSF Epo concentration in any infant who undergoes a diagnostic lumbar puncture during the initial hospitalization. If residual CSF is available for the PENUT study, then a small systemic blood sample (0.15 mL) will ideally also be collected within one hour of when the CSF sample is collected and processed to plasma as described above. These biologic specimens will be sent to the UW CCC lab for analysis. Diagnostic spinal taps provide a serendipitous opportunity to correlate circulating Epo/cytokine levels with CSF levels. If significantly elevated Epo concentrations are present, further analysis, either by Western blotting or MALDI TOF mass spectrometric measurement will be considered. If no blood can be obtained, CSF should still be collected and sent to the CCC.

Brain MRI: A subset of 220 subjects will undergo a brain MRI at 36 weeks PMA. Prior to scanning, infants will be fed and swaddled. If needed, the patient can be immobilized using a MedVac Infant Vacuum Immobilization Bag (contour Fabricators, Inc., Fenton, Michigan), or comparable device as per usual site practices for neonatal MRI scans.[119] Per usual site practice, ear plugs, and/or ear covers [MiniMuffs® (Natus Medical Inc., San Carlos CA)] will provide noise reduction. Exams will be rescheduled if the child cannot be quieted sufficiently for the exam. Subjects will be monitored with an MRI-compatible pulse oximeter. A caregiver trained in MRI procedures and neonatal resuscitation will be in attendance.

Table 4. Phase III Trial

Time Line	Procedure	Sites	Control	Epo
0-24 hours from birth	Consent	All	All	All
Consent to 24 h of age	Randomization	All	All	All
Consent to 24 h of age	Cranial Ultrasound	All	All	All
Consent to 24 h of age	Baseline blood (0.5 mL) and urine sample (1 -2 mL)	All	All	All
Study day 1	Epo dose 1 IV	All	Placebo	Epo 1000 U/kg
Study day 3	Epo dose 2 IV	All	Placebo	Epo 1000 U/kg
Study day 5	Epo dose 3 IV	All	Placebo	Epo 1000 U/kg
Study day 3-5	Urine sample (1-2 mL)	All	All	All
Study day 7	Epo dose 4 IV	All	Placebo	Epo 1000 U/kg
Study day 7	Blood draw #2 (0.5 mL)	All	All	All
Study day 8	Begin iron supplementation per guidelines	All	All	All
Study day 7-9	Urine sample (1-2 mL)	All	All	All
Study day 9	Blood draw #3 (0.5 mL)	All	All	All

Study day 9	Epo dose 5 IV	All	Placebo	Epo 1000 U/kg
Study day 11	Epo dose 6 IV	All	Placebo	Epo 1000 U/kg
First Monday, Wednesday or Friday following IV doses	Begin maintenance Epo vs. sham injections (nothing)	All	No injection	Epo 400 U/kg
Study day 14 ± 1 day	Blood draw #4 (0.5 mL), Urine sample (1-2 mL)	All	All	All
Study week 3, 4, 6, 8, 10, 12	Urine samples (1-2 mL)	All	All	All
Continue study drug until 32-6/7 weeks PMA	Maintenance Epo or sham injections (no shots)	All	No injection	Epo 400 U/kg
36-0/7 to 36-6/7 weeks	MRI	MRI sites	110 per arm	110 per arm
Discharge	Physical exam, Urine sample and questionnaire	All	All	All
4 m corrected age	Phone contact	All	All	All
8 m corrected age	Phone contact	All	All	All
12 m corrected age	Phone contact	All	All	All
18 m corrected age	Phone contact	All	All	All
22-26 m corrected age	Bayley III, Neuro exam, M-CHAT-R	All	All	All
22-26 m corrected age	Weight, blood pressure, urine collection (1-2 mL), and (optional) blood draw #5 (0.5 mL)	All	All	All
30 m chronological age	Phone contact	All	All	All
36 m chronological age	Phone contact	All	All	All
42 m chronological age	Phone contact	All	All	All
48 m chronological age	Phone contact	All	All	All
52 m chronological age	Phone contact	All	All	All
60 m chronological age	Phone contact	All	All	All

Epo Risks: In adults, complications of prolonged Epo treatment include polycythemia, seizures, hypertension, stroke, myocardial infarction, congestive heart failure, tumor progression, and shortened time to death.

None of these adverse effects have been reported in Epo-treated neonates. No prospective studies of neonatal Epo treatment have reported group differences in the incidence of neonatal morbidities, including ICH, ROP, NEC, BPD, or late onset sepsis,[120] but a Cochrane review reported an increased risk of ROP with early treatment.[121] This report was recently revised, with no statistical increase in ROP noted for early or late Epo administration.[122] One retrospective report also described an increase in cutaneous hemangiomas in Epo-treated preterm infants.[123]

In the 2 pilot studies of high dose Epo administered to preterm infants done, one at the University of Washington Medical Center and one in Switzerland, no increased risk of any complications were noted in a total of 60 treated patients, including ROP.[33, 35] In the UW study, we observed 5 cases of severe ROP in 23 surviving controls compared to 6 of 24 Epo treated infants.[35]

Safety data from a prospective randomized controlled phase II trial of high dose Epo in Switzerland (ClinicalTrials.gov: NCT00413946), in which 3000 U/kg daily was given to infants 26-0/7 to 31-6/7 weeks of gestation at 3, 12-18 and 36-42 hours after birth (N=229 treated, 214 controls) has been reassuring. There were no relevant differences between the groups for short-term outcomes including mortality, ROP, IVH, sepsis, NEC, and BPD.[124] Higher hematocrit was noted at day 7-10 in the treated group. Improved white matter was also noted at term corrected age by MRI.[125, 126]
Three updated Cochrane reviews have been published reviewing the safety and efficacy of Epo for erythropoietic purposes,[122, 127, 128] and another meta-analysis focused specifically on Epo and ROP.[129] Conclusions from these reviews are as follows: Epo, whether used early or late, reduces the use of one or more RBC transfusions, and the number of RBC transfusions per infant. No increase in any of the complications of prematurity are noted, i.e. use of erythropoietic doses of Epo are safe in preterm infants. The safety of higher, neuroprotective doses has not yet been systematically evaluated in large population studies.

Epo is a potent erythropoietic growth factor. Thus, high doses of Epo given for neuroprotective treatment might be expected to increase erythropoiesis, and possibly megakaryocytopoiesis. In neonatal rats, there is a transient increase in hematocrit following high-dose Epo,[62] but in preterm infants, while 3 doses of high dose Epo increased reticulocytosis, they did not affect hematocrit, likely due to early phlebotomy losses.[35] In term infants with neonatal encephalopathy, after up to 6 doses of high dose Epo, hematocrit fell an average of 14%, reflecting phlebotomy losses in these sick infants.[130] The effect of brief treatments of high-dose Epo on iron balance is not known. Prolonged Epo treatment in neonates must be accompanied by iron supplementation, and does improve erythropoiesis.[116] This is, in fact, currently the primary indication for Epo use in neonates. Dosing of 400 U/kg/dose three times a week has been shown to decrease (but not eliminate) need for blood transfusions.[131]

1) Safety during the high dose treatment period. Serious adverse events (SAEs) specific to Epo will be defined as any of the following during the treatment period:

- Hypertension: subject required prolonged antihypertensive therapy (> 1 month) and/or will be discharged on medication
- Polycythemia (central hematocrit > 65%, or hematocrit increase ≥ 15% not due to red blood cell transfusion)
- Major venous or arterial thrombosis (involving a major vessel **not related to an infusion line,** requiring anticoagulation, or symptomatic such as causing superior vena cava syndrome)

Serious adverse events most likely related to prematurity will include

- Severe pulmonary hemorrhage (severe, e.g., need for increased respiratory support, positive pressure. *Not* pink tinged secretions with suctioning).
- Necrotizing enterocolitis (stage 2B or 3)
- Severe sepsis: Blood culture-proven bacterial or fungal sepsis requiring blood pressure support or significant new respiratory support.
- Intracranial hemorrhage (grade III or IV)
- ROP requiring intervention (surgery or Avastin).

ROP will be included as a severe adverse event, although due to the timing of screening, which begins at 31 weeks, it will most likely be too late to discontinue treatment. Each case of ROP will be reviewed in real time. Subjects will be screened for ROP as per the 2006 guidelines: for

24-27 week gestation infants, the first screening will occur at 31 weeks PMA.[132] The timing of follow-up ROP exams will be determined by findings on the initial exam.

Resolution of SAEs:

- NEC
  -When infant is able to take full feeds, or what is considered equivalent to full feeding for that particular infant. If the child does not make it to full feedings due to short gut, note the method of feeding at the time of discharge (e.g. Home TPN)

- ROP
  -When an infant is diagnosed by an ophthalmologist exam with mature vessels
  -They no longer require frequent follow up ophthalmology appointments

- IVH
  -When shunt placement has occurred.
  -When sequential cranial ultrasounds show the ventricle size is stable or getting smaller.

- Hypertension
  -When hypertensive medications are discontinued
  -If infant is discharged on antihypertensive medications

- Sepsis
  -When antibiotics are completed.

- Thrombosis
  -When anticoagulation treatment ends.

- Pulmonary Hemorrhage
  -When no more blood in tracheal secretions and with stable vent settings.

Additional serious events which may be expected or unexpected include:

- Death
- Cardiac arrest
- Other unexpected life threatening event

The following flow diagram will be used to determine how to label an adverse event.

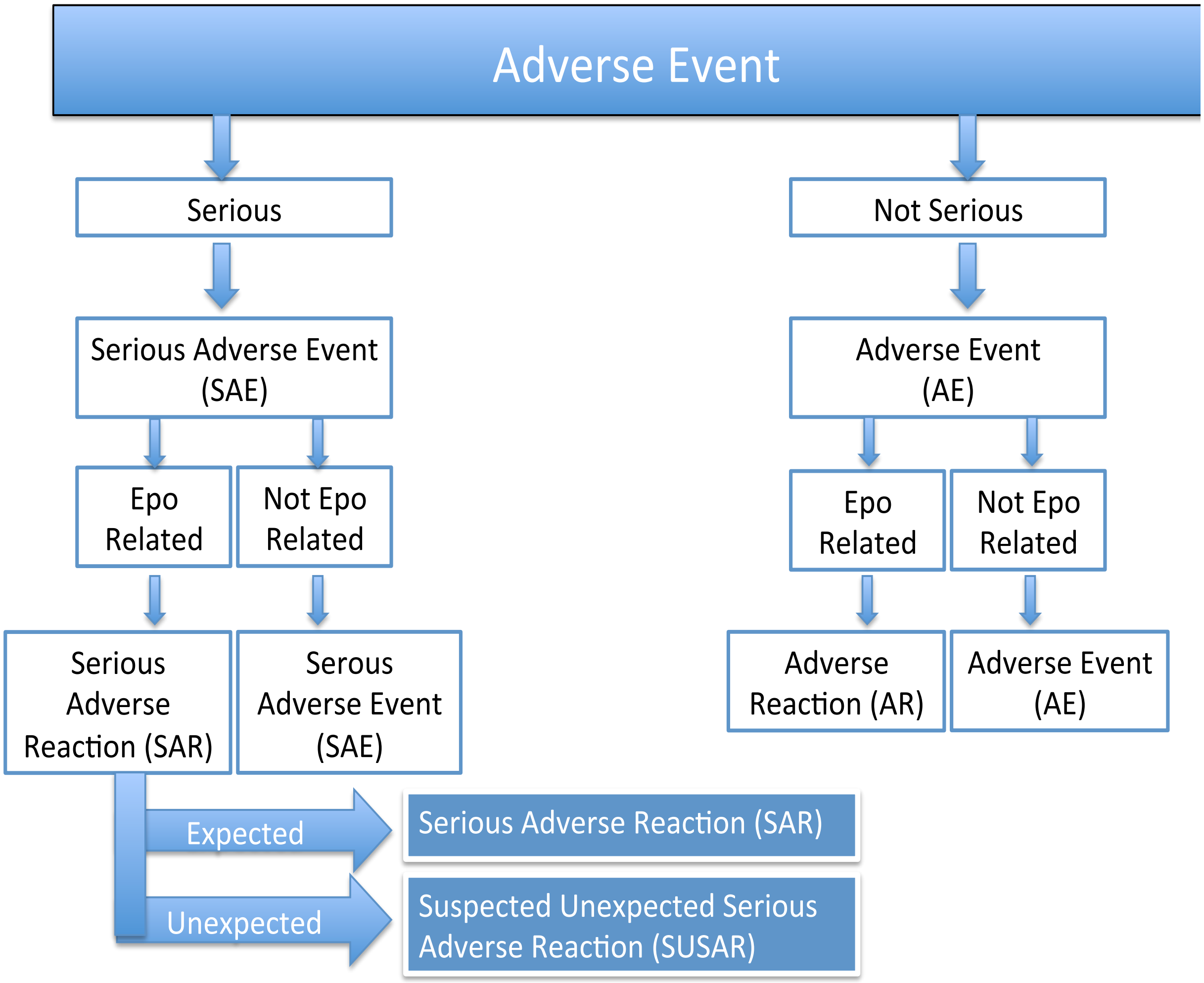

Criteria for Temporarily Withholding/Stopping Study Drug and/or Supplemental Iron.

1. **Polycythemia:** Central hematocrit (Hct) > 65%: Study drug should be held until Hct is ≤ 55%.
2. **Severe sepsis**: Blood culture-proven bacterial or fungal sepsis requiring blood pressure support or significant new respiratory support. Supplemental iron *should be held until blood culture is negative for 72 hours.* Rationale: Iron has been reported to be permissive for selected gram-negative bacteria and might worsen the patient's condition during sepsis. **There is no known relationship between Epo and sepsis**, so study drug does not need to be held.
3. **Unexplained recurrent seizure** (unrelated to ICH, PVL or other known pathology): Study drug should be held until seizures are well controlled by medication. Restarting study drug will be determined by the Medical Monitor, DSMB, and CCC.
4. Major **venous or arterial thrombosis (clot).**
   a. Study drug should be held for any thrombosis that is treated with a course of anticoagulation.
   b. Study drug should be held for any **symptomatic** thrombosis involving a major vessel (e.g. symptoms such as superior vena cava syndrome)

5. **Sustained hypertension *requiring medical intervention***: Study drug should be discontinued if blood pressure requires treatment. When blood pressure returns to normal range (systolic blood pressure < 100 mmHg), study drug can be resumed. This is true even if patient is still being treated, but blood pressure is being controlled.

In the event that study drug is discontinued in any subject, sites will encourage parents to continue to allow their child to participate in the follow-up evaluations.

2) Safety during the initial hospitalization. Due to the inherent vulnerability of this patient population, the rate of comorbidity is high, particularly in the most immature infants. The most current week-specific data for large populations of similar infants are provided by the neonatal research network (9575 infants of extremely low GA (22–28 weeks),[5] and by the ELGAN study group (1506 infants 23-27 weeks).[43, 133-136] We will use standard definitions of respiratory distress syndrome (RDS), BPD, ICH, PVL, early-onset and late-onset sepsis, NEC, patent ductus arteriosus (PDA), clinically diagnosed seizures (confirmed by EEG), hypertension, and ROP.[5] We will track the incidence of each of these disorders for all enrolled subjects using REDCap data entry forms.

Prior to study drug dosing, study staff will check subject's medical record for SAEs and criteria for temporarily withholding/stopping study drug and/or supplemental iron.

3) Long term outcomes. During the phone interviews that will occur at 4, 8, 12, 18 months, and at the face-to-face 2 year (22-26 months) corrected age visit, data will be obtained regarding interval hospitalizations, medication use, resource utilization, medical diagnoses, and oxygen requirement. At 12 months, parents will also be queried about their child's development. At two years corrected age, weight, blood pressure, and neurodevelopmental outcomes will be documented (neurologic exam, Bayley III scores, and M-CHAT-R evaluation). Urine and blood will also be collected at this time.

5.2 Handling of Study Interventions

Procrit will be utilized for this study (manufactured by Amgen, distributed by Centocor Ortho Biotech).

Study Drug Procurement by Site Research Pharmacies

Investigational Drug Services or corresponding pharmacy services at each site will purchase single dose, preservative free vials of PROCRIT, Epoetin alfa, 2000 Units/mL (NDC 59676-302-01) for use on this study. An inventory of drug will be purchased specifically for this study. The invoices for the purchase of the Procrit will be retained at the site in the same manner that a study drug shipping receipt would be retained. A copy of the invoice and the drug accountability logs must be submitted for reimbursement at approximately six month intervals by pharmacy. Because of the blinded nature of the PENUT Trial, the pharmacy must send this information directly to the CCC financial coordinator. The study investigator(s) and coordinator(s) cannot review this information.

Single-dose, Preservative-free Vial: Each 1 mL of solution contains 2000 Units of Epoetin alfa, 2.5 mg Albumin (human), 5.8 mg sodium citrate, 5.8 mg sodium chloride, and 0.06 mg citric acid in Water for Injection, USP (pH 6.9 ± 0.3). This formulation contains no preservative.

Preservative free normal saline will be used as placebo for the first 6 doses, and will be provided by local sites.

Blinding/Drug Administration.
After enrollment, the DCC will provide randomization information to the site pharmacy. The site pharmacy will then dispense study drug in a closed container. Epo and placebo (saline) are the same in appearance, and will be labeled "study drug". The first 6 doses of study drug will be administered IV push followed by 1 mL saline flush given over 2 minutes. These IV doses can be administered either by the bedside nurse or a research nurse since the study drug will be blinded by the pharmacy.

Maintenance Epo will be administered by subcutaneous injection using a 27 or 30 gauge needle on Monday, Wednesday and Friday, beginning on the day closest to completing the high dose series. To maintain the blind, pharmacy will dispense "PENUT STUDY drug" in a syringe to all infants. Control infants will not undergo any maintenance injections. A nurse who is not directly involved with the care of the patient, and who will maintain confidentiality will administer treatments behind curtains or blinds. A cohort of charge nurses, or General Clinical Research Center (GCRC) nurses would be ideal for this task. After injection or sham injection, all infants will have the injection site covered by a 2 x 2 gauze and wrapped with a gauze bandage, which is secured by taping it to itself. The time, date, and study drug intervention will be documented for each drug administration event.

Exceptional situations:

- If a dose of study drug is unavoidably delayed or missed, the dose should be given as soon as possible. This should be recorded as a study deviation. The normal schedule of administration should then be resumed. At least 24 hours must separate the high dose study drug doses. This allows the plasma Epo concentration to return to baseline.

- If severe edema (anasarca) or other circumstances prohibit the use of subcutaneous injection, up to three doses of Epo or saline placebo may be given intravenously using the normal IV protocol. In this case, pharmacy must be notified so they can provide an appropriate study drug formulation for IV use (saline rather than an empty syringe used for sham injection). This should not be recorded as a deviation for up to 3 doses. Subsequent IV doses would be considered a deviation.

- If the baby's IV is removed prior to completing the 6 intravenous study drug doses, the dose can be administered subcutaneously. This should be recorded as a study deviation.

The risks of intravenous administration of medication include infiltration, infection, and possible bleeding. The risks of subcutaneous injections include bruising, pain at the site of injection, and infection.

Iron supplementation: Iron supplements (oral and intravenous preparations) will be obtained from each site pharmacy as per their usual practice.

Iron Treatment Plan. Please see Appendix 7.
Maintaining iron sufficiency in a growing preterm infant is important for normal brain growth. Because of this, we have established iron guidelines that are to be followed at all sites. When enteral feedings are started, a standard iron containing formula will be used if breast milk is unavailable. Once infants (all subjects) have an enteral intake of 60 mL/kg/d and are at least one week old, they will be started on enteral iron at a dose of 3 mg/kg/d total. Enteral iron will be increased to 6 mg/kg/d when infants achieve an enteral intake of 100 to 120 mL/kg/d.[137] This is a well-tolerated dose that helps to prevent iron deficiency in preterm infants.[137] Serum ferritin or

ZnPP/H ratios should be checked at 14 and 42 days, and iron adjusted accordingly. If the ferritin is not in the desired range at the 14 day check, please adjust the iron dose and recheck ferritin (or ZnPP/H) at 28 days (see figure). If subjects are not able to tolerate enteral feedings and oral iron supplements, they will be given maintenance iron parenterally as noted in Appendix 7.

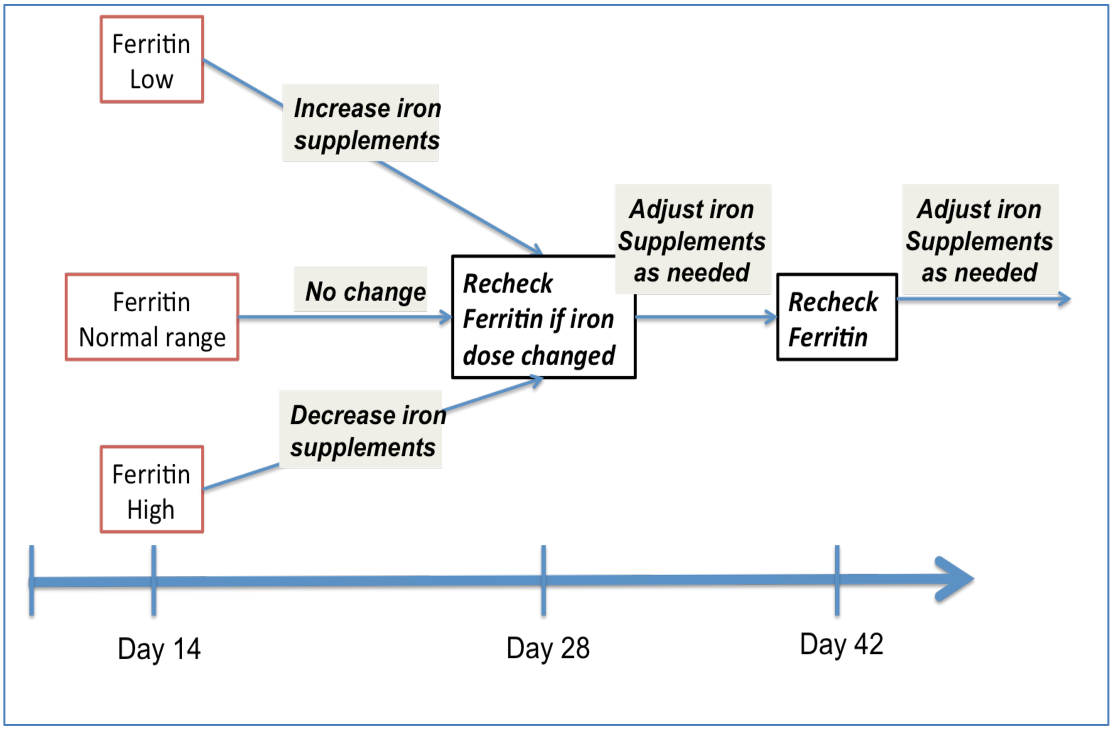

We estimate that less than 20% of babies will receive one parenteral dose of iron, since feedings are most often started by day 2 or 3, and advanced by 10 to 20 mL/kg/day. More than two parenteral doses will be rare. If the child is NPO for a prolonged period, parenteral iron dosing should be adjusted based on serum ferritin or ZnPP/H.

5.3 Concomitant Interventions

Infants < 27-6/7 weeks of gestation require treatment for associated problems of prematurity. Interventions include, but are not limited to, mechanical ventilation, sedation, treatment of seizures, treatment of PDA either medically or surgically, treatment of sepsis (bacterial or fungal), transfusions with packed red blood cells or platelets, treatment of NEC either medically or surgically, and assessment and treatment for ROP. Preterm infants are also at risk for acute kidney injury and late hypertension. Data will be collected on associated conditions and medications used to treat them during the course of the subject enrollment.

5.3.1 Required Interventions. 1) Epo/placebo treatment as designated by randomization; 2) cranial ultrasound within 6 hours of (preferably prior to) first study drug dose; 3) iron supplementation; 4) blood drawing 2.5 mL for Epo and biomarkers plus up to 1.2 mL for iron studies; 11 urine samples obtained at baseline and study days 3-5, 7-9, 14±1 day, then during weeks 3, 4, 6, 8, 10 and 12, and at the 2 year follow-up; 5) MRI at 36 weeks PMA depending on study site; neurodevelopmental follow-up at 22-26 months corrected age at all study sites. With parental permission, ongoing follow-up will be obtained by phone at 6 month intervals.

5.3.2 Prohibited Interventions. Use of any erythropoietic agents such as Epo or Darbepoetin off protocol is prohibited for subjects enrolled in this trial up to 44 weeks PMA.

5.3.3 Precautionary Interventions. The attending physician or responsible family member can remove a subject from the study at any time. If a subject develops any SAE presumed to be related to study drug, study drug will be discontinued as determined by the site PI and the CCC PI in consultation with the Medical Monitor.

5.4 Adherence Assessment

All medication dosing will occur in the NICU and will be documented by the nurse and the pharmacy. Accuracy and compliance will be assessed by measured Epo concentrations drawn as shown in Figure 3.

A cranial ultrasound will be done before (or within 6 hours of) initial study drug dosing and will be logged into the PENUT Portal.

Case report forms (CRF) will be completed and entered into the PENUT Portal: baseline, high dose and discharge.

In a subset of 220 subjects, a brain MRI will occur prior to discharge at 36 weeks PMA, and will be documented. The research study coordinator will log phone contacts after discharge, and the follow-up assessments will be documented on study forms, which will be submitted electronically to REDCap.

All contact with families after discharge will be recorded in the Follow-Up CRFs. The follow-up phone calls at 4, 8, 12 and 18 months corrected age will assess medical health and functional condition and remind parents of need for neurodevelopmental assessment. Attendance at follow-up clinic for the in-person 2 year corrected age assessment will be documented.

Protocol Violation/Deviation Reporting.

Violations of the study drug administration protocol require completion of a Study Deviations and Violations form and notification to the CCC and DCC within 7 working days. Protocol deviations should also be recorded on the Study Deviations and Violations form.

Major protocol violations include: enrollment in light of exclusion criteria, consent obtained not in accordance with IRB guidelines, unblinding of study personnel, study drug administration or dosing error.

Study deviations include: failure to administer all doses of study drug, study drug administration deviated from PENUT protocol, no baseline cranial ultrasound done, no baseline blood drawn, baseline cranial ultrasound delayed > 6 hours after 1st dose study drug and first study drug dose give > 26 hours of life (24 hours plus 2 hours of wiggle room).

6. CLINICAL AND LABORATORY EVALUATIONS

6.1 Schedule of Evaluations

Table 5. Evaluation	Screening		Pre-Entry (Prior to 24 hrs)	Entry	1-7 day	7-10 days	12-15 days	28-42nd days	32 weeks PMA	34-36 weeks PMA	Discharge	4 months CA	8 months CA	12 months CA	18 months CA	24-26 months CA	30 months	36 months	42 months	48 months	52 months	60 months
Screen admissions to antepartum unit and NICU	X		X																			
Documentation of Gestational Age			X																			
Informed Consent			X																			
Maternal Demographics and History				X																		
Screening Cranial ultrasounds				X		X				X												
Blood Pressure				X	X	X	X	X	X	X	X					X						
Urine collection				X	X	X	X X	X X	X	X	X					X						
Epo concentration				X	X	X	X															
Adherence Assessments				X	X	X	X															
Stored Plasma for biomarkers				X		X	X															
Ferritin or ZnPP/H							X	X														
MRI (subset of 220 subjects)										X												
Hematology (CBC/transfusions/phlebotomy loss) as available from clinical care				X			X	X	X		X											
Liver/kidney Function Tests (as clinically available)							X	X	X	X	X											

Complete Physical Exam including weight, head circumference and length				X			X				X											
Telephone contact to review current status												X	X	X	X		X	X	X	X	X	X
Comprehensive Neurodevelopmental Assessment																X						

6.2 Data to be collected on Each Subject

Gestational age, birth weight, maternal history, placenta histology (intrauterine infection, insufficiency), Apgar scores at 1, 5 and 10 minutes, will be documented in addition to measures of maternal education, SES, and other factors known to affect cognitive development (e.g. drug/alcohol exposure). NINDS common language will be used when possible. A cranial ultrasound will be obtained prior to study drug dosing.

6.3 Timing of Evaluations

Data collection for hematology, chemistry, liver and kidney function will be as clinically available. These tests are done routinely in all extremely preterm infants as part of usual care, and will not be required as part of the study protocol. The research nurse or designee will collect these results from the medical record.

Cranial ultrasounds at baseline, 7-10 days and at 36 weeks PMA (30 days of age or later is acceptable) will be sent to UW CCC, as will all brain MRIs. Analysis will be done using the most severe lesion noted.

6.3.1 Pre-Randomization Evaluations

These evaluations occur prior to the subject receiving any study interventions.

Screening

If appropriate, families will be approached for study entry while on the antepartum service, if delivery seems reasonably imminent. All NICU admissions will also be screened daily for eligible patients.

Pre-Entry

After birth, consent must be obtained by 24 hours of age to be eligible for study participation. The time window for initiation of study intervention from birth is 24 hours.

6.3.2 On-Study/On-Intervention Evaluations

After consent is obtained, a cranial ultrasound must be obtained prior to, or within 6 hours, of the first study drug dose. Results of the cranial ultrasound will not affect randomization or clinical care. These data will be used for later analysis of outcomes only.

Physical exam will be done at study entry, after the first 6 doses of intravenous study drug, and at discharge. Complete blood counts (CBC) drawn as part of clinical care will be recorded, as will laboratory evaluations of renal and hepatic function.

Blood draws: Blood draws are timed according to Study drug dosing (see Figure 3). All subjects will have 5 blood draws (0.5 mL volume each) as part of this study. This blood will be used to determine plasma Epo concentrations, biomarkers of brain injury, and the effect of Epo on systemic inflammatory response. The cell pellets will be frozen and saved for future studies that are beyond the scope of this study. A final (optional) blood draw will be obtained at the 2 year follow-up visit. This blood will be evaluated for renal function for the purposes of identifying markers of kidney injury at follow-up.

Collection Schedule

1. Day 0: Baseline - prior to first study drug dose (may use cord blood)
2. Day 7: 30 min after the 4th study drug dose (peak)
3. Day 9: 30 min prior to the 5th study drug dose (trough)
4. Day 14 ± 1 day: Denote the time of draw on sample relative to drug dosing
5. 22-26 months corrected age

Blood draws will be synchronized with the clinically needed blood samples when possible. Epo measurements will be done in all subjects, and cytokine measurements (Aim 3) will be done in the subset of 220 subjects (110 per arm) who will also be undergoing an MRI at 36 weeks PMA.

Following serum ferritin (0.4 mL) or ZnPP/H (0.1 mL) to assess iron status is strongly recommended beginning at 14 days. If these lab values are outside the normal range, it is recommended that iron supplementation be adjusted, and repeat labs (ferritin or ZnPP/H) be followed more closely (at 2 week intervals). This would increase the total blood volume drawn for study purposes by 0.2 (if ZnPP/H is assessed) to 0.8 additional mL of blood (if ferritin is assessed). These volumes (Maximum of 2.8 mL) are within the IRB recommendations for infants of this weight: "Existing guidelines for blood sample volume limits (ranging from 1–5% of total blood volume within 24 hours and up to 10% of total blood volume over 8 weeks) are consistent with the limited evidence available on "minimal risk" to children".[138] Given that the blood volume of premature infants ranges from 80 to 85 mL/kg, their total blood volume would range from 40 mL (80 x 0.5 Kg) to 85 mL (85 x 1 Kg). The maximum amount of blood to be drawn for study purposes is 2 + 0.8 mL = 2.8 mL, which is within the guidelines.

Blood draw precautions: If there is a venous or arterial catheter in place appropriate for blood drawing, this will be used preferentially. If no access is available, standard neonatal phlebotomy techniques will be used.

Urine collections

Rationale: Little is known about acute kidney injury (AKI) in extremely preterm infants. Serum creatinine (SCr) and urine output are not ideal markers to define AKI, especially in neonates. Even in adults, the increase in SCr is known to be a relatively late occurrence. The use of SCr in neonates is complicated by the fact that in the first few days of life, it reflects maternal SCr.

Urine biomarkers of cell injury/repair are felt to have great potential. However, before these novel biomarkers can be used clinically, we must evaluate their ability to predict a SCr rise, as well as short and long-term clinical outcomes. The goal of collecting detailed information about renal function in these infants is ultimately to improve the ability to diagnose AKI, and to better understand the epidemiology and pathophysiology of AKI in extremely low gestational age neonates. BUN and creatinine are commonly checked as part of routine care of extreme preterm infants. When obtained for clinical indications, these values will be recorded.

Neonatal AKI has been classically defined by oliguria/anuria and/or a persistent SCr elevation. We will use categorical definitions to make the diagnosis of AKI, similar to definitions published by KDIGO (2012) http://www.kdigo.org and the AKIN groups (2007). Jetton, et al., proposed a modification of the adult Acute Kidney Injury Network Criteria (AKIN) definition of AKI as a standardized definition of AKI for neonates based on a rise in SCr from a previously documented low, rather than on an absolute SCr threshold.

Stage 0: No change or rise < 0.3 mg/dL
Stage 1: Increase in SCr 0.3 mg/dL or a SCr 150–200% from previous trough value
Stage 2: Increase in SCr 201–300% from previous trough value
Stage 3: Increase in SCr > 300% from previous trough value or 2.5 mg/dL or need for dialysis

Urine will be obtained at timed intervals as shown in Figure 4. With the exception of the initial urine, which will be collected at the time of enrollment, if the date of urine collection falls on a Saturday or Sunday, the urine will be collected on the closest weekday. Do not delay study drug dosing if no urine is available at enrollment, but still obtain first urine available. Urine samples are requested at the time of enrollment, on day 3 to 5, day 7 to 9, with a preference for day 7 if that is possible, day 14 ± 1 day, and then during week 3, 4, 6, 8, 10 and 12. If possible, pick one day of the week to collect urines, and do this consistently. This is to minimize the work for the site coordinator as much as possible.

Figure 4.

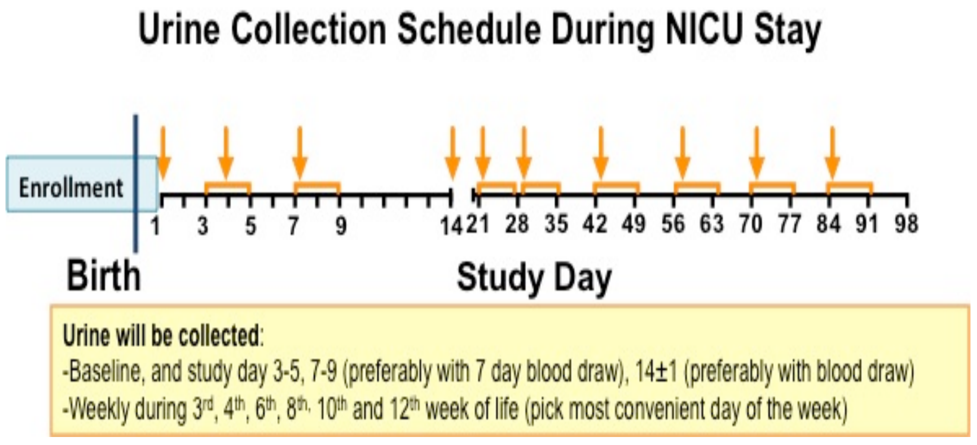

The urine samples will be banked for future analyses of biomarkers (neutrophil gelatinase associated lipocalin (NGAL), KIM-1, osteopontin (OPN), cystatin C (CysC), uromodulin (UMOD), epithelial growth factor (EGF) and urine β2-Microglobulin (β2MG). There are increasing data in older children as to the validity of such markers for diagnosis of AKI, but very little data in preterm neonates. Additionally, prior to including these markers as part of the neonatal AKI definition, more knowledge is needed on other clinical factors which may affect urinary concentrations of these biomarkers independent of renal function (e.g. inflammation, NEC or other neonatal specific conditions). Advantages to eventually including novel renal injury biomarkers in a future definition of neonatal AKI are that they would ideally represent actual renal tubular injury versus the solely functional markers of AKI currently used.

Urine will be collected by placing cotton balls in the infant's diaper with a diaper change. (If needed, a bag can be used to collect urine instead of cotton balls.) At the next diaper change, these cotton balls will be placed in an empty 10 mL syringe and the plunger will be used to squeeze the urine out into collection tubes. The collection tubes will be labeled with the infant's study ID number, and date. The urine will then be frozen and sent in batches to the University of Washington for further processing and analysis.

Brain Imaging in Premature Infants. Please see Appendix 6.
The review titled: "Practice parameter: neuroimaging of the neonate: report of the Quality Standards Subcommittee of the American Academy of Neurology and the Practice Committee of the Child Neurology Society" published in 2002 gave recommendations for the timing and grading of ultrasound finings.[139] Cranial ultrasounds are recommended at day 7-10 and at 36-0/7 to 36-6/7 weeks PMA or prior to discharge if this occurs earlier. Our study guidelines are modified from these recommendations in that an additional research cranial ultrasound will be done on day 1, prior to (or within 6 hours of) the first study drug administration. While our guidelines request a late cranial ultrasound or MRI be done at 36 weeks PMA, we will accept a CUS done after 30 days of age.

Magnetic Resonance Imaging. Please see Appendix 2 for pulse sequences.
MRI's will be obtained on 110 subjects from each study arm at 36 weeks PMA (220 total). All scans will be done on a 3T Siemens or 3T Philips scanner using an optimized protocol determined by the Imaging committee. These scanners are available at the University of Florida in Gainesville, Florida Hospital for Children in Orlando, Methodist Children's Hospital in San Antonio, University of Minnesota in Minneapolis, Wake Forest School of Medicine in Winston-Salem, Johns Hopkins in Baltimore, University of Washington in Seattle and Maria Fareri Children's Hospital in Valhalla. (Please see Imaging SOP.)

Automated Developing Tissue Segmentation: Analyses will be based on automated processing steps developed specifically for analysis of early developing brain anatomy, as part of separate on-going R01 projects on fetal and premature neonatal brain image analysis, directed by Colin Studholme, PhD (R01 NS 061957 and R01 NS 055064). Validated automated techniques will segment gray matter, myelinated, and un-myelinated white matter using 3D T1W MRI data. These make use of age-specific spatial statistical priors synthesized from a computational spatio-temporal atlas[140, 141] that models age-related variations in tissue volume, tissue probability, and MRI contrast for an automated expectation maximization based labeling scheme, illustrated in Figure 5.

Figure 5. Preliminary results using a specialized template driven expectation maximization (EM) scheme (left) to label T1W MRI data of a premature neonate (right) with developed and developing tissues (cyan-myelinated white matter boundary, blue-unmyelinated white matter boundary, red-CSF boundary

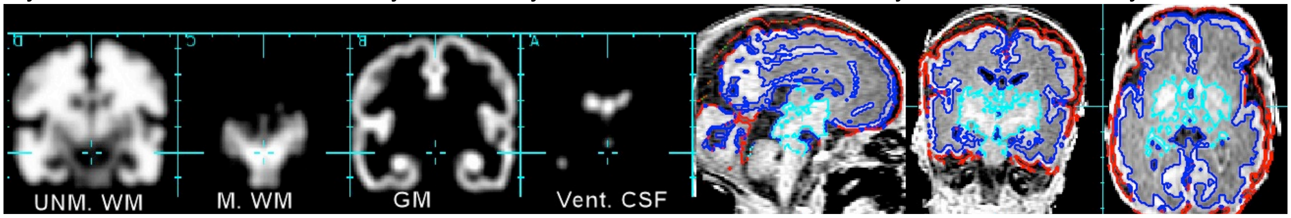

Following tissue segmentation, anatomical regions will be automatically labeled (Figure 6) using fine scale unbiased spatial normalization to form an unbiased minimum deformation group average anatomy.

Spatial Normalization for population based analyses. Accurate spatial normalization of developing anatomies allows population-based mapping of structural differences in subjects. For structural image analysis we will use template-free, symmetric, group wise, spatial normalization of anatomies to form an unbiased average anatomy from all scans being studied. This will make use of accurate, automated developing brain tissue segmentation techniques to align only boundaries present in each subject's anatomy and exclude focal pathological variations in tissue contrast such as blood clots that may be present in some anatomies and potentially induce incorrect mappings between brain structures.

Evaluation of Global and Regional Tissue Growth (GRTG) summary measures. We will first use the automated tissue segmentations to evaluate summary measures for global and regional growth rates in each subject for conventional statistical analyses. Gray matter, myelinated white matter and unmyelinated white matter will be estimated by integrating tissue label probabilities over the whole brain and within a set of pre-

Figure 6: Left: Example delineation of the preterm cerebellum to create a set of 10 reference MRI scans with delineated anatomy. Right: 3D Rendering illustrating initial gyral marking protocols and techniques applied to a pair of MRI studies of a single infant acquired at 32 and 38 wks PMA.

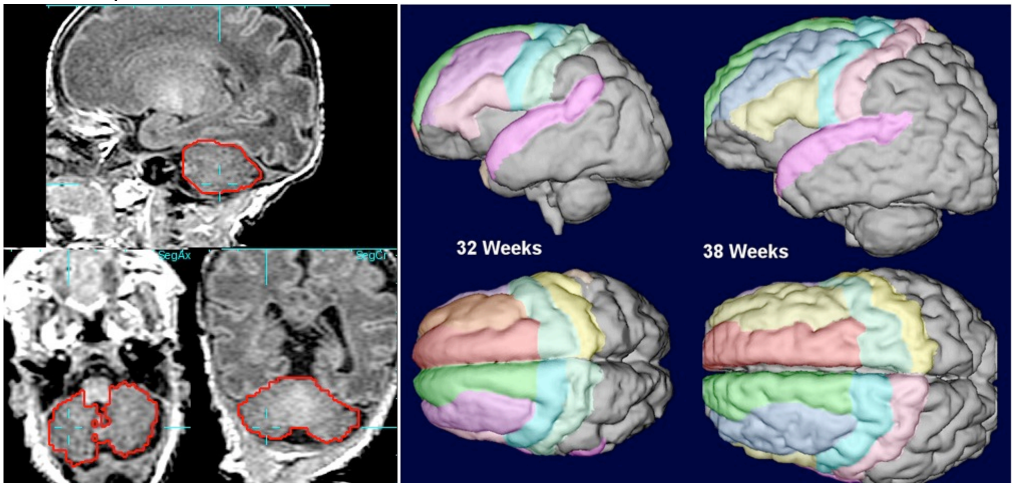

defined brain regions. Each MRI will be automatically labeled with an optimized patch-based brain labeling technique[142] based on a subset of manually delineated MRI scans that are aligned to form a statistical model of the labeling. The labeling approach combines multi-atlas based methods with patch-based techniques to optimally assign region labels to each new individual MRI scan. The resulting label maps combined with tissue segmentations provide summary measures that can be analyzed using linear modeling described in more detail by the DCC.

Evaluation of patterns of tissue growth using Tensor-Based Morphometry (TBM) of Structural MRI. We will use TBM to look for spatially localized biomarkers of abnormal brain tissue growth across the population using techniques developed and validated in fetal brain imaging.[44] The local volume changes induced by the mapping from the group average to each individual brain will be used to characterize the developing brain anatomies. These will be evaluated from the Jacobian determinant of the deformation at each voxel.[143] We will apply general linear modeling to analyze these maps to account for age and other covariates together with main outcome measures on a voxel-by-voxel level, generating regression coefficients and associated t-statistics at every voxel in the average anatomies by spatial parametric mapping.[144] Correction for multiple comparisons will be achieved using an efficient parallel implementation of non-parametric permutation testing.[145] This analysis permits a spatial hypothesis free exploration of how local tissue growth rates are related to outcome and treatment variables across the study population.

Evaluation of Cortical Folding using Surface Curvature Mapping (SCM). We will use the automated segmentations of cortical tissue boundaries to evaluate the local and global state of neonatal brain folding to look for perturbations in cortical development, using techniques our group has previously used in both fetuses and premature neonates.[146, 147] Each cortical surface will be tessellated into a locally topologically-correct triangular mesh[148] and a set of normalized curvature measures will be evaluated at each point on the surface to provide direct quantitative measures of cortical evolution. Each subject's surface folding map will be transformed into the unbiased population average anatomy using the deformations described above for TBM analysis. These are mapped onto a common tessellated average surface[146] to allow statistical parametric mapping using a vertex-by-vertex general linear model. As with TBM, this generates a map of regression coefficients and t-statistics (here on a surface of vertex locations), which is corrected using an efficient parallel implementation of non-parametric permutation testing,[145] again permitting a spatial, hypothesis-free exploration of how local and global cortical folding is related to outcome and treatment variables across the study population. Figure 6 shows an example of changes in surface folding over time in the developing fetus.

MRI Analysis. All MRI images will be sent to UW CCC for analysis, where they will be will be converted to DICOM format and stored on the DICOM server. Todd Richards, MD will write customized software to prepare data for analysis at the UW CCC site such as reformatting, resorting, and extraction of diffusion b-values b-vectors for tensor calculations. He has already written b-values, b-vector extraction code for the Philips Acheiva scanners but will need to customize code for the Siemens scanners. Standardized phantoms (The Phantom Laboratory, Incorporated) will be used at each site to establish data integrity for quality control of MR data.

Qualitative image analysis will be performed by the clinical neuroradiologist at each participating site so the information is available to the subject's care team. For study purposes, each MRI will be evaluated by Drs. Dennis Shaw and Manjiri Dighe, who will be blinded to the treatment group, and all clinical data. Qualitative evaluation will be done using the recently published MR Imaging Assessment Tool created by the Inder group to define brain abnormalities in very preterm infants at term corrected age.[149] This scoring system defines abnormalities of white matter, gray matter, and includes measures of hemorrhage, and brain loss. The table below is excerpted from this manuscript.

Table 1: Prevalence of infants with each item in cerebral WM score (VPT infants/term infants)

Variables	WM Score				
	Score 0	Score 1	Score 2	Score 3	Score 4
Cerebral WM					
Cystic lesions	None (90/22)	Focal unilateral (3/0)	Focal bilateral (1/0)	Extensive unilateral (2/0)	Extensive bilateral (1/0)
Focal signal abnormality	None (77/20)	Focal punctate (13/2)	Extensive punctate (5/0)	Linear (2/0)	
Myelination delay	PLIC & corona radiata (65/22)	Only PLIC (26/0)	Minimal—no PLIC (6/0)		
Thinning of the corpus callosum	None (40/18)	Partial (genu/body < 1.3 mm or splenium < 2.0 mm) (53/4)	Global (genu/body < 1.3 mm and splenium < 2.0 mm) (4/0)		
Dilated lateral ventricles	Both sides VD < 7.5 mm (26/17)	One side 7.5 mm ≤ VD < 10 mm (19/4)	Both sides 7.5 mm ≤ VD < 10 mm or one side VD ≥ 10 mm (42/1)	Both sides VD ≥ 10 mm (10/0)	
Volume reduction	cBPW ≥77 mm (21/19)	77 mm > cBPW ≥72 mm (30/2)	72 mm > cBPW ≥67 mm (40/1)	67 mm > cBPW (6/0)	

Note:—cBPW indicates corrected biparietal width; *PLIC, posterior limb of internal capsule; VD, ventricular diameter.

Quantitative analysis. We will use a range of highly sensitive automated techniques to quantitatively assess brain development at 36 weeks PMA using MRI analysis tools developed as part of on-going projects in the biomedical image computing group at UW (http://depts.washington.edu/bicg) led by Dr. Studholme. We will examine global and regional summary measures of tissue volume,[150] cortical folding[147] and microscopic water diffusion properties from each subject scan to examine whether the state of overall brain growth at 36 weeks PMA has been modified by the administration of Epo. We will then explore the possible presence of focal differences in growth patterns that may be related to specific functional deficits. This will use group wise spatial normalization to examine the spatial pattern of tissue volume increase,[44] and water diffusion, together with patterns of cortical surface folding.[146]

DTI Analysis: The methodology detailed below is currently used by our research group at the University of Washington's Diagnostic Imaging Sciences Center (DISC).

Preprocessing. DTI quantification will be preceded by head motion and eddy current correction using affine registration to a reference volume[151] with FDT (FMRIB's Diffusion Toolbox; http://www.fmrib.ox.ac.uk/fsl/fdt/index.html). Using the field maps, B0-field inhomogeneity induced geometric distortion in the eddy current-corrected images will be corrected with PRELUDE (phase Region Expanding Labeller for Unwrapping Discrete Estimates)[152] and FUGUE (FMRIB's Utility for Geometrically Unwarping EPIs; http://www.fmrib.ox.ac.uk.offcampus.lib.washington.edu/fsl/fugue/). FSL software DTIFIT will be used to fit the diffusion tensor model at each voxel in order to calculate DTI Eigen vectors, Eigen values, fractional anisotropy, axial diffusivity, radial diffusivity, and mean diffusivity (http://www.fmrib.ox.ac.uk/fsl/fdt/fdt_dtifit.html).

Development of 36 Week Infant Head Model for MRI. We will use software (MINC) developed at the Montreal Neurological Institute (http://www.bic.mni.mcgill.ca/ServicesSoftware/MINC) in order to co-register and combine 50 MRI structural brain images of normal infants at 36 weeks in order to make a head model to be used in DTI and VBM group comparisons Dr. Richards has experience using this software at I-LABS in collaboration with Dr. Patricia Kuhl where he used MINC to make a head model for 6 month old infant brain using 43 brains.

Figure 7. TBSS

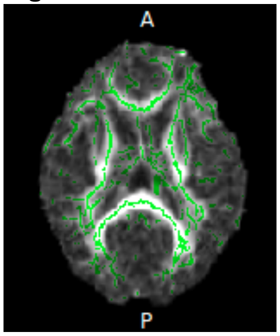

Evaluation of white matter integrity using DTI. Group differences in fractional anisotropy (FA), axial diffusivity, radial diffusivity, and mean diffusivity (similar to ADC)

will be determined using TBSS (Tract-Based Spatial Statistics).[153] TBSS was developed to conduct voxel wise analysis of multi-subject diffusion data utilizing improved non-linear registration techniques. An example of a 6 month old child studied at the University of Washington is shown in Figure 7. Data processing will be conducted according to the standard method detailed in the TBSS instruction manual (http://www.fmrib.ox.ac.uk/fsl/tbss/index.html). We plan to use Randomise, a permutation method, to test for between-group differences in these DTI measures. Correction for multiple comparisons will be done using whole brain cluster-based thresholding method: voxel height, $p < 0.01$; cluster extent, $p < 0.05$. Our group is well published in this area of diffusion imaging, DTI and DTI analysis.[154-157] Statistical comparison for TBSS will be performed using FSL-software called Randomise (http://www.fmrib.ox.ac.uk/fsl/randomise/index.html). This program will be used to perform a non-parametric voxel by voxel ANOVA (and correlations with clinical score) with multiple comparison correction using the Threshold-Free Cluster Enhancement option. Randomise is a permutation program enabling modeling and inference using standard General Linear Model design matrix setup using cluster-based tests. For more detail on permutation testing in neuroimaging see Nichols and Holmes.[145] The multiple comparison correction was performed based on cluster statistical characteristics such as extent of cluster size. Dr. Richards has written software to create the design matrix that will be used for both the ANOVA and clinical score correlations in software Randomise.

As part of the overall QA effort, we will examine various measures of study implementation across sites. In particular, recruitment, retention, data completeness, and measurement precision will be tabulated and compared across sites and will be included in our web-based reports. QA efforts and site visits will be focused on any sites that show evidence of problems.

Inflammatory Mediators. To test whether Epo treatment decreases serial measures of circulating inflammatory mediators and biomarkers of brain injury.

Circulating Proteins:
We will use Meso Scale Discovery (MSD) technology to measure the following inflammatory markers and growth factors: BDNF, Interferon-gamma (IFN-γ),[82] Interleukin (IL)-1 β,[158] IL-6, IL-8, IL-10, tumor necrosis factor-α (TNF-α), transforming growth factor (TGF)-β, matrix metalloproteinase (MMP)-2 and MMP-9,[159] macrophage inflammatory protein-1α (MIP-1α), MIP-1β[136], monocyte chemotactic protein-1 (MCP-1)[136] and tissue inhibitor of metalloproteinase (TIMP)-1.[159]

Markers of neurotoxicity and brain injury will include: S100B, glial fibrillary acidic protein (GFAP), neuron specific enolase (NSE), Activin A, and Ubiquitin C-terminal hydrolase-L1 (UCH-L1).[160-162]

A number of circulating proteins have been evaluated as potential biomarkers of brain injury. Promising biomarkers include the GFAP, Ubiquitin C-terminal hydrolase-L1 (UCH-L1), myelin basic protein (MBP), S100B, Activin A, and neuron-specific enolase (NSE), although the sensitivity, specificity and timing of elevation after injury in preterm infants have yet to be determined. All of these proteins, with varying levels of specificity, are elevated after brain injury in studies of adult or neonates. Due to the immaturity of myelination, MBP is unlikely to be useful in preterm infants.

GFAP is a specific marker of differentiated astrocytes and increased circulating concentrations are detectable within hours and peak at up to four days after an ischemic stroke.[160-163] Astrocyte foot processes make up the sub-endothelial component of the blood-brain barrier (BBB), so elevations of GFAP can also reflect loss of BBB integrity. UCHL1 expression is highly specific to neurons and to cells of the neuroendocrine system. Elevations have been demonstrated in term infants with HIE. S100B belongs to a family of calcium handling proteins and is localized predominately in glial cells in brain. It is thought to function in neurite extension, inhibition of PKC-mediated phosphorylation, axonal proliferation, and inhibition of microtubule assembly. NSE is a glycolytic enzyme localized primarily to neurons. However NSE is also expressed in platelets and erythrocytes, so elevations may not be specific to brain injury. GFAP has not been studied in the context of preterm brain injury, but a direct comparison of GFAP, S100B, and NSE showed that GFAP was a superior

biomarker of brain injury in the setting of traumatic brain injury.[164]

Activin A is a member of the transforming growth factor-β superfamily and is expressed by neurons. It is a trophic factor that regulates differentiation and proliferation of neurons.[165] In a cohort of 53 infants, Florio, et al., showed that high concentrations of Activin A (> 0.8 mcg/L) in blood samples drawn during their first hour of life was highly correlated with ICH in preterm infants < 32 weeks gestation (100% sensitivity and 93% specificity, with positive predictive value of 79%).[166, 167] Activin A is also increased in term newborns with moderate or severe asphyxia suggesting that activin is released after neuronal injury.[168]

Population Epo Pharmacokinetic Analysis. We will measure timed Epo concentrations to monitor for accumulation and safety, and to confirm Epo dosing. Plasma Epo concentrations will be measured in duplicate using Meso Scale Discovery (MSD) technology at the UW Laboratory Core. Batched samples will be run every 12-18 months. A population pharmacokinetic analysis will be performed using sparse plasma Epo concentration data. NONMEM software (Icon Development Solutions, Ellicott City, MD) will be used to determine compartmental population pharmacokinetic parameters (i.e., clearance and volume). The base structural model will be implemented as established in the population pharmacokinetic model developed from the intensive pharmacokinetic data of our pilot study.[35] The random-effect model will be updated using the concentration data from this study. A covariate analysis will also be performed in a standard step-wise forward addition followed by backward elimination method to determine the influence of clinical factors (e.g. gestational age, birth weight, serum creatinine, concomitant medications) on pharmacokinetic parameters. Based on the final population model, post-hoc Bayesian individual pharmacokinetic parameters will then be estimated for each participant. Pharmacodynamic relationships will be determined between Epo pharmacokinetics and the clinical and biochemical markers measured during the investigation.

Blood (0.5 mL/sample, 2.5 mL total) will be obtained through an indwelling umbilical arterial or venous catheter when possible. Samples will be spun for 10 min at 1000 g; plasma and cellular components will be frozen in separate labeled tubes at -70°C to -80°C. Samples will be labeled with a code provided by the DCC to maintain blinding. All samples will be sent to Dr. Juul's lab at the University of Washington. Each site will be sent premade packages containing the instructions, data sheets, sample labels, blood collection tubes and mailing labels with which to process the blood samples (See Laboratory SOP).

Neurodevelopmental Follow-Up: High-risk follow-up for ELGANs is part of standard care at all sites. Routine visits generally occur at 4 months, 8 months, 12 months, 18 months and two years corrected age. The timing of visits may vary depending on the child's need for intervention and site-specific protocols. If subjects are not scheduled at any one of these time points for routine high risk follow-up, parents/guardians will be contacted by telephone at 4, 8, 12, and 18 months corrected age. The 30 minute phone contact will include an update of the subject's medical problems, medications, use of services such as physical therapy. Please see Appendix 3 Follow-Up Manual for further details.

The primary outcome will be neurodevelopment at 22-26 months corrected age. All personnel involved in the neurodevelopmental assessments will be blind to study treatment. Assessment at 22-26 months corrected age will include:

- Bayley III Scales of Infant and Toddler Development
- Standardized neurological examination based on ELGAN Neurological Exam Study protocol (Appendix 3)
- The Gross Motor Function Classification System (GMFCS)
- Modified Checklist for Autism in Toddlers (M-CHAT-R) (Appendix 3)

Bayley Scales of Infant Development (BSID) is the standard test used to evaluate early neurodevelopmental outcomes of preterm infants.[13, 19, 169, 170] The second edition Bayley Scales, used from 1969 to 2005, was

modestly predictive of cognitive function at school age with MDI < 70 showing a positive predictive value of 0.19 – 0.8 for identification of children with an IQ < 70 at school age.[171-175] The cognitive portion of the Bayley test was revised in 2005[176, 177] (Bayley III Scales of Infant Development): the Mental Development index (MDI) reported in the Bayley II was a composite of cognitive, expressive and receptive language; in the revised Bayley III, individual scores for cognitive, expressive and receptive language are reported in addition to an assessment of social-emotional, and adaptive behavior. As experience has accumulated, it has become apparent that children score higher on the Bayley III compared to the Bayley II.[1, 177-183] Thus a cognitive score of 85 on the Bayley III likely corresponds to a Bayley II score of 70. Given that children assessed with the Bayley III are just now reaching school age, the value of the third edition of the Bayley Scales for prediction of cognitive impairment at school age and beyond is still under evaluation.[177] In order to ensure that the outcomes of this study are clinically important, we have identified two important stepped outcomes: the primary outcome is very stringent, and uses a cut off of 2 standard deviations below the mean for cognitive or motor scales (< 70). The secondary outcome uses a cut off of one standard deviation below the mean for these criteria (< 85), which will still have a significant impact on the child, family, and healthcare system.

Inter-Rater Reliability Training:
Bayley Scales of Infant and Toddler Development, 3rd edition. Individuals performing the BSID-III assessments for this study will be required to be certified after attending a formal Training & Orientation Workshop 6 months before testing as organized by Drs. Michael O'Shea and Jean Lowe (run in tandem with a neurological training session). The certification process is comprised of two parts. First, each candidate Infant Assessor will review the test publisher's "Enhanced Administration DVD," which will be employed in this RCT as the "gold standard" for administration and scoring of the Bayley III. Second, the candidate Infant Assessor will administer the Bayley to healthy full-term infants who are 24 months old. Video-recordings will be made of these administrations. The candidate Assessor will score each of his/her pilot administrations. A copy of the candidate's test protocol will be sent to the Study Psychologist (Jean Lowe, PhD) at the coordinating center, along with a copy of the video recording. The Study Psychologist will use these materials to assess the candidate's ability to (a) engage the infant in the Bayley activities, (b) administer and score each Bayley item, and (c) compile all summary scores. Infant Assessors will be required to meet the calibration standard for certification: 88% score concordance on items correctly administered. The Study Psychologist will provide prompt item-specific feedback, so that the candidate can make any needed modifications. Once Infant Assessors have begun to see enrolled patients, we will ask each Assessor to record one Bayley administration after the first 10 patients, or after the first 6 months, and then semi-annually. These video-recordings will also be reviewed by the Study Psychologist to guard against "calibration drift" over time. Training for Bayley assessment staff will include a semi-annual assessment to ensure continued reliable and valid assessment data. For non-English speaking infants, we will use an interpreter who interprets each of the Assessor's requests to the child.

Neurological exam. To standardize the quality of data regarding neurological exams, examiners will attend a one-day workshop at 4 convenient hub locations in the U.S., view a training video, and then classify neurological findings illustrated on an assessment video.[184] Inter-observer variability assessments will be done to determine agreement with gold standard responses. Annotated feedback will be given to examiners regarding items that had a less than 85% correct rate, and, based on experience in the ELGAN study, we expect agreement rate to rise to over 90%.[43, 184] To minimize expectation bias, examiners will be blinded to the child's medical history and brain-imaging studies. Findings from the neurological exam will serve as the basis for an algorithm that classifies infants into one of four groups: no CP, diparetic CP, hemiparetic CP, and quadriparetic CP.[185] These categorizations correlate highly with long-term neurodevelopmental outcomes. We will use the neurologic exam forms created for the NINDS-funded ELGAN study (Appendix 3). In addition, CP severity will be determined using the Gross Motor Function Classification System (GMFCS) developed by Palisano.[3, 4, 186, 187] A GMFCS score of 2 or greater will be considered severe CP, given that these children will not be able to walk independently at age 2.

b) Standardized Diagnosis of Cerebral Palsy and Quantification of Motor Delay
The presence and sub-type of CP will be determined using the broadly accepted and standardized ELGAN Neurological Exam scoring system and CD based video teaching system.[184, 185] This software and training program developed by the PENUT collaborator Dr. Karl Kuban provides a distributable method of neurologic testing of subjects in a formalized, systemized method that is highly reproducible.[184] CP severity will be determined using the Gross Motor Function Classification System (GMFCS).[4, 186, 187]

Rationale for diagnosing sub-types of CP:
Although all forms of CP will be tabulated as part of the primary outcome, relationships between CP sub-types and cognitive development vary by the brain region affected and so we will classify CP into three sub-categories:
- Spastic Quadriparesis: has a higher association with structural injury and quadriparetic children are 9-times more likely than diparetic children to be severely impaired and 5-times more likely than diparetic to be microcephalic. They are also twice as likely to have MDI < 70 and a high rate of positive M-CHAT-R scores vs. diparetic and children without CP.

- Spastic Hemiparesis: represent an intermediate level of injury where 53% of this form of CP have Bayley < 70 vs. 75% for quadriparesis and only 34% for diparesis; consistent with a more common association with unilateral injury and focal lesions like middle cerebral artery stroke, that spare fibers closest to the ventricle in control of lower extremities.

- Spastic Diparesis: Diparesis is more commonly associated with symmetrical white matter injury close to the ventricle presumable due to injury of leg fibers of the pyramidal system that are closest to ventricles.

Gross Motor Function Classification System: The Gross Motor Function Classification System (GMFCS) system developed by Palisano, et al. focuses on children's functional achievements rather than on their limitations. It places emphasis on the child’s routine performance (not necessarily their best capacity) in the home or community setting.

The GMFCS system uses descriptions defining 5 levels of function that represent a broad ordinal scale where the distance between levels is not considered equal. Distinctions between levels are based on the need for assistive technology including mobility devices (such as walkers and wheeled mobility), and to a much lesser extent the quality of movements. Levels between 3 and 5 (the highest possible score) indicate progressively more serious limitations of gross motor function and that child is severely handicapped.[3] Table 6 shows the classification relevant to our study population.

Table 6. Summary of the Gross Motor Classification System for Cerebral Palsy. Adapted from Palisano et al., 1997.

Years	Level I	Level II	Level III	Level IV	Level V
0 ↓	- Able to sit up and balance while manipulating objects with both hands<br>- Crawls on hands and knees<br>- Begins to walk between 1.5-2yrs without assistance	- Maintains floor sitting but needs to use hands for support to maintain balance<br>- Creeps on stomach or crawls on hands and knees<br>- Pulls to stand & take steps while holding furniture	- Maintains floor sitting while low back is supported<br>- Rolls and creeps around on stomach	- Trunk support required for floor sitting<br>- Can roll to supine and may roll to prone positions	- Unable to maintain antigravity head and trunk postures while prone and sitting<br>- Requires assistance to roll
2 ↓ 4	- Able to move in and out of floor sitting positions and stand without assistance<br>- Walks as preferred method of mobility without assistive devices	- Maintains floor sitting but has difficulty with balance when both hands occupied<br>- Crawls, pulls to stand on stable surface & walks short distances with assistive devices	- Requires assistance to assume floor sitting, can maintain by "W sitting"<br>- Crawl on hands & knees, move using stable surfaces & walks short distances with assistive devices, adult assistance	- Floor sit when placed but unable to maintain balance & alignment without using hands for support<br>- Require mobility devices for sitting and standing, short-distance mobility achieved through rolling, creeping on stomach, crawling	- Voluntary control of movement and all areas of motor function restricted<br>- No means of independent mobility; require power wheelchair with extensive adaptations throughout life

The 2 year assessment will provide a window into early language development and early gross- and fine-motor development. We plan to submit further grants for long-term follow-up at 5 years of age, which correlates better with ultimate function.[188]

Modified Checklist for Autism in Toddlers (M-CHAT-R). Parent questionnaire (Appendix 3) will be administered to the mother/caretaker at the 2 year corrected age follow-up visit. This instrument is validated for screening toddlers between 16 and 30 months of age, to assess risk for autism spectrum disorders (ASD).

"The primary goal of the M-CHAT-R was to maximize sensitivity, meaning to detect as many cases of ASD as possible. Therefore, there is a high false positive rate, meaning that not all children who score at risk for ASD will be diagnosed with ASD. To address this, we have developed a structured follow-up interview for use in conjunction with the M-CHAT-R; it is available at www.firstsigns.org." Users should be aware that even with the follow-up questions, a significant number of the children who fail the M-CHAT-R will not be diagnosed with an ASD; however, these children are at risk for other developmental disorders or delays, and therefore, evaluation is warranted for any child who fails the screening.

The M-CHAT-R can be scored in less than two minutes. We will use the scoring template available at www.firstsigns.org. Children who fail more than 3 items total or 2 critical items (particularly if these scores remain elevated after the follow-up interview) will be referred for diagnostic evaluation by a specialist trained to evaluate ASD in very young children.

As the field of Neonatology has matured, more nuanced follow-up of NICU graduates has become available. It is now clear that preterm babies have a higher risk than do term infants for psychological and behavioral problems, with autism spectrum disorders and attention deficit problems identified specifically.[189-193] We will therefore screen all subjects with the M-CHAT-R parental questionnaire at the 2 year corrected age follow-up visit.[194] We anticipate positive screening will be higher in this ELGAN population than in term infants,[43] and if, as indicated in several MRI studies,[46, 193, 195-201] MRI imaging of brain structures correlates to psychological and behavioral function, we anticipate that Epo-treated neonates will have better preserved brain structure, and therefore, function.

6.3.3 Subject Retention. Additional contact to increase the likelihood of follow-up participation will include

sending birthday cards and appointment reminders. Follow-up rates of > 90% have been achieved in most of our study sites, using these methods. An offset for parents' travel/work loss expenses ($100) will be provided at the completion of the face-to-face follow-up visit at 2 years corrected age. An additional $100 per visit is included in the budget to provide for unusually high travel expenses when needed. We estimate this will be needed for 15% of subjects.

6.3.4 Intervention Discontinuation Evaluations

If a subject is removed from study treatment for any reason (parent decision, Attending physician choice, or because of SAE such as a symptomatic clot requiring anticoagulation), the reason for discontinuation will be recorded. Since this is an intention-to-treat study design, any subjects who discontinue intervention will continue to be followed and evaluated on study. Like all study subjects, these families will have a discharge questionnaire filled out, and will be contacted by phone at 4, 8, 12 and 18 months. They will be sent birthday cards for their baby. A small monetary incentive will be given to each family at the completion of the 2 year follow-up visit. For those individuals that must travel over 2.5 hours for the follow-up visit, overnight accommodations may be offered.

6.3.5 On Study/Off-Intervention Evaluations

The MRI should be done between 36-0/7 and 36-6/7 weeks of PMA. This will be done in a subset of 220 patients at study sites that have a 3T Siemens or 3T Philips magnet available.

6.3.6 Final On-Study Evaluations

Study drug treatment will end on the Monday, Wednesday or Friday closest to 32-6/7 weeks PMA, and each subject will be followed for 2 years, until their neurodevelopmental assessment at 22-26 months corrected age.

At the 2 year corrected age follow-up visits, documentation of the standardized neurologic exam, and results of the Bayley III exam will be recorded.

DOCUMENTATION AND RECORD RETENTION

Documentation. Each site must provide the UW CCC lead PENUT study coordinator, Stephanie Hauge, with the following documents prior to study initiation. A copy of these documents must be maintained in the site investigator's study files.

<u>IRB approved informed consent form</u> - All IRB approvals and correspondence (including approved revisions, protocol, advertisements, etc.) - Copies of all correspondence pertaining to the study (excluding any budgetary matters) - Copies of all serious adverse events submitted to the IRB - Copy of all safety reports.

The UW DCC co-supports an installation of REDCap, software specifically designed for electronic data capture that we have used successfully in other multi-site studies. REDCap features include differentiated user roles and privileges, password and user authentication security, electronic signatures, SSL encryption, and comprehensive auditing to record and monitor access and data changes (http://www.project-redcap.org/software.php). REDCap will serve as the architectural backbone for all data captured prospectively, with all data linked by study subject ID.

Record Retention. The clinical site is responsible for maintaining all records (i.e., case report forms, original data, screening logs, signed informed consent forms, correspondence, etc.) until notified, in writing, by the UW CCC, that these records are no longer needed. The Investigator must notify the UW CCC lead study coordinator if the site or records are relocated, if the investigator leaves the institution, etc., and a new address for the records must be provided.

6.3.7 Off-Study Requirements

When the subject has completed the 2 year corrected age follow-up visit, there are no further requirements for study participation. Participation in any further follow-up will require new consent. If consent is given, phone

follow-up will continue at 6 month intervals up to a maximum age of 5 years. At that point, we hope to enroll willing participants in a follow-up study that will follow the children up to 8 years of age.

6.4 Special Instructions and Definitions of Evaluations

Screen admissions to antepartum and NICU: Screening for eligible subjects will be done daily. For antepartum admissions, this will involve determining whether any admissions within the past 24 hours are likely to deliver imminently, and if so, whether they are within the gestational age criteria for the study. If both these criteria are satisfied, the Attending Perinatologist will be asked whether this patient might be an appropriate study candidate, and if so, whether it is permissible to approach the mother to discuss the study.

For neonatal admissions to the NICU, screening will involve determining the infant's time of birth and gestational age. If they qualify for the study, the Attending Neonatologist will be asked whether they meet inclusion (but not exclusion) criteria. If the baby meets criteria, the Attending will be asked whether it is permissible to approach the family to discuss the study. Once it is confirmed that the family is willing to hear about the study, they will be approached for informed consent.

A screening log will be completed for all screened patients.

6.4.1 Informed Consent

Antenatal consent will be obtained when feasible. Prenatally, the study investigator will obtain permission from the Maternal Fetal Medicine Attending to approach the mother to discuss the study. Postnatally, permission to approach the family will be obtained from the Attending Neonatologist. The Attending physician will seek parental agreement for an investigator to meet and discuss the study. If the parents are interested, the study investigator will discuss the study with family and seek consent in person. The consenting legal guardian will receive a copy of the consent form to review, and once signed, will be given a copy to keep. Ideally, if the Attending physician is also a study investigator, an alternate study investigator, or their designee, should obtain consent, so as to avoid the appearance of coercion. If this is not required by the site IRB, an investigator who is also the Attending physician may obtain consent. Investigators will only approach family after infant's attending heath care provider gives permission and family indicates that they are interested in further information about the study. No alteration in care will otherwise occur. The attending neonatologist and family can withdraw child from study at any time. If permissible by the site IRB, phone consent may be obtained, but must be reaffirmed with the family when they are present. Investigator should retain original signed document. Consent will be obtained by the Investigator in a room which ensures the privacy of the family, and which is free of potential coercive influences. Consent for participation must be obtained before the baby is 24 hours old.

If a family has limited or no English speaking abilities, a certified interpreter will be provided. They will review the consent form with the family, and interpret the verbal explanation of the study during the discussion between the Investigator and the family members. If individual sites have a large population of non-English speakers, consent forms will be translated into the appropriate languages. If an interpreter is not available in a timely manner, the family will not be approached.

The parents of the research participants will be given opportunity to review the study both verbally and in writing. The will be given opportunity to ask questions of the investigator prior to giving consent.

If there are changes in the protocol or safety information that require consent forms to be updated, they will be sent through the IRB process for approval. When entering a patient into the PENUT Portal electronic database, documentation of a signed consent form as well as a signed HIPAA form is required prior to randomization.

A model informed consent form is included as Appendix 1.

6.4.2 Documentation of Gestational Age will be done according to the following hierarchy, and the method by which gestational age was determined will be logged on the enrollment form:

1. Gestational age by in vitro fertilization if available.
2. Gestational age by first trimester assessment (0-14-0/7 weeks)
3. Gestational age by second trimester assessment (up to 28-0/7 weeks)
4. Last menstrual period (LMP)
5. Newborn maturational assessment

Documentation of intracranial hemorrhage. Screening Cranial Ultrasounds will be obtained as per the guidelines. Only the first ultrasound is done for study purposes. The subsequent two ultrasounds (or MRI at 36 weeks) will be done as part of routine clinical care. The Imaging Guidelines delineate how pathology will be documented and graded.

Documentation of clinical findings during the initial hospitalization will be done in REDCap after chart extraction after the first 6 doses of study drug have been administered, and within one month of discharge.

Documentation of neurodevelopmental follow-up will be at the time of the 2 year corrected age follow-up visit. A standardized neurologic exam and Bayley III exam will be done. The M-CHAT-R questionnaire will also be administered at this time.

6.4.3 Maternal demographics and history will be obtained and documented on the demographics and maternal history forms after consent is obtained for participation in the study.

6.4.4 Treatment history. Not applicable since subjects are newborns

6.4.5 Concomitant treatments while the subject is in the NICU will be documented.

6.4.6 Study Intervention Modifications. Study drug and/or iron supplementation will be held or stopped for the following criteria:

- **Polycythemia:** Central hematocrit (Hct) > 65%: Study drug should be held until Hct is ≤ 55%.
- **Severe sepsis**: Blood culture-proven bacterial or fungal sepsis requiring blood pressure support or new respiratory support. Supplemental parenteral iron *should be held until blood culture is negative for 72 hours.* Rationale: Iron has been reported to be permissive for selected gram-negative bacteria and might worsen the patient's condition during sepsis. **There is no known relationship between Epo and sepsis**, so study drug does not need to be held.
- **Unexplained recurrent seizures** (unrelated to ICH, PVL or other known pathology): Study drug should be held until seizures are well controlled by medication. Restarting study drug will be determined by the Medical Monitor, DSMB, and CCC.
- Major **venous or arterial thrombosis (clot).**
- Study drug should be held for any thrombosis that is treated with anticoagulation, and this should be reported to the CCC PI as an SAE.
- Study drug should be held for any **symptomatic** thrombosis involving a major vessel (e.g. symptoms such as superior vena cava syndrome)
- **Sustained hypertension *requiring medical intervention:*** Study drug should be discontinued if blood pressure requires treatment. When blood pressure returns to normal range (systolic blood pressure < 100 mmHg), study drug can be resumed. This is true even if patient is still being treated, but blood pressure is being controlled.
- If the subject requires prolonged antihypertensive therapy (> 1 month) and/or will be discharged on medication this is considered an SAE. Medications used to treat hypertension will be recorded. Any work up done to investigate causes of hypertension will be recorded.

6.4.7 Clinical Assessments.
Clinical safety parameters
1) Complete physical exam will be done on study entry, after the first 6 doses of study drug (day 12-15) and at discharge. Growth parameters (head circumference, height and weight) will be obtained at these times. The presence of microcephaly (OFC < 10th percentile) or relative microcephaly (discrepancy of > 50 percentile between weight, length and head circumference) will be recorded.

2) Vital signs and blood pressure. All ELGANs are monitored continuously for heart rate and arterial saturation (pulse oximetry), with either continuous or intermittent blood pressure readings as part of routine care in the NICU. The high and low blood pressure will be recorded for the first 14 days of the study, and then BP measurements will be recorded at 6 set time points through discharge.

3) *Hematologic data*. Complete blood count (CBC) including hematocrit, white blood cell count, absolute neutrophil count, platelet counts and blood smear are obtained routinely in extremely preterm neonates as part of their care. This is done to evaluate for infection, and to evaluate the need for transfusion (ELGANs receive an average of 4 transfusions during their first month of life). Results of these tests will be recorded weekly, as available, through 36-6/7 weeks PMA or hospital discharge. The number and volume of blood transfusions, donor exposures, and phlebotomized blood volume will be recorded for the entire hospital stay. Ferritin or ZnPP/H will be followed to monitor iron status. While important for all preterm newborns, this is strongly recommended for infants receiving parenteral iron. Iron supplementation will be modified based on these results (See Appendix 7 Iron Guidelines).

4) *Renal function.* Daily weights, and fluid intake and output will be recorded as available in the chart. BUN and creatinine will be recorded as available in the chart. If renal ultrasounds are done for clinical indications, the results from these studies will be recorded. Ten urine samples will be collected non-invasively at timed intervals from the time of enrollment until discharge.

5) *Liver function*. Conjugated bilirubin will be recorded weekly as available, through 36-6/7 weeks PMA. These data are checked routinely on all infants requiring parenteral nutrition support, and as part of routine assessment of nutritional status.

6) *Respiratory data*. Respiratory complications of prematurity will be documented, including respiratory distress syndrome, pulmonary hemorrhage, pneumothorax, pneumonia, duration of mechanical ventilation (> 1 day, > 1 week), and oxygen use at 28 days and BPD at 36 weeks PMA.

7) *Complications of extreme prematurity* defined as follows will be recorded:

- Bronchopulmonary dysplasia (BPD). BPD will be defined at 36 ± 1 weeks corrected gestational age. Infants requiring nasal cannula oxygen, CPAP/high flow nasal cannula or mechanical ventilation will be considered mild, moderate or severe BPD, respectively.

- Necrotizing Enterocolitis (NEC). Bell's staging criteria will be used to define NEC. All surgeries for NEC, and for strictures or bowel obstructions occurring as sequelae of NEC, will be recorded. NEC stage II or III is considered an SAE.

- Retinopathy of prematurity (ROP). All infants will be followed using the screening recommendations published in 2006.[202] The international classification of ROP will be used.[203] Severe ROP requiring intervention is considered an SAE

- Intracranial Hemorrhage (ICH), white matter injury (WMI) or hydrocephalus (HC). A cranial ultrasound will be done prior to the first study drug dose as part of the study. Brain imaging will also be done as part of

routine care at 7-10 days and 36 weeks PMA (30 days of age or later is acceptable). The presence, location and extent of any intracranial bleeding, hydrocephalus or periventricular echolucencies/densities will be documented. Grade III and IV bleeds will be considered an SAE.

- Clinical seizures. If clinical seizures are suspected, an EEG will be done to confirm the diagnosis at the Attending physician's discretion. All such results will be recorded.

  - **Sustained hypertension *requiring medical intervention:*** Study drug should be discontinued if blood pressure requires treatment. When blood pressure returns to normal range (systolic blood pressure < 100 mmHg), study drug can be resumed. This is true even if patient is still being treated, but blood pressure is being controlled.

  - If the subject requires prolonged antihypertensive therapy (> 1 month) and/or will be discharged on medication this is considered an SAE. Medications used to treat hypertension will be recorded. Any work up done to investigate causes of hypertension will be recorded.

- Patent ductus arteriosus that is treated with either medical or surgical intervention. The indications for treatment will be recorded (ECHO parameters and/or clinical symptoms). Age at treatment will be recorded.

- Sepsis, confirmed by positive blood culture, or presumed (clinical symptoms present and antibiotic treatment instituted for 7-10 days despite negative cultures). Culture proven sepsis associated with shock or significant respiratory deterioration is an indication to hold parenteral iron (not study drug).

- Renal (acute or chronic renal failure) and hepatic complications (cholestasis, hepatitis).

- Thyroid function (TSH and free T4) will be collected as available.

- Mortality. The timing and circumstances of any deaths in this study population will be recorded and reviewed by the DSMB.

Data on medication administration, including use of steroids, methylxanthines, antibiotics, diuretics, vasopressors, antihypertensive agents, sedatives, anticoagulants, anticonvulsants, thyroid treatment, and indomethacin/ibuprofen will be collected.

8) *Blood samples*. Blood (0.5 mL/sample) will be obtained through an indwelling umbilical arterial or venous catheter when possible. If no access is available, all efforts will be made to combine the blood draw with clinically indicated phlebotomy times. The timing of blood draws is shown in Figure 3. An additional (optional) blood sample will be drawn at the 2 year follow-up visit.

The CCC will provide sample collection packs to each site. These will contain green top tubes for blood collection, a microfuge tube for plasma collection, and labels for subject identification and date. Samples will be spun, and plasma and cell pellet frozen at -70°C to -80°C. Batched samples from each site will be sent to Dr. Juul's lab at the University of Washington via FedEx.

Biomarkers. Plasma from these samples will be used to investigate the effect of Epo on inflammatory mediators and growth factors, as well as Epo pharmacokinetics.

Sample storage. Samples will be spun on site to separate plasma from cells. Plasma and cells will be stored in separate, labeled containers at -70ºC to -80°C. They will be sent to the UW in batched quantities, after every 4-5 subjects. The stored cells for each subject will be kept for later study of problems related to prematurity that

are beyond the scope of this study.

Urine samples will be banked at the University of Washington for future analyses of biomarkers, (neutrophil gelatinase associated lipocalin (NGAL), osteopontin (OPN), cystatin C (CysC), uromodulin (UMOD), epithelial growth factor (EGF) and urine β2-Microglobulin (β2MG)).
9) *MRI* will be done at selected sites at 36-0/7 to 36-6/7 weeks PMA using a predefined protocol. If an MRI is needed for clinical indications during this time frame, and if the appropriate protocol is utilized, it may be used for the purposes of the study

10) *Neurodevelopmental assessment* will be completed on each subject at 22-26 months corrected age:

- Bayley III Scales of Infant Development: Composite Language and Composite Motor Scale in 5 domains - cognitive language (receptive, expressive), motor (fine and gross), social-emotional, and adaptive.[176]
- Modified Checklist for Autism in Toddlers (M-CHAT-R) (Appendix 3)
- Standardized neurological examination (Appendix 3)
- The Gross Motor Function Classification System (GMFCS)

6.4.8 Epo pharmacokinetics. Epo concentrations will be obtained at four time points as outlined in Figure 3, section 5.1. Population Pharmacokinetic Analysis will be done, and Epo concentrations will also be used to document study adherence for subjects assigned to the Epo (and control) arms. Plasma for inflammatory mediators and biomarkers of brain injury will be obtained at the same blood draw at baseline and study days 7 and 14.

7.0 MANAGEMENT of ADVERSE EVENTS

All complications will be treated in the site NICUs.

8.0 CRITERIA FOR INTERVENTION DISCONTINUATION

The attending neonatologist or parent may withdraw the infant study subject at any time for any reason. The reason for such withdrawal will be recorded. The study interventions will be discontinued in any infant subject who suffers a SAE due to Epo administration. The research coordinator and study investigator will evaluate all subjects on an ongoing basis for evidence of Epo SAE occurrence.

Criteria for Temporarily Withholding/Stopping Study Drug and/or Supplemental Iron.

1. **Polycythemia:** Central hematocrit (Hct) > 65%: Study drug should be held until Hct is ≤ 55%.
2. **Severe sepsis**: Blood culture-proven bacterial or fungal sepsis requiring blood pressure support or significant new respiratory support. Supplemental iron *should be held until blood culture is negative for 72 hours.* Rationale: Iron has been reported to be permissive for selected gram-negative bacteria and might worsen the patient's condition during sepsis. **There is no known relationship between Epo and sepsis**, so study drug does not need to be held.
3. **Unexplained recurrent seizures** (unrelated to ICH, PVL or other known pathology): Study drug should be held until seizures are well controlled by medication. Restarting study drug will be determined by the Medical Monitor, DSMB, and CCC.
4. Major **venous or arterial thrombosis (clot).**

Study drug should be held for any thrombosis that is treated with a course of anticoagulation.
Study drug should be held for any **symptomatic** thrombosis involving a major vessel (e.g. symptoms such as superior vena cava syndrome).

5. **Sustained hypertension *requiring medical intervention***: Study drug should be discontinued if blood pressure requires treatment. When blood pressure returns to normal range (systolic blood pressure < 100 mmHg), study drug can be resumed. This is true even if patient is still being treated, but blood pressure is being controlled.

All necessary medical interventions will be available in the event of serious adverse events stemming from a subjects involvement in research. All serious adverse events will be monitored closely until resolution, and they will be recorded and reported to the local IRB (as required) as well as the CCC PI (Dr. Juul) who will then notify the Medical Monitor as well as the DSMB and NINDS (via Peter Gilbert). In the event that a subject is withdrawn from the study for any reason, all efforts will be made to encourage parents to allow their child to continue to participate in follow-up visits.

9.0 STATISTICAL CONSIDERATIONS

9.1 General Design Issues

Hypothesis: Epo treatment from 24 hours to 32-6/7 weeks postmenstrual age (PMA) of Extremely Low Gestational Age Neonates (ELGANs) will safely decrease the combined outcome of death or neurologic impairment from 40% to 30% measured at two years of age.

Design: We will enroll 940/2 = 470 subjects in each of two treatment groups: Epo-treated (from 24 hours of age to 32-6/7 weeks PMA) vs. placebo control.

Randomization sequences will be created centrally by the DCC. We will use block randomization within site using variable blocks of size 4, 6, 8 and 10 subjects. Using block randomization ensures that equal numbers of subjects are randomized to the intervention and control arm and that the two groups are balanced at period enrollment intervals. For multiple births (twins, triplets) all infants will be randomized to the same treatment group (e.g. effective randomization of the mother).

Randomization will be stratified on site, gestational age category (24-25 weeks, 26-27 weeks), and on the number of babies inborn for a given pregnancy (one, two, three or more). Randomization sequences will be provided to the research pharmacy at each site through a study binder. The binder will contain the complete set of study IDs and associated randomized assignments and will be utilized as a look-up table over the course of the study and for each patient's protocol administration of the study drug. Patient study IDs will utilize the following format: <3 digit site code>-XXX-YY, where XXX is the mother ID and YY is the baby ID within mother (01, 02, 03… etc.). Study IDs within the binder will be listed by site and mother ID. Singletons, twins and triplets are stratified by gestational age as shown in Table 7. For example, twin infants from mother 201 at site UOW will have study IDs of UOW-201-01 and UOW-201-02. Using Table 7.1 to look up the mother ID UOW-201, these two infants will be assigned to receive Epo over the course of the study protocol.

Table 7. Randomization and stratification

	24-0/7 to 25-6/7	26-0/7 to 27-6/7
Singletons	100-YY	400-YY
Twins	200-YY	500-YY
Triplets	300-YY	600-YY

Table 7.1. Example study drug look-up table in Pharmacy study binder.

Singleton, 24-25 weeks gestation			Twins, 24-25 weeks gestation			Triplets, 24-25 weeks gestation		
ID	Baby	Treatment	ID	Baby	Treatment	ID	Baby	Treatment
UOW-101	-01	Epo	UOW-201	-01	Epo	UOW-301	-01	Placebo
UOW-102	-01	Placebo		-02			-02	
UOW-103	-01	Epo					-03	
UOW-104	-01	Placebo	UOW-202	-01	Placebo	UOW-302	-01	Epo
UOW-105	-01	Placebo		-02			-02	
UOW-106	-01	Epo					-03	

Singleton, 26-27 weeks gestation			Twins, 26-27 weeks gestation			Triplets, 26-27 weeks gestation		
ID	Baby	Treatment	ID	Baby	Treatment	ID	Baby	Treatment
UOW-401	-01	Epo	UOW-501	-01	Epo	UOW-601	-01	Placebo
UOW-402	-01	Placebo		-02			-02	
UOW-403	-01	Epo					-03	
UOW-404	-01	Placebo	UOW-502	-01	Placebo	UOW-602	-01	Epo
UOW-405	-01	Placebo		-02			-02	
UOW-406	-01	Epo					-03	

A modified intent-to-treat (mITT) approach[204] will be used, with all randomized infants who receive the first dose of study treatment to be included in the analysis. All pre-specified hypotheses will be tested using a two-sided type I error of 0.05 with no formal adjustment for multiple comparisons unless otherwise specified (such as with safety outcomes). Secondary analyses that focus on separate hypotheses will not require correction for multiple comparisons, but those analyses that use multivariate measures such as multiple brain image parameters would be corrected for multiple comparisons using standard methods.

Given that we anticipate enrollment of multiple births we require that all analyses properly account for the within-sibship correlation of outcomes. We will use Generalized Estimating Equations (GEE), which is a versatile regression approach for the analysis of discrete and continuous outcomes.[205] Use of "robust" standard errors will provide valid statistical inference and fully account for the clustering of data.

9.2 Outcomes

Primary outcome variable: The primary outcome is the composite outcome of death or neurodevelopmental impairment at 22-26 months corrected age. Neurodevelopmental impairment (NDI) is defined as the presence of any one of the following: CP, Bayley III cognitive or motor scale < 70. There is a known inflation of scores from the Bayley II to III[1, 178, 179] and we will therefore also consider a threshold of < 85 for secondary analysis. Subjects will be stratified by gestational age (24-0/7 to 25-6/7 and 26-0/7 to 27-6/7), number of babies in the pregnancy, and by study site. Table 8 shows how CP will be categorized based on features present on standardized neurologic exam.

Table 8. Motor outcome - 4 level classification

	GMFCS						
	**0**	**0.5**	**1**	**2**	**3**	**4**	**5**
**No CP**	None	None	Mild	Moderate	Severe	Severe	Severe
**HP or DP**	Mild	Mild	Moderate	Moderate	Severe	Severe	Severe
**QP**	Moderate*	Moderate*	Severe*	Severe	Severe	Severe	Severe

** It is unlikely that a child with quadriparetic CP will have a GMFCS of 0-1. However, this scenario is possible in cases of bilateral hemiparesis in which arms are more affected than legs. In such cases, the bilateral nature of the deficit, and the significant neurologic abnormalities that are noted on a standardized neurologic examination, warrant a designation of moderate/severe neurodevelopmental impairment.*

QP: quadriplegic; HP: hemiplegic; DP: diplegic

Primary Analysis: The primary analysis will be a test of equality of the rate of the primary outcome (death or NDI) across the two randomized investigational groups. Specifically, we will use a GEE Wald test based on logistic regression, with stratification by recruitment center, multiples in gestation and gestational age. We will perform intent to treat analysis and expect minimal non-compliance due to the nature of the intervention in relation to in-patient care. For the primary endpoint we expect uniform and complete ascertainment of death but may not evaluate all subjects for developmental impairment. We plan to perform a primary analysis based on complete cases and will exclude those subjects for whom vital status is known (alive) but NDI cannot be assessed. Sensitivity analysis will use multiple imputation to evaluate the potential impact of any missing data. Secondary analysis will be for quantitative measures of brain volume, and for these endpoints an unadjusted t-test provides inference regarding the mean response across the treatment groups. We will adjust all secondary outcome analyses for recruitment site using regression methods.

Power and Sample Size for Primary Outcome: In order to determine the necessary sample size for efficacy evaluation, we need to formulate assumptions for the primary outcome rate in the treated and untreated groups.

The primary outcome measure is the rate of death or severe neurodevelopmental impairment (NDI). Using data from two sources, we can compute the expected rates of death or NDI for the neonates that we will enroll. The Vermont Oxford Network 2008 Follow-up Report[206] evaluated the disability status of infants born in 2008 only, and the combined 2004-2008 cohorts. Follow-up status was determined at age 18-24 months and information regarding death and NDI is provided for subgroups of children based on their gestational age. Therefore, we can use these data to forecast expected trial results for our eligible subjects (24-27 weeks gestational age).

The VON reports on severe NDI and specifically states that components of severe disability include: cerebral palsy, or a Bayley score less than 70 or too severely delayed for Bayley testing.[206]

In addition, data from Gargus et al. (2009)[19] provides follow-up information at 18-22 months for approximately 3,800 neonates born between 24 and 27 weeks gestational age, based on babies born between 1998 and 2001.

We have combined the VON and Gargus (2009) data in order to determine the anticipated characteristics of our proposed trial. While the VON data is contemporary, it has relatively low follow-up for two-year outcomes (approximately 50%) and therefore may be biased toward more easily followed or more favorable subjects.

Table 9 displays our best estimates of gestational age specific and overall rates of death and NDI.

Table 9. Combined Vermont Oxford Network (VON) and Gargus (2009) data showing death rates and severe neurodevelopmental impairment (NDI) rates for different gestational ages (GA).

Gestational age (weeks)	Fraction of enrolled	Death	NDI	Total (Death+NDI)	Expected Treated (Death + NDI*0.45)
24	0.25	32	25.0	54.4	44.8
25	0.25	19	24.2	40.1	32.3
26	0.25	13	21.2	30.4	24.7
27	0.25	9	18.1	24.5	19.0
**Overall**		**18.2**	**22.1**	**40.4**	**30.4**

Our assumed death rates of 32, 19, 13 and 9 percent are exactly those presented in the VON 2008 report, and are lower than the rates reported in Gargus (2009) where among neonates born between 1998 and 2001, GA-specific death rates of 45, 25, 18, and 15 percent were observed. Using our combined data assumptions, we expect an overall death rate of approximately 18%, which is approximately the rate observed in VON (17%), and is lower than the 26% death rate observed for Gargus (2009).

In addition, Gargus (2009) report GA-specific rates of NDI as 21, 25, 23, and 19 percent. The VON 2008 report estimates NDI rates as 22, 21, 17, and 16 percent respectively. We have combined VON and Gargus (2009) data and further assumed that NDI rates are decreasing with increasing gestational age to obtain our NDI estimates. Using these data we expect an overall NDI rate of 22%, which equals the overall rate observed in the VON 2008 report and in Gargus (2009).

In order to estimate the overall rate observed among treated neonates, we have assumed that there will be no effect of treatment on death, but that Epo will lead to a decrease in the rate of NDI. If we assume a multiplicative reduction in the NDI rate of 0.45 then we expect a treated NDI rate of 12 percent and an overall rate of death+NDI of 30.4% as compared to the control rate of 40.4%. Therefore, in order to obtain a target sample size we assume: an overall control rate of 40%, and an overall treated rate of 30% corresponding to an overall treatment rate ratio of 0.75.

Using the control and treated rates of 40% and 30% respectively leads to a sample size of 376 evaluated subjects per arm or a total evaluated sample size of 752 subjects.

Efficacy Trial Statistical Analysis Plan – Secondary Outcomes

Secondary Aims and Outcomes:

- To compare safety measures between infants receiving Epo and placebo to determine whether there are risks associated with Epo administration.
- To compare moderate impairment (< 85 on Bayley III cognitive or motor score, or CP).
- To compare neuroimaging outcomes across the two treatment groups.
- To assess whether treatment effects vary by gender.
- To evaluate whether individual biomarkers measured through 36 weeks are predictive of 2-year outcomes, and whether a derived multivariate combination of markers has predictive performance greater than individual marker performance.

Analysis for Secondary Aims and Outcomes:

Safety: The primary safety outcomes are the serious adverse events and adverse events listed in the CRFs.

Analysis will compare the proportion of subjects with an AE across the two treatment groups and will use a 2-sample test of proportions. Inference for the (5) individual SAE outcomes will use a Bonferroni correction for multiple comparisons.

Power: The expected prevalence of SAEs range from less than 1% for polycythemia, to 18% for death. In order to characterize power we show the effect sizes for which we have 80% power based on having n=376 subjects evaluated in each group:

Table 10.

Baseline (control) rate	Alternative rate for 80% power Using alpha=0.05	Using alpha=0.01
5%	11% (RR = 2.2)	13% (RR = 2.6)
10%	18% (RR = 1.8)	19% (RR = 1.9)
15%	24% (RR = 1.6	25% (RR = 1.7)
20%	30% (RR = 1.5)	31% (RR = 1.5)

Secondary Efficacy Outcome: Our key secondary long-term outcome is the rate of death or NDI using severe or moderate impairment defined by a Bayley less than 85. In order to estimate power for the secondary outcome we assume the same overall rates for death and severe NDI as above, and then assume a 20% rate for moderate impairment. Similar to the primary outcome, we assume that the death rate is unchanged by Epo and that the rate of severe NDI is reduced from 22% to 12%. We also assume that the moderate NDI group would have an effect similar to the severe group where the 20% rate would be reduced by a factor of 0.45 to

	**Control**	**Treated**
Death	18%	18%
Severe NDI	22%	12%
Moderate NDI	20%	16% = 11% remain mod + 5% from severe
**Total**	60%	46%

11% but that among the 10% of severe subjects impacted by Epo, half of these would only move from severe to moderate impairment. Therefore we assume:

Power: Using these assumptions and the target evaluated of n=752 leads to greater than 95% power to detect a difference on the secondary outcome.

Imaging Outcomes: The neuroimaging outcomes are: Myelinated white matter volume; Total gray matter volume; and White matter integrity (TBSS FA corpus callosum). We will conduct a single MANOVA test using the multivariate outcome and comparing treated and control subjects. The mean and standard deviation will also be calculated (by treatment group) for each individual measure. Note that MR measures will be obtained on a subset of infants (110 per treatment group).

Given the *a priori* hypothesis that treatment effect may differ according to gender we will conduct a single subgroup analysis that assesses treatment effects separately for males and for females. Subgroup specific treatment effects will be computed and inference will be based on a single Gender-by-Treatment test for interaction using logistic regression.

Power: Multiple MRI variables will be collected and analyzed in this study. To provide a basic sample size for a treatment effect we selected one common basic measure of structural injury that has been identified in the literature for the premature neonates that we hypothesize will be preserved: The size of the cerebellum tissue

when compared to the whole head volume. We used a comparable set of T1 weighted brain scans of 22 premature babies aged between 34 and 36 PMA, with a representative range of brain injuries present on MRI and applied the proposed segmentation methods to the data. We estimated the ratio of the cerebellum to whole brain size as our marker across this group and used the mean and variance of these measures on which to base our power calculation for a treatment effect. Assuming age matched groups in the treated and untreated groups, from this data we estimate that 110 subjects will be required to see a 5% increase in the cerebellum size. The recognition of cerebellar atrophy in extremely preterm infants has only recently come to light with the increased use of MRI.[193, 207-212] Cerebellar atrophy has been linked to cognitive as well as motor deficits.[209, 213, 214] In summary, we will focus on four primary measures that are established to be predictive of 2-year neurodevelopmental status and therefore can be used to establish support for the hypothesis of long-term benefit of Epo treatment. The neuroimaging biomarkers are: 1) myelinated white matter volume; 2) total gray matter volume; 3) cerebellar volume and 4) white matter integrity assessed by TBSS (using FA corpus callosum).

Biomarker Analysis: We will consider two main classes of potential predictors of 2-year status: neuroimaging measures and inflammatory markers. Interest is in the prognostic potential of individual and/or combined biomarker measurements. Given that the primary outcome is a binary measure (NDI), we will evaluate the predictive potential of individual quantitative measures using ROC curves showing the full potential of sensitivity and specificity across marker cut points. We will compute ROC curves for the (4) primary neuroimaging measures, and separately for individual inflammatory markers. Only 220 subjects will have data on the inflammatory markers, and these will be the same subjects identified for MR measures. We will derive two multivariate predictive models: using the inflammatory markers; and using the MR measures. We will use AIC and 10-fold cross-validation to develop and validate predictive models. A final multivariate model will combine markers from both MR and inflammatory measures, and 10-fold cross-validation will permit inference in the incremental value of adding markers in combination by comparing ROC curves and associated area under the ROC curve (AUC).

9.3 Meeting Recruitment Targets

We have chosen sites for this trial that can be expected to enroll a minimum of 24 infants/year in the study. We plan to recruit at 18 sites, several of which have participated in the NO CLD and TOLSURF studies. Based on our experience to date with these studies, we anticipate that the participating sites will meet their enrollment targets and that subject accrual will follow the projected timeline. We have provided very conservative estimates of enrollment, recognizing that some sites will do better, and some worse than predicted. Accordingly, we will assess site enrollment every 6 months after initiation of the study. Should enrollment at an individual site fall below 6 in a 6 month period, the UW CCC PI and Executive Committee will give warning and evaluate whether it is appropriate to drop the site from the trial. If no patients are entered during a 6 month period the Executive Committee will recommend that the site be dropped and a new site recruited. If a single site appears to be enrolling more than 20% of the total infants enrolled in the trial we will temporarily limit enrollment at that site.

In order to obtain the target number of evaluated subjects we will recruit from 18 centers. Our current centers are listed below in Table 11, with numbers estimated based on the previous 2 years of admissions. If these 18 sites are insufficient to maintain an average of 204 subjects enrolled per 6 month period, additional sites will be added. This will be monitored closely.

Table 11. Subject Availability at Each Enrolling Site

Site	# admitted < 27-6/7 wks	# eligible	# survive	% enrolled	% followed	# Total (2.5 years)
Beth Israel Deaconess Hospital, Boston	47	41	40	0.5	93	47

Children's Hospital of the Univ of Illinois	57	50	42	0.5	90	47
Children's Hospital of Minnesota, St Paul	55	41	39	0.5	85	41
Children's Hospital of Minnesota, Minneapolis	99	80	70	0.5	96	84
University of Minnesota Children's Hospital	52	48	46	0.5	100	46
Maria Fareri Children's Hospital, New York	110	88	72	0.5	90	81
Methodist Children's Hospital, San Antonio	64	56	51	0.5	80	51
Prentice Women's Hospital, Chicago	87	83	70	0.5	93	81
University of Florida, Gainesville	100	85	68	0.5	85	72
Florida Hospital for Children, Orlando	75	57	56	0.5	80	56
University of Louisville, Kentucky	94	86	71	0.5	90	80
Univ of New Mexico Children's Hospital	50	25	22	0.5	100	28
University of Arkansas, Little Rock	120	100	80	0.5	90	90
University of Utah, Salt Lake City	68	54	49	0.5	90	55
University of Washington, Seattle	87	69	58	0.5	85	62
Wake Forest School of Medicine, Winston-Salem	120	100	89	0.5	92	102
Johns Hopkins Medical Center, Baltimore	87	80	70	0.5	90	63**
South Miami Hospital, Miami	47	42	40	0.5	90	36***

Hopkins joined study in Feb 2015; South Miami joined study in June 2015
**Over 1.8 years; ***Over 1.5 years

In both the phase I/II Epo trial of extremely low birth weight infants and the NEAT trial (Epo neuroprotection of term infants with hypoxic ischemic encephalopathy), consent was obtained in over 90% of approached parents. This extremely high consent rate reflects the concern parents have about their children's neurodevelopment, and their willingness to participate in research that might result in improved outcome.

Site performance will be tracked with both enrollment and follow-up tables. Examples are shown below.

EXAMPLE ENROLLMENT AND FOLLOW-UP TABLES

Shell Table 1. Open Report: Cumulative and Current Report Period Study Recruitment.

	Current Report Period			Cumulative		
Site	Screened	Eligible	Enrolled	Screened	Eligible	Enrolled
Site-Name-1						
…						
Site-Name-18						
Total						

Shell Table 2. Open Report: Follow-up rates by site, n / N (%).

Site	4 month	8 month	12 month	18 month	24 month
Site-Name-1					
…					
Site-Name-18					
Total					

9.4 Data Monitoring

Per NIH Guidelines (1998), this Data and Safety Monitoring Plan (DSMP) defines the oversight and monitoring activities that will ensure and maintain both the safety of participants and the scientific integrity and validity of the trial data. The plan also describes the procedures for adverse event reporting and detailed guidelines for recommendations related to trial continuation.

PENUT is a multi-center randomized placebo-controlled phase III trial to determine whether recombinant human erythropoietin (Epo) will safely improve the long-term neurodevelopmental outcome of preterm infants 24-0/7 to 27-6/7 weeks of gestation. The PENUT study will randomize n=940 subjects to receive either Epo or placebo.

The DSMB will monitor the rates of these comorbidities at 6 month intervals, comparing the rates in treated and control infants. Expected rates will be based on the published literature. For example, in the Network trial, 93% had RDS, 46% PDA, 36% had any ICH with 16% severe, 11% NEC, 36% late-onset sepsis, and 12% ROP. In the ELGAN study, 21% died by 2 years of age, 24% of all children had an ICH, 12% had moderate/severe ventriculomegaly, and 24% had an ultrasound lesions (echodense or lucent).[134]

Safety Analysis: For each SAE we will tabulate the event rate by treatment group, and then compare rates using Fisher's exact test. The DCC will prepare monthly summaries of reported SAEs for review by the Medical Monitor, the CCC PI and NINDS.

9.4.1 Data and Safety Monitoring Board (DSMB)

Review of the PENUT trial's study performance and safety outcomes will be conducted by the Data and Safety Monitoring Board (DSMB) as required by the NIH for multi-site clinical investigations. The DSMB consists of 5 members with a designated chair (Ronnie Guillet), and safety officer (Jack Widness). Committee membership includes expertise in both biostatistics and bioethics, and should have clinical expertise appropriate for the intervention and target population. The DSMB is expected to meet two times (every 6 months) per year to review study performance and safety outcomes in Open and Closed Reports, and to review study enrollment reports quarterly. Ad hoc sessions may be scheduled as required should a serious adverse event need to be reviewed by the group.

Aggregate safety and efficacy data and study performance monitoring data will be presented during the open sessions of DSMB meetings. Blinded safety and efficacy data will be presented by treatment arm during the closed sessions. Review by the DSMB provides assurances that the trial can continue without jeopardizing patient safety. The DSMB is also responsible for protecting the confidentiality of the trial data and for monitoring the quality of both the data and study implementation procedures.

The DCC will work closely with the CCC in developing and implementing a comprehensive system for safety monitoring. Safety monitoring includes the systematic review of safety data for trends that may impact patient safety. The processing, reviewing, and reporting of adverse events (AEs) and serious adverse events (SAEs) are also part of this process. The infants in this study are hospitalized at study entry, and the duration of their hospitalization is highly variable and not predictable at birth. It ranges from as short as three months to more than a year.

Data and Safety Monitoring Schedule: Our target enrollment is n=940 which is expected to accrue during the first 2.5 years of the trial. Therefore, we will enroll approximately 200 subjects every 6 months. Our planned DSMB safety analyses will occur every 6 months after trial initiation.

9.4.2 Monitoring Guidelines

Based on findings following review of study data by the DSMB, the Board may recommend: continuation of the trial, termination of the trial, or modifications to the protocol (e.g. adding new measurements for safety

monitoring, discontinuing high risk subjects, extending the trial in time, increasing the trial sample). Decision guidelines in the PENUT trial are based on group differences in adverse event rates as explained below, and on whether SAE rates exceed pre-determined thresholds.

9.4.2.1 Performance Monitoring
Performance monitoring will be an ongoing activity performed by the study principal investigator and statistician, and status reports will be reviewed by the DSMB during their regular meetings. Procedural reviews to address protocol compliance with respect to subject recruitment and eligibility, retention and follow-up, randomization and blinding and quality of data will be conducted and monthly reports generated. Any protocol violation that affects patient safety will be reported to the DSMB immediately.

Performance data will be reviewed in aggregate and by site. It is expected that:

- The response rate for determination of the primary outcome (e.g. 24 month Bayley) will be no less than 80%;

- Missing interviews will be no greater than 15% at the 4, 8, 12 and 24 month follow-ups;

- The overall enrollment rate will not drop below the expected rate (34 subjects per month for 24 months) by more than 25%.

Data will be entered into a central trial management system utilizing a REDCap database hosted at the DCC. Data will be entered into fields with automated validation and logical check built in. Data will be double entered on the outcome measures of Bayley III and CP assessment by standardized neurologic exam. Compliance will be assessed based on the weekly conference calls and data submitted to the DCC on a weekly basis. If it is determined by the study PI that either 1) study protocol is not being followed, or 2) that reporting is inadequate at any site, further action will be taken to address these issues. These actions may include additional in-person site visits if appropriate or additional educational/problem-solving sessions by phone or in person regarding the study protocol.

9.4.2.2 Safety Monitoring:
The research coordinator at each site will monitor each subject daily for the presence of any complications until discharge. Serious adverse events will be brought to the attention of the CCC PI (Dr. Juul) who will report them to the Medical Monitor (Jack Widness), DSMB and NINDS (via Peter Gilbert). Sites will notify their local IRBs of SAEs per site institutional requirements. An independent Medical Monitor will review all cases of serious adverse events. A potential risk that is unique to preterm infants is the risk of ROP.[121] In the published studies of preterm neonates receiving potentially neuroprotective doses of Epo, no difference has been noted between treatment and control groups.[33, 35, 114, 115]

As part of the Data and Safety Monitoring Plan (DSMP) we will perform continuous and interim analysis of accruing safety data. We have defined potentially treatment (Epo) related serious adverse events (SAEs) that will be monitored throughout the course of the study. Specifically, for SAEs, we will compare absolute rates to expected rates based on published data for similar newborns, and will seek careful DSMB review and guidance when observed rates exceed pre-specified thresholds. In addition, at planned interim analysis we will formally compare the event rates across the two treatment groups using appropriate small sample methods such as Fisher's exact test.

9.4.2.3 Data and Safety Monitoring Schedule.
See section 9.4.3.1, Table 14.

9.4.2.4 Treatment Efficacy Monitoring
Treatment efficacy will be monitored by the DSMB, but recognizing that 24 month efficacy outcomes will not be

obtained until after enrollment is complete (or nearly complete). Therefore, although formal interim analyses will be conducted using group-sequential boundaries there may not be compelling reasons to terminate the trial on the basis of accruing efficacy data. Efficacy measures will be provided in a blinded fashion to the DSMB with treatment groups labeled “A” and “B” as well as a z-score assessment of the evidence for the difference between treatment arms at 24 months. An O’Brien-Fleming interim stopping boundary will be provided to the DSMB to help assess the magnitude of the z-score.

9.4.3 Scheduled Reporting

One month prior to each DSMB review, the Data Coordinating Center (DCC) will summarize monthly administrative reports that describe study progress including subject accrual by site, demographics, and the sites’ adherence to inclusion/exclusion criteria and other protocol requirements, including retention rates at each follow-up point. These reports are prepared monthly and reviewed internally by the study research team for ongoing quality control and are also presented to the DSMB and NIH as requested. The DCC will also produce safety reports that list adverse events, serious adverse events, deaths, and disease or treatment specific events by site and in aggregate to the DSMB.

With each review the DSMB will approve the study and protocol as is, recommend protocol changes in the interest of patient safety or stop the study based on overwhelming evidence of treatment benefit or safety concerns. The DSMB will provide the recommendation in written minutes provided to the NIH. If the NIH concurs with these recommendations, they will be forwarded to the principal investigator. The recommendations will include any suggested changes in the proposed timing of future DSMB reviews. The review may result in an amendment to the protocol, which must be approved by the IRB, the NIH and the sponsor.

The DSMB report will begin with a brief narrative section that describes the status of the study, progress or findings to-date, issues, and the procedures that produced the report (e.g., data obtained by a specific date). The report will provide a study description along with a current organization chart, current timetable and study schedule as well as a list of study clinical and administrative centers. Data will be presented that describe the administrative status of the study including recruitment and forms handling. Study data reports describe demographic and baseline clinical characteristics and provide a safety assessment. Tables will be provided by site as well as for the whole study population. AE/SAE rates for each group will be presented.

Following each DSMB meeting, the NINDS will send the study’s principal investigator a letter confirming that the DSMB met, reviewed all accumulated study data, and made a recommendation that the study continue as planned (or, possibly, to modify the protocol). The principal investigator will forward a copy of this letter to each of the clinical investigators who, in turn, are responsible for forwarding it to their local IRB.

9.4.3.1 Serious Adverse Event Reporting

We divide potential serious adverse events (SAEs) by the severity: Below we detail the defined SAEs with associated expected event rates and thresholds that would trigger DSMB review. Most of the SAEs would occur during the Epo treatment period or during hospitalization and therefore would be immediately recorded.

Table 12. Serious Adverse Events

Serious Adverse Event (SAE) Potentially Epo related	Expected Rate*	Threshold
Hypertension	20%	25%
Polycythemia (hematocrit > 65%)	Rare < 1%	2%
Major venous or arterial thrombosis (clot) not associated with a central line	Rare < 5%	10%
Other unexpected life threatening event	Rare < 5%	10%

Serious Adverse Event (SAE) Prematurity related	Expected Rate*	Threshold
Pulmonary Hemorrhage (Severe)	7%	15%
NEC (Stage 2b or 3)	12%	25%
Sepsis (severe)	33%	50%
Intracranial hemorrhage (grade III or IV)	16%	25%
Retinopathy of Prematurity (Severe)	7%	14%
**Other Serious Adverse Event: Expected or unexpected**		
Death	Approx. 18%	30%
Cardiac arrest	Rare < 1%	2%
Other life threatening event	Rare < 1%	2%

Adverse Event (AE): In addition to the monitoring of SAEs, we will also measure and compare rates of adverse events across the two treatment arms. Given the high-risk study population a number of adverse events are expected, and the key monitoring function will be to assess whether evidence is accruing that suggests a differential adverse event rate associated with treatment.

Table 13. Adverse Events

Adverse Event (AE)	Expected Rate
Respiratory distress syndrome (RDS)	93%
Patent ductus arteriosus (PDA)	46%
Bronchopulmonary dysplasia (BPD)	42%
Central line-related thrombosis (clot)	40%
Intracranial hemorrhage (ICH)	36%total<br>16% (grade III & IV)
Necrotizing enterocolitis (NEC)	11%
Periventricular leukomalacia (PVL)	4%
Hydrocephalus	2%
Clinical Seizures	6%-13%
ROP (stages 1-2)	59%

Estimates here are based on three primary sources: Stoll et al. (2010); O'Shea et al. (2009) ELGAN study; and The Vermont Oxford Network (VON) 2008 Follow-up Report (2011).[5, 43, 206]

Table 14. Cumulative number of subjects evaluated for safety events and for the long-term efficacy outcome.

Month	6	12	18	24	30	36	42	48	54	60
Safety (3 month)	125	375	625	875	940					
Efficacy (24 month)	0	0	0	0	125	375	625	875	940	

Safety evaluation will be based on all available follow-up but we expect the majority of SAEs and AEs to occur during hospitalization and therefore within the first three months study follow-up. SAE's and AE's will be considered by gestational age category, given that complication rates of infants 24 and 25 weeks of gestation are expected to be higher than those at 26 and 27 weeks of gestation.

Figure 8: Probability (power) of exceeding established thresholds for safety outcomes. Here we consider an expected event rate of 5% with a threshold of 10% used to trigger DSMB action.

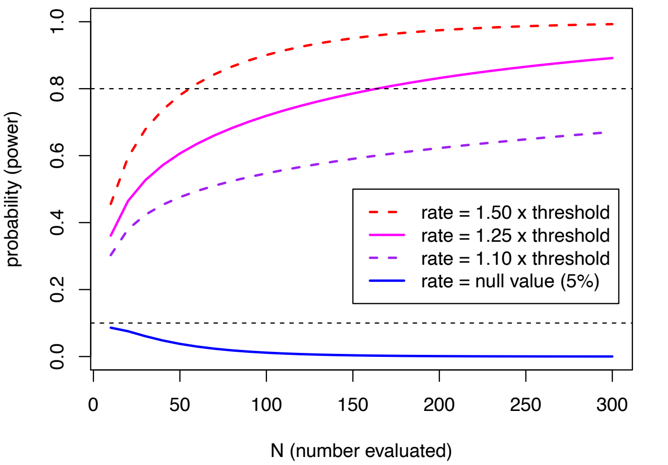

SAEs and Threshold for Action: For each SAE listed in Table 9 we have established expected rates of SAEs for the target study population, and thresholds for each treatment arm at which careful evaluation would be required by DSMB review. The DSMB would be asked to review all information associated with the line listing for the SAEs and then make a decision as to whether the study should be modified, continued or stopped. In Figure 8 we show the operating characteristics for an SAE that has an expected (normal) rate of 5%, and a threshold of 10% for review. If the true SAE rate is 1.5 times the threshold (e.g. 15%) then with 100 or more subjects there is a greater than 80% probability (power) that the observed rate of greater than the threshold would occur and trigger DSMB review. However, if the true SAE rate was only 1.25 times greater than the threshold then 150 or more subjects would be needed to have 80% power. Although a total of 5 SAEs are monitored for their absolute rate, we will not use any multiple comparison correction for continuous monitoring of safety leading to DSMB review. However, formal interim analysis comparing safety across the two study groups will account for both multiple comparisons due to multiple individual safety outcomes, and for multiple interim analyses.

SAE analysis uses a doubling of rates to trigger consideration of DSMB action irrespective of statistical significance at interim analysis, yet formal monitoring will be conducted every 6 months to evaluate the strength of statistical evidence for a difference across groups. Therefore, our proposed interim analyses will evaluate the accruing data using both formal statistical comparison in addition to consideration of absolute rates and is thus more conservative than use of only one criterion. Finally, we plan to conduct interim analyses that will also report on the specific SAEs and AEs listed in Tables 10 and 11, and will provide p-values comparing treatment groups for these safety outcomes in addition to the SAE outcomes. A medical safety officer not involved in the study will review all SAEs, and the DSMB (appointed by NINDS) will decide when they want to review each type of SAE or AE. We recommend that each case of ROP be reviewed as an SAE in real time.

9.5 Formal Interim Analysis

Our primary objective for interim analysis is to allow careful and continuing analysis of safety outcomes. Specifically, we propose to conduct formal statistical analysis and inference for each SAE and AE at three interim and one final analysis time. We will continue to analyze all AE events that have occurred during follow-up, but focus interim analysis on those events that occur within the first three months of follow-up since this is the time period in which the major treatment related events would be expected. We will conduct formal safety evaluation at 6, 12, 18, and 36 months following the start of enrollment. As shown above in Table 11, we expect to have 3-month data available on 125 subjects at the first safety analysis. We plan to monitor death and will control the overall significance level using O'Brien-Fleming boundaries (net alpha=0.05 significance, accounting for three interim and one final analysis).[215] For the other 4 SAEs, we will again use sequential monitoring boundaries to control the outcome-specific family-wise error rate (using a Bonferroni corrected

alpha of 0.05/5 = 0.01).

We have 10 AE outcomes and will display both a standard O'Brien-Fleming sequential monitoring guideline based on alpha=0.05, and a Bonferroni corrected sequential guideline using a significance of 0.05/9 = 0.0056 to correct for multiple outcomes.

The CCC PI will be responsible for notifying the Medical Monitor of any safety events, and the Medical Monitor will notify the DSMB if indicated. All events will be reported to the DSMB in semi-annual reports.

10.0 DATA COLLECTION, SITE MONITORING, AND ADVERSE EVENT REPORTING

10.1 Records to Be Kept

All patient data will be identified by a Study Identification Number (SID). The link between the SID and the patient name/medical record number will be maintained in a locked file available only to the site investigator. This link will be destroyed after study completion, data analysis and publication of results. Retained medical record information will include maternal education, SES, other factors known to affect cognitive development (e.g. drug. alcohol exposure), date of birth, birth weight, medical treatments, complications, length of hospitalization, outcome measures including neurodevelopmental examination results, Bayley III test results, and neuroimages. All retained data will be coded with a SID so no direct link will be available to others besides the site investigator.

Data Storage and Security

Access to research data collected by the PENUT project will be restricted to study team members at each site and UW DCC personnel. The clinical recruitment sites will maintain a secure electronic database (e.g. Access, MySQL, REDCap, etc.) that links the SID generated by the PENUT Portal to study subject contact information. The clinical recruitment sites are responsible for scheduling patient follow-up visits, phone calls, and collection of survey data. The database will be stored on a secure electronic server with user name and passwords log in for individual users and will be backed up nightly.

The REDCap servers are virtual machines (VMs) located on UW DCC hardware in a secure server room. This server room meets the technical requirements for HIPAA compliance and hosts other servers containing PHI.

Storage for all study data is backed by 2 dedicated Network Appliance FAS2050 storage appliances. The filer provides highly fault tolerant storage using large RAID volumes, on-line hot-spare drives, and built-in, proprietary 'snapshot' file system technology that automatically creates hourly, daily, and weekly on-line backups of modified files. Each filer provides approximately 2.6 terabytes (2.6TB) of usable storage space.

The VM Operating System will be kept fully patched and firewalled in accordance with UW Medicine Information Security Policy SEC05.04, which can be found online at http://security.uwmedicine.org/policies.

The UW DCC maintains a Linux-based infrastructure server that uses and manages a central department storage server. A Silicon Mechanics SM-1272A rack-mount server installed in the UW DCC server room rack and an HP dc5750 desktop PC are installed as so-called "logical firewalls", such that that all PCs and operate on a private logical network.

10.2 Role of Data Management

10.2.1 Each clinical site will be responsible for data collection on enrolled subjects and data entry into the REDCap system. Once the study is complete as described in 10.1, each clinical site will be responsible for destruction of links and any retained clinical information.

10.2.2 It will be the responsibility of the Data Coordinating Center (DCC) to maintain confidentiality of all the submitted data by appropriate data encryption methods and password protection entry to database. Please refer to the DCC Manual of Procedures for more detailed information on data handling.

10.3 Quality Assurance

Each clinical site will need local IRB approval of the study protocol prior to enrollment. Each clinical site will be visited by Dr. Sandra Juul and Stephanie Hauge, the UW CCC lead study coordinator or her designee, prior to initiation of subject enrollment. This visit will focus on protocol review, data collection and entry and maintenance of quality control. In addition, the UW CCC lead study coordinator or her designee will visit each clinical site on a regular basis, at least once a year to assure protocol compliance and quality data entry. These visits will include review of all pertinent records, maintenance of study and pharmacy regulatory documents, and review/resolve any data accuracy concerns. Each site will be required to make all study documents and pertinent records available for inspection by the research coordinator or other monitoring authorities.

Data Quality Assurance. We will monitor the accuracy of data entry by the sites both internally and externally. We will review study data on arrival for completeness. We will then subject each submitted data set to a set of preliminary checks to search for values that are out-of-range or otherwise inappropriate.

External monitoring will consist of regular monitoring visits to every site while actively enrolling. Initial monitoring visits will take place prior to subject enrollment, and then every six to 12 months. Using the Patient Monitoring Report (example below), a subset of all data points in the CRFs will be compared with the medical record for 25% of enrolled subjects. Any outstanding data queries will be resolved with the research coordinator at the time of the visit. After each study site visit a report will be prepared and copies sent to the Study File, the study PI (S. Juul), the site PI, and the site coordinator.

PENUT Study ID: UOW-101-01 Report Date: 2015-06-25

V = Verified
NC = Needs correction

Study Enrollment / Baseline Packet

V NC		V NC	
□ □	Site: University of Washington	□ □	Maternal education: Some college, no degree
□ □	Consent date:	□ □	Maternal language: English
□ □	Birth time:	□ □	Birth weight: 698g
□ □	Randomized:	□ □	Head circumference: 21.5cm
□ □	Inc. criteria met. If not, which failed:____________	□ □	Baseline physical exam date:
□ □	Exc. criteria met. If not, which failed:____________	□ □	– Hemangiomas present: No
□ □	Gestational age:	□ □	Baseline head ultrasound:
□ □	Number of babies in gestation: 1	□ □	Gender: Male

PENUT Study Doses

V NC			V NC		
□ □	High Dose 1: 2013-12-26 13:08	23.2h	□ □	Maint. Dose 9: 2014-01-24 09:12	48h
□ □	High Dose 2: 2013-12-28 12:32	47.4h	□ □	Maint. Dose 10: 2014-01-27 12:12	75h
□ □	High Dose 3: 2013-12-30 12:30	48h	□ □	Maint. Dose 11: 2014-01-29 11:59	47.8h
□ □	High Dose 4: 2014-01-01 12:30	48h	□ □	Maint. Dose 12: 2014-01-31 12:35	48.6h
□ □	High Dose 5: 2014-01-03 12:45	48.2h	□ □	Maint. Dose 13: 2014-02-03 12:06	71.5h
□ □	High Dose 6: 2014-01-05 12:30	47.8h	□ □	Maint. Dose 14: 2014-02-05 12:03	48h
□ □	Maint. Dose 1: 2014-01-06 11:45	23.2h	□ □	Maint. Dose 15: 2014-02-07 11:52	47.8h
□ □	Maint. Dose 2: 2014-01-08 11:00	47.2h	□ □	Maint. Dose 16: 2014-02-10 14:05	74.2h
□ □	Maint. Dose 3: 2014-01-10 12:00	49h	□ □	Maint. Dose 17: 2014-02-12 11:25	45.3h
□ □	Maint. Dose 4: 2014-01-13 14:00	74h	□ □	Maint. Dose 18: 2014-02-14 13:44	50.3h
□ □	Maint. Dose 5: 2014-01-15 09:10	43.2h	□ □	Maint. Dose 19: 2014-02-17 11:05	69.3h
□ □	Maint. Dose 6: 2014-01-17 09:06	47.9h	□ □	Maint. Dose 20: 2014-02-19 10:00	46.9h
□ □	Maint. Dose 7: 2014-01-20 11:41	74.6h	□ □	Maint. Dose 21: 2014-02-21 14:10	52.2h
□ □	Maint. Dose 8: 2014-01-22 09:13	45.5h	□ □	Maint. Dose 22: Not given	

High Dose Packet / Tables

V NC		V NC	
□ □	Thrombosis: No / Not noted	□ □	Hemangiomas present: No / Not noted
	Fluid balance – day 3		Blood pressure – day 3
□ □	– Date: 2013-12-28	□ □	– Date: 2013-12-28
□ □	– Weight: 665 g	□ □	– Highest Systolic: 45 mmHg
□ □	– Fluid In: 111.8 mL/day	□ □	– Corresponding Diastolic: 26 mmHg
□ □	– Fluid Out: 93 mL/day	□ □	– Lowest Systolic: 36 mmHg
	Fluid balance – day 10	□ □	– Corresponding Diastolic: 24 mmHg
□ □	– Date: 2014-01-04		Blood pressure – day 10
□ □	– Weight: 780 g	□ □	– Date: 2014-01-04
□ □	– Fluid In: 111.8 mL/day	□ □	– Highest Systolic: 56 mmHg
□ □	– Fluid Out: 77 mL/day	□ □	– Corresponding Diastolic: 32 mmHg
		□ □	– Lowest Systolic: 31 mmHg
		□ □	– Corresponding Diastolic: 25 mmHg

Biological Sample Collection

V NC	Blood Samples	V NC	Urine Samples		V NC		
□ □	Baseline: 2013-12-26 11:57	□ □	Bl: 2013-12-26	10.1h	□ □	#7: 2014-02-17	7.6w
□ □	Day 7: 2014-01-01 13:06	□ □	#1: 2014-01-01	6.4d	□ □	#8: 2014-02-19	7.9w
□ □	Day 9: 2014-01-03 12:00	□ □	#2: 2014-01-08	13.4d	□ □	#9: 2014-03-05	9.9w
□ □	Day 14: 2014-01-08 11:10	□ □	#3: 2014-01-15	20.4d	□ □	#10: 2014-03-19	11.9w
	**CSF Samples**	□ □	#4: 2014-01-22	3.9w	□ □	#11: 2014-04-03	14.1w
□ □	#1: Data missing	□ □	#5: 2014-02-05	5.9w	□ □	#12:	
□ □	#2: Data missing	□ □	#6: 2014-02-06	6.1w			
□ □	#3: Data missing						

Page 1

PENUT Study ID: UOW-101-01 Report Date: 2015-06-25

V = Verified
NC = Needs correction

Discharge Packet / Tables

V NC
☐ ☐ Discharge date: 2014-04-07
☐ ☐ Discharge disposition: Home / foster care
☐ ☐ Dicharge physical exam date: 2014-04-06
☐ ☐ – Weight: 3504g
☐ ☐ – Head circumference: 34.5cm
☐ ☐ – Hemangiomas present: No

Hypertension

☐ ☐ – Treatment < 2w of life: No
☐ ☐ – Treatment > 2w of life: No

Hypotension

☐ ☐ – Treatment < 2w of life: Yes
☐ ☐ – Treatment > 2w of life: No
☐ ☐ Opthalmologic exam: No
☐ ☐ 36-week HUS or MRI: 2014-03-17
☐ ☐ Transferred to another hospital: No
☐ ☐ Discharge Questionnaire: 2014-04-07
☐ ☐ 4-Month Interview: 2014-08-11
☐ ☐ 8-Month Interview: 2014-12-08
☐ ☐ 12-Month Interview: 2015-03-16

V NC
☐ ☐ **Transfusions**

Transfusion Data

Date	Product	Volume
2013-12-26	Packed red blood cells	22
2013-12-31	Packed red blood cells	10
2014-01-06	Packed red blood cells	12
2014-01-11	Packed red blood cells	13

☐ ☐ **Infections**

Infections Data

Date	Organism	Name	Specimen
2014-01-30	bacterial	E. coli	U

☐ ☐ **Blood Pressure Medications**

BP Meds Data

Start Date	Stop Date	Medication
2013-12-25	2013-12-26	Dopamine
2014-01-01	2014-01-08	Dopamine

☐ ☐ **Steroids**

Steroids Data

Start Date	Stop Date	Name	Dose
2014-01-05	2014-01-05	Hydrocortisone	0.4
2014-01-06	2014-01-06	Hydrocortisone	1.23
2014-01-07	2014-01-08	Hydrocortisone	3.32
2014-01-09	2014-01-09	Hydrocortisone	2.26
2014-01-10	2014-01-10	Hydrocortisone	1.2
2014-01-11	2014-01-11	Hydrocortisone	1.4
2014-01-12	2014-01-15	Hydrocortisone	1.2
2014-01-16	2014-01-16	Dexamethasone	0.18
2014-01-16	2014-01-16	Hydrocortisone	0.8
2014-01-17	2014-01-17	Dexamethasone	0.14
2014-01-18	2014-01-18	Hydrocortisone	0.8
2014-01-18	2014-01-18	Dexamethasone	0.10
2014-01-19	2014-01-22	Hydrocortisone	1.2
2014-01-23	2014-01-23	Hydrocortisone	1.0
2014-01-24	2014-01-27	Hydrocortisone	1.2
2014-01-28	2014-02-01	Hydrocortisone	0.9
2014-02-02	2014-02-05	Hydrocortisone	0.6
2014-02-06	2014-02-08	Hydrocortisone	0.3

SAEs, Violations, Status

■ = SAE recorded in database

V NC
☐ ☐ Hypertension
☐ ☐ Polycythemia
☐ ☐ Major venous/arterial thrombosis
☐ ☐ Pulmonary hemorrhage
☐ ☐ Intracranial hemorrhage (grade 3 or 4)
☐ ☐ NEC (stage 2b or 3)
☐ ☐ Sepsis (Severe)
☐ ☐ Retinopathy or prematurity (severe)
☐ ☐ Death
☐ ☐ Cardiac Arrest
☐ ☐ Other unexpected life threatening event

Participant Status

☐ ☐ Discontinued study drug dosing: No
☐ ☐ Discontinued ALL study activities: No
☐ ☐ Participant died: No

Protocol Deviations/Violations

☐ ☐ None listed

Page 2

PENUT Study ID: UOW-101-01

Report Date: 2015-06-25

V = Verified
NC = Needs correction

☐ ☐ Iron

Iron Data

Date From	Date To	Route	Medication	Dose
2014-01-02	2014-01-02	Iron IV	Iron Sucrose	1
2014-01-09	2014-01-09	Iron IV	Iron Sucrose	1
2014-01-13	2014-01-13	Iron IV	Iron Sucrose	1
2014-01-14	2014-01-14	Iron PO	Ferrin Sol (ferrous sulfate)	1.35
2014-01-15	2014-01-15	Iron PO	Ferrin Sol (ferrous sulfate)	4.05
2014-01-16	2014-01-27	Iron PO	Ferrin Sol (ferrous sulfate)	5.4
2014-01-28	2014-01-28	Iron PO	Ferrin Sol (ferrous sulfate)	5.8
2014-01-29	2014-01-29	Iron PO	Ferrin Sol (ferrous sulfate)	6.2
2014-01-30	2014-01-30	Iron PO	Ferrin Sol (ferrous sulfate)	3.1
2014-02-02	2014-02-02	Iron PO	Ferrin Sol (ferrous sulfate)	6.2
2014-02-03	2014-02-03	Iron PO	Ferrin Sol (ferrous sulfate)	3.1
2014-02-04	2014-02-09	Iron PO	Ferrin Sol (ferrous sulfate)	6.2
2014-02-10	2014-02-10	Iron PO	Ferrin Sol (ferrous sulfate)	8.9
2014-02-11	2014-02-11	Iron PO	Ferrin Sol (ferrous sulfate)	17.4
2014-02-12	2014-02-18	Iron PO	Ferrin Sol (ferrous sulfate)	11.6
2014-02-19	2014-02-23	Iron PO	Ferrin Sol (ferrous sulfate)	14.4
2014-02-24	2014-02-24	Iron PO	Ferrin Sol (ferrous sulfate)	13.4
2014-02-25	2014-03-02	Iron PO	Ferrin Sol (ferrous sulfate)	18.6
2014-03-03	2014-03-03	Iron PO	Ferrin Sol (ferrous sulfate)	12.4
2014-03-04	2014-03-04	Iron PO	Ferrin Sol (ferrous sulfate)	18.6
2014-03-05	2014-03-05	Iron PO	Ferrin Sol (ferrous sulfate)	19.4
2014-03-06	2014-03-11	Iron PO	Ferrin Sol (ferrous sulfate)	21
2014-03-12	2014-03-12	Iron PO	Ferrin Sol (ferrous sulfate)	14.9
2014-03-13	2014-03-17	Iron PO	Ferrin Sol (ferrous sulfate)	23.7
2014-03-18	2014-03-18	Iron PO	Ferrin Sol (ferrous sulfate)	25.5
2014-03-19	2014-03-19	Iron PO	Ferrin Sol (ferrous sulfate)	17.6
2014-03-20	2014-04-07	Iron PO	Poly-Vi-Sol with Iron	10
2014-03-20	2014-03-24	Iron PO	Ferrin Sol (ferrous sulfate)	11
2014-03-25	2014-03-25	Iron PO	Ferrin Sol (ferrous sulfate)	19.7
2014-03-26	2014-03-30	Iron PO	Ferrin Sol (ferrous sulfate)	17.4
2014-03-31	2014-03-31	Iron PO	Ferrin Sol (ferrous sulfate)	8.7
2014-04-01	2014-04-01	Iron PO	Ferrin Sol (ferrous sulfate)	37.5
2014-04-02	2014-04-06	Iron PO	Ferrin Sol (ferrous sulfate)	25
2014-04-07	2014-04-07	Iron PO	Ferrin Sol (ferrous sulfate)	12.5

☐ ☐ Labs

Labs Data

Date	ZnPPH	Creatinine	Ferritin	HCT Low	HCT High
2013-12-25				31	31
2013-12-26		0.88		25	29
2013-12-27		0.99			
2013-12-28		0.98			
2013-12-29		1.23		34	34
2013-12-30		1.09		31	31
2013-12-31		1.36		35	35
2014-01-01		1.16		33	33
2014-01-02		1.17			
2014-01-03		1.06		38	38
2014-01-04		1.11		37	37
2014-01-05		0.95			
2014-01-06		1		32	32
2014-01-07		0.96			
2014-01-08		0.89			
2014-01-10		0.75		33	33
2014-01-11		0.64			
2014-01-13	>250	0.82		38	38
2014-01-15		0.79			
2014-01-18		0.92			
2014-01-22		0.59			
2014-01-26	>250	0.64		38	38
2014-01-27					
2014-01-28					
2014-01-30				37	37
2014-01-31		0.44			
2014-02-01		0.51			
2014-02-07	>250			36	36
2014-02-21	>250			37	37
2014-02-24			33		
2014-03-03	>250			36	36
2014-03-24	>250			31	31
2014-04-07	217			30	30

Comments

Site Monitor Signature, Date

10.4 Serious Adverse Event Reporting

Since reporting rules vary by institution, by IRB, and by government agency (NIH), the following statements are a conservative guide to reporting serious adverse events for this trial and may be further refined with DSMB guidance.

When an SAE occurs, the PI of the CCC (S. Juul) will be notified by the site within 24-72 hours per protocol (see Figure 9). She, or her designee, is responsible for reporting the SAE to the Medical Monitor (Jack Widness), the DSMB and NINDS (via Peter Gilbert). The immediate reports will be followed promptly by detailed, written reports within 3-7 days per protocol. SAE reporting will also follow the requirements of the local IRB and the FDA. SAEs and/or laboratory abnormalities identified in the protocol as critical to participant safety must be reported. All SAEs experienced by participants during the study time frame specified in the protocol (from the time of study drug administration to discharge) are to be reported. A death, found at any time point, including follow-up, should be reported. The DCC will provide monthly summaries of all SAEs to the CCC PI, DCC PI, Medical Monitor, and Peter Gilbert (NINDS). The Medical Monitor is responsible for notifying the FDA when indicated.
Figure 9 shows the flow diagram of how adverse events will be reported.

Figure 9. SAE Flow Diagram

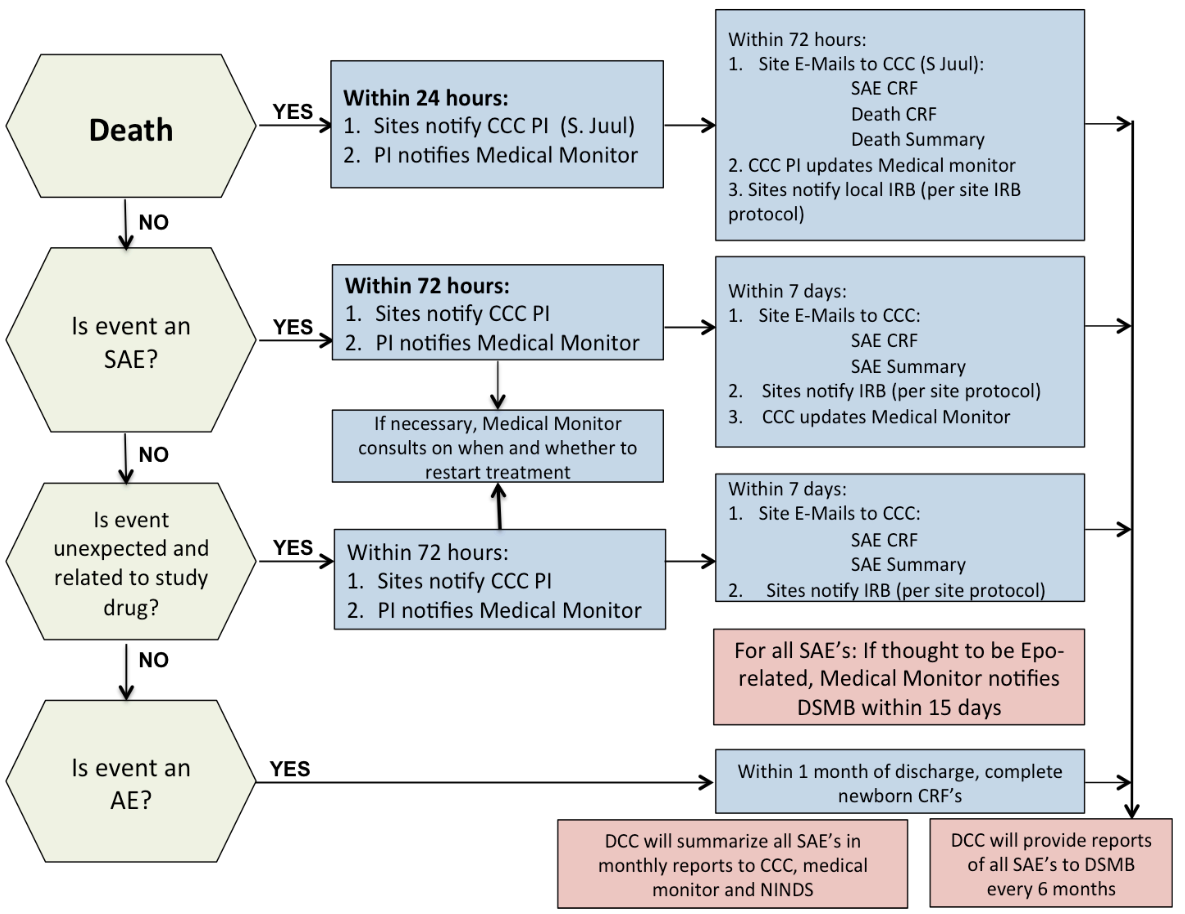

Unexpected adverse events which are serious, but not life threatening, and have a causal relation to the research, (unexpected in this context means not mentioned in the informed consent) must be reported to the PI of the CCC (S. Juul) within 72 hours per protocol (see Figure 9). She, or her designee, is responsible for notifying the Medical Monitor, DSMB and NINDS within 7 days. The local IRB should be notified as per local regulations by the Site.
The DSMB may call an emergency meeting, if necessary.

11.0 HUMAN SUBJECTS

11.1 Institutional Review Board (IRB) Review and Informed Consent

This protocol and the informed consnt document and any subsequent modifications will be reviewed and approved by the IRB or ethics committee responsible for oversight of the study. A signed consent form will be obtained from the subject's parent, legal guardian, or person with power of attorney. The consent form will describe the purpose of the study, the procedures to be followed, and the risks and benefits of participation. A copy of the consent form will be given to the subject, parent, or legal guardian, and this fact will be documented in the subject's record. A model informed consent form is included in Appendix 1.

11.2 Subject Confidentiality

The UW DCC will support an https secured web page (https://www.penut-trial.org) that provides a centralized location for information about the Preterm Epo Neuroprotection (PENUT) Trial for patients, providers, investigators, and institutional agencies. The web page will also contain a link to the PENUT Portal https://www.penut-trial.org/portal where all research personnel will log in with individual user names and passwords to securely perform study data management activities.

The PENUT Portal controls the assignment of sequentially generated Study Identification Numbers (SID) for all patients screened under the PENUT project. All patient contact information will be linked to the SID within databases hosted at the clinical recruitment sites and in a REDCap database at the DCC that stored separately from all other study or clinical data.

All laboratory specimens, evaluation forms, reports, video recordings, and other records that leave the site will be identified only by the SID to maintain subject confidentiality. All records will be kept in a locked file cabinet at each site. All computer entry and networking programs will be done using SIDs only. Clinical information will not be released without written permission of the subject, except as necessary for monitoring by IRB, the FDA, the NINDS, the OHRP, the sponsor, or the sponsor's designee.

Certain aspects of the subject's medical history and demographics will be collected for this study. Loss of privacy may lead to problems with insurability or social stigmatization. However, all data will be collected by study personnel with due attention to patient privacy. A Certificate of Confidentiality from the United States Department of Health and Human Services (DHHS) will be obtained by the CCC PI for all sites, to protect the subjects' confidential information. With this Certificate, study investigators cannot be forced (for example by court order or subpoena) to disclose information that may identify subject, subject's parent, legal guardian, or person with power of attorney any federal, state, local, civil, legislative, administrative, or other proceedings. The researchers will use the Certificate to resist any demands for information that would identify any specific individual, except to prevent serious harm to anyone.

11.3 Study Modification/Discontinuation

The study may be modified or discontinued at any time by the DSMB, IRB, the NINDS, the sponsor, the OHRP, the FDA, or other government agencies as part of their duties to ensure that research subjects are protected.

12.0 PUBLICATION OF RESEARCH FINDINGS

A Publications and Presentations Policy document has been developed to describe processes for defining study publications and presentations, for assigning authors in accord with JAMA criteria, and for reviewing publications prior to submission. (Please see Appendix 4). A proposal for a manuscript will be initiated by

submitting a structured summary proposal that includes an analysis plan. All proposals will be reviewed by the Publications and Presentations and Executive Committees. Similarly, all abstracts, presentations, and publications must also be approved by the Publications and Presentations and Executive Committees, and it will be the responsibility of the DCC to ensure that the process is transparent and timely. An Excel spreadsheet or the study website will include a searchable list of all analysis proposals and will track their status toward publication.

We expect that Epo treatment will improve NDI-free survival compared to that seen in the ELGAN and NICHD trials. We expect that this benefit will translate into shorter and less complicated hospital stays and better neurodevelopmental outcomes. This outcome of the trial would represent an important advance in the care of ELGANs that could change the standard of care for high-risk infants. If Epo treatment has no demonstrable benefit, or if adverse effects are observed, this information will also be useful in the field because Epo is currently used anecdotally in many NICUs, without trial-based evidence, for some severely ill infants. The results of the PENUT trial will be of particular interest to pediatricians trained in neonatal-perinatal medicine. This group of practitioners is largely centered in academic or medical centers and they attend several meetings each year where new information about treatment can be presented. The PENUT trial results will be presented at regional Pediatric Research Society meetings such as the Western and Eastern Society for Pediatric Research, and at national and international meetings including the Pediatric Academic Societies combined meetings, the European Society for Pediatric Research meeting, the American Academy of Pediatrics meetings, and at other venues such as “Hot Topics in Neonatology”.

13.0 REFERENCES

BIBLIOGRAPHY

1. Moore T, Johnson S, Haider S, Hennessy E, Marlow N. The Bayley-III cognitive and language scales: how do scores relate to the Bayley II? Arch Dis Child. 2011;96(Suppl 1):A1–A100. doi: 10.1136/adc.2011.212563.85.
2. Rouse DJ, Hirtz DG, Thom E, Varner MW, Spong CY, Mercer BM, et al. A randomized, controlled trial of magnesium sulfate for the prevention of cerebral palsy. N Engl J Med. 2008;359(9):895-905. Epub 2008/08/30. doi: 10.1056/NEJMoa0801187. PubMed PMID: 18753646; PubMed Central PMCID: PMC2803083.
3. Palisano R, Rosenbaum P, Walter S, Russell D, Wood E, Galuppi B. Development and reliability of a system to classify gross motor function in children with cerebral palsy. Dev Med Child Neurol. 1997;39(4):214-23. Epub 1997/04/01. PubMed PMID: 9183258.
4. Palisano RJ, Rosenbaum P, Bartlett D, Livingston MH. Content validity of the expanded and revised Gross Motor Function Classification System. Dev Med Child Neurol. 2008;50(10):744-50. Epub 2008/10/07. doi: 10.1111/j.1469-8749.2008.03089.x. PubMed PMID: 18834387.
5. Stoll BJ, Hansen NI, Bell EF, Shankaran S, Laptook AR, Walsh MC, et al. Neonatal outcomes of extremely preterm infants from the NICHD Neonatal Research Network. Pediatrics. 2010;126(3):443-56. Epub 2010/08/25. doi: 10.1542/peds.2009-2959. PubMed PMID: 20732945; PubMed Central PMCID: PMC2982806.
6. La Pine TR, Jackson JC, Bennett FC. Outcome of infants weighing less than 800 grams at birth: 15 years' experience. Pediatrics. 1995;96(3 Pt 1):479-83. PubMed PMID: 7544456.
7. Vohr BR, Wright LL, Dusick AM, Perritt R, Poole WK, Tyson JE, et al. Center differences and outcomes of extremely low birth weight infants. Pediatrics. 2004;113(4):781-9. PubMed PMID: 15060228.
8. Hack M, Fanaroff AA. Outcomes of children of extremely low birthweight and gestational age in the 1990s. Semin Neonatol. 2000;5(2):89-106. PubMed PMID: 10859704.
9. Wilson-Costello D, Friedman H, Minich N, Fanaroff AA, Hack M. Improved survival rates with increased neurodevelopmental disability for extremely low birth weight infants in the 1990s. Pediatrics. 2005;115(4):997-1003. PubMed PMID: 15805376.
10. Tyson JE, Parikh NA, Langer J, Green C, Higgins RD. Intensive care for extreme prematurity--moving beyond gestational age. N Engl J Med. 2008;358(16):1672-81. PubMed PMID: 18420500.
11. Eichenwald EC, Stark AR. Management and outcomes of very low birth weight. N Engl J Med. 2008;358(16):1700-11. PubMed PMID: 18420502.
12. Hoekstra RE, Ferrara TB, Couser RJ, Payne NR, Connett JE. Survival and long-term neurodevelopmental outcome of extremely premature infants born at 23-26 weeks' gestational age at a tertiary center. Pediatrics. 2004;113(1 Pt 1):e1-6. Epub 2004/01/02. PubMed PMID: 14702487.
13. Hintz SR, Kendrick DE, Stoll BJ, Vohr BR, Fanaroff AA, Donovan EF, et al. Neurodevelopmental and growth outcomes of extremely low birth weight infants after necrotizing enterocolitis. Pediatrics. 2005;115(3):696-703. Epub 2005/03/03. doi: 10.1542/peds.2004-0569. PubMed PMID: 15741374.
14. Wood NS, Marlow N, Costeloe K, Gibson AT, Wilkinson AR. Neurologic and developmental disability after extremely preterm birth. EPICure Study Group. N Engl J Med. 2000;343(6):378-84. PubMed PMID: 10933736.
15. Wood NS, Costeloe K, Gibson AT, Hennessy EM, Marlow N, Wilkinson AR. The EPICure study: growth and associated problems in children born at 25 weeks of gestational age or less. Arch Dis Child Fetal Neonatal Ed. 2003;88(6):F492-500. PubMed PMID: 14602697.
16. Wood NS, Costeloe K, Gibson AT, Hennessy EM, Marlow N, Wilkinson AR. The EPICure study: associations and antecedents of neurological and developmental disability at 30 months of age following extremely preterm birth. Arch Dis Child Fetal Neonatal Ed. 2005;90(2):F134-40. PubMed PMID: 15724037.
17. Beauchamp MH, Thompson DK, Howard K, Doyle LW, Egan GF, Inder TE, et al. Preterm infant hippocampal volumes correlate with later working memory deficits. Brain. 2008;131(Pt 11):2986-94. PubMed PMID: 18799516.
18. Edgin JO, Inder TE, Anderson PJ, Hood KM, Clark CA, Woodward LJ. Executive functioning in preschool children

born very preterm: relationship with early white matter pathology. J Int Neuropsychol Soc. 2008;14(1):90-101. PubMed PMID: 18078535.

19. Gargus RA, Vohr BR, Tyson JE, High P, Higgins RD, Wrage LA, et al. Unimpaired outcomes for extremely low birth weight infants at 18 to 22 months. Pediatrics. 2009;124(1):112-21. PubMed Central PMCID: PMC2856069.
20. Marlow N, Hennessy EM, Bracewell MA, Wolke D. Motor and executive function at 6 years of age after extremely preterm birth. Pediatrics. 2007;120(4):793-804. PubMed PMID: 17908767.
21. Johnson S, Hollis C, Kochhar P, Hennessy E, Wolke D, Marlow N. Autism spectrum disorders in extremely preterm children. J Pediatr. 2010;156(4):525-31. PubMed PMID: 20056232.
22. Buchmayer S, Johansson S, Johansson A, Hultman CM, Sparen P, Cnattingius S. Can association between preterm birth and autism be explained by maternal or neonatal morbidity? Pediatrics. 2009;124(5):e817-25. PubMed PMID: 19841112.
23. Kuban KC, O'Shea TM, Allred EN, Tager-Flusberg H, Goldstein DJ, Leviton A. Positive screening on the Modified Checklist for Autism in Toddlers (M-CHAT) in extremely low gestational age newborns. J Pediatr. 2009;154(4):535-40 e1. doi: 10.1016/j.jpeds.2008.10.011. PubMed PMID: 19185317; PubMed Central PMCID: PMCPMC2693887.
24. Scott MN, Taylor HG, Fristad MA, Klein N, Espy KA, Minich N, et al. Behavior Disorders in Extremely Preterm/Extremely Low Birth Weight Children in Kindergarten. J Dev Behav Pediatr. 2012. Epub 2012/01/17. doi: 10.1097/DBP.0b013e3182475287. PubMed PMID: 22245934.
25. Petrou S, Henderson J, Bracewell M, Hockley C, Wolke D, Marlow N. Pushing the boundaries of viability: the economic impact of extreme preterm birth. Early Hum Dev. 2006;82(2):77-84. PubMed PMID: 16466865.
26. Rushing S, Ment LR. Preterm birth: a cost benefit analysis. Seminars in Perinatology. 2004;28(6):444-50. Epub 2005/02/08. PubMed PMID: 15693401.
27. van der Kooij MA, Groenendaal F, Kavelaars A, Heijnen CJ, van Bel F. Neuroprotective properties and mechanisms of erythropoietin in in vitro and in vivo experimental models for hypoxia/ischemia. Brain Res Rev. 2008;59(1):22-33. PubMed PMID: 18514916.
28. Gonzalez FF, Abel R, Almli CR, Mu D, Wendland M, Ferriero DM. Erythropoietin sustains cognitive function and brain volume after neonatal stroke. Dev Neurosci. 2009;31(5):403-11. PubMed PMID: 19672069.
29. Kellert BA, McPherson RJ, Juul SE. A comparison of high-dose recombinant erythropoietin treatment regimens in brain-injured neonatal rats. Pediatr Res. 2007;61(4):451-5. doi: 10.1203/pdr.0b013e3180332cec. PubMed PMID: 17515870.
30. Rees S, Hale N, De Matteo R, Cardamone L, Tolcos M, Loeliger M, et al. Erythropoietin is neuroprotective in a preterm ovine model of endotoxin-induced brain injury. J Neuropathol Exp Neurol. 2010;69(3):306-19. Epub 2010/02/10. doi: 10.1097/NEN.0b013e3181d27138. PubMed PMID: 20142760.
31. Loeliger MM, Mackintosh A, De Matteo R, Harding R, Rees SM. Erythropoietin protects the developing retina in an ovine model of endotoxin-induced retinal injury. Invest Ophthalmol Vis Sci. 2011;52(5):2656-61. Epub 2011/01/20. doi: 10.1167/iovs.10-6455. PubMed PMID: 21245409.
32. Juul SE, McPherson RJ, Farrell FX, Jolliffe L, Ness DJ, Gleason CA. Erytropoietin concentrations in cerebrospinal fluid of nonhuman primates and fetal sheep following high-dose recombinant erythropoietin. Biol Neonate. 2004;85(2):138-44. PubMed PMID: 14639039.
33. Fauchere JC, Dame C, Vonthein R, Koller B, Arri S, Wolf M, et al. An approach to using recombinant erythropoietin for neuroprotection in very preterm infants. Pediatrics. 2008;122(2):375-82. PubMed PMID: 18676556.
34. Weber A, Dzietko M, Berns M, Felderhoff-Mueser U, Heinemann U, Maier RF, et al. Neuronal damage after moderate hypoxia and erythropoietin. Neurobiol Dis. 2005;20(2):594-600. PubMed PMID: 15935685.
35. Juul SE, McPherson RJ, Bauer LA, Ledbetter KJ, Gleason CA, Mayock DE. A phase I/II trial of high-dose erythropoietin in extremely low birth weight infants: pharmacokinetics and safety. Pediatrics. 2008;122(2):383-91. PubMed PMID: 18676557.
36. Widness JA, Veng-Pedersen P, Peters C, Pereira LM, Schmidt RL, Lowe LS. Erythropoietin pharmacokinetics in premature infants: developmental, nonlinearity, and treatment effects. J Appl Physiol. 1996;80(1):140-8.
37. Statler PA, McPherson RJ, Bauer LA, Kellert BA, Juul SE. Pharmacokinetics of high-dose recombinant erythropoietin in plasma and brain of neonatal rats. Pediatr Res. 2007;61(6):671-5. doi:

10.1203/pdr.0b013e31805341dc. PubMed PMID: 17426655.

38. Mwansa-Kambafwile J, Cousens S, Hansen T, Lawn JE. Antenatal steroids in preterm labour for the prevention of neonatal deaths due to complications of preterm birth. Int J Epidemiol. 2010;39 Suppl 1:i122-33. Epub 2010/04/02. doi: 10.1093/ije/dyq029. PubMed PMID: 20348115; PubMed Central PMCID: PMC2845868.

39. Gluckman PD, Wyatt JS, Azzopardi D, Ballard R, Edwards AD, Ferriero DM, et al. Selective head cooling with mild systemic hypothermia after neonatal encephalopathy: multicentre randomised trial. Lancet. 2005;365(9460):663-70. PubMed PMID: 15721471.

40. Shankaran S, Laptook AR, Ehrenkranz RA, Tyson JE, McDonald SA, Donovan EF, et al. Whole-body hypothermia for neonates with hypoxic-ischemic encephalopathy. N Engl J Med. 2005;353(15):1574-84. PubMed PMID: 16221780.

41. Azzopardi DV, Strohm B, Edwards AD, Dyet L, Halliday HL, Juszczak E, et al. Moderate hypothermia to treat perinatal asphyxial encephalopathy. N Engl J Med. 2009;361(14):1349-58. PubMed PMID: 19797281.

42. Field DJ, Dorling JS, Manktelow BN, Draper ES. Survival of extremely premature babies in a geographically defined population: prospective cohort study of 1994-9 compared with 2000-5. Bmj. 2008;336(7655):1221-3. Epub 2008/05/13. doi: 10.1136/bmj.39555.670718.BE. PubMed PMID: 18469017; PubMed Central PMCID: PMC2405852.

43. O'Shea TM, Allred EN, Dammann O, Hirtz D, Kuban KC, Paneth N, et al. The ELGAN study of the brain and related disorders in extremely low gestational age newborns. Early Hum Dev. 2009;85(11):719-25. Epub 2009/09/22. doi: 10.1016/j.earlhumdev.2009.08.060. PubMed PMID: 19765918; PubMed Central PMCID: PMC2801579.

44. Rajagopalan V, Scott JA, Habas PA, Corbett-Detig JM, Kim K, Rousseau F, et al. Local tissue growth patterns underlying normal fetal human brain gyrification quantified in utero. J Neurosci. 2011;31(8):2878-87.

45. Lodygensky GA, Vasung L, Sizonenko SV, Huppi PS. Neuroimaging of cortical development and brain connectivity in human newborns and animal models. J Anat. 2010;217(4):418-28. Epub 2010/10/29. doi: 10.1111/j.1469-7580.2010.01280.x. PubMed PMID: 20979587.

46. Huppi PS. Growth and development of the brain and impact on cognitive outcomes. Nestle Nutr Workshop Ser Pediatr Program. 2010;65:137-49; discussion 49-51. PubMed PMID: 20139679.

47. Back SA, Luo NL, Borenstein NS, Levine JM, Volpe JJ, Kinney HC. Late oligodendrocyte progenitors coincide with the developmental window of vulnerability for human perinatal white matter injury. J Neurosci. 2001;21(4):1302-12. PubMed PMID: 11160401.

48. Back SA, Rivkees SA. Emerging concepts in periventricular white matter injury. Semin Perinatol. 2004;28(6):405-14. PubMed PMID: 15693397.

49. Volpe JJ. Brain injury in the premature infant: overview of clinical aspects, neuropathology, and pathogenesis. Semin Pediatr Neurol. 1998;5(3):135-51. PubMed PMID: 9777673.

50. Volpe JJ. Neurobiology of periventricular leukomalacia in the premature infant. Pediatr Res. 2001;50(5):553-62. PubMed PMID: 11641446.

51. Volpe JJ. Perinatal brain injury: From pathogenesis to neuroprotection. Ment Retard Dev Disabil Res Rev. 2001;7(1):56-64. PubMed PMID: 11241883.

52. Volpe JJ. Brain injury in premature infants: a complex amalgam of destructive and developmental disturbances. Lancet Neurol. 2009;8(1):110-24. Epub 2008/12/17. PubMed PMID: 19081519; PubMed Central PMCID: PMC2707149.

53. Volpe JJ. The encephalopathy of prematurity--brain injury and impaired brain development inextricably intertwined. Semin Pediatr Neurol. 2009;16(4):167-78. Epub 2009/12/01. PubMed PMID: 19945651; PubMed Central PMCID: PMC2799246.

54. Back SA, Han BH, Luo NL, Chricton CA, Xanthoudakis S, Tam J, et al. Selective vulnerability of late oligodendrocyte progenitors to hypoxia-ischemia. J Neurosci. 2002;22(2):455-63. PubMed PMID: 11784790.

55. Back SA, Luo NL, Mallinson RA, O'Malley JP, Wallen LD, Frei B, et al. Selective vulnerability of preterm white matter to oxidative damage defined by F2-isoprostanes. Ann Neurol. 2005;58(1):108-20. PubMed PMID: 15984031.

56. Back SA, Riddle A, McClure MM. Maturation-dependent vulnerability of perinatal white matter in premature birth. Stroke. 2007;38(2 Suppl):724-30. PubMed PMID: 17261726.

57. Volpe JJ. Neurology of the Newborn. 4th ed: W.B. Saunders; 2001. 912 p.
58. Zacharias R, Schmidt M, Kny J, Sifringer M, Bercker S, Bittigau P, et al. Dose-dependent effects of erythropoietin in propofol anesthetized neonatal rats. Brain Res. 2010;1343:14-9. PubMed PMID: 20452333.
59. Kumral A, Gonenc S, Acikgoz O, Sonmez A, Genc K, Yilmaz O, et al. Erythropoietin increases glutathione peroxidase enzyme activity and decreases lipid peroxidation levels in hypoxic-ischemic brain injury in neonatal rats. Biol Neonate. 2005;87(1):15-8. PubMed PMID: 15334031.
60. Gonzalez FF, McQuillen P, Mu D, Chang Y, Wendland M, Vexler Z, et al. Erythropoietin enhances long-term neuroprotection and neurogenesis in neonatal stroke. Dev Neurosci. 2007;29:321-30.
61. Demers EJ, McPherson RJ, Juul SE. Erythropoietin protects dopaminergic neurons and improves neurobehavioral outcomes in juvenile rats after neonatal hypoxia-ischemia. Pediatr Res. 2005;58(2):297-301. PubMed PMID: 16055937.
62. McPherson RJ, Demers EJ, Juul SE. Safety of high-dose recombinant erythropoietin in a neonatal rat model. Neonatology. 2007;91(1):36-43. PubMed PMID: 17344650.
63. Iwai M, Stetler RA, Xing J, Hu X, Gao Y, Zhang W, et al. Enhanced oligodendrogenesis and recovery of neurological function by erythropoietin after neonatal hypoxic/ischemic brain injury. Stroke. 2010;41(5):1032-7. Epub 2010/04/03. doi: 10.1161/STROKEAHA.109.570325. PubMed PMID: 20360553; PubMed Central PMCID: PMC2919308.
64. Reitmeir R, Kilic E, Kilic U, Bacigaluppi M, ElAli A, Salani G, et al. Post-acute delivery of erythropoietin induces stroke recovery by promoting perilesional tissue remodelling and contralesional pyramidal tract plasticity. Brain. 2011;134(Pt 1):84-99. Epub 2010/12/28. doi: 10.1093/brain/awq344. PubMed PMID: 21186263.
65. Leviton A, Fichorova R, Yamamoto Y, Allred EN, Dammann O, Hecht J, et al. Inflammation-related proteins in the blood of extremely low gestational age newborns. The contribution of inflammation to the appearance of developmental regulation. Cytokine. 2011;53(1):66-73. doi: 10.1016/j.cyto.2010.09.003. PubMed PMID: 20934883; PubMed Central PMCID: PMCPMC2987520.
66. Martin CR, Dammann O, Allred EN, Patel S, O'Shea TM, Kuban KC, et al. Neurodevelopment of extremely preterm infants who had necrotizing enterocolitis with or without late bacteremia. J Pediatr. 2010;157(5):751-6 e1. Epub 2010/07/06. doi: 10.1016/j.jpeds.2010.05.042. PubMed PMID: 20598317; PubMed Central PMCID: PMC2952050.
67. Ivacko JA, Sun R, Silverstein FS. Hypoxic-ischemic brain injury induces an acute microglial reaction in perinatal rats. Pediatr Res. 1996;39(1):39-47. PubMed PMID: 8825384.
68. Foster-Barber A, Dickens B, Ferriero DM. Human perinatal asphyxia: correlation of neonatal cytokines with MRI and outcome. Dev Neurosci. 2001;23(3):213-8. PubMed PMID: 11598323.
69. Bartha AI, Foster-Barber A, Miller SP, Vigneron DB, Glidden DV, Barkovich AJ, et al. Neonatal encephalopathy: association of cytokines with MR spectroscopy and outcome. Pediatr Res. 2004;56(6):960-6. PubMed PMID: 15496611.
70. Juul S, Felderhoff-Mueser U. Epo and other hematopoietic factors. Semin Fetal Neonatal Med. 2007;12(4):250-8. Epub 2007/02/27. PubMed PMID: 17321813; PubMed Central PMCID: PMC2018740.
71. Gorio A, Gokmen N, Erbayraktar S, Yilmaz O, Madaschi L, Cichetti C, et al. Recombinant human erythropoietin counteracts secondary injury and markedly enhances neurological recovery from experimental spinal cord trauma. PNAS. 2002;99(14):9450-5. Epub 2002/06/26. doi: 10.1073/pnas.142287899. PubMed PMID: 12082184; PubMed Central PMCID: PMC123161.
72. Villa P, Bigini P, Mennini T, Agnello D, Laragione T, Cagnotto A, et al. Erythropoietin selectively attenuates cytokine production and inflammation in cerebral ischemia by targeting neuronal apoptosis. J Exp Med. 2003;198(6):971-5. Epub 2003/09/17. PubMed Central PMCID: PMC2194205.
73. Sun Y, Calvert JW, Zhang JH. Neonatal hypoxia/ischemia is associated with decreased inflammatory mediators after erythropoietin administration. Stroke. 2005;36(8):1672-8. PubMed PMID: 16040592.
74. Siren AL, Fratelli M, Brines M, Goemans C, Casagrande S, Lewczuk P, et al. Erythropoietin prevents neuronal apoptosis after cerebral ischemia and metabolic stress. PNAS. 2001;98(7):4044-9. PubMed PMID: 11259643.
75. Bian XX, Yuan XS, Qi CP. Effect of recombinant human erythropoietin on serum S100B protein and interleukin-6 levels after traumatic brain injury in the rat. Neurol Med Chir (Tokyo). 2010;50(5):361-6. Epub 2010/05/28.

PubMed PMID: 20505289.
76. Juul SE, Beyer RP, Bammler TK, McPherson RJ, Wilkerson J, Farin FM. Microarray analysis of high-dose recombinant erythropoietin treatment of unilateral brain injury in neonatal mouse hippocampus. Pediatr Res. 2009;65(5):485-92. Epub 2009/02/05. doi: 10.1203/PDR.0b013e31819d90c8. PubMed PMID: 19190543.
77. Yatsiv I, Grigoriadis N, Simeonidou C, Stahel PF, Schmidt OI, Alexandrovitch AG, et al. Erythropoietin is neuroprotective, improves functional recovery, and reduces neuronal apoptosis and inflammation in a rodent model of experimental closed head injury. FASEB J. 2005;19(12):1701-3. PubMed PMID: 16099948.
78. Vitellaro-Zuccarello L, Mazzetti S, Madaschi L, Bosisio P, Fontana E, Gorio A, et al. Chronic erythropoietin-mediated effects on the expression of astrocyte markers in a rat model of contusive spinal cord injury. Neuroscience. 2008;151(2):452-66. PubMed PMID: 18065151.
79. Li L, Jiang Q, Ding G, Zhang L, Zhang ZG, Li Q, et al. MRI identification of white matter reorganization enhanced by erythropoietin treatment in a rat model of focal ischemia. Stroke. 2009;40(3):936-41. Epub 2009/01/20. doi: 10.1161/STROKEAHA.108.527713. PubMed PMID: 19150870; PubMed Central PMCID: PMC2730918.
80. Zhang L, Chopp M, Zhang RL, Wang L, Zhang J, Wang Y, et al. Erythropoietin amplifies stroke-induced oligodendrogenesis in the rat. PLoS ONE. 2010;5(6):e11016. Epub 2010/06/17. doi: 10.1371/journal.pone.0011016. PubMed PMID: 20552017; PubMed Central PMCID: PMC2884017.
81. Yamada M, Burke C, Colditz P, Johnson DW, Gobe GC. Erythropoietin protects against apoptosis and increases expression of non-neuronal cell markers in the hypoxia-injured developing brain. J Pathol. 2011. Epub 2011/03/16. doi: 10.1002/path.2862. PubMed PMID: 21404277.
82. Savino C, Pedotti R, Baggi F, Ubiali F, Gallo B, Nava S, et al. Delayed administration of erythropoietin and its non-erythropoietic derivatives ameliorates chronic murine autoimmune encephalomyelitis. J Neuroimmunol. 2006;172(1-2):27-37. Epub 2005/12/13. doi: 10.1016/j.jneuroim.2005.10.016. PubMed PMID: 16337691.
83. Vitellaro-Zuccarello L, Mazzetti S, Madaschi L, Bosisio P, Gorio A, De Biasi S. Erythropoietin-mediated preservation of the white matter in rat spinal cord injury. Neuroscience. 2007;144(3):865-77. Epub 2006/12/05. doi: 10.1016/j.neuroscience.2006.10.023. PubMed PMID: 17141961.
84. Ha-Vinh Leuchter R, Gui L, Hagmann C, Lodygensky GA, Sizonenko S, Martin E, et al. Neuroprotective effects of early Epo in preterm infants: an MRI study. Pediatric Research. 2011;70:28. doi: 10.1038/pr.2011.253.
85. Sugawa M, Sakurai Y, Ishikawa-Ieda Y, Suzuki H, Asou H. Effects of erythropoietin on glial cell development; oligodendrocyte maturation and astrocyte proliferation. Neurosci Res. 2002;44(4):391-403. Epub 2002/11/26. PubMed PMID: 12445627.
86. Genc K, Genc S, Baskin H, Semin I. Erythropoietin decreases cytotoxicity and nitric oxide formation induced by inflammatory stimuli in rat oligodendrocytes. Physiol Res. 2006;55(1):33-8. Epub 2005/04/29. PubMed PMID: 15857166.
87. Mizuno K, Hida H, Masuda T, Nishino H, Togari H. Pretreatment with low doses of erythropoietin ameliorates brain damage in periventricular leukomalacia by targeting late oligodendrocyte progenitors: a rat model. Neonatology. 2008;94(4):255-66. Epub 2008/09/12. doi: 10.1159/000151644. PubMed PMID: 18784421.
88. Oppenheim RW. Cell death during development of the nervous system. Annu Rev Neurosci. 1991;14:453-501. PubMed PMID: 2031577.
89. McDonald JW, Behrens MI, Chung C, Bhattacharyya T, Choi DW. Susceptibility to apoptosis is enhanced in immature cortical neurons. Brain Res. 1997;759(2):228-32. PubMed PMID: 9221941.
90. Digicaylioglu M, Lipton SA. Erythropoietin-mediated neuroprotection involves cross-talk between Jak2 and NF-kappaB signalling cascades. Nature. 2001;412(6847):641-7. PubMed PMID: 11493922.
91. Xiong T, Qu Y, Mu D, Ferriero D. Erythropoietin for neonatal brain injury: opportunity and challenge. Int J Dev Neurosci. 2011. Epub 2011/02/01PMID: 21277366. doi: 10.1016/j.ijdevneu.2010.12.007. PubMed PMID: 21277366.
92. Dzietko M, Felderhoff-Mueser U, Sifringer M, Krutz B, Bittigau P, Thor F, et al. Erythropoietin protects the developing brain against N-methyl-D-aspartate receptor antagonist neurotoxicity. Neurobiol Dis. 2004;15(2):177-87. doi: 10.1016/j.nbd.2003.10.006. PubMed PMID: 15006687.
93. Wang L, Zhang Z, Wang Y, Zhang R, Chopp M. Treatment of stroke with erythropoietin enhances neurogenesis

and angiogenesis and improves neurological function in rats. Stroke. 2004;35(7):1732-7. PubMed PMID: 15178821.

94. Iwai M, Cao G, Yin W, Stetler RA, Liu J, Chen J. Erythropoietin promotes neuronal replacement through revascularization and neurogenesis after neonatal hypoxia/ischemia in rats. Stroke 2007;38:2795-803.
95. Shingo T, Sorokan ST, Shimazaki T, Weiss S. Erythropoietin regulates the in vitro and in vivo production of neuronal progenitors by mammalian forebrain neural stem cells. J Neurosci. 2001;21(24):9733-43. PubMed PMID: 11739582.
96. Osredkar D, Sall JW, Bickler PE, Ferriero DM. Erythropoietin promotes hippocampal neurogenesis in in vitro models of neonatal stroke. Neurobiol Dis. 2010;38(2):259-65. Epub 2010/02/02. PubMed PMID: 20117210; PubMed Central PMCID: PMC2854222.
97. Ransome MI, Turnley AM. Systemically delivered Erythropoietin transiently enhances adult hippocampal neurogenesis. J Neurochem. 2007;102(6):1953-65. PubMed PMID: 17555554.
98. Bocker-Meffert S, Rosenstiel P, Rohl C, Warneke N, Held-Feindt J, Sievers J, et al. Erythropoietin and VEGF promote neural outgrowth from retinal explants in postnatal rats. Invest Ophthalmol Vis Sci. 2002;43(6):2021-6. PubMed PMID: 12037014.
99. Yang Z, Covey MV, Bitel CL, Ni L, Jonakait GM, Levison SW. Sustained neocortical neurogenesis after neonatal hypoxic/ischemic injury. Ann Neurol. 2007;61(3):199-208. Epub 2007/02/09. doi: 10.1002/ana.21068. PubMed PMID: 17286251.
100. Wang L, Chopp M, Gregg SR, Zhang RL, Teng H, Jiang A, et al. Neural progenitor cells treated with EPO induce angiogenesis through the production of VEGF. J Cereb Blood Flow Metab. 2008;28(7):1361-8. PubMed PMID: 18414495.
101. Marzocchi B, Perrone S, Paffetti P, Magi B, Bini L, Tani C, et al. Nonprotein-bound iron and plasma protein oxidative stress at birth. Pediatr Res. 2005;58(6):1295-9. PubMed PMID: 16306211.
102. Buonocore G, Perrone S, Longini M, Paffetti P, Vezzosi P, Gatti MG, et al. Non protein bound iron as early predictive marker of neonatal brain damage. Brain. 2003;126(Pt 5):1224-30. PubMed PMID: 12690060.
103. Collard KJ. Is there a causal relationship between the receipt of blood transfusions and the development of chronic lung disease of prematurity? Med Hypotheses. 2006;66(2):355-64. PubMed PMID: 16236459.
104. Ozment CP, Turi JL. Iron overload following red blood cell transfusion and its impact on disease severity. Biochim Biophys Acta. 2009;1790(7):694-701. Epub 2008/11/11.PubMed PMID: 18992790.
105. Juul SE, Zerzan JC, Strandjord TP, Woodrum DE. Zinc protoporphyrin/heme as an indicator of iron status in NICU patients. J Pediatr. 2003;142(3):273-8. doi: 10.1067/mpd.2003.101. PubMed PMID: 12640375.
106. Wallach I, Zhang J, Hartmann A, van Landeghem FK, Ivanova A, Klar M, et al. Erythropoietin-receptor gene regulation in neuronal cells. Pediatr Res. 2009;65(6):619-24. Epub 2009/02/17. doi: 10.1203/PDR.0b013e31819ea3b8. PubMed PMID: 19218878.
107. Nagai A, Nakagawa E, Choi HB, Hatori K, Kobayashi S, Kim SU. Erythropoietin and erythropoietin receptors in human CNS neurons, astrocytes, microglia, and oligodendrocytes grown in culture. J Neuropathol Exp Neurol. 2001;60(4):386-92. Epub 2001/04/18. PubMed PMID: 11305874.
108. Chong ZZ, Kang JQ, Maiese K. Erythropoietin fosters both intrinsic and extrinsic neuronal protection through modulation of microglia, Akt1, Bad, and caspase-mediated pathways. Br J Pharmacol. 2003;138(6):1107-18. PubMed PMID: 12684267.
109. Kumral A, Tugyan K, Gonenc S, Genc K, Genc S, Sonmez U, et al. Protective effects of erythropoietin against ethanol-induced apoptotic neurodegenaration and oxidative stress in the developing C57BL/6 mouse brain. Brain Res Dev Brain Res. 2005;160(2):146-56. PubMed PMID: 16236368.
110. Chattopadhyay A, Choudhury TD, Bandyopadhyay D, Datta AG. Protective effect of erythropoietin on the oxidative damage of erythrocyte membrane by hydroxyl radical. Biochem Pharmacol. 2000;59(4):419-25. PubMed PMID: 10644050.
111. Zhu C, Kang W, Xu F, Cheng X, Zhang Z, Jia L, et al. Erythropoietin improved neurologic outcomes in newborns with hypoxic-ischemic encephalopathy. Pediatrics. 2009;124(2):e218-26. Epub 2009/08/05. doi: peds.2008-3553 [pii] 10.1542/peds.2008-3553. PubMed PMID: 19651565.

112. Elmahdy H, El-Mashad AR, El-Bahrawy H, El-Gohary T, El-Barbary A, Aly H. Human recombinant erythropoietin in asphyxia neonatorum: pilot trial. Pediatrics. 2010;125(5):e1135-42. PubMed PMID: 20385632.
113. Brown MS, Eichorst D, Lala-Black B, Gonzalez R. Higher cumulative doses of erythropoietin and developmental outcomes in preterm infants. Pediatrics. 2009;124(4):e681-7. Epub 2009/09/30. doi: peds.2008-2701 [pii] PubMed PMID: 19786428.
114. Neubauer AP, Voss W, Wachtendorf M, Jungmann T. Erythropoietin improves neurodevelopmental outcome of extremely preterm infants. Ann Neurol. 2010;67(5):657-66. Epub 2010/05/04. doi: 10.1002/ana.21977. PubMed PMID: 20437563.
115. Bierer R, Peceny MC, Hartenberger CH, Ohls RK. Erythropoietin concentrations and neurodevelopmental outcome in preterm infants. Pediatrics. 2006;118(3):e635-40. PubMed PMID: 16908620.
116. Smeets G, George A. Instantaneous laser Doppler velocimeter using a fast wavelength tracking Michelson interferometer. The Review of scientific instruments. 1978;49(11):1589. doi: 10.1063/1.1135316. PubMed PMID: 18699009.
117. McAdams RM, McPherson RJ, Mayock DE, Juul SE. Neurodevelopmental Outcomes For Extremely Low Birth Weight Infants Given Neonatal High-Dose Erythropoietin. J Perinatol. 2012;In Press.
118. Juul S. Erythropoietin in anemia of prematurity. J Matern Fetal Neona. 2012;25(Suppl 5):80-4. Epub 2012/10/03. doi: 10.3109/14767058.2012.716987. PubMed PMID: 23025776.
119. Mathur AM, Neil JJ, McKinstry RC, Inder TE. Transport, monitoring, and successful brain MR imaging in unsedated neonates. Pediatr Radiol. 2008;38(3):260-4. PubMed PMID: 18175110.
120. Ohls RK. The use of erythropoietin in neonates. Clin Perinatol. 2000;27(3):681-96. PubMed PMID: 10986635.
121. Ohlsson A, Aher SM. Early erythropoietin for preventing red blood cell transfusion in preterm and/or low birth weight infants. Cochrane Database Syst Rev. 2006;3:CD004863. PubMed PMID: 16856062.
122. Ohlsson A, Aher SM. Early erythropoietin for preventing red blood cell transfusion in preterm and/or low birth weight infants. Cochrane Database Syst Rev. 2014;4:CD004863. doi: 10.1002/14651858.CD004863.pub4. PubMed PMID: 24771408.
123. Doege C, Pritsch M, Fruhwald MC, Bauer J. An association between infantile haemangiomas and erythropoietin treatment in preterm infants. Arch Dis Child Fetal Neonatal Ed. 2012;97(1):F45-9. Epub 2011/05/07. doi: 10.1136/adc.2010.187344. PubMed PMID: 21546402.
124. Fauchere JC, Koller BM, Tschopp A, Dame C, Ruegger C, Bucher HU, et al. Safety of Early High-Dose Recombinant Erythropoietin for Neuroprotection in Very Preterm Infants. J Pediatr. 2015. doi: 10.1016/j.jpeds.2015.02.052. PubMed PMID: 25863661.
125. Leuchter RH, Gui L, Poncet A, Hagmann C, Lodygensky GA, Martin E, et al. Association between early administration of high-dose erythropoietin in preterm infants and brain MRI abnormality at term-equivalent age. JAMA. 2014;312(8):817-24. doi: 10.1001/jama.2014.9645. PubMed PMID: 25157725.
126. O'Gorman RL, Bucher HU, Held U, Koller BM, Huppi PS, Hagmann CF, et al. Tract-based spatial statistics to assess the neuroprotective effect of early erythropoietin on white matter development in preterm infants. Brain. 2014. doi: 10.1093/brain/awu363. PubMed PMID: 25534356.
127. Aher SM, Ohlsson A. Late erythropoietin for preventing red blood cell transfusion in preterm and/or low birth weight infants. Cochrane Database Syst Rev. 2014;4:CD004868. doi: 10.1002/14651858.CD004868.pub4. PubMed PMID: 24760628.
128. Aher SM, Ohlsson A. Early versus late erythropoietin for preventing red blood cell transfusion in preterm and/or low birth weight infants. Cochrane Database Syst Rev. 2012;10:CD004865. doi: 10.1002/14651858.CD004865.pub3. PubMed PMID: 23076909.
129. Xu XJ, Huang HY, Chen HL. Erythropoietin and retinopathy of prematurity: a meta-analysis. Eur J Pediatr. 2014. doi: 10.1007/s00431-014-2332-4. PubMed PMID: 24849614.
130. Wu YW, Bauer LA, Ballard RA, Ferriero DM, Glidden DV, Mayock DE, et al. Erythropoietin for neuroprotection in neonatal encephalopathy: safety and pharmacokinetics. Pediatrics. 2012;130(4):683-91. Epub 2012/09/26. doi: 10.1542/peds.2012-0498. PubMed PMID: 23008465; PubMed Central PMCID: PMC3457622.
131. Ohls RK, Ehrenkranz RA, Wright LL, Lemons JA, Korones SB, Stoll BJ, et al. Effects of early erythropoietin therapy

on the transfusion requirements of preterm infants below 1250 grams birth weight: a multicenter, randomized, controlled trial. Pediatrics. 2001;108(4):934-42. PubMed PMID: 11581447.

132. AAP. Screening examination of premature infants for retinopathy of prematurity. Pediatrics. 2006;117(2):572-6. Epub 2006/02/03. doi: 10.1542/peds.2005-2749. PubMed PMID: 16452383.
133. Dammann O, Naples M, Bednarek F, Shah B, Kuban KC, O'Shea TM, et al. SNAP-II and SNAPPE-II and the risk of structural and functional brain disorders in extremely low gestational age newborns: the ELGAN study. Neonatology. 2010;97(2):71-82. Epub 2009/08/13. doi: 10.1159/000232588. PubMed PMID: 19672122; PubMed Central PMCID: PMC2790760.
134. Leviton A, Allred EN, Kuban KC, Hecht JL, Onderdonk AB, O'Shea T M, et al. Microbiologic and histologic characteristics of the extremely preterm infant's placenta predict white matter damage and later cerebral palsy. the ELGAN study. Pediatric Research. 2010;67(1):95-101. Epub 2009/09/12. doi: 10.1203/PDR.0b013e3181bf5fab. PubMed PMID: 19745780; PubMed Central PMCID: PMC2794973.
135. Leviton A, Dammann O, Engelke S, Allred E, Kuban KC, O'Shea TM, et al. The clustering of disorders in infants born before the 28th week of gestation. Acta Paediatrica. 2010;99(12):1795-800. PubMed PMID: 20712837.
136. O'Shea TM, Allred EN, Kuban KC, Dammann O, Paneth N, Fichorova R, et al. Elevated concentrations of inflammation-related proteins in postnatal blood predict severe developmental delay at 2 years of age in extremely preterm infants. The Journal of Pediatrics. 2012;160(3):395-401 e4. Epub 2011/10/18. doi: 10.1016/j.jpeds.2011.08.069. PubMed PMID: 22000304; PubMed Central PMCID: PMC3279610.
137. Franz AR, Mihatsch WA, Sander S, Kron M, Pohlandt F. Prospective randomized trial of early versus late enteral iron supplementation in infants with a birth weight of less than 1301 grams. Pediatrics. 2000;106(4):700-6. PubMed PMID: 11015511.
138. Howie SRC. Blood sample volumes in child health research: review of safe limits. Bulletin of the World Health Organization. 2011;89(1):46-53.
139. Ment LR, Bada HS, Barnes P, Grant PE, Hirtz D, Papile LA, et al. Practice parameter: neuroimaging of the neonate: report of the Quality Standards Subcommittee of the American Academy of Neurology and the Practice Committee of the Child Neurology Society. Neurology. 2002;58(12):1726-38. Epub 2002/06/27. PubMed PMID: 12084869.
140. Habas PA, Kim K, Corbett-Detig JM, Rousseau F, Glenn OA, Barkovich AJ, et al. A spatiotemporal atlas of MR intensity, tissue probability and shape of the fetal brain with application to segmentation. Neuroimage. 2010;53(2):460-70. Epub 2010/07/06. PubMed PMID: 20600970; PubMed Central PMCID: PMC2930902.
141. Habas PA, Kim K, Rousseau F, Glenn OA, Barkovich AJ, Studholme C. Atlas-based segmentation of the germinal matrix from in utero clinical MRI of the fetal brain. Med Image Comput Comput Assist Interv. 2008;11(Pt 1):351-8. Epub 2008/11/05. PubMed PMID: 18979766; PubMed Central PMCID: PMC3343876.
142. Rousseau F, Habas PA, Studholme C. A supervised patch-based approach for human brain labeling. IEEE Trans Med Imaging. 2011;In Press.
143. Davatzikos C, Vaillant M, Resnick SM, Prince JL, Letovsky S, Bryan RN. A computerized approach for morphological analysis of the corpus callosum. J Comput Assist Tomogr. 1996;20(1):88-97. Epub 1996/01/01. PubMed PMID: 8576488.
144. Friston K, Holmes A, Worsley K, Poline J, Frith C, Frackowiak RSJ. Statistical parametric maps in functional imaging: A general linear approach. Human Brain Mapping. 1995;2:189-210.
145. Nichols TE, Holmes AP. Nonparametric permutation tests for functional neuroimaging: a primer with examples. Human Brain Mapping. 2002;15(1):1-25. Epub 2001/12/18. PubMed PMID: 11747097.
146. Habas PA, Scott JA, Roosta A, Rajagopalan V, Kim K, Rousseau F, et al. Early folding patterns and asymmetries of the normal human brain detected from in utero MRI. Cereb Cortex. 2011;In Press.
147. Rodriguez-Carranza CE, Mukherjee P, Vigneron D, Barkovich J, Studholme C. A framework for in vivo quantification of regional brain folding in premature neonates. Neuroimage. 2008;41(2):462-78. Epub 2008/04/11. doi: S1053-8119(08)00031-1 [pii] 10.1016/j.neuroimage.2008.01.008. PubMed PMID: 18400518; PubMed Central PMCID: PMC2741002.
148. Lopes A, Brodlie K. Improving the robustness and accuracy of the marching cubes algorithm for isosurfacing. .

IEEE Trans Vis Comput Graph. 2003;9:16-29.
149. Kidokoro H, Neil JJ, Inder TE. New MR Imaging Assessment Tool to Define Brain Abnormalities in Very Preterm Infants at Term. AJNR Am J Neuroradiol. 2013. Epub 2013/04/27. doi: 10.3174/ajnr.A3521. PubMed PMID: 23620070.
150. Habas PA, Corbett-Detig JM, Kim K, Rousseau F, Glenn OA, Barkovich AJ, et al., editors. Global and regional patterns of tissue volume growth in the normal fetal brain from in utero MRI. 16th Annual Meeting of the Organization for Human Brain Mapping; 2010; Barcelona, Spain.
151. Jenkinson M, Smith S. A global optimisation method for robust affine registration of brain images. Med Image Anal. 2001;5(2):143-56. Epub 2001/08/23. doi: S1361841501000366 [pii]. PubMed PMID: 11516708.
152. Jenkinson M. Fast, automated, N-dimensional phase-unwrapping algorithm. Magn Reson Med. 2003;49(1):193-7. Epub 2003/01/02. doi: 10.1002/mrm.10354. PubMed PMID: 12509838.
153. Smith SM, Jenkinson M, Johansen-Berg H, Rueckert D, Nichols TE, Mackay CE, et al. Tract-based spatial statistics: voxelwise analysis of multi-subject diffusion data. Neuroimage. 2006;31(4):1487-505. Epub 2006/04/21. doi: S1053-8119(06)00138-8 [pii] 10.1016/j.neuroimage.2006.02.024. PubMed PMID: 16624579.
154. Richards T, Stevenson J, Crouch J, Johnson LC, Maravilla K, Stock P, et al. Tract-Based Spatial Statistics of Diffusion Tensor Imaging in Adults with Dyslexia. American Journal of Neuroradiology. 2008;29:1134-9.
155. Weaver KE, Richards TL, Liang O, Laurino MY, Samii A, Aylward EH. Longitudinal diffusion tensor imaging in Huntington's Disease. Exp Neurol. 2009;216(2):525-9. Epub 2009/03/26. PubMed PMID: 19320010.
156. Heide AC, Richards TL, Alvord EC, Jr., Peterson J, Rose LM. Diffusion imaging of experimental allergic encephalomyelitis. Magn Reson Med. 1993;29(4):478-84. PubMed PMID: 8464364.
157. Richards TL, Alvord EC, Jr., He Y, Petersen K, Peterson J, Cosgrove S, et al. Experimental allergic encephalomyelitis in non-human primates: diffusion imaging of acute and chronic brain lesions. Mult Scler. 1995;1(2):109-17. PubMed PMID: 9345461.
158. Chen G, Shi JX, Hang CH, Xie W, Liu J, Liu X. Inhibitory effect on cerebral inflammatory agents that accompany traumatic brain injury in a rat model: a potential neuroprotective mechanism of recombinant human erythropoietin (rhEPO). Neurosci Lett. 2007;425(3):177-82. PubMed PMID: 17825990.
159. Bednarek N, Svedin P, Garnotel R, Favrais G, Loron G, Schwendiman l, et al. Increased MMP-9 and TIMP-1 in mouse neonatal brain and plasma and in human neonatal plasma after hypoxia–ischemia: a potential marker of neonatal encephalopathy. Pediatr Res. 2012;71:63-70.
160. Douglas-Escobar M, Yang C, Bennett J, Shuster J, Theriaque D, Leibovici A, et al. A pilot study of novel biomarkers in neonates with hypoxic-ischemic encephalopathy. Pediatric Research. 2010;68(6):531-6. Epub 2010/08/26. doi: 10.1203/PDR.0b013e3181f85a03. PubMed PMID: 20736881.
161. Ehrenreich H, Kastner A, Weissenborn K, Streeter J, Sperling S, Wang KK, et al. Circulating damage marker profiles support a neuroprotective effect of erythropoietin in ischemic stroke patients. Mol Med. 2011. Epub 2011/09/14. doi: 10.2119/molmed.2011.00259. PubMed PMID: 21912808.
162. Ennen CS, Huisman TA, Savage WJ, Northington FJ, Jennings JM, Everett AD, et al. Glial fibrillary acidic protein as a biomarker for neonatal hypoxic-ischemic encephalopathy treated with whole-body cooling. Am J Obstet Gynecol. 2011;205(3):251 e1-7. Epub 2011/07/26. doi: 10.1016/j.ajog.2011.06.025. PubMed PMID: 21784396.
163. Bembea MM, Savage W, Strouse JJ, Schwartz JM, Graham E, Thompson CB, et al. Glial fibrillary acidic protein as a brain injury biomarker in children undergoing extracorporeal membrane oxygenation. Pediatr Crit Care Med. 2011;12(5):572-9. Epub 2010/11/09. doi: 10.1097/PCC.0b013e3181fe3ec7. PubMed PMID: 21057367.
164. Honda M, Tsuruta R, Kaneko T, Kasaoka S, Yagi T, Todani M, et al. Serum glial fibrillary acidic protein is a highly specific biomarker for traumatic brain injury in humans compared with S-100B and neuron-specific enolase. J Trauma. 2010;69(1):104-9. Epub 2010/01/23. doi: 10.1097/TA.0b013e3181bbd485. PubMed PMID: 20093985.
165. Florio P, Luisi S, Moataza B, Torricelli M, Iman I, Hala M, et al. High urinary concentrations of activin A in asphyxiated full-term newborns with moderate or severe hypoxic ischemic encephalopathy. Clin Chem. 2007;53(3):520-2. PubMed PMID: 17259240.
166. Florio P, Perrone S, Luisi S, Vezzosi P, Longini M, Marzocchi B, et al. Increased plasma concentrations of activin a predict intraventricular hemorrhage in preterm newborns. Clin Chem. 2006;52(8):1516-21. Epub 2006/06/03. doi:

10.1373/clinchem.2005.065979. PubMed PMID: 16740650.

167. Florio P, Reis FM, Severi FM, Luisi S, Imperatore A, Palumbo MA, et al. Umbilical cord serum activin A levels are increased in pre-eclampsia with impaired blood flow in the uteroplacental and fetal circulation. Placenta. 2006;27(4-5):432-7. Epub 2005/07/30. doi: 10.1016/j.placenta.2005.04.008. PubMed PMID: 16051348.
168. Florio P, Luisi S, Bruschettini M, Grutzfeld D, Dobrzanska A, Bruschettini P, et al. Cerebrospinal fluid activin a measurement in asphyxiated full-term newborns predicts hypoxic ischemic encephalopathy. Clin Chem. 2004;50(12):2386-9. Epub 2004/11/26. doi: 10.1373/clinchem.2004.035774. PubMed PMID: 15563489.
169. Laptook AR, O'Shea TM, Shankaran S, Bhaskar B. Adverse neurodevelopmental outcomes among extremely low birth weight infants with a normal head ultrasound: prevalence and antecedents. Pediatrics. 2005;115(3):673-80. PubMed PMID: 15741371.
170. Hintz SR, Kendrick DE, Vohr BR, Poole WK, Higgins RD. Changes in neurodevelopmental outcomes at 18 to 22 months' corrected age among infants of less than 25 weeks' gestational age born in 1993-1999. Pediatrics. 2005;115(6):1645-51. Epub 2005/06/03. doi: 10.1542/peds.2004-2215. PubMed PMID: 15930228.
171. Hack M, Taylor HG, Drotar D, Schluchter M, Cartar L, Wilson-Costello D, et al. Poor predictive validity of the Bayley Scales of Infant Development for cognitive function of extremely low birth weight children at school age. Pediatrics. 2005;116(2):333-41. PubMed PMID: 16061586.
172. Roberts G, Anderson PJ, Doyle LW. The stability of the diagnosis of developmental disability between ages 2 and 8 in a geographic cohort of very preterm children born in 1997. Arch Dis Child. 2010;95(10):786-90. Epub 2009/10/16. doi: 10.1136/adc.2009.160283. PubMed PMID: 19828882.
173. Schmidt B, Anderson PJ, Doyle LW, Dewey D, Grunau RE, Asztalos EV, et al. Survival without disability to age 5 years after neonatal caffeine therapy for apnea of prematurity. JAMA. 2012;307(3):275-82. Epub 2012/01/19. doi: 10.1001/jama.2011.2024. PubMed PMID: 22253394.
174. Potharst ES, Houtzager BA, van Sonderen L, Tamminga P, Kok JH, Last BF, et al. Prediction of cognitive abilities at the age of 5 years using developmental follow-up assessments at the age of 2 and 3 years in very preterm children. Dev Med Child Neurol. 2012;54(3):240-6. Epub 2011/12/23. doi: 10.1111/j.1469-8749.2011.04181.x. PubMed PMID: 22188215.
175. Munck P, Niemi P, Lapinleimu H, Lehtonen L, Haataja L. Stability of cognitive outcome from 2 to 5 years of age in very low birth weight children. Pediatrics. 2012;129(3):503-8. Epub 2012/03/01. doi: 10.1542/peds.2011-1566. PubMed PMID: 22371467.
176. Bayley N. Bayley scales of infant and toddler development, third edition. San Antonio, TX: Harcourt Assessment, Inc; 2006.
177. Vohr BR, Stephens BE, Higgins RD, Bann CM, Hintz SR, Das A, et al. Are Outcomes of Extremely Preterm Infants Improving? Impact of Bayley Assessment on Outcomes. The Journal of Pediatrics. 2012. Epub 2012/03/17. doi: 10.1016/j.jpeds.2012.01.057. PubMed PMID: 22421261.
178. Msall ME. Measuring outcomes after extreme prematurity with the Bayley-III Scales of infant and toddler development: a cautionary tale from Australia. Arch Pediatr Adolesc Med. 2010;164(4):391-3. PubMed PMID: 20368495.
179. Anderson PJ, De Luca CR, Hutchinson E, Roberts G, Doyle LW. Underestimation of developmental delay by the new Bayley-III Scale. Arch Pediatr Adolesc Med. 2010;164(4):352-6. Epub 2010/04/07. doi: 10.1001/archpediatrics.2010.20. PubMed PMID: 20368488.
180. Robertson CM, Hendson L, Biggs WS, Acton BV. Application of the Flynn effect for the Bayley III Scales. Arch Pediatr Adolesc Med. 2010;164(11):1072-3; author reply 3. Epub 2010/11/03. doi: 10.1001/archpediatrics.2010.199. PubMed PMID: 21041604.
181. Msall ME. Overestimating neuroprotection in congenital heart disease: problems with Bayley III outcomes. Pediatrics. 2011;128(4):e993-4. Epub 2011/09/29. doi: 10.1542/peds.2011-2166. PubMed PMID: 21949147.
182. Lowe JR, Erickson SJ, Schrader R, Duncan AF. Comparison of the Bayley II Mental Developmental Index and the Bayley III Cognitive Scale: are we measuring the same thing? Acta Paediatrica. 2012;101(2):e55-8. Epub 2011/11/08. doi: 10.1111/j.1651-2227.2011.02517.x. PubMed PMID: 22054168.
183. Silveira RC, Filipouski GR, Goldstein DJ, O’Shea TM, Procianoy RS. Agreement between Bayley Scales Second and

Third Edition Assessments of Very Low Birth Weight Infants. Archives of Pediatric and Adolescent Medicine. 2012;In Press.

184. Kuban KC, O'Shea M, Allred E, Leviton A, Gilmore H, DuPlessis A, et al. Video and CD-ROM as a training tool for performing neurologic examinations of 1-year-old children in a multicenter epidemiologic study. J Child Neurol. 2005;20(10):829-31. Epub 2006/01/19. PubMed PMID: 16417880.
185. Kuban KC, Allred EN, O'Shea M, Paneth N, Pagano M, Leviton A. An algorithm for identifying and classifying cerebral palsy in young children. The Journal of Pediatrics. 2008;153(4):466-72. Epub 2008/06/07. doi: 10.1016/j.jpeds.2008.04.013. PubMed PMID: 18534210; PubMed Central PMCID: PMC2581842.
186. Palisano RJ, Cameron D, Rosenbaum PL, Walter SD, Russell D. Stability of the gross motor function classification system. Dev Med Child Neurol. 2006;48(6):424-8. Epub 2006/05/17. doi: 10.1017/S0012162206000934. PubMed PMID: 16700931.
187. Rosenbaum PL, Palisano RJ, Bartlett DJ, Galuppi BE, Russell DJ. Development of the Gross Motor Function Classification System for cerebral palsy. Dev Med Child Neurol. 2008;50(4):249-53. Epub 2008/03/06. doi: 10.1111/j.1469-8749.2008.02045.x. PubMed PMID: 18318732.
188. Johnson S, Wolke D, Hennessy E, Marlow N. Educational outcomes in extremely preterm children: neuropsychological correlates and predictors of attainment. Dev Neuropsychol. 2011;36(1):74-95. Epub 2011/01/22. doi: 10.1080/87565641.2011.540541. PubMed PMID: 21253992.
189. Johnson S, Marlow N. Preterm Birth and Childhood Psychiatric Disorders. Pediatric Research. 2011. Epub 2011/02/04. doi: 10.1203/PDR.0b013e318212faa0. PubMed PMID: 21289534.
190. Mulder H, Pitchford NJ, Marlow N. Inattentive behaviour is associated with poor working memory and slow processing speed in very pre-term children in middle childhood. Br J Educ Psychol. 2011;81(1):147-60. Epub 2011/03/12. doi: 10.1348/000709910X505527. PubMed PMID: 21391967.
191. Lind A, Korkman M, Lehtonen L, Lapinleimu H, Parkkola R, Matomaki J, et al. Cognitive and neuropsychological outcomes at 5 years of age in preterm children born in the 2000s. Dev Med Child Neurol. 2011;53(3):256-62. Epub 2010/12/21. doi: 10.1111/j.1469-8749.2010.03828.x. PubMed PMID: 21166668.
192. Raz S, Debastos AK, Newman JB, Batton D. Extreme prematurity and neuropsychological outcome in the preschool years. J Int Neuropsychol Soc. 2010;16(1):169-79. PubMed PMID: 19900351.
193. Limperopoulos C. Autism spectrum disorders in survivors of extreme prematurity. Clin Perinatol. 2009;36(4):791-805, vi. PubMed PMID: 19944836.
194. Kuban KC, Allred EN, O'Shea TM, Paneth N, Westra S, Miller C, et al. Developmental correlates of head circumference at birth and two years in a cohort of extremely low gestational age newborns. The Journal of Pediatrics. 2009;155(3):344-9 e1-3. PubMed Central PMCID: PMC2803763.
195. Kontis D, Catani M, Cuddy M, Walshe M, Nosarti C, Jones D, et al. Diffusion tensor MRI of the corpus callosum and cognitive function in adults born preterm. Neuroreport. 2009;20(4):424-8. Epub 2009/02/17. doi: 10.1097/WNR.0b013e328325a8f9. PubMed PMID: 19218872.
196. Gaddlin PO, Finnstrom O, Samuelsson S, Wadsby M, Wang C, Leijon I. Academic achievement, behavioural outcomes and MRI findings at 15 years of age in very low birthweight children. Acta Paediatrica. 2008;97(10):1426-32. Epub 2008/07/16. doi: 10.1111/j.1651-2227.2008.00925.x. PubMed PMID: 18624991.
197. Nagy Z, Ashburner J, Andersson J, Jbabdi S, Draganski B, Skare S, et al. Structural correlates of preterm birth in the adolescent brain. Pediatrics. 2009;124(5):e964-72. PubMed PMID: 19858152.
198. Mathur AM, Neil JJ, Inder TE. Understanding brain injury and neurodevelopmental disabilities in the preterm infant: the evolving role of advanced magnetic resonance imaging. Semin Perinatol. 2010;34(1):57-66. PubMed PMID: 20109973.
199. Wustenberg T, Begemann M, Bartels C, Gefeller O, Stawicki S, Hinze-Selch D, et al. Recombinant human erythropoietin delays loss of gray matter in chronic schizophrenia. Mol Psychiatry. 2010. PubMed PMID: 20479759.
200. Northam GB, Liegeois F, Chong WK, Wyatt JS, Baldeweg T. Total brain white matter is a major determinant of IQ in adolescents born preterm. Ann Neurol. 2011;69(4):702-11. Epub 2011/03/11. doi: 10.1002/ana.22263. PubMed PMID: 21391229.

201. Woodward LJ, Clark CA, Pritchard VE, Anderson PJ, Inder TE. Neonatal white matter abnormalities predict global executive function impairment in children born very preterm. Dev Neuropsychol. 2011;36(1):22-41. Epub 2011/01/22. doi: 10.1080/87565641.2011.540530. PubMed PMID: 21253989.
202. Screening examination of premature infants for retinopathy of prematurity. Pediatrics. 2006;117(2):572-6. PubMed PMID: 16452383.
203. The International Classification of Retinopathy of Prematurity revisited. Arch Ophthalmol. 2005;123(7):991-9. PubMed PMID: 16009843.
204. Fisher LD, Dixon DO, Herson J, Frankowski RK, Hearron MS, Peace KE. Intention to treat in clinical trials. In: Peace KE, editor. Statistical Issues in Drug Research and Development. New York: Marcel Dekker; 1991. p. 331-50.
205. Diggle PJ, Heagerty PJ, Liang KY, Zeger SL. Analysis of Longitudinal Data. Second Edition ed: Oxford University Press; 2002.
206. Vermont Oxford Network ELBW Follow-up Report Birth Year 2008 All Centers [Internet]Sept 2011.
207. Volpe JJ. Cerebellum of the premature infant: rapidly developing, vulnerable, clinically important. J Child Neurol. 2009;24(9):1085-104. PubMed PMID: 19745085; PubMed Central PMCID: PMC2799249.
208. Limperopoulos C, Chilingaryan G, Guizard N, Robertson RL, du Plessis AJ. Cerebellar injury in the premature infant is associated with impaired growth of specific cerebral regions. Pediatr Res. 2010. Epub 2010/04/15. doi: 10.1203/PDR.0b013e3181e1d032. PubMed PMID: 20389260.
209. Limperopoulos C, Bassan H, Gauvreau K, Robertson RL, Jr., Sullivan NR, Benson CB, et al. Does cerebellar injury in premature infants contribute to the high prevalence of long-term cognitive, learning, and behavioral disability in survivors? Pediatrics. 2007;120(3):584-93. PubMed PMID: 17766532.
210. Limperopoulos C, Soul JS, Gauvreau K, Huppi PS, Warfield SK, Bassan H, et al. Late gestation cerebellar growth is rapid and impeded by premature birth. Pediatrics. 2005;115(3):688-95. PubMed PMID: 15741373.
211. Messerschmidt A, Pataraia A, Helmer H, Kasprian G, Sauer A, Brugger PC, et al. Fetal MRI for prediction of neonatal mortality following preterm premature rupture of the fetal membranes. Pediatr Radiol. 2011;41(11):1416-20. Epub 2011/09/13. doi: 10.1007/s00247-011-2199-8. PubMed PMID: 21909717.
212. Messerschmidt A, Fuiko R, Prayer D, Brugger PC, Boltshauser E, Zoder G, et al. Disrupted cerebellar development in preterm infants is associated with impaired neurodevelopmental outcome. Eur J Pediatr. 2008;167(10):1141-7. Epub 2008/01/04. doi: 10.1007/s00431-007-0647-0. PubMed PMID: 18172680.
213. Limperopoulos C, Robertson RL, Sullivan NR, Bassan H, du Plessis AJ. Cerebellar injury in term infants: clinical characteristics, magnetic resonance imaging findings, and outcome. Pediatr Neurol. 2009;41(1):1-8. doi: 10.1016/j.pediatrneurol.2009.02.007. PubMed PMID: 19520266.
214. Tam EW, Rosenbluth G, Rogers EE, Ferriero DM, Glidden D, Goldstein RB, et al. Cerebellar hemorrhage on magnetic resonance imaging in preterm newborns associated with abnormal neurologic outcome. J Pediatr. 2011;158(2):245-50. Epub 2010/09/14. doi: 10.1016/j.jpeds.2010.07.049. PubMed PMID: 20833401; PubMed Central PMCID: PMC3010295.
215. O'Brien PC, Fleming TR. A multiple testing procedure for clinical trials. Biometrics. 1979;35(3):549-56. Epub 1979/09/01. PubMed PMID: 497341.

Summary of Amendments Version 2 to Version 5

Secondary Objectives were modified to include:

- To determine the incidence of acute kidney injury (AKI) in this cohort of extremely low gestational age neonates (REPAIReD ancillary study).

- Collection of 10 urine samples from baseline to discharge was added

The 2-year outcome was defined as 22-26 months corrected age instead of 24-26 months

There were multiple clarifications of procedures and evaluations.
For example:

Interventions and Duration
Enrollment will occur prior to 24 hours of age. After enrollment but prior to the first dose of study drug, a cranial ultrasound will be obtained to document whether an intracranial hemorrhage has occurred prior to dosing. Baseline blood will be drawn to measure Epo concentration, inflammatory mediators and biomarkers of brain injury. Subjects will be randomized to Epo treatment or vehicle, based on randomization by the data coordinating center (DCC). Each subject will be on the study (intervention + time off intervention) until completion of the follow-up visit at 24-26 months.

Group 1 (Epo): 1000 U/kg/dose i.v. every 48 hours x 6 doses. Beginning at 13 days, and continuing to 32-6/7 weeks, maintenance Epo (400 U/kg/dose three times per week) will be administered by s.c. injection.

Group 2: (Placebo): Saline placebo i.v. every 48 hours x 6 doses. Control infants will not receive any maintenance doses of study drug, and will not undergo any subcutaneous injections, but, similar to treated subjects, will have an adhesive bandage placed (so-called "sham injections"). To maintain blinding, treatments will be administered by the research nurse behind blinds.

Changed to:

Interventions and Duration
Enrollment will occur prior to 24 hours of age. After enrollment but prior to the first dose of study drug, a cranial ultrasound (CUS) will be obtained to document whether an intracranial hemorrhage has occurred prior to dosing (while the goal will be to obtain the ultrasound prior to study drug dosing, a 6 hour grace period will be allowed). Baseline blood will be drawn to measure Epo concentration, inflammatory mediators and biomarkers of brain injury. Subjects will be randomized to Epo treatment or placebo by the data coordinating center (DCC). Each subject will be on the study (intervention + time off intervention) until completion of the follow-

up visit at 22-26 months corrected age. After the in-person 2-year visit, with parental permission, PENUT subjects will continue to be followed at 6 month intervals by phone.

Group 1 (Epo):
1. High dose Epo (1000 units/kg) will be administered intravenously every 48 hours x 6 doses. Dosing will be based on birth weight. Study drug may be administered by the bedside nurse or a research nurse, given that the study drug appears identical.

2. Maintenance Epo (400 units/kg) will be administered by subcutaneous injection three times weekly. Dosing will be adjusted weekly after the subject has regained birth weight. A nurse who is not directly involved with the care of the patient, and who will maintain confidentiality will administer all treatments. A cohort of charge nurses, or General Clinical Research Center (GCRC) nurses would be ideal for this task.

Group 2 (Placebo):
1. Placebo (saline) will be given intravenously every 48 hours x 6 doses. The volume of the saline placebo will be equivalent to the volume which would be administered if the subject was receiving active drug. Study drug may be administered by the bedside nurse or a research nurse, given that the study drug appears identical.

2. Empty syringes (SHAM doses) will be delivered to infants in the control group however they will not undergo subcutaneous injections. Maintenance doses will be administered behind blinds or in a private room, and the "injection site" will be covered as described above. A nurse who is not directly involved with the care of the patient, and who will maintain confidentiality will administer all treatments. A cohort of charge nurses, or General Clinical Research Center (GCRC) nurses would be ideal for this task.

Exceptional situations:

- If a dose of study drug is unavoidably delayed or missed, the dose should be given as soon as possible. This should be recorded as a study deviation. The normal schedule of administration should then be resumed. At least 24 hours must separate the high dose study drug doses. This allows the plasma Epo concentration to return to baseline.

- If severe edema (anasarca) or other circumstances prohibit the use of subcutaneous injection, up to three doses of Epo or saline placebo may be given intravenously using the normal IV protocol. In this case, pharmacy must be notified so they can provide an appropriate study drug formulation for IV use (saline rather than an empty syringe used for sham injection). This should not be recorded as a deviation for up to 3 doses. Subsequent IV doses would be considered a deviation.

- If the baby's IV is removed prior to completing the 6 intravenous study drug doses, the dose can be administered subcutaneously. This should be recorded as a study deviation.

Added in Protocol 5:

Objective 5. To establish the incidence of acute kidney injury in ELGANs, and to determine whether Epo treatment influences this.
Hypothesis 5. Epo treatment may affect kidney development and incidence of acute kidney injury.

Urine collections
Rationale: Little is known about acute kidney injury (AKI) in extremely preterm infants. Serum creatinine (SCr) and urine output are not ideal markers to define AKI, especially in neonates. Even in adults, the increase in SCr is known to be a relatively late occurrence. The use of SCr in neonates is complicated by the fact that in the first few days of life, it reflects maternal SCr.

Urine biomarkers of cell injury/repair are felt to have great potential. However, before these novel biomarkers can be used clinically, we must evaluate their ability to predict a SCr rise, as well as short and long-term clinical outcomes. The goal of collecting detailed information about renal function in these infants is ultimately to improve the ability to diagnose AKI, and to better understand the epidemiology and pathophysiology of AKI in extremely low gestational age neonates. BUN and creatinine are commonly checked as part of routine care of extreme preterm infants. When obtained for clinical indications, these values will be recorded.

Neonatal AKI has been classically defined by oliguria/anuria and/or a persistent SCr elevation. We will use categorical definitions to make the diagnosis of AKI, similar to definitions published by KDIGO (2012) http://www.kdigo.org and the AKIN groups (2007). Jetton, et al., proposed a modification of the adult Acute Kidney Injury Network Criteria (AKIN) definition of AKI as a standardized definition of AKI for neonates based on a rise in SCr from a previously documented low, rather than on an absolute SCr threshold.

Stage 0: No change or rise < 0.3 mg/dL
Stage 1: Increase in SCr 0.3 mg/dL or a SCr 150–200% from previous trough value
Stage 2: Increase in SCr 201–300% from previous trough value
Stage 3: Increase in SCr > 300% from previous trough value or 2.5 mg/dL or need for dialysis

Urine will be obtained at timed intervals as shown in Figure 4. With the exception of the initial urine, which will be collected at the time of enrollment, if the date of urine collection falls on a Saturday or Sunday, the urine will be collected on the closest weekday. Do not delay study drug dosing if no urine is available at enrollment, but still obtain first urine available. Urine samples are requested at the time of enrollment, on day 3 to 5, day 7 to 9, with a preference for day 7 if that is possible, day 14 ± 1 day, and then during week 3, 4, 6, 8, 10 and 12. If possible, pick one day of the week to collect urines, and do this consistently. This is to minimize the work for the site coordinator as much as possible.

Figure 4.

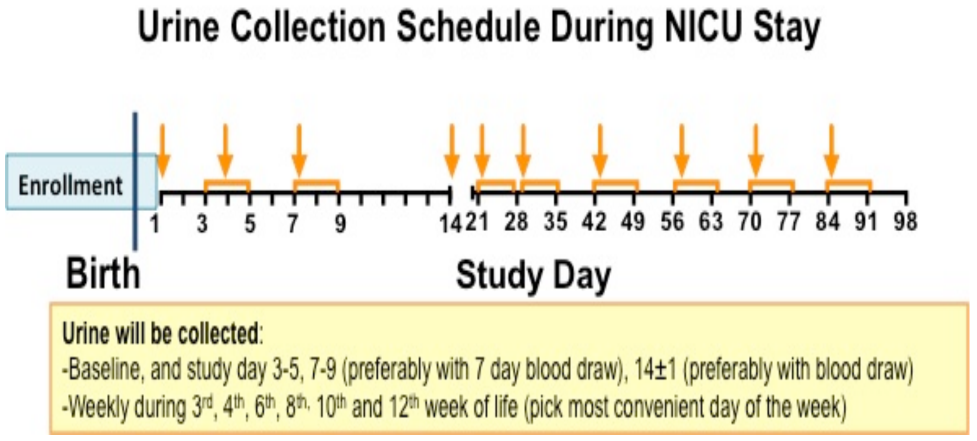

The urine samples will be banked at UW for future analyses of biomarkers (neutrophil gelatinase associated lipocalin (NGAL), KIM-1, osteopontin (OPN), cystatin C (CysC), uromodulin (UMOD), epithelial growth factor (EGF) and urine β2-Microglobulin (β2MG). There are increasing data in older children as to the validity of such markers for diagnosis of AKI, but very little data in preterm neonates. Additionally, prior to including these markers as part of the neonatal AKI definition, more knowledge is needed on other clinical factors which may affect urinary concentrations of these biomarkers independent of renal function (e.g. inflammation, NEC or other neonatal specific conditions). Advantages to eventually including novel renal injury biomarkers in a future definition of neonatal AKI are that they would ideally represent actual renal tubular injury versus the solely functional markers of AKI currently used.

4) *Renal function.* Daily weights, and fluid intake and output will be recorded as available in the chart. BUN and creatinine will be recorded as available in the chart. If renal ultrasounds are done for clinical indications, the results from these studies will be recorded. Ten urine samples will be collected non-invasively at timed intervals from the time of enrollment until discharge.

Exclusion criteria were changed from V2 to V5 as follows:

Exclusion Criteria (V2)

1. Major life-threatening anomalies (brain, cardiac, fetal diagnosis of chromosomal anomalies)
2. Hematopoietic crises such as DIC or hemolysis due to blood group incompatibilities
3. Polycythemia (hematocrit > 65%)
4. Congenital infection
5. Prior use of Epo

Exclusion Criteria (V5)

- Known major life-threatening anomalies (e.g. fetal diagnosis of brain, cardiac, or renal malformations
- Known or suspected chromosomal anomalies
- Severe hematologic crises such as clinically evident disseminated intravascular coagulopathy, twin-twin transfusion such that 1 twin is not eligible due to polycythemia or hydrops
- Polycythemia (hematocrit > 65%)
- Hydrops fetalis
- Known congenital infection such as toxoplasmosis, CMV, rubella or syphilis.
- Prior administration of erythropoietin to the baby
- Infant is likely to die due to severity of illness or withdrawal of support is being considered prior to enrollment

Table 4. Phase III Trial V5 includes Blood pressure, weight, (optional) blood draw (0.5 mL), urine collection, and additional phone calls every 6 months after the 2 year in person visit.

Clarification of SAEs, definitions, reporting and resolution of SAEs was added to V5:
1) Safety during the high dose treatment period. Serious adverse events (SAEs) specific to Epo will be defined as any of the following during the treatment period:

- Hypertension: subject required prolonged antihypertensive therapy (> 1 month) and/or will be discharged on medication
- Polycythemia (central hematocrit > 65%, or hematocrit increase ≥ 15% not due to red blood cell transfusion)
- Major venous or arterial thrombosis (involving a major vessel **not related to an infusion line,** requiring anticoagulation, or symptomatic such as causing superior vena cava syndrome)

Serious adverse events most likely related to prematurity will include

- Severe pulmonary hemorrhage (severe, e.g., need for increased respiratory support, positive pressure. *Not* pink tinged secretions with suctioning).
- Necrotizing enterocolitis (stage 2B or 3)
- Severe sepsis: Blood culture-proven bacterial or fungal sepsis requiring blood pressure support or significant new respiratory support.
- Intracranial hemorrhage (grade III or IV)
- ROP requiring intervention (surgery or Avastin).

ROP will be included as a severe adverse event, although due to the timing of screening, which begins at 31 weeks, it will most likely be too late to discontinue treatment. Each case of ROP will be reviewed in real time. Subjects will be screened for ROP as per the 2006 guidelines: for 24-27 week gestation infants, the first screening will occur at 31 weeks PMA.[132] The timing of follow-up ROP exams will be determined by findings on the initial exam.

Resolution of SAEs:

- NEC

-When infant is able to take full feeds, or what is considered equivalent to full feeding for that particular infant. If the child does not make it to full feedings due to short gut, note the method of feeding at the time of discharge (e.g. Home TPN)

- ROP
  -When an infant is diagnosed by an ophthalmologist exam with mature vessels
  -They no longer require frequent follow up ophthalmology appointments

- IVH
  -When shunt placement has occurred.
  -When sequential cranial ultrasounds show the ventricle size is stable or getting smaller.

- Hypertension
  -When hypertensive medications are discontinued
  -If infant is discharged on antihypertensive medications

- Sepsis
  -When antibiotics are completed.

- Thrombosis
  -When anticoagulation treatment ends.

- Pulmonary Hemorrhage
  -When no more blood in tracheal secretions and with stable vent settings.

Additional serious events which may be expected or unexpected include:

- Death
- Cardiac arrest
- Other unexpected life threatening event

Criteria for Temporarily Withholding/Stopping Study Drug and/or Supplemental Iron were clarified as follows:

1. **Polycythemia:** Central hematocrit (Hct) > 65%: Study drug should be held until Hct is $\leq$ 55%.
2. **Severe sepsis**: Blood culture-proven bacterial or fungal sepsis requiring blood pressure support or significant new respiratory support. Supplemental iron *should be held until blood culture is negative for 72 hours.* Rationale: Iron has been reported to be permissive for selected gram-negative bacteria and might worsen the patient's condition during sepsis. **There is no known relationship between Epo and sepsis**, so study drug does not need to be held.

3. **Unexplained recurrent seizure** (unrelated to ICH, PVL or other known pathology): Study drug should be held until seizures are well controlled by medication. Restarting study drug will be determined by the Medical Monitor, DSMB, and CCC.

4. Major **venous or arterial thrombosis (clot).**
   a. Study drug should be held for any thrombosis that is treated with a course of anticoagulation.
   b. Study drug should be held for any **symptomatic** thrombosis involving a major vessel (e.g. symptoms such as superior vena cava syndrome)

5. **Sustained hypertension *requiring medical intervention***: Study drug should be discontinued if blood pressure requires treatment. When blood pressure returns to normal range (systolic blood pressure < 100 mmHg), study drug can be resumed. This is true even if patient is still being treated, but blood pressure is being controlled.

Additional information regarding pharmacy procedures and iron protocols were added in V5

Information about adherence to protocol was added:

5.4 Adherence Assessment

All contact with families after discharge will be recorded in the Follow-Up CRFs. The follow-up phone calls at 4, 8, 12 and 18 months corrected age will assess medical health and functional condition and remind parents of need for neurodevelopmental assessment. Attendance at follow-up clinic for the in-person 2 year corrected age assessment will be documented.

Urine will be collected by placing cotton balls in the infant's diaper with a diaper change. (If needed, a bag can be used to collect urine instead of cotton balls.) At the next diaper change, these cotton balls will be placed in an empty 10 mL syringe and the plunger will be used to squeeze the urine out into collection tubes. The collection tubes will be labeled with the infant's study ID number, and date. The urine will then be frozen and sent in batches to the University of Washington for further processing and analysis.

NEJM Manuscript 19-07423.R2 – A Randomized trial of Erythropoietin: Neuroprotection for Preterm Infants

PENUT Trial Statistical Analysis Plan

We submit the original statistical analysis plan:

1. Statistical Analysis Plan Version 1.0 (PENUT-Trial_Statistical_Analysis_Plan_V1.0.doc)

And the final statistical analysis plan:

2. Statistical Analysis Plan Version 2.0 (PENUT-Trial_Statistical_Analysis_Plan_V2.0.doc)

Summary of changes from the original statistical analysis plan to the final statistical analysis plan:

1. Clarified wording in the second sentence under section 2.1 Primary Analysis to more clearly align with what was intended and done: *"Specifically, we will use a GEE Wald test based on logistic regression clustering on maternal ID to account for within-sibship correlation of outcomes, and adjusting for recruitment center and gestational age (24 to 25 6/7 weeks versus 26 to 27 6/7 weeks) as fixed effects."*
2. Added Section '2.1.1 Subgroup Analyses', which was described in the protocol but was omitted in the original statistical analysis plan.
3. Added Section '2.1.2 Sensitivity Analysis of Primary Outcome', which describes a sensitivity analysis where we included infants who died prior to the first study drug dose, NDI outcomes for subjects who were followed up out of window, and adjudicated consensus NDI outcomes for subjects missing the primary endpoint due to a partially completed follow-up exam. For remaining subjects that were alive but missing NDI, we performed multiple imputation to examine the sensitivity of the complete-case results to the results where all subjects are included.

PENUT TRIAL STATISTICAL ANALYSIS PLAN

1. General Design Considerations

Hypothesis: Epo treatment from 24 hours to 32-6/7 weeks postmenstrual age (PMA) of Extremely Low Gestational Age Neonates (ELGANs) will safely decrease the combined outcome of death or neurologic impairment from 40% to 30% measured at two years of age.

Design: We will enroll 940/2 = 470 subjects in each of two treatment groups: Epo-treated (from 24 hours of age to 32-6/7 weeks PMA) vs. placebo control.

Randomization sequences will be created centrally by the DCC. We will use block randomization within site using variable blocks of size 4, 6, 8 and 10 subjects. Using block randomization ensures that equal numbers of subjects are randomized to the intervention and control arm and that the two groups are balanced at period enrollment intervals. For multiple births (twins, triplets) all infants will be randomized to the same treatment group (e.g. effective randomization of the mother).

A modified intent-to-treat (mITT) approach will be used, with all randomized infants who receive the first dose of study treatment to be included in the analysis. All pre-specified hypotheses will be tested using a two-sided type I error of 0.05 with no formal adjustment for multiple comparisons unless otherwise specified (such as with safety outcomes). Secondary analyses that focus on separate hypotheses will not require correction for multiple comparisons, but those analyses that use multivariate measures such as multiple brain image parameters would be corrected for multiple comparisons using standard methods.

Given that we anticipate enrollment of multiple births we require that all analyses properly account for the within-sibship correlation of outcomes. We will use Generalized Estimating Equations (GEE), which is a versatile regression approach for the analysis of discrete and continuous outcomes. Use of "robust" standard errors will provide valid statistical inference and fully account for the clustering of data.

2. Primary Outcome

The primary outcome is the composite outcome of death or neurodevelopmental impairment at 22-26 months corrected age. Neurodevelopmental impairment (NDI) is defined as the presence of any one of the following: CP, Bayley III cognitive or motor scale < 70. There is a known inflation of scores from the Bayley II to III and we will therefore also consider a threshold of < 85 for secondary analysis. Subjects will be stratified by gestational age (24-0/7 to 25-6/7 and 26-0/7 to 27-6/7), number of babies in the pregnancy, and by study site. Table 1 shows how CP will be categorized based on features present on standardized neurologic exam.

Table 1. Motor deficit severity defined by CP and GMFCS function score

Motor Deficit	QP	Any CP with GMFCS 3-5	HP or DP with GMFCS 1-2	HP or DP with GMFCS 0	No CP but GMFCS 1-2	No CP and GMFCS 0
Severe	+	+				
Moderate			+		+	
Mild				+		
None						+

2.1. Primary Analysis: The primary analysis will be a test of equality of the rate of the primary outcome (death or NDI) across the two randomized investigational groups. Specifically, we will use a GEE Wald test based on logistic regression, with stratification by recruitment center, multiples in gestation and gestational age. We will perform intent to treat analysis and expect minimal non-compliance due to the nature of the intervention in relation to in-patient care. For the primary endpoint we expect uniform and complete ascertainment of death but may not evaluate all subjects for developmental impairment. We plan to perform a primary analysis based on complete cases and will exclude those subjects for whom vital status is known (alive) but NDI cannot be assessed. Secondary analysis will be for quantitative measures of brain volume, and for these endpoints an unadjusted t-test provides inference regarding the mean response across the treatment groups. We will adjust all secondary outcome analyses for recruitment site using regression methods.

2.2. Power and Sample Size for Primary Outcome: In order to determine the necessary sample size for efficacy evaluation, we need to formulate assumptions for the primary outcome rate in the treated and untreated groups.

The primary outcome measure is the rate of death or severe neurodevelopmental impairment (NDI). Using data from two sources, we can compute the expected rates of death or NDI for the neonates that we will enroll. The Vermont Oxford Network 2008 Follow-up Report[210] evaluated the disability status of infants born in 2008 only, and the combined 2004-2008 cohorts. Follow-up status was determined at age 18-24 months and information regarding death and NDI is provided for subgroups of children based on their gestational age. Therefore, we can use these data to forecast expected trial results for our eligible subjects (24-27 weeks gestational age).

The VON reports on severe NDI and specifically states that components of severe disability include: cerebral palsy, or a Bayley score less than 70 or too severely delayed for Bayley testing.[210]

In addition, data from Gargus et al. (2009) provides follow-up information at 18-22 months for approximately 3,800 neonates born between 24 and 27 weeks gestational age, based on babies born between 1998 and 2001.

We have combined the VON and Gargus (2009) data in order to determine the anticipated characteristics of our proposed trial. While the VON data is contemporary, it has relatively low follow-up for two-year outcomes (approximately 50%) and therefore may be biased toward more easily followed or more favorable subjects. Table 9 displays our best estimates of gestational age specific and overall rates of death and NDI.

Our assumed death rates of 32, 19, 13 and 9 percent are exactly those presented in the VON 2008 report, and are lower than the rates reported in Gargus (2009) where among neonates born between 1998 and 2001, GA-specific death rates of 45, 25, 18, and 15 percent were observed. Using our combined data assumptions, we expect an overall death rate of approximately 18%, which is approximately the rate observed in VON (17%), and is lower than the 26% death rate observed for Gargus (2009).

Table 2. Combined Vermont Oxford Network (VON) and Gargus (2009) data showing death rates and severe neurodevelopmental impairment (NDI) rates for different gestational ages (GA).

Gestational age (weeks)	Fraction of enrolled	Death	NDI	Total (Death+NDI)	Expected Treated (Death + NDI*0.45)
24	0.25	32	25.0	54.4	44.8
25	0.25	19	24.2	40.1	32.3
26	0.25	13	21.2	30.4	24.7
27	0.25	9	18.1	24.5	19.0
[illegible]		[illegible]	[illegible]	[illegible]	[illegible]

In addition, Gargus (2009) report GA-specific rates of NDI as 21, 25, 23, and 19 percent. The VON 2008 report estimates NDI rates as 22, 21, 17, and 16 percent respectively. We have combined VON and Gargus (2009) data and further assumed that NDI rates are decreasing with increasing gestational age to obtain our NDI estimates. Using these data we expect an overall NDI rate of 22%, which equals the overall rate observed in the VON 2008 report and in Gargus (2009).

In order to estimate the overall rate observed among treated neonates, we have assumed that there will be no effect of treatment on death, but that Epo will lead to a decrease in the rate of NDI. If we assume a multiplicative reduction in the NDI rate of 0.45 then we expect a treated NDI rate of 12 percent and an overall rate of death+NDI of 30.4% as compared to the control rate of 40.4%. Therefore, in order to obtain a target sample size we assume: an overall control rate of 40%, and an overall treated rate of 30% corresponding to an overall treatment rate ratio of 0.75.

Using the control and treated rates of 40% and 30% respectively leads to a sample size of 376 evaluated subjects per arm or a total evaluated sample size of 752 subjects.

3. Secondary Outcomes

Secondary Aims and Outcomes:

- To compare safety measures between infants receiving Epo and placebo to determine whether there are risks associated with Epo administration.
- To compare moderate impairment (< 85 on Bayley III cognitive or motor score, or CP).
- To compare neuroimaging outcomes across the two treatment groups.
- To assess whether treatment effects vary by gender.
- To evaluate whether individual biomarkers measured through 36 weeks are predictive of 2-year outcomes, and whether a derived multivariate combination of markers has predictive performance greater than individual marker performance.

3.1. Safety: The primary safety outcomes are the serious adverse events and adverse events listed in the CRFs. Analysis will compare the proportion of subjects with an AE across the two treatment groups and will use a 2-sample test of proportions. Inference for the (5) individual SAE outcomes will use a Bonferroni correction for multiple comparisons.

3.1.1. Power: The expected prevalence of SAEs range from less than 1% for polycythemia, to 18% for death. In order to characterize power we show the effect sizes for which we have 80% power based on having n=376 subjects evaluated in each group:

Table 3. Detectable differences in safety events.

Baseline (control) rate	Alternative rate for 80% power<br>Using alpha=0.05	Using alpha=0.01
5%	11% (RR = 2.2)	13% (RR = 2.6)
10%	18% (RR = 1.8)	19% (RR = 1.9)
15%	24% (RR = 1.6	25% (RR = 1.7)
20%	30% (RR = 1.5)	31% (RR = 1.5)

3.2. Secondary Efficacy Outcome: Our key secondary long-term outcome is the rate of death or NDI using severe or moderate impairment defined by a Bayley less than 85. In order to estimate power for the secondary outcome we assume the same overall rates for death and severe NDI as above, and then assume a 20% rate for moderate impairment. Similar to the primary outcome, we assume that the death rate is unchanged by Epo and that the rate of severe NDI is reduced from 22% to 12%. We also assume that the moderate NDI group would have an effect similar to the severe group where the 20% rate would be reduced by a factor of 0.45 to 11% but that among the 10% of severe subjects impacted by Epo, half of these would only move from severe to moderate impairment.

Table 4. Anticipated secondary efficacy outcome by group

	Control	Treated
Death	18%	18%
Severe NDI	22%	12%
Moderate NDI	20%	16% = 11% remain mod + 5% from severe

3.2.1. Power: Using these assumptions and the target evaluated of n=752 leads to greater than 95% power to detect a difference on the secondary outcome.

3.3. Imaging Outcomes: The neuroimaging outcomes are: Myelinated white matter volume; Total gray matter volume; and White matter integrity (TBSS FA corpus callosum). We will conduct a single MANOVA test using the multivariate outcome and comparing treated and control subjects. The mean and standard deviation will also be calculated (by treatment group) for each individual measure. Note that MR measures will be obtained on a subset of infants (110 per treatment group).

Given the *a priori* hypothesis that treatment effect may differ according to gender we will conduct a single subgroup analysis that assesses treatment effects separately for males and for females. Subgroup specific treatment effects will be computed and inference will be based on a single Gender-by-Treatment test for interaction using a logistic-based GEE regression model.

3.3.1. Power: Multiple MRI variables will be collected and analyzed in this study. To provide a basic sample size for a treatment effect we selected one common basic measure of structural injury that has been identified in the literature for the premature neonates that we hypothesize will be preserved: The size of the cerebellum tissue when compared to the whole head volume. We used a comparable set of T1 weighted brain scans of 22 premature babies aged between 34 and 36 PMA, with a representative range of brain injuries present on MRI and applied the proposed segmentation methods to the data. We estimated the ratio of the cerebellum to whole brain size as our marker across this group and used the mean and variance of these measures on which to base our power calculation for a treatment effect. Assuming age matched groups in the treated and untreated groups, from this data we estimate that 110 subjects will be required to see a 5% increase in the cerebellum size. The recognition of cerebellar atrophy in extremely preterm infants has only recently come to light with the increased use of MRI. Cerebellar atrophy has been linked to cognitive as well as motor deficits. In summary, we will focus on four primary measures that are established to be predictive of 2-year neurodevelopmental status and therefore can be used to establish support for the hypothesis of long-term benefit of Epo treatment. The neuroimaging biomarkers are: 1) myelinated white matter volume; 2) total gray matter volume; 3) cerebellar volume and 4) white matter integrity assessed by TBSS (using FA corpus callosum).

3.4. Biomarker Analysis: We will consider two main classes of potential predictors of 2-year status: neuroimaging measures and inflammatory markers. Interest is in the prognostic potential of individual and/or combined biomarker measurements. Given that the primary outcome is a binary measure (NDI), we will evaluate the predictive potential of individual quantitative measures using ROC curves showing the full potential of sensitivity and specificity across marker cut points. We will compute ROC curves for the (4) primary neuroimaging measures, and separately for individual inflammatory markers. Only 220 subjects will have data on the inflammatory markers, and these will be the same subjects identified for MR measures. We will derive two multivariate predictive models: using the inflammatory markers; and using the MR measures. We will use AIC and 10-fold cross-validation to develop and validate predictive models. A final multivariate model will combine markers from both MR and inflammatory measures, and 10-fold cross-validation will permit inference in the incremental value of adding markers in combination by comparing ROC curves and associated area under the ROC curve (AUC).

4. Data and Safety Monitoring

Per NIH Guidelines (1998), this Data and Safety Monitoring Plan (DSMP) defines the oversight and monitoring activities that will ensure and maintain both the safety of participants and the scientific integrity and validity of the trial data. The plan also describes the procedures for adverse event reporting and detailed guidelines for recommendations related to trial continuation.

PENUT is a multi-center randomized placebo-controlled phase III trial to determine whether recombinant human erythropoietin (Epo) will safely improve the long-term neurodevelopmental outcome of preterm infants 24-0/7 to 27-6/7 weeks of gestation. The PENUT study will randomize n=940 subjects to receive either Epo or placebo.

The DSMB will monitor the rates of these comorbidities at 6 month intervals, comparing the rates in treated and control infants. Expected rates will be based on the published literature. For example, in the Network trial, 93% had RDS, 46% PDA, 36% had any ICH with 16% severe, 11% NEC, 36% late-onset sepsis, and 12% ROP. In the ELGAN study, 21% died by 2 years of age, 24% of all children had an ICH, 12% had moderate/severe ventriculomegaly, and 24% had ultrasound lesions (echodense or lucent).

4.1. Safety Analysis: For each SAE we will tabulate the event rate by treatment group, and then compare rates using Fisher's exact test. The DCC will prepare monthly summaries of reported SAEs for review by the Medical Monitor, the CCC PI and NINDS.

4.2. Data and Safety Monitoring Board (DSMB)

Review of the PENUT trial's study performance and safety outcomes will be conducted by the Data and Safety Monitoring Board (DSMB) as required by the NIH for multi-site clinical investigations. The DSMB consists of 5 members with a designated chair and safety officer. Committee membership includes expertise in both biostatistics and bioethics, and should have clinical expertise appropriate for the intervention and target population. The DSMB is expected to meet two times (every 6 months) per year to review study performance and safety outcomes

in Open and Closed Reports, and to review study enrollment reports quarterly. Ad hoc sessions may be scheduled as required should a serious adverse event need to be reviewed by the group.

Aggregate safety and efficacy data and study performance monitoring data will be presented during the open sessions of DSMB meetings. Blinded safety and efficacy data will be presented by treatment arm during the closed sessions. Review by the DSMB provides assurances that the trial can continue without jeopardizing patient safety. The DSMB is also responsible for protecting the confidentiality of the trial data and for monitoring the quality of both the data and study implementation procedures.

The DCC will work closely with the CCC in developing and implementing a comprehensive system for safety monitoring. Safety monitoring includes the systematic review of safety data for trends that may impact patient safety. The processing, reviewing, and reporting of adverse events (AEs) and serious adverse events (SAEs) are also part of this process. The infants in this study are hospitalized at study entry, and the duration of their hospitalization is highly variable and not predictable at birth. It ranges from as short as three months to more than a year.

Data and Safety Monitoring Schedule: Our target enrollment is n=940 which is expected to accrue during the first 2.5 years of the trial. Therefore, we will enroll approximately 200 subjects every 6 months. Our planned DSMB safety analyses will occur every 6 months after trial initiation.

4.3. Monitoring Guidelines

Based on findings following review of study data by the DSMB, the Board may recommend: continuation of the trial, termination of the trial, or modifications to the protocol (e.g. adding new measurements for safety monitoring, discontinuing high risk subjects, extending the trial in time, increasing the trial sample). Decision guidelines in the PENUT trial are based on group differences in adverse event rates as explained below, and on whether SAE rates exceed pre-determined thresholds.

4.4. Performance Monitoring

Performance monitoring will be an ongoing activity performed by the study principal investigator and statistician, and status reports will be reviewed by the DSMB during their regular meetings. Procedural reviews to address protocol compliance with respect to subject recruitment and eligibility, retention and follow-up, randomization and blinding and quality of data will be conducted and monthly reports generated. Any protocol violation that affects patient safety will be reported to the DSMB immediately.

Performance data will be reviewed in aggregate and by site. It is expected that:

- The response rate for determination of the primary outcome (e.g. 24 month Bayley) will be no less than 80%;
- Missing interviews will be no greater than 15% at the 4, 8, 12 and 24 month follow-ups;
- The overall enrollment rate will not drop below the expected rate (34 subjects per month for 24 months) by more than 25%.

Data will be entered into a central trial management system utilizing a REDCap database hosted at the DCC. Data will be entered into fields with automated validation and logical check built in. Data will be double entered on the outcome measures of Bayley III and CP assessment by standardized neurologic exam. Compliance will be assessed based on the weekly conference calls and data submitted to the DCC on a weekly basis. If it is determined by the study PI that either 1) study protocol is not being followed, or 2) that reporting is inadequate at any site, further action will be taken to address these issues. These actions may include additional in-person site visits if appropriate or additional educational/problem-solving sessions by phone or in person regarding the study protocol.

4.5. Safety Monitoring:

The research coordinator at each site will monitor each subject daily for the presence of any complications until discharge. Serious adverse events will be brought to the attention of the CCC PI who will report them to the Medical Monitor DSMB and NINDS. Sites will notify their local IRBs of SAEs per site institutional requirements. An independent Medical Monitor will review all cases of serious adverse events. A potential risk that is unique to preterm infants is the risk of ROP. In the published studies of preterm neonates receiving potentially neuroprotective doses of Epo, no difference has been noted between treatment and control groups.

As part of the Data and Safety Monitoring Plan (DSMP) we will perform continuous and interim analysis of accruing safety data. We have defined potentially treatment (Epo) related serious adverse events (SAEs) that will be monitored throughout the course of the study. Specifically, for SAEs, we will compare absolute rates to expected rates based on published data for similar newborns, and will seek careful DSMB review and guidance when observed rates exceed pre-specified thresholds. In addition, at planned interim analysis we will formally compare the event rates across the two treatment groups using appropriate small sample methods such as Fisher's exact test.

4.6. Treatment Efficacy Monitoring

Treatment efficacy will be monitored by the DSMB, but recognizing that 24-month efficacy outcomes will not be obtained until after enrollment is complete (or nearly complete). Therefore, although formal interim analyses will be conducted using group-sequential boundaries there may not be compelling reasons to terminate the trial on the basis of accruing efficacy data. Efficacy measures will be provided in a blinded fashion to the DSMB with treatment groups labeled "A" and "B" as well as a z-score assessment of the evidence for the difference between treatment arms at 24 months. An O'Brien-Fleming interim stopping boundary will be provided to the DSMB to help assess the magnitude of the z-score.

4.7. Scheduled Reporting

One month prior to each DSMB review, the Data Coordinating Center (DCC) will summarize monthly administrative reports that describe study progress including subject accrual by site, demographics, and the sites' adherence to inclusion/exclusion criteria and other protocol requirements, including retention rates at each follow-up point. These reports are prepared monthly and reviewed internally by the study research team for ongoing quality control and are

also presented to the DSMB and NIH as requested. The DCC will also produce safety reports that list adverse events, serious adverse events, deaths, and disease or treatment specific events by site and in aggregate to the DSMB.

With each review, the DSMB will approve the study and protocol as is, recommend protocol changes in the interest of patient safety or stop the study based on overwhelming evidence of treatment benefit or safety concerns. The DSMB will provide the recommendation in written minutes provided to the NIH. If the NIH concurs with these recommendations, they will be forwarded to the principal investigator. The recommendations will include any suggested changes in the proposed timing of future DSMB reviews. The review may result in an amendment to the protocol, which must be approved by the IRB, the NIH and the sponsor.

The DSMB report will begin with a brief narrative section that describes the status of the study, progress or findings to-date, issues, and the procedures that produced the report (e.g., data obtained by a specific date). The report will provide a study description along with a current organization chart, current timetable and study schedule as well as a list of study clinical and administrative centers. Data will be presented that describe the administrative status of the study including recruitment and forms handling. Study data reports describe demographic and baseline clinical characteristics and provide a safety assessment. Tables will be provided by site as well as for the whole study population. AE/SAE rates for each group will be presented.

Following each DSMB meeting, the NINDS will send the study's principal investigator a letter confirming that the DSMB met, reviewed all accumulated study data, and made a recommendation that the study continue as planned (or, possibly, to modify the protocol). The principal investigator will forward a copy of this letter to each of the clinical investigators who, in turn, are responsible for forwarding it to their local IRB.

4.8. Serious Adverse Event Reporting and Monitoring
We divided potential serious adverse events (SAEs) by the severity. In Table 5, we detail the defined SAEs with associated expected event rates and thresholds that would trigger DSMB review. Most of the SAEs would occur during the Epo treatment period or during hospitalization and therefore would be immediately recorded.

Table 5. Serious Adverse Events

Serious Adverse Event (SAE) Potentially Epo related	Expected Rate	Threshold
Hypertension	20%	25%
Polycythemia (hematocrit > 65%)	Rare < 1%	2%
Major venous or arterial thrombosis (clot) not associated with a central line	Rare < 5%	10%
Other unexpected life threatening event	Rare < 5%	10%
**Serious Adverse Event (SAE) Prematurity related**	**Expected Rate**	**Threshold**
Pulmonary Hemorrhage (Severe)	7%	15%
NEC (Stage 2b or 3)	12%	25%
Sepsis (severe)	33%	50%
Intracranial hemorrhage (grade III or IV)	16%	25%
Retinopathy of Prematurity (Severe)	7%	14%
**Other Serious Adverse Event: Expected or unexpected**		
Death	Approx. 18%	30%
Cardiac arrest	Rare < 1%	2%
Other life threatening event	Rare < 1%	2%

In addition to the monitoring of SAEs, we will also measure and compare rates of adverse events (AEs) across the two treatment arms (Table 6). Given the high-risk study population a number of adverse events are expected, and the key monitoring function will be to assess whether evidence is accruing that suggests a differential adverse event rate associated with treatment.

Table 6. Adverse Events

Adverse Event (AE)	Expected Rate
Respiratory distress syndrome (RDS)	93%
Patent ductus arteriosus (PDA)	46%
Bronchopulmonary dysplasia (BPD)	42%
Central line-related thrombosis (clot)	40%
Intracranial hemorrhage (ICH)	36% total<br>16% (grade III & IV)
Necrotizing enterocolitis (NEC)	11%
Periventricular leukomalacia (PVL)	4%
Hydrocephalus	2%
Clinical Seizures	6%-13%
ROP (stages 1-2)	59%

Estimates here are based on three primary sources: Stoll et al. (2010); O'Shea et al. (2009) ELGAN study; and The Vermont Oxford Network (VON) 2008 Follow-up Report (2011). Safety evaluation will be based on all available follow-up but we expect the majority of SAEs and AEs to occur during hospitalization and therefore within the first three months study follow-up. SAE's and AE's will be considered by gestational age category, given that complication rates of infants 24 and 25 weeks of gestation are expected to be higher than those at 26 and 27 weeks of gestation.

Figure 8: Probability (power) of exceeding established thresholds for safety outcomes. Here we consider an expected event rate of 5% with a threshold of 10% used to trigger DSMB action.

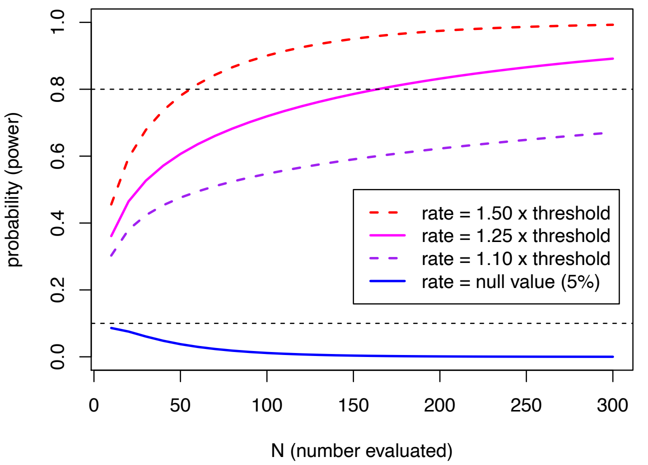

SAEs and Threshold for Action: For each SAE listed in Table 4 we have established expected rates of SAEs for the target study population, and thresholds for each treatment arm at which careful evaluation would be required by DSMB review. The DSMB would be asked to review all information associated with the line listing for the SAEs and then make a decision as to whether the study should be modified, continued or stopped. In Figure 8 we show the operating characteristics for an SAE that has an expected (normal) rate of 5%, and a threshold of 10% for review. If the true SAE rate is 1.5 times the threshold (e.g. 15%) then with 100 or more subjects there is a greater than 80% probability (power) that the observed rate of greater than the threshold would occur and trigger DSMB review. However, if the true SAE rate was only 1.25 times greater than the threshold then 150 or more subjects would be needed to have 80% power. Although a total of 10 SAEs are monitored for their absolute rate, we will not use any multiple comparison correction for continuous monitoring of safety leading to DSMB review. However, formal interim analysis comparing safety across the two study groups will account for both multiple comparisons due to multiple individual safety outcomes, and for multiple interim analyses.

SAE analysis uses a doubling of rates to trigger consideration of DSMB action irrespective of statistical significance at interim analysis, yet formal monitoring will be conducted every 6 months to evaluate the strength of statistical evidence for a difference across groups. Therefore, our proposed interim analyses will evaluate the accruing data using both formal statistical comparison in addition to consideration of absolute rates and is thus more conservative than use of only one criterion. Finally, we plan to conduct interim analyses that will also report on the specific SAEs and AEs listed in Tables 4 and 5, and will provide p-values comparing treatment groups for these safety outcomes in addition to the SAE outcomes. A medical safety officer not involved in the study will review all SAEs, and the DSMB (appointed by NINDS) will decide when they want to review each type of SAE or AE.

6.9 Formal Interim Analyses: Our primary objective for interim analysis is to allow careful and continuing analysis of safety outcomes. Specifically, we propose to conduct formal statistical analysis and inference for each SAE and AE at three interim and one final analysis time. We will continue to analyze all AE events that have occurred during follow-up, but focus interim analysis on those events that occur within the first three months of follow-up since this is the time period in which the major treatment related events would be expected. We will conduct formal interim safety monitoring analysis at approximately 25% (n=235), 50% (n=470), and 75% (n=705) and 100% of target enrollment of n=940. We plan to monitor death and will control the overall significance level using O'Brien-Fleming boundaries (net alpha=0.05 significance, accounting for three interim and one final analysis).[219] For the other 4 SAEs, we will again use sequential monitoring boundaries to control the outcome-specific family-wise error rate (using a Bonferroni corrected alpha of 0.05/5 = 0.01).

The DSMP specifies formal interim safety monitoring analysis at approximately 25% (n=235), 50% (n=470), and 75% (n=705) and 100% of target enrollment of n=940. For death, we will use O'Brien-Fleming sequential monitoring guidelines with a net alpha=0.05, and a Bonferroni corrected sequential guideline for the 10 SAE for a significance level of 0.05/10 = 0.005 to correct for multiple events. The O'Brien-Fleming sequential monitoring p-values are listed below in Table 7.

Table 7. O'Brien-Fleming p-values for interim monitoring of death and SAEs.

Enrollment	P-value for monitoring death	P-value for monitoring all other SAEs
25% (n=235)	0.000014734	0.000000000295
50% (n=470)	0.0030359	0.000038113
75% (n=705)	0.016248	0.00092412
100% (n=940)	0.030701	0.0040378

The CCC PI will be responsible for notifying the Medical Monitor of any safety events, and the Medical Monitor will notify the DSMB if indicated. All events will be reported to the DSMB in semi-annual reports.

PENUT TRIAL STATISTICAL ANALYSIS PLAN

1. General Design Considerations

Hypothesis: Epo treatment from 24 hours to 32-6/7 weeks postmenstrual age (PMA) of Extremely Low Gestational Age Neonates (ELGANs) will safely decrease the combined outcome of death or neurologic impairment from 40% to 30% measured at two years of age.

Design: We will enroll 940/2 = 470 subjects in each of two treatment groups: Epo-treated (from 24 hours of age to 32-6/7 weeks PMA) vs. placebo control.

Randomization sequences will be created centrally by the DCC. We will use block randomization within site using variable blocks of size 4, 6, 8 and 10 subjects. Using block randomization ensures that equal numbers of subjects are randomized to the intervention and control arm and that the two groups are balanced at period enrollment intervals. For multiple births (twins, triplets) all infants will be randomized to the same treatment group (e.g. effective randomization of the mother).

A modified intent-to-treat (mITT) approach will be used, with all randomized infants who receive the first dose of study treatment to be included in the analysis. All pre-specified hypotheses will be tested using a two-sided type I error of 0.05 with no formal adjustment for multiple comparisons unless otherwise specified (such as with safety outcomes). Secondary analyses that focus on separate hypotheses will not require correction for multiple comparisons, but those analyses that use multivariate measures such as multiple brain image parameters would be corrected for multiple comparisons using standard methods.

Given that we anticipate enrollment of multiple births we require that all analyses properly account for the within-sibship correlation of outcomes. We will use Generalized Estimating Equations (GEE), which is a versatile regression approach for the analysis of discrete and continuous outcomes. Use of “robust” standard errors will provide valid statistical inference and fully account for the clustering of data.

2. Primary Outcome

The primary outcome is the composite outcome of death or neurodevelopmental impairment at 22-26 months corrected age. Neurodevelopmental impairment (NDI) is defined as the presence of any one of the following: CP, Bayley III cognitive or motor scale < 70. There is a known inflation of scores from the Bayley II to III and we will therefore also consider a threshold of < 85 for secondary analysis. Subjects will be stratified by gestational age (24-0/7 to 25-6/7 and 26-0/7 to 27-6/7), number of babies in the pregnancy, and by study site. Table 1 shows how CP will be categorized based on features present on standardized neurologic exam.

Table 1. Motor deficit severity defined by CP and GMFCS function score

Motor Deficit	QP	Any CP with GMFCS 3-5	HP or DP with GMFCS 1-2	HP or DP with GMFCS 0	No CP but GMFCS 1-2	No CP and GMFCS 0
Severe	+	+				
Moderate			+		+	
Mild				+		
None						+

2.1. Primary Analysis: The primary analysis will be a test of equality of the rate of the primary outcome (death or NDI) across the two randomized investigational groups. Specifically, we will use a GEE Wald test based on logistic regression clustering on maternal ID to account for within-sibship correlation of outcomes, and adjusting for recruitment center and gestational age (24 to 25 6/7 weeks versus 26 to 27 6/7 weeks) as fixed effects. We will perform intent to treat analysis and expect minimal non-compliance due to the nature of the intervention in relation to in-patient care. For the primary endpoint we expect uniform and complete ascertainment of death but may not evaluate all subjects for developmental impairment. We plan to perform a primary analysis based on complete cases and will exclude those subjects for whom vital status is known (alive) but NDI cannot be assessed. Secondary analysis will be for quantitative measures of brain volume, and for these endpoints an unadjusted t-test provides inference regarding the mean response across the treatment groups. We will adjust all secondary outcome analyses for recruitment site using regression methods.

2.1.1 Subgroup Analyses: Sex and gestational age (24 to 25 6/7 weeks versus 26 to 27 6/7 weeks) were pre-specified subgroups, where treatment effect heterogeneity on the complete-case primary outcome was evaluated separately for each variable through a Wald test of the statistical interaction terms with treatment. Confidence intervals (CI) are reported for the treatment effect within each subgroup.

2.1.2 Sensitivity Analysis of Primary Outcome: The primary analysis was based on complete cases and excluded those subjects known to be alive but not assessed for NDI. In a sensitivity analysis, we included infants who died prior to the first study drug dose, NDI outcomes for subjects who were followed up out of window, and adjudicated consensus NDI outcomes for subjects missing the primary endpoint due to a partially completed follow-up exam. For remaining subjects with missing NDI outcomes, we conducted multiple imputation (`mice` R package; m=10 imputations) to impute NDI using baseline and phone follow-up variables associated with missingness or NDI.

2.2. Power and Sample Size for Primary Outcome: In order to determine the necessary sample size for efficacy evaluation, we need to formulate assumptions for the primary outcome rate in the treated and untreated groups.

The primary outcome measure is the rate of death or severe neurodevelopmental impairment (NDI). Using data from two sources, we can compute the expected rates of death or NDI for the neonates that we will enroll. The Vermont Oxford Network 2008 Follow-up Report[210] evaluated the disability status of infants born in 2008 only, and the combined 2004-2008 cohorts. Follow-up status was determined at age 18-24 months and information regarding death and NDI is provided for subgroups of children based on their gestational age. Therefore, we can use these data to forecast expected trial results for our eligible subjects (24-27 weeks gestational age).

The VON reports on severe NDI and specifically states that components of severe disability include: cerebral palsy, or a Bayley score less than 70 or too severely delayed for Bayley testing.[210]

In addition, data from Gargus et al. (2009) provides follow-up information at 18-22 months for approximately 3,800 neonates born between 24 and 27 weeks gestational age, based on babies born between 1998 and 2001.

We have combined the VON and Gargus (2009) data in order to determine the anticipated characteristics of our proposed trial. While the VON data is contemporary, it has relatively low follow-up for two-year outcomes (approximately 50%) and therefore may be biased toward more easily followed or more favorable subjects. Table 9 displays our best estimates of gestational age specific and overall rates of death and NDI.

Our assumed death rates of 32, 19, 13 and 9 percent are exactly those presented in the VON 2008 report, and are lower than the rates reported in Gargus (2009) where among neonates born between 1998 and 2001, GA-specific death rates of 45, 25, 18, and 15 percent were observed. Using our combined data assumptions, we expect an overall death rate of approximately 18%, which is approximately the rate observed in VON (17%), and is lower than the 26% death rate

Table 2. Combined Vermont Oxford Network (VON) and Gargus (2009) data showing death rates and severe neurodevelopmental impairment (NDI) rates for different gestational ages (GA).

Gestational age (weeks)	Fraction of enrolled	Death	NDI	Total (Death+NDI)	Expected Treated (Death + NDI*0.45)
24	0.25	32	25.0	54.4	44.8
25	0.25	19	24.2	40.1	32.3
26	0.25	13	21.2	30.4	24.7
27	0.25	9	18.1	24.5	19.0
**[illegible]**		**[illegible]**	**[illegible]**	**[illegible]**	**[illegible]**

observed for Gargus (2009).

In addition, Gargus (2009) report GA-specific rates of NDI as 21, 25, 23, and 19 percent. The VON 2008 report estimates NDI rates as 22, 21, 17, and 16 percent respectively. We have combined

VON and Gargus (2009) data and further assumed that NDI rates are decreasing with increasing gestational age to obtain our NDI estimates. Using these data we expect an overall NDI rate of 22%, which equals the overall rate observed in the VON 2008 report and in Gargus (2009).

In order to estimate the overall rate observed among treated neonates, we have assumed that there will be no effect of treatment on death, but that Epo will lead to a decrease in the rate of NDI. If we assume a multiplicative reduction in the NDI rate of 0.45 then we expect a treated NDI rate of 12 percent and an overall rate of death+NDI of 30.4% as compared to the control rate of 40.4%. Therefore, in order to obtain a target sample size we assume: an overall control rate of 40%, and an overall treated rate of 30% corresponding to an overall treatment rate ratio of 0.75.

Using the control and treated rates of 40% and 30% respectively leads to a sample size of 376 evaluated subjects per arm or a total evaluated sample size of 752 subjects.

3. Secondary Outcomes

Secondary Aims and Outcomes:

- To compare safety measures between infants receiving Epo and placebo to determine whether there are risks associated with Epo administration.
- To compare moderate impairment (< 85 on Bayley III cognitive or motor score, or CP).
- To compare neuroimaging outcomes across the two treatment groups.
- To assess whether treatment effects vary by gender.
- To evaluate whether individual biomarkers measured through 36 weeks are predictive of 2-year outcomes, and whether a derived multivariate combination of markers has predictive performance greater than individual marker performance.

3.1. Safety: The primary safety outcomes are the serious adverse events and adverse events listed in the CRFs. Analysis will compare the proportion of subjects with an AE across the two treatment groups and will use a 2-sample test of proportions. Inference for the (5) individual SAE outcomes will use a Bonferroni correction for multiple comparisons.

3.1.1. Power: The expected prevalence of SAEs range from less than 1% for polycythemia, to 18% for death. In order to characterize power we show the effect sizes for which we have 80% power based on having n=376 subjects evaluated in each group:

Table 3. Detectable differences in safety events.

Baseline (control) rate	Alternative rate for 80% power<br>Using alpha=0.05	Using alpha=0.01
5%	11% (RR = 2.2)	13% (RR = 2.6)
10%	18% (RR = 1.8)	19% (RR = 1.9)
15%	24% (RR = 1.6	25% (RR = 1.7)
20%	30% (RR = 1.5)	31% (RR = 1.5)

3.2. Secondary Efficacy Outcome: Our key secondary long-term outcome is the rate of death or NDI using severe or moderate impairment defined by a Bayley less than 85. In order to estimate power for the secondary outcome we assume the same overall rates for death and severe NDI as above, and then assume a 20% rate for moderate impairment. Similar to the primary outcome, we assume that the death rate is unchanged by Epo and that the rate of severe NDI is reduced from 22% to 12%. We also assume that the moderate NDI group would have an effect similar to the severe group where the 20% rate would be reduced by a factor of 0.45 to 11% but that among the 10% of severe subjects impacted by Epo, half of these would only move from severe to moderate impairment.

Table 4. Anticipated secondary efficacy outcome by group

	**Control**	**Treated**
Death	18%	18%
Severe NDI	22%	12%
Moderate NDI	20%	16% = 11% remain mod + 5% from severe
[illegible]	[illegible]	[illegible]

3.2.1. Power: Using these assumptions and the target evaluated of n=752 leads to greater than 95% power to detect a difference on the secondary outcome.

3.3. Imaging Outcomes: The neuroimaging outcomes are: Myelinated white matter volume; Total gray matter volume; and White matter integrity (TBSS FA corpus callosum). We will conduct a single MANOVA test using the multivariate outcome and comparing treated and control subjects. The mean and standard deviation will also be calculated (by treatment group) for each individual measure. Note that MR measures will be obtained on a subset of infants (110 per treatment group).

Given the *a priori* hypothesis that treatment effect may differ according to gender we will conduct a single subgroup analysis that assesses treatment effects separately for males and for females. Subgroup specific treatment effects will be computed and inference will be based on a single Gender-by-Treatment test for interaction using a logistic-based GEE regression model.

3.3.1. Power: Multiple MRI variables will be collected and analyzed in this study. To provide a basic sample size for a treatment effect we selected one common basic measure of structural injury that has been identified in the literature for the premature neonates that we hypothesize will be preserved: The size of the cerebellum tissue when compared to the whole head volume. We used a comparable set of T1 weighted brain scans of 22 premature babies aged between 34 and 36 PMA, with a representative range of brain injuries present on MRI and applied the proposed segmentation methods to the data. We estimated the ratio of the cerebellum to whole brain size as our marker across this group and used the mean and variance of these measures on which to base our power calculation for a treatment effect. Assuming age matched groups in the treated and untreated groups, from this data we estimate that 110 subjects will be required to

see a 5% increase in the cerebellum size. The recognition of cerebellar atrophy in extremely preterm infants has only recently come to light with the increased use of MRI. Cerebellar atrophy has been linked to cognitive as well as motor deficits. In summary, we will focus on four primary measures that are established to be predictive of 2-year neurodevelopmental status and therefore can be used to establish support for the hypothesis of long-term benefit of Epo treatment. The neuroimaging biomarkers are: 1) myelinated white matter volume; 2) total gray matter volume; 3) cerebellar volume and 4) white matter integrity assessed by TBSS (using FA corpus callosum).

3.4. Biomarker Analysis: We will consider two main classes of potential predictors of 2-year status: neuroimaging measures and inflammatory markers. Interest is in the prognostic potential of individual and/or combined biomarker measurements. Given that the primary outcome is a binary measure (NDI), we will evaluate the predictive potential of individual quantitative measures using ROC curves showing the full potential of sensitivity and specificity across marker cut points. We will compute ROC curves for the (4) primary neuroimaging measures, and separately for individual inflammatory markers. Only 220 subjects will have data on the inflammatory markers, and these will be the same subjects identified for MR measures. We will derive two multivariate predictive models: using the inflammatory markers; and using the MR measures. We will use AIC and 10-fold cross-validation to develop and validate predictive models. A final multivariate model will combine markers from both MR and inflammatory measures, and 10-fold cross-validation will permit inference in the incremental value of adding markers in combination by comparing ROC curves and associated area under the ROC curve (AUC).

4. Data and Safety Monitoring

Per NIH Guidelines (1998), this Data and Safety Monitoring Plan (DSMP) defines the oversight and monitoring activities that will ensure and maintain both the safety of participants and the scientific integrity and validity of the trial data. The plan also describes the procedures for adverse event reporting and detailed guidelines for recommendations related to trial continuation.

PENUT is a multi-center randomized placebo-controlled phase III trial to determine whether recombinant human erythropoietin (Epo) will safely improve the long-term neurodevelopmental outcome of preterm infants 24-0/7 to 27-6/7 weeks of gestation. The PENUT study will randomize n=940 subjects to receive either Epo or placebo.

The DSMB will monitor the rates of these comorbidities at 6 month intervals, comparing the rates in treated and control infants. Expected rates will be based on the published literature. For example, in the Network trial, 93% had RDS, 46% PDA, 36% had any ICH with 16% severe, 11% NEC, 36% late-onset sepsis, and 12% ROP. In the ELGAN study, 21% died by 2 years of age, 24% of all children had an ICH, 12% had moderate/severe ventriculomegaly, and 24% had ultrasound lesions (echodense or lucent).

4.1. Safety Analysis: For each SAE we will tabulate the event rate by treatment group, and then compare rates using Fisher’s exact test. The DCC will prepare monthly summaries of reported SAEs for review by the Medical Monitor, the CCC PI and NINDS.

4.2. Data and Safety Monitoring Board (DSMB)
Review of the PENUT trial's study performance and safety outcomes will be conducted by the Data and Safety Monitoring Board (DSMB) as required by the NIH for multi-site clinical investigations. The DSMB consists of 5 members with a designated chair and safety officer. Committee membership includes expertise in both biostatistics and bioethics, and should have clinical expertise appropriate for the intervention and target population. The DSMB is expected to meet two times (every 6 months) per year to review study performance and safety outcomes in Open and Closed Reports, and to review study enrollment reports quarterly. Ad hoc sessions may be scheduled as required should a serious adverse event need to be reviewed by the group.

Aggregate safety and efficacy data and study performance monitoring data will be presented during the open sessions of DSMB meetings. Blinded safety and efficacy data will be presented by treatment arm during the closed sessions. Review by the DSMB provides assurances that the trial can continue without jeopardizing patient safety. The DSMB is also responsible for protecting the confidentiality of the trial data and for monitoring the quality of both the data and study implementation procedures.

The DCC will work closely with the CCC in developing and implementing a comprehensive system for safety monitoring. Safety monitoring includes the systematic review of safety data for trends that may impact patient safety. The processing, reviewing, and reporting of adverse events (AEs) and serious adverse events (SAEs) are also part of this process. The infants in this study are hospitalized at study entry, and the duration of their hospitalization is highly variable and not predictable at birth. It ranges from as short as three months to more than a year.

Data and Safety Monitoring Schedule: Our target enrollment is n=940 which is expected to accrue during the first 2.5 years of the trial. Therefore, we will enroll approximately 200 subjects every 6 months. Our planned DSMB safety analyses will occur every 6 months after trial initiation.

4.3. Monitoring Guidelines
Based on findings following review of study data by the DSMB, the Board may recommend: continuation of the trial, termination of the trial, or modifications to the protocol (e.g. adding new measurements for safety monitoring, discontinuing high risk subjects, extending the trial in time, increasing the trial sample). Decision guidelines in the PENUT trial are based on group differences in adverse event rates as explained below, and on whether SAE rates exceed pre-determined thresholds.

4.4. Performance Monitoring
Performance monitoring will be an ongoing activity performed by the study principal investigator and statistician, and status reports will be reviewed by the DSMB during their regular meetings. Procedural reviews to address protocol compliance with respect to subject recruitment and eligibility, retention and follow-up, randomization and blinding and quality of data will be conducted and monthly reports generated. Any protocol violation that affects patient safety will be reported to the DSMB immediately.

Performance data will be reviewed in aggregate and by site. It is expected that:

- The response rate for determination of the primary outcome (e.g. 24 month Bayley) will be no less than 80%;
- Missing interviews will be no greater than 15% at the 4, 8, 12 and 24 month follow-ups;
- The overall enrollment rate will not drop below the expected rate (34 subjects per month for 24 months) by more than 25%.

Data will be entered into a central trial management system utilizing a REDCap database hosted at the DCC. Data will be entered into fields with automated validation and logical check built in. Data will be double entered on the outcome measures of Bayley III and CP assessment by standardized neurologic exam. Compliance will be assessed based on the weekly conference calls and data submitted to the DCC on a weekly basis. If it is determined by the study PI that either 1) study protocol is not being followed, or 2) that reporting is inadequate at any site, further action will be taken to address these issues. These actions may include additional in-person site visits if appropriate or additional educational/problem-solving sessions by phone or in person regarding the study protocol.

4.5. Safety Monitoring:

The research coordinator at each site will monitor each subject daily for the presence of any complications until discharge. Serious adverse events will be brought to the attention of the CCC PI who will report them to the Medical Monitor DSMB and NINDS. Sites will notify their local IRBs of SAEs per site institutional requirements. An independent Medical Monitor will review all cases of serious adverse events. A potential risk that is unique to preterm infants is the risk of ROP. In the published studies of preterm neonates receiving potentially neuroprotective doses of Epo, no difference has been noted between treatment and control groups.

As part of the Data and Safety Monitoring Plan (DSMP) we will perform continuous and interim analysis of accruing safety data. We have defined potentially treatment (Epo) related serious adverse events (SAEs) that will be monitored throughout the course of the study. Specifically, for SAEs, we will compare absolute rates to expected rates based on published data for similar newborns, and will seek careful DSMB review and guidance when observed rates exceed pre-specified thresholds. In addition, at planned interim analysis we will formally compare the event rates across the two treatment groups using appropriate small sample methods such as Fisher's exact test.

4.6. Treatment Efficacy Monitoring

Treatment efficacy will be monitored by the DSMB, but recognizing that 24-month efficacy outcomes will not be obtained until after enrollment is complete (or nearly complete). Therefore, although formal interim analyses will be conducted using group-sequential boundaries there may not be compelling reasons to terminate the trial on the basis of accruing efficacy data. Efficacy measures will be provided in a blinded fashion to the DSMB with treatment groups labeled "A" and "B" as well as a z-score assessment of the evidence for the difference

between treatment arms at 24 months. An O'Brien-Fleming interim stopping boundary will be provided to the DSMB to help assess the magnitude of the z-score.

4.7. Scheduled Reporting

One month prior to each DSMB review, the Data Coordinating Center (DCC) will summarize monthly administrative reports that describe study progress including subject accrual by site, demographics, and the sites' adherence to inclusion/exclusion criteria and other protocol requirements, including retention rates at each follow-up point. These reports are prepared monthly and reviewed internally by the study research team for ongoing quality control and are also presented to the DSMB and NIH as requested. The DCC will also produce safety reports that list adverse events, serious adverse events, deaths, and disease or treatment specific events by site and in aggregate to the DSMB.

With each review, the DSMB will approve the study and protocol as is, recommend protocol changes in the interest of patient safety or stop the study based on overwhelming evidence of treatment benefit or safety concerns. The DSMB will provide the recommendation in written minutes provided to the NIH. If the NIH concurs with these recommendations, they will be forwarded to the principal investigator. The recommendations will include any suggested changes in the proposed timing of future DSMB reviews. The review may result in an amendment to the protocol, which must be approved by the IRB, the NIH and the sponsor.

The DSMB report will begin with a brief narrative section that describes the status of the study, progress or findings to-date, issues, and the procedures that produced the report (e.g., data obtained by a specific date). The report will provide a study description along with a current organization chart, current timetable and study schedule as well as a list of study clinical and administrative centers. Data will be presented that describe the administrative status of the study including recruitment and forms handling. Study data reports describe demographic and baseline clinical characteristics and provide a safety assessment. Tables will be provided by site as well as for the whole study population. AE/SAE rates for each group will be presented.

Following each DSMB meeting, the NINDS will send the study's principal investigator a letter confirming that the DSMB met, reviewed all accumulated study data, and made a recommendation that the study continue as planned (or, possibly, to modify the protocol). The principal investigator will forward a copy of this letter to each of the clinical investigators who, in turn, are responsible for forwarding it to their local IRB.

4.8. Serious Adverse Event Reporting and Monitoring

We divided potential serious adverse events (SAEs) by the severity. In Table 5, we detail the defined SAEs with associated expected event rates and thresholds that would trigger DSMB review. Most of the SAEs would occur during the Epo treatment period or during hospitalization and therefore would be immediately recorded.

Table 5. Serious Adverse Events

Serious Adverse Event (SAE) Potentially Epo related	Expected Rate	Threshold
Hypertension	20%	25%
Polycythemia (hematocrit > 65%)	Rare < 1%	2%
Major venous or arterial thrombosis (clot) not associated with a central line	Rare < 5%	10%
Other unexpected life threatening event	Rare < 5%	10%
**Serious Adverse Event (SAE) Prematurity related**	**Expected Rate**	**Threshold**
Pulmonary Hemorrhage (Severe)	7%	15%
NEC (Stage 2b or 3)	12%	25%
Sepsis (severe)	33%	50%
Intracranial hemorrhage (grade III or IV)	16%	25%
Retinopathy of Prematurity (Severe)	7%	14%
**Other Serious Adverse Event: Expected or unexpected**		
Death	Approx. 18%	30%
Cardiac arrest	Rare < 1%	2%
Other life threatening event	Rare < 1%	2%

In addition to the monitoring of SAEs, we will also measure and compare rates of adverse events (AEs) across the two treatment arms (Table 6). Given the high-risk study population a number of adverse events are expected, and the key monitoring function will be to assess whether evidence is accruing that suggests a differential adverse event rate associated with treatment.

Table 6. Adverse Events

Adverse Event (AE)	Expected Rate
Respiratory distress syndrome (RDS)	93%
Patent ductus arteriosus (PDA)	46%
Bronchopulmonary dysplasia (BPD)	42%
Central line-related thrombosis (clot)	40%
Intracranial hemorrhage (ICH)	36% total<br>16% (grade III & IV)
Necrotizing enterocolitis (NEC)	11%
Periventricular leukomalacia (PVL)	4%
Hydrocephalus	2%
Clinical Seizures	6%-13%
ROP (stages 1-2)	59%

Estimates here are based on three primary sources: Stoll et al. (2010); O'Shea et al. (2009) ELGAN study; and The Vermont Oxford Network (VON) 2008 Follow-up Report (2011). Safety evaluation will be based on all available follow-up but we expect the majority of SAEs and AEs to occur during hospitalization and therefore within the first three months study follow-up. SAE's and AE's will be considered by gestational age category, given that complication rates of infants 24 and 25 weeks of gestation are expected to be higher than those at 26 and 27 weeks of gestation.

Figure 8: Probability (power) of exceeding established thresholds for safety outcomes. Here we consider an expected event rate of 5% with a threshold of 10% used to trigger DSMB action.

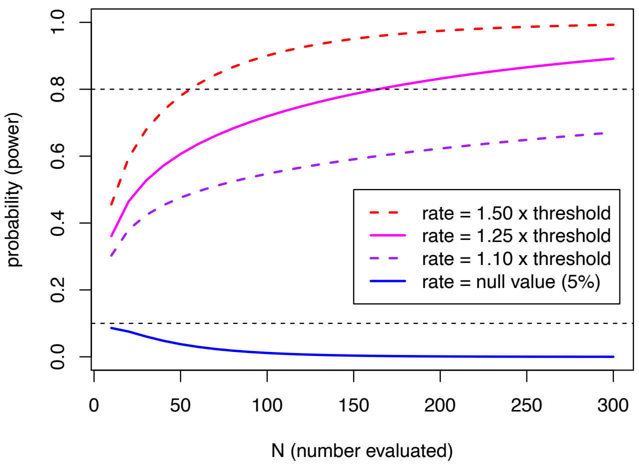

SAEs and Threshold for Action: For each SAE listed in Table 4 we have established expected rates of SAEs for the target study population, and thresholds for each treatment arm at which careful evaluation would be required by DSMB review. The DSMB would be asked to review all information associated with the line listing for the SAEs and then make a decision as to whether the study should be modified, continued or stopped. In Figure 8 we show the operating characteristics for an SAE that has an expected (normal) rate of 5%, and a threshold of 10% for review. If the true SAE rate is 1.5 times the threshold (e.g. 15%) then with 100 or more subjects there is a greater than 80% probability (power) that the observed rate of greater than the threshold would occur and trigger DSMB review. However, if the true SAE rate was only 1.25 times greater than the threshold then 150 or more subjects would be needed to have 80% power. Although a total of 10 SAEs are monitored for their absolute rate, we will not use any multiple comparison correction for continuous monitoring of safety leading to DSMB review. However, formal interim analysis comparing safety across the two study groups will account for both multiple comparisons due to multiple individual safety outcomes, and for multiple interim analyses.

SAE analysis uses a doubling of rates to trigger consideration of DSMB action irrespective of statistical significance at interim analysis, yet formal monitoring will be conducted every 6 months to evaluate the strength of statistical evidence for a difference across groups. Therefore, our proposed interim analyses will evaluate the accruing data using both formal statistical comparison in addition to consideration of absolute rates and is thus more conservative than use of only one criterion. Finally, we plan to conduct interim analyses that will also report on the specific SAEs and AEs listed in Tables 4 and 5, and will provide p-values comparing treatment groups for these safety outcomes in addition to the SAE outcomes. A medical safety officer not involved in the study will review all SAEs, and the DSMB (appointed by NINDS) will decide when they want to review each type of SAE or AE.

6.9 Formal Interim Analyses: Our primary objective for interim analysis is to allow careful and continuing analysis of safety outcomes. Specifically, we propose to conduct formal statistical analysis and inference for each SAE and AE at three interim and one final analysis time. We will continue to analyze all AE events that have occurred during follow-up, but focus interim analysis on those events that occur within the first three months of follow-up since this is the time period in which the major treatment related events would be expected. We will conduct formal interim safety monitoring analysis at approximately 25% (n=235), 50% (n=470), and 75% (n=705) and 100% of target enrollment of n=940. We plan to monitor death and will control the overall significance level using O'Brien-Fleming boundaries (net alpha=0.05 significance, accounting for three interim and one final analysis).[219] For the other 4 SAEs, we will again use sequential monitoring boundaries to control the outcome-specific family-wise error rate (using a Bonferroni corrected alpha of 0.05/5 = 0.01).

The DSMP specifies formal interim safety monitoring analysis at approximately 25% (n=235), 50% (n=470), and 75% (n=705) and 100% of target enrollment of n=940. For death, we will use O'Brien-Fleming sequential monitoring guidelines with a net alpha=0.05, and a Bonferroni corrected sequential guideline for the 10 SAE for a significance level of 0.05/10 = 0.005 to correct for multiple events. The O'Brien-Fleming sequential monitoring p-values are listed below in Table 7.

Table 7. O'Brien-Fleming p-values for interim monitoring of death and SAEs.

Enrollment	P-value for monitoring death	P-value for monitoring all other SAEs
25% (n=235)	0.000014734	0.000000000295
50% (n=470)	0.0030359	0.000038113
75% (n=705)	0.016248	0.00092412
100% (n=940)	0.030701	0.0040378

The CCC PI will be responsible for notifying the Medical Monitor of any safety events, and the Medical Monitor will notify the DSMB if indicated. All events will be reported to the DSMB in semi-annual reports.
