# Supplementary material for: Acute Kidney Injury Defined by Fluid-Corrected Creatinine in Premature Neonates: A Secondary Analysis of the PENUT Randomized Clinical Trial
Source: JAMA Netw Open. 2023 Aug 10;6(8):e2328182. doi: 10.1001/jamanetworkopen.2023.28182 (PMC10415963; doi:10.1001/jamanetworkopen.2023.28182)
Supplement: Supplement 2. — eTable 1. Definition and Distribution of AKI Status by Serum Creatinine and Fluid-Corrected Serum Creatinine AKI eFigure. CONSORT Diagram eTable 2. Comparison of Maternal And Neonatal Characteristics by Primary Outcome of Mechanical Ventilation at Post-Natal Day 14 eTable 3. Comparison of Maternal and Neonatal Characteristics by Severe Fluid-Corrected AKI Status [file jamanetwopen-e2328182-s002.pdf]

Supplemental Online Content

Starr MC, Griffin RL, Harer MW, et al. Acute kidney injury defined by fluid-corrected creatinine in premature neonates: a secondary analysis of the PENUT randomized clinical trial. *JAMA Netw Open*. 2023;6(8):e2328182. doi:10.1001/ jamanetworkopen.2023.28182

eTable 1. Definition and Distribution of AKI Status by Serum Creatinine and Fluid-Corrected Serum Creatinine AKI
eFigure. CONSORT Diagram
eTable 2. Comparison of Maternal And Neonatal Characteristics by Primary Outcome of Mechanical Ventilation at Post-Natal Day 14
eTable 3. Comparison of Maternal and Neonatal Characteristics by Severe Fluid-Corrected AKI Status

This supplemental material has been provided by the authors to give readers additional information about their work.

© 2023 Starr MC et al. *JAMA Network Open.*

eTable 1. Definition and Distribution of AKI Status by Serum Creatinine and Fluid Corrected Serum Creatinine AKI

		Fluid Corrected AKI Status		Total
		-	+	
**AKI (SCr)**	**-**	No AKI<br>597	Unveiled AKI<br>111	708
	**+**	Over Diagnosed AKI<br>13	True AKI<br>202	215
**Total**		610	313	923

AKI, Acute Kidney Injury

© 2023 Starr MC et al. *JAMA Network Open.*

eFigure 1. CONSORT Diagram.

941 subjects were enrolled in the PENUT study. We excluded five neonates who were removed from the parent study (4 died prior to receiving study drug, and one who was enrolled incorrectly). Furthermore, we also excluded 13 neonates who died on days 0, 1 or 2. Therefore, 923 neonates with sufficient data for analysis were included in this analysis.

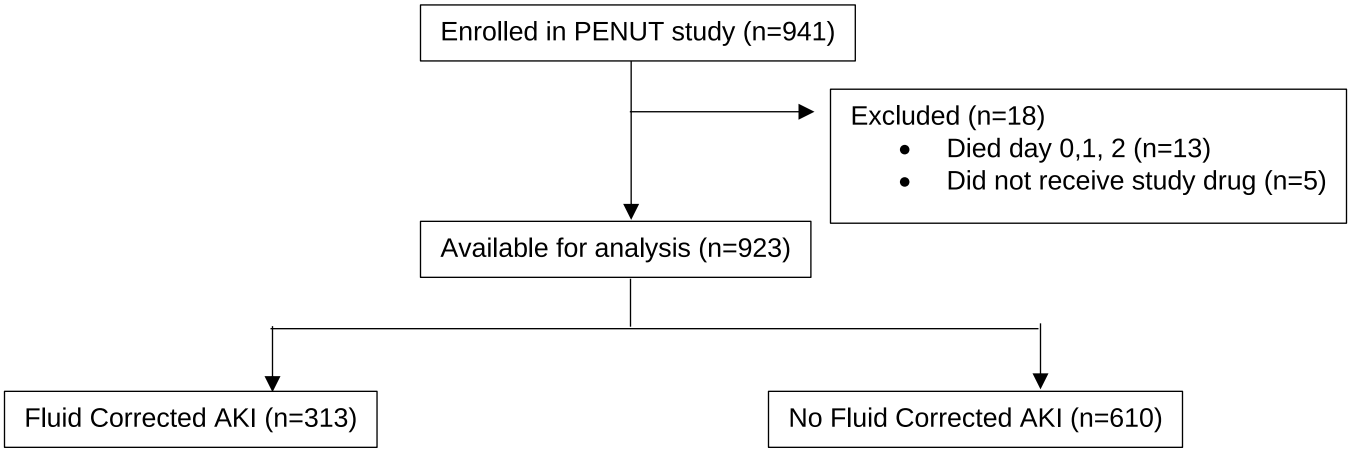

© 2023 Starr MC et al. *JAMA Network Open.*

eTable 2. Comparison of Maternal and Neonatal Characteristics by Primary Outcome of Mechanical Ventilation at Post-Natal Day 14

Characteristic, n (%)	Mechanical Ventilation at 14 postnatal days, n (%)	No Mechanical Ventilation at 14 postnatal days, n (%)	p-value[a]
n	480	410	
Epo	242 (50.4)	193 (47.1)	0.32
Male	260 (54.2)	202 (49.3)	0.14
Gestational Age, weeks			
24	191 (39.8)	26 (6.3)	<0.001
25	154 (32.1)	80 (19.5)	
26	84 (17.5)	129 (31.5)	
27	51 (10.6)	175 (42.7)	
Birth weight, g, mean (SD)	724.6 (164.1)	889.2 (172.1)	<0.001
Small size for gestational age	88 (18.4)	50 (12.3)	0.013
Apgar 1 min, mean (SD)	3.4 (2.1)	4.5 (2.3)	<0.001
Apgar 5 min, mean (SD)	5.7 (2.2)	6.8 (1.7)	<0.001
Delivery room resuscitation			
Any	471 (98.3)	392 (35.6)	0.016
Intubation	434 *90.4)	287 (70.0)	<0.001
Surfactant	275 (57.3)	191 (46.6)	0.001
Chest compressions	48 (10.0)	17 (4.1)	<0.001
Resuscitation drugs	21 (4.4)	7 (1.7)	0.023
Maternal characteristics			
Multiple gestations	119 (24.8)	114 (27.8)	0.31
Diabetes	22 (4.6)	24 (5.9)	0.39
Hypertension	43 (9.0)	25 (6.1)	0.11
Pre-eclampsia	70 (14.6)	63 (15.4)	0.74
Maternal Race			
Black	136 (28.3)	92 (22.4)	0.047
White	298 (62.1)	286 (69.8)	
Other[b]	27 (5.6)	24 (5.9)	
Unknown	19 (4.0)	8 (2.0)	
Maternal Ethnicity			
Hispanic or Latino	87 (18.1)	104 (25.4)	0.007
Not Hispanic or Latino	384 (80.0)	304 (74.1)	
Unknown	9 (1.9)	2 (0.5)	
Neonatal course			
PDA (treated)	275 (57.3)	99 (24.2)	<0.001
Severe IVH	86 (17.9)	23 (5.6)	<0.001
BPD	241 (50.2)	73 (17.8)	<0.001

BPD, Bronchopulmonary Dysplasia; Epo, Erythropoietin; IVH, Intraventricular hemorrhage; PDA, patent ductus arteriosus; SD, Standard Deviation

[a] Based on chi-square test for categorical and t-test for continuous variables

[b] Other maternal race categorization includes American Indiana or Alaska Native, Asian, Native Hawaiian or Other Pacific Islander

eTable 3. Comparison of Maternal and Neonatal Characteristics by Severe Fluid Corrected AKI Status

Characteristic, n (%)	True Severe AKI	Unveiled Severe AKI	Over Diagnosed Severe AKI	No Severe AKI	p-value[a]
n	58	43	3	819	
Epo	33 (56.9)	28 (65.1)	2 (66.7)	406 (49.6)	0.16
Male	29 (50.0)	24 (55.8)	3 (100.0)	423 (51.6)	0.37
Gestational Age, wk					
24	19 (32.8)	20 (46.5)	1 (33.3)	187 (22.8)	0.0011
25	22 (37.9)	13 (30.2)	0 (0.0)	207 (25.2)	
26	8 (13.8)	6 (14.0)	1 (33.3)	205 (25.0)	
27	9 (15.5)	4 (9.3)	1 (33.3)	220 (26.9)	
Birth weight, g, median (IQR)	715 (610-837)	710 (640-822)	916 (860-1040)	810 (670-950)	<0.001
Small size for gestational age	13 (22.8)	8 (18.6)	1 (33.3)	119 (14.6)	0.27
Apgar 1 min, median (IQR)	4 (2-6)	4 (2-6)	3 (1-7)	4 (2-6)	0.93
Apgar 5 min, median (IQR)	6 (5-7)	7 (4-8)	3 (1-8)	7 (5-8)	0.18
Delivery resuscitation					
Any	58 (100.0)	43 (100.0)	3 (100.0)	792 (96.8)	0.33
Intubation	54 (93.1)	39 (90.7)	2 (66.7)	653 (79.7)	0.024
Surfactant	238 (62.8)	38 (65.5)	2 (66.7)	413 (50.4)	0.062
Chest compressions	6 (10.3)	1 (2.3)	0 (0.0)	65 (7.9)	0.46
Resuscitation drugs	4 (6.9)	0 (0.0)	0 (0.0)	28 (3.4)	0.30
Maternal characteristics					
Multiple gestations	8 (13.8)	12 (27.9)	3 (100.0)	220 (26.9)	0.0041
Diabetes	3 (5.2)	0 (0.0)	0 (0.0)	45 (5.5)	0.45
Hypertension	7 (12.1)	4 (9.3)	0 (0.0)	59 (7.2)	0.52
Pre-eclampsia	7 (12.1)	3 (7.0)	0 (0.0)	130 (15.9)	0.32
Maternal Race					
Black	16 (27.6)	14 (32.6)	0 (0.0)	209 (25.5)	0.035
White	40 (69.0)	27 (62.8)	2 (66.7)	535 (65.3)	
Other[b]	0 (0.0)	0 (0.0)	0 (0.0)	52 (6.3)	
Unknown	2 (3.4)	2 (4.7)	1 (33.3)	23 (2.8)	
Maternal Ethnicity					
Hispanic or Latino	18 (31.0)	13 (30.2)	2 (66.7)	164 (20.0)	<0.001
Not Hispanic or Latino	40 (69.0)	29 (67.4)	0 (0.0)	646 (78.9)	
Unknown	0 (0.0)	1 (2.3)	1 (33.3)	9 (1.1)	
Neonatal course					
PDA (treated)	23 (40.0)	27 (64.3)	1 (33.3)	333 (40.7)	0.025
Severe IVH	11 (19.0)	10 (23.3)	1 (33.3)	100 (12.2)	0.065
BPD	26 (44.8)	22 (51.2)	1 (33.3)	292 (35.7)	0.12
**Outcomes**					
Mechanical Ventilation at 14 postnatal days					
High-flow cannula	6 (10.3)	4 (10.0)	1 (33.3)	118 (15.0)	<0.001
Non-invasive	6 (10.3)	3 (7.5)	1 (33.3)	271 (34.3)	
Invasive	46 (79.3)	33 (82.5)	1 (33.3)	400 (50.7)	
Hospital length of stay, median (IQR)	91 (66-107)	98 (59-117)	105 (14-116)	92 (72-114)	0.79
Mortality	9 (15.5)	7 (16.3)	1 (33.3)	83 (10.1)	0.20

BPD, Bronchopulmonary Dysplasia; Epo, Erythropoietin; IVH, Intraventricular hemorrhage; IQR, Intraquartle range, SD, Standard Deviation; PDA, Patent Ductus Arteriosus

[a] Based on chi-square test for categorical and t-test for continuous variables

[b] Other maternal race categorization includes American Indiana or Alaska Native, Asian, Native Hawaiian or Other Pacific Islander

© 2023 Starr MC et al. *JAMA Network Open.*
