# Supplementary material for: Acute Kidney Injury Defined by Fluid-Corrected Creatinine in Premature Neonates: A Secondary Analysis of the PENUT Randomized Clinical Trial
Source: JAMA Netw Open. 2023 Aug 10;6(8):e2328182. doi: 10.1001/jamanetworkopen.2023.28182 (PMC10415963; doi:10.1001/jamanetworkopen.2023.28182)
Supplement: Supplement 3. — Data Sharing Statement [file jamanetwopen-e2328182-s003.pdf]

Data Sharing Statement

Starr. Acute Kidney Injury Defined by Fluid-Corrected Creatinine in Premature Neonates. *JAMA Netw Open*. Published August 10, 2023. doi:10.1001/jamanetworkopen.2023.28182

Data
Data available: Yes
Data types: Deidentified participant data, Data dictionary
How to access data: De-identified individual participant data are available through the NINDS Data Archive: https://www.ninds.nih.gov/Current-;Research/Research-Funded-NINDS/Clinical-Research/Archived-Clinical-Research-Datasets. The data is de-identified and a limited access data is available through a request form on that page. Data dictionaries, in addition to study protocol, the statistical analysis plan, and the informed consent form are included. The data are available to researchers who provide a methodologically sound proposal for use in achieving the goals of the approved proposal.
When available: With publication

Supporting Documents
Document types: Informed consent form
How to access documents: Data dictionaries, in addition to study protocol, the statistical analysis plan, and the informed consent form are included.
https://www.ninds.nih.gov/Current-;Research/Research-Funded-NINDS/Clinical-Research/Archived-Clinical-Research-Datasets
When available: With publication

Additional Information
Who can access the data: The data are available to researchers who provide a methodologically sound proposal for use in achieving the goals of the approved proposal.
Types of analyses: As above
Mechanisms of data availability: https://www.ninds.nih.gov/Current-;Research/Research-Funded-NINDS/Clinical-Research/Archived-Clinical-Research-Datasets.
